# The current state of international research on the effectiveness of school nurses in promoting the health of children and adolescents: An overview of reviews

**DOI:** 10.1371/journal.pone.0275724

**Published:** 2023-02-22

**Authors:** Silke Pawils, Susanne Heumann, Sophie Alina Schneider, Franka Metzner, Daniel Mays

**Affiliations:** 1 Department of Medical Psychology, University Medical Center Hamburg-Eppendorf, Hamburg, Germany; 2 Professorship for Educational Science with a Focus on Special Education ("Emotional and Social Development"), University of Siegen, Siegen, Germany; University of Eastern Finland: Ita-Suomen yliopisto, FINLAND

## Abstract

**Objective:**

School nurses are engaging worldwide to promote and protect children’s health. Many researchers who examined the effectiveness of the school nurse criticized the inadequate methodology employed in many of the studies. We therefore carried out an evaluation on the effectiveness of school nurses based on a rigorous methodological approach.

**Methods:**

In this overview of reviews we performed an electronic databank search and global research results on the effectiveness of school nurses were sought. We identified 1,494 records through database search. Abstracts and full texts were screened and summarized using the dual control principle. We summarized the aspects of quality criteria as well as the significance of the effectiveness of the school nurse. In the first step, k = 16 systematic reviews were summarized and evaluated following the AMSTAR-2 guidelines. In a second step, j = 357 primary studies included in these k = 16 reviews were summarized and assessed following the GRADE guidelines.

**Results:**

Research results on the effectiveness of school nurses show that school nurses play a key role in improving the health of children with asthma (j = 6) and diabetes (j = 2), results on combating obesity are less certain (j = 6). The quality of identified reviews is mostly very low with only six studies of medium quality, of which one identified as a meta-analysis. A total of j = 289 primary studies were identified. Approximately 25% (j = 74) of identified primary studies were either randomized controlled trials (RCT) or observational studies, of which roughly 20% (j = 16) had a low risk of bias. Studies with physiological variables such as blood glucose or asthma labeling led to higher quality results.

**Conclusion:**

This paper represents an initial contribution and recommends further evaluation of the effectiveness of school nurses, particularly in the areas of mental health or children from low socioeconomic backgrounds. The general lack of quality standards in school nursing research should be integrated into the scientific discourse of school nursing researchers to provide robust evidence for policy planners and researchers.

## 1 Introduction

School nurses are medically trained professionals who work in both the school and healthcare sectors, with the aim of making the school a health-promoting environment for teachers and pupils [1–3]. School nurses play a pivotal role in improving the health and well-being of children and adolescents by providing health promotion, health counseling, referral to other sources of help, active treatment, education, family support, care coordination, and multiagency work [1,4–6]. School nurses have a wide range of roles and responsibilities that cover three core aspects of school health 1) health literacy, 2) medical care, and 3) health promotion [7–9]. These three areas of school nursing complement each other in terms of the common goal of making the school a health-promoting environment but differ in their approaches and strategies to achieve this goal. Health literacy has the aim of enabling students and teachers to find, understand, evaluate, and apply health information to health-related decisions in order to maintain or improve health and quality of life [10,11]. Health literacy holds the educational perspective with the goal of knowledge gain. In contrast, health promotion and medical health care share the aspect of medically oriented interventions but differ from one another in their perspective on health aspects. Medical health care at schools takes the pathogenetic perspective. Here, the focus is on assessing risks for disease progression and treatment of specific diseases that can be positively influenced by specific school-based interventions. Health promotion in schools, on the other hand, argues salutogenetically. Here, the focus is on the development and maintenance of health in children and adolescents. This can be achieved through resource-strengthening measures at schools, promoting healthy behaviors such as sports and nutrition and through preventive care services, e.g. care coordination by school nurses [11,12]. All three aspects of school health cannot be clearly distinguished from each other.

Most international research on school nurse interventions addressing the three aspects of school health originated in Anglo-Saxon countries [13–15] where the school nurse was first introduced and where most research has been conducted. Many of these studies evaluate school-nurse led interventions and refer to the number of days absent due to medical conditions [13,16], health risk factors such as obesity [17,18] and cigarette consumption [19,20], children with asthma symptoms [21,22], mental health conditions [23,24], the management of chronic diseases [3,25] or preventing various forms of child abuse [26–28]. Apart from more positive health outcomes for children, the support provided by the school nurse may lessen the burden on teachers confronted with such problems in the classroom [29–31].

The importance of the school nurse came particularly apparent when the COVID-19 pandemic hit and new health-related challenges for both pupils and teachers arose. Recent studies focused on an examination of the role of school nurses and their valuable input particularly on the question of the wisdom of closing schools or keeping them open [32]. Additionally studies also addressed how their work could help to minimize community-wide risk [33] through improved hygiene concepts [34] and effective immunization programs [35].

Besides the aforementioned health-related benefits, studies calculated financial savings in health care for society as a whole [36,37] by involving school nurses at primary and secondary schools. Wang and Vernon-Smiley [38] estimated that in one year alone, for every dollar invested in their program in the US, $2.20 is saved. Binder [36] investigated whether poor parental health awareness, poor integration in society as well as rising numbers of children with chronic health issues could ultimately cause higher follow-up costs than investing in prevention.

While there is a large body of research available about school nursing, it becomes apparent that despite the abundance of literature on the subject, scientifically sound conclusions regarding the effectiveness of school nurses are anything but clear-cut. Researchers [20,21] have repeatedly criticized the lack of methodological quality and the resulting lack of robust, meaningful research findings regarding the effectiveness of school nurses. The main points of criticism are the lack of evidence-based, quantitative data with randomized controlled trial designs and the predominantly descriptive study designs [39].

We see an urgent need to address this increasingly vocal criticism of school nurse research and to organize the miscellany of research findings. For this reason, we conducted an *overview of reviews*, the first of its kind in school nurse research. This paper is a response to the prevailing criticism of poor study quality, which is why *reviews* and *primary studies* are subject to strict methodological guidelines. By introducing methodological standards, our aim is to ensure clinical validity regarding school nurse effectiveness.

Our aim is to critically assess the current state of the relevant literature by applying rigorous and valid quality criteria. Well-established quality assessment tools such as AMSTAR-2 and GRADE guidelines were chosen and adapted to our purposes, as these tools are widely recognized among researchers and overcome the criticisms aimed at school nursing research. This paper will review studies which are in line with well-established methodological guidelines to arrive at sound conclusions on interventions and on meaningful results on health outcomes.

## 2 Materials and methods

**Fig 1:**
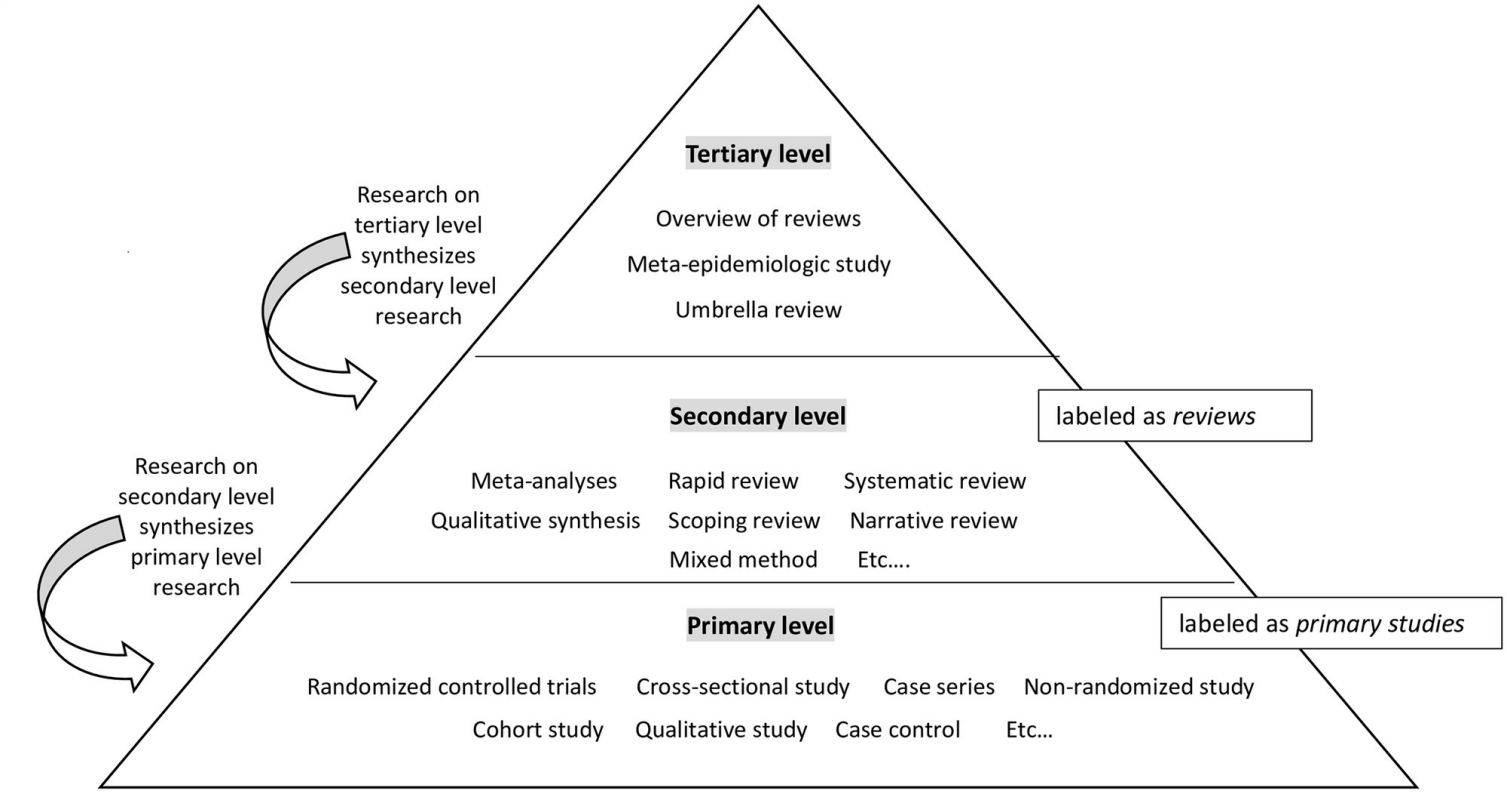
Hierarchy of research levels. *Notes*. On tertiary level (e.g., overview of reviews) we synthesize studies on secondary level. On secondary level (e.g., meta-analysis) synthesize studies on primary level.

Studies are divided into three categories: primary, secondary and tertiary level. Primary level studies are e.g. randomized controlled trial designs or observational studies. For the sake of simplicity, studies on primary level are called *primary studies* in this paper. Secondary level studies are e.g. meta-analyses or systematic reviews, and are called *reviews*. Tertiary level studies are e.g. *overview of reviews* or umbrella reviews. The present paper is an *overview of reviews*. *Overview of reviews* encompass *reviews*, which in turn encompass *primary studie* (Fig 1).

The methodological approach of conducting an *overview of reviews* is based on the guidelines of Biondi-Zoccai [40], who describe the research process and tools to summarize evidence relevant for policymakers in evidence-based medicine. Due to the lack of guidelines for *overview of reviews* in the educational or psychological field, this paper applies (to our knowledge for the first time in school nurse research) clinically relevant standards, which is intended to create solid and meaningful results and is a prerequisite for optimal decision-making. The methodological research standards were based on Zawacki-Richter, Kerres [41], who describe the methodological procedure of systematic reviews in the educational research field. The *overview of reviews* differs from *reviews* in only a few respects (e.g. databases are searched exclusively for *reviews*) [40]. For this reason, this study also followed the Preferred Reporting Items for Systematic reviews and Meta-Analyses (PRISMA) [42], including the recommended Cochrane Checklist (Attachment 1). The quality of included *reviews* was assessed according to the validated scale Assessment of Multiple Systematic Reviews 2 (AMSTAR-2) [43]. The strict quality rating was adapted to allow the reader a comparison of our *reviews*. Biondi-Zoccai [40] recommended an analysis of *primary studies* that were summarized in each *review*, in order to be able to make authoritative statements regarding its informative value. To assess the body of evidence of *primary studies* the Grading of Recommendations Assessment, Development and Evaluation (GRADE) [44] was used. GRADE provides instructions for assessing the strength of evidence for each outcome in a *review* [44]. In our work, the GRADE assessment remains at descriptive level and risk of bias, imprecision, indirectness of evidence, publication bias and impact are described. Magnitude of effects, dose-response relations and the impact of residual confounding were removed, as these criteria are not relevant for studies from the educational and psychological field.

Our approach to this work is explained in a protocol that was created *a priori* and continuously updated during the research process and uploaded to the PROSPERO website on 08.02.2021 [45]. The protocol can be viewed on PROSPERO with the registration number CRD42021235152. All relevant data are within the manuscript and its Supporting Information files.

### 2.1 Inclusion and exclusion criteria

We outlined our inclusion and exclusion criteria in terms of the PICOS format. We included studies examining children aged 5 to 21 (IC1), with either the school nurse himself/herself being the intervention or a school nurse-led health program as the intervention (IC2). The criterion of the comparison group was not applicable for this work. Studies examining the effect of various health outcomes, school attendance, academic achievements, risky and difficult behavior in the school setting were included (IC3). Only research in English and German and *reviews* were searched (IC4) with no date restrictions. We excluded *primary studies* and studies with interventions that were not conducted by at least one school nurse or in the school setting. Studies without specific health outcomes for school children, as well as recommendations for school nurses, were also excluded (Table 1).

**Table 1:** Inclusion and exclusion criteria based on PICOS scheme.

INCLUSION CRITERIA (IC)		
*IC1*	*P* *opulation*	
	IC 1.1	School children aged between 5 and 21 Healthy children Disadvantaged children Children with chronic illnesses
	IC 1.2	Teachers in primary schools, middle schools and high schools (and other types of schools based on countries) Graduated teachers in training Teachers with at least one lesson per week
	IC 1.3	School nurses who work in primary schools, middle schools or high schools Specially trained nurses with educational focus Working in schools as well as in general health care
*IC2*	*I* *ntervention*	
	IC 2.1	Effectiveness of the school nurse in dealing with chronic illnesses or disabilities (e.g. in regards to medication, care, special hospital observations or in handling acute emergencies) in implementing recommendations (e.g. regarding seeing or hearing aids, occupational therapy or psychotherapy) in dealing with signs of violence or risks to children’s well-being in contributing to dental prophylaxis in assisting specific health needs or infectious diseases (e.g. with infectious or food-based diseases)
	IC 2.2	Effectiveness of the interventions performed by the school nurses Anti-aggression programs Programs on a healthy diet Meditation (release of stress and anxiety) Prevention programs
*IC3*	*O* *utcome*	
	IC 3.1	Physical and psychological health outcomes for children and teachers
	IC 3.2	Outcomes regarding health literacy of children and teachers
*IC4*	*S* *tudy Design*	
	IC 4	Study Design: Study designs on secondary level, e.g. meta-analysis, mixed method systematic review, narrative review, qualitative synthesis, scoping review, systematic review, rapid review
*IC5*	*Publication*	
	IC 5.1	peer-reviewed reviews (journal article)
	IC 5.2	published in English or German language
EXCLUSION CRITERIA (EC)		
*EC1*	*Participants*	
	EC 1.1	Parents
	EC 1.2	Children younger than 5 years old or older than 21 years old
	EC 1.3	All professions in the school context except for teachers and school nurses (e.g. social workers, school psychologists, doctors)
*EC2*	*Interventions*	
	EC 2.1	All regarding the role of school nurses Financial feasibility
	EC 2.2	All interventions that do not examine the effectiveness of the intervention performed by the school nurse
	EC 2.3	„Best Practice" for school nurses
	EC 2.4	Interventions that do not examine the effectiveness of the school nurse
	EC 2.5	Framework for school nurses
	EC 2.6	Challenges for school nurses
	EC 2.7	Curriculum
*EC 3*	*Outcome*	
	EC 3.1	Outcomes that do not regard the physical or psychological health of children and teachers
	EC 3.2	Outcomes that do not regard the impact on health literacy
	EC 3.3	Pros and cons for school nurses
	EC 3.4	Implications for school nurses
	EC 3.5	How school nurses benefit
	EC 3.6	How parents benefit
	EC 3.7	How school psychologists benefit
	EC 3.8	No health outcome
*EC 4*	*Study design*	
	EC 4	Study designs on primary level, e.g. case study, case series, case-control-study, cohort study, cross-sectional study, qualitative study, pre-clinical study, randomized controlled trial

### 2.2 Information sources

We conducted a search of peer-reviewed literature in Medline, Cochrane Library, Cinahl, Web of Science, Scopus, PubMed, Subject portal Pedagogy [Fachportal Pädagogik], Educational Resource Information Center (ERIC) and German National Bank Catalogue [Katalog der deutschen Nationalbank] from November 2020 to January 2021. Additionally, we searched Google Scholar, reference lists, and also contacted leading researchers in the field of school nursing for additional overviews, finishing the search process in February 2021.

### 2.3 Search strategy

Before starting the search process, we conducted a pilot study of the scope of school nurse-related literature. For this purpose, we used the Medline database as a trial database, analyzing the first 150 results using the search term "school nurs*". The results of this pilot study were presented to an expert panel of eight members, optimizing our methodology and focus (e.g. deciding to only include *reviews*). In addition, the PROSPERO database was searched to exclude possible content overlap with studies not yet published [45]. Following the suggestion of Zawacki-Richter, Kerres (41), a record log was initiated to develop a search string (Table 2). Our final search string (“School-nurs* (only in title)” AND (“Review* OR meta-analysis”) NOT (“Barrier* OR framework OR role* (only in title))) was searched in 9 databases and had to be adapted for the database ERIC (“school nurse” AND “review” OR “reviews” NOT “barrier” NOT “barriers” NOT “framework” NOT “frameworks” NOT “role” NOT “roles”) and for German databases (“Schulgesundheitsfachkraft” OR “Schulgesundheitspflege”, which translates into school nurse).

**Table 2:** Terms used in systematic database literature search.

Category	PubMed, Medline, Web of Science, Cochrane, CINAHL, Scopus	ERIC*
A: School Nurse	school-nurs* (only in title)	“school nurse”
B: Method	review*OR meta-analysis	Review OR reviews
C: Excluded terms	Barrier* OR role*OR framework	NOT barrier NOT barriers NOT framework NOT frameworks NOT role NOT roles

*Notes*. Categories were combined into a search string as follows: A and B NOT C.

*The database ERIC did not recognize the word “meta-analysis”. Therefor this search term was removed. ERIC does not have the option to use “*” to show all endings to a word. The hyphen between “school” and “nurse” showed no results. To avoid search results with only “school” or only “nurse” ERIC gives the option of enclosing a term in quotation marks. For this reason, our search string had to be adapted.

Additionally, reference lists were searched (FM), Google Scholar was searched using the terms "school nurse and review" (SH) and the National Association of School Nursing and leading researchers (n = 9) in school nursing research were contacted (SH and SS).

### 2.4 Screening and study selection

Citations identified from the systematic search were exported to EndNote (EndNote 20.1, Bld 12060), a reference management tool. Duplications were removed and two independent reviewers (SH and FM) screened all titles and abstracts using the inclusion and exclusion criteria, adding an explanation for the exclusion of the excluded references. Articles that were labelled as “excluded” by both researchers were removed, while articles that received conflicting votes (ineligible vs. potentially or probably eligible) were discussed and a consensus was reached. Interrater reliability was calculated using IBM SPSS 23 (IBM Corp., Armonk, NY). The agreement rate was measured by determining the percentage of the sum of all matching “included” and “excluded” references, where the total number of references assessed equalled 100%. The same two reviewers (SH and FM) screened the full texts of all the probably eligible articles using the same inclusion and exclusion criteria. If consensus was not possible during the screening of title and abstract or full text screening, a third or fourth reviewer (SP or DM), who had the casting vote, would have been asked to independently screen the article. However, this was never necessary as consensus was always reached.

### 2.5 Data extraction process of *reviews*

The selection of characteristics to be extracted from reviews was discussed with the research team, consisting of five members, and unanimous agreement was reached. Data items included author, year, country, time-period covered, type of *review*, number of *primary studies* included, subject-matter and summary of findings. One reviewer (SH) and an assistant researcher (SS) independently extracted data from each study and were generally in agreement.

### 2.6 Data extraction process of *primary studies*

**Fig 2:**
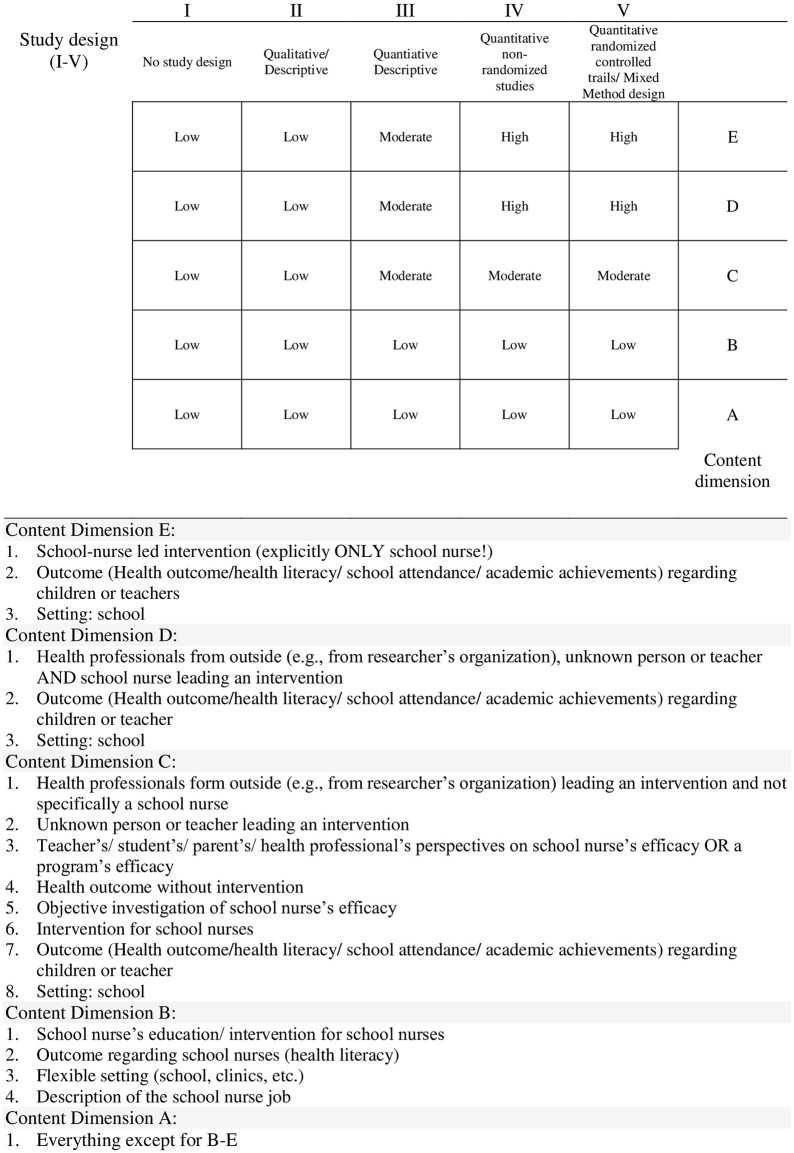
Matrix for categorization of content dimension and study design.

In order to extract data from *primary studies*, they first had to be identified in the respective *reviews*. Once identified, researchers exported references into EndNote, removed duplications, and searched full texts. The selection of characteristics to be extracted was discussed with the same research team, and unanimous agreement was reached. Data items included author, year, country, studied population, research design according to the Mixed Method Appraisal Tool (MMAT) [46], main topic, study subject-matter, intervention, data collection, outcome, and information value. The same reviewer (SH) and assistant researcher (SS) independently extracted data from each *primary study*. Due to the huge number of *primary studies*, references had to be divided up, so that no data extraction agreement could be measured. To counteract bias, the data extraction of the respective other was checked selectively. The description of *primary studies* is the basis for further data analysis and is discussed in the results. Due to the heterogeneity and the large and hence unmanageable amount of data, an additional criterion (information value) was added, which requires further explanation: This criterion represents a rating based on two dimensions: a) the study design used and b) the content covered. A matrix was developed to categorize each study. The first dimension represents the quality of the study design (I-V), based on the MMAT criteria. The second dimension classifies the relevance of the content (A-E), depending on the extent to which the *primary study* examines the effectiveness of school nurses (Fig 2).

### 2.7 Assessment of study quality of *reviews* based on AMSTAR-2

The AMSTAR-2 checklist [43] was used to assess the quality of *reviews*. One reviewer (SH) assessed all *reviews*, and the assistant researcher (SS) duplicated the appraisal with 80% agreement. AMSTAR-2 is a critical appraisal tool for *reviews*, such as meta-analysis, that include *primary studies* covering healthcare interventions. Because school nurse research is mainly comprised of descriptive literature, *reviews* included in this study cannot meet the strict guidelines according to AMSTAR-2. Despite these strict guidelines, we decided to differentiate between the quality of *reviews* by slightly adjusting the AMSTAR-2 criteria.

The following clusters were assessed): A) Research question & selection process, B) assessment of included *primary studies*, C) interpretation of results, and D) report of potential source of conflict.

Each cluster (A-D) consists of criteria which in turn contains sub-categories. Depending on the percentage of sub-criteria met in a criterion, numbers 0–4 were assigned for each quartile, with “0” no sub-criterion and “4” over 75% of sub-criteria met. The numbers in each category were then added together. The maximum score, 48 points, represents the best possible quality of *reviews*. The classification into low, medium and high quality is determined by dividing the maximum score by 3, so that studies with more than 32 points are of high quality, studies with 16 to 31 points are of medium quality and studies with less than 16 points are of low quality.

As a result, *reviews* were graded according to the number of sub-criteria actually met, and not downgraded if one sub-criterion was missing, and *reviews* that would have been downgraded under normal circumstances because a requirement was not met would in our study only be downgraded if less than 75% of the required sub-categories had not been met (Table 3).

**Table 3:** Adapted rating of the quality of *reviews*, based on AMSTAR-2 [43].

Categorisation of the subcategories that apply to the corresponding rating (0–4)		Categorisation of the total score to the quality rating (low, medium, high)	
Percentage of sub-criteria met (%)	Category	Overall score	Category
0%	0	<16	Low
1–24%	1	16–32	Moderate
25–49%	2	>32	High
50–74%	3		
75–100%	4		

### 2.8 Assessment of body of evidence of *primary studies* based on GRADE

The body of evidence was only conducted for *primary studies* with randomized controlled trials (RCTs) or observational studies, i.e. non-randomized studies (Obs), based on the GRADE assessment tool [44]. Because information relevant to the assessment, such as the confidence interval, was often not included in *primary studies*, the body of evidence remained on a descriptive level. Relevant characteristics to evaluate the body of evidence such as the indirectness of evidence (population studied, type of intervention, measures and desired measures), publication bias, risk of bias, imprecision (number of participants, confidence interval) and impact are described for each health outcome.

### 2.9 Assessment of risk of bias

Our assessment of the level of effectiveness and the resulting recommendations are less credible if the studies have significant limitations, such as the risk of bias (RoB) [44]. For this reason, we assessed the RoB for RCTs and Obs. All other study designs may not be applicable for RoB assessment. The GRADE handbook [47] for grading the quality of evidence was used as a guideline and limitations that influence the risk of bias were identified. The magnitude of an effect decreases when studies suffer from major limitations that are likely to lead to a biased assessment of the intervention. Lack of allocation concealment, lack of blinding, incomplete accounting of patients and outcome events, selective outcome reporting, failure to develop and apply appropriate eligibility criteria (inclusion of control population), flawed measurement of both exposure and outcome, failure to adequately control confounding and incomplete or inadequately short follow-up were considered when assessing the risk of bias for each RCT or Obs study.

**Fig 3:**
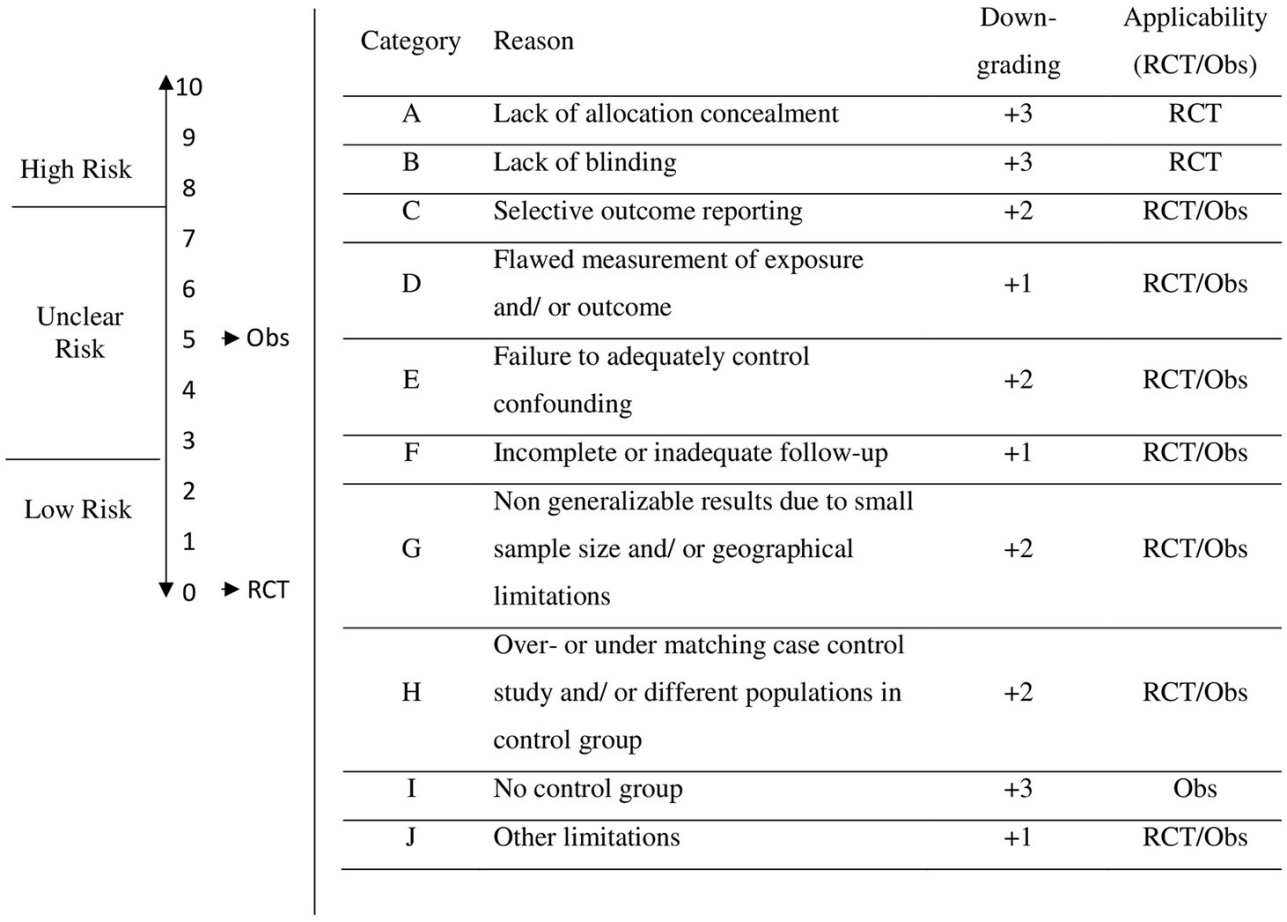
Risk of bias assessment based on GRADE assessment. *Note*. Starting point for RCTs is “Low Risk” with 0 points. Starting point for Obs is at “Unclear Risk” with 5 points. For each limitation, the corresponding downgrade (+1, +2, +3) is made on the scale (0 to 10), which results in the categorization of the risk of bias (Low, Unclear, High). Downgrading scores and categorization to low, unclear, and high risk were defined by authors.

Authors of this study developed a scale to determine the degree of risk of bias: low (0 to 2 points), unclear (3 to 7 points) and high risk (8 to 10 points). Starting point for RCTs is “Low Risk” with 0 points. Starting point for Obs. is at “Unclear Risk” with 5 points. For each limitation, the risk of bias increases by the corresponding grade (+1, +2, +3) on the scale (0 to 10), which results in the categorization of the RoB (Low, Unclear, High) (Fig 3).

### 2.10 Narrative synthesis of results

During the evaluation of the pilot study, we found that *primary studies* and *reviews*, on the school nursing subject-matter were qualitatively deficient, in the sense that hardly any meta-analyses were conducted, and most studies used descriptive designs rather than RCTs or Obs. In order to find out to what extent conclusive statements can be made regarding the effectiveness of school nurses, a comprehensive and transparent analysis of the studies was conducted, using well-validated and acknowledged clinical research methods, such as the AMSTAR-2 [43] and GRADE assessment tool [44].

The quality assessment of *reviews* was based on AMSTAR-2 criteria, whereby the scaling and subsequent grading of the quality of *reviews* was adapted by the research team (see 2.6). The body of evidence of *primary studies* was based on GRADE recommendations, and the evaluation did not go beyond a descriptive level (see 2.8). Characteristics to evaluate the body of evidence are study design, the measurement instrument and the characteristic being measured, publication bias, risk of bias, number of subjects, confidence interval, effect size, and comparability of population groups and interventions. These characteristics were classified for each health outcome.

## 3 Results

### 3.1 Study selection

**Fig 4:**
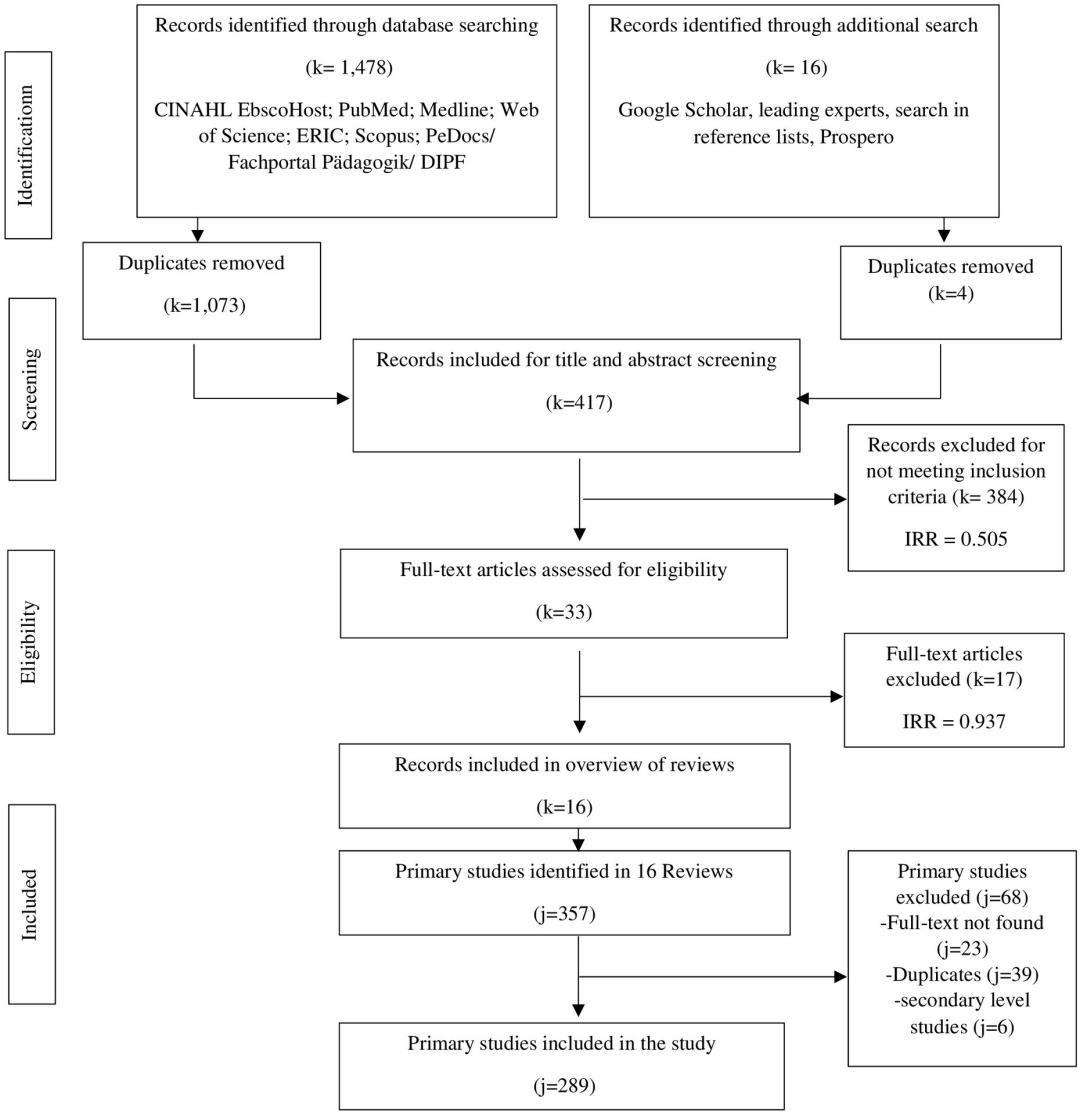
PRISMA flow chart [42] for *reviews*. *Notes*: k = *reviews*; j = *primary studies*. A search f 9 databases identified 1,478 articles. The additional search yielded 16 more articles. After subtracting duplicates (k = l,077), abstract and title of 417 articles were screened. 33 articles met the inclusion criteria (k = 385 excluded). After full texts of the 33 articles were screened, 16 articles met the inclusion criteria (k = 17 excluded). *Primary studies* identified in 16 reviews were identified (j = 357). After subtracting duplicates (j = 39) and excluding *reviews* (j = 6), and a study that could not be found (j = l), 311 *primary studies* were identified, and full texts were searched for. Further 23 *primary studies* had to be excluded as full texts could not be found, which totaled in 289 included *primary studies* in this *Overview of Reviews*.

**Fig 5:**
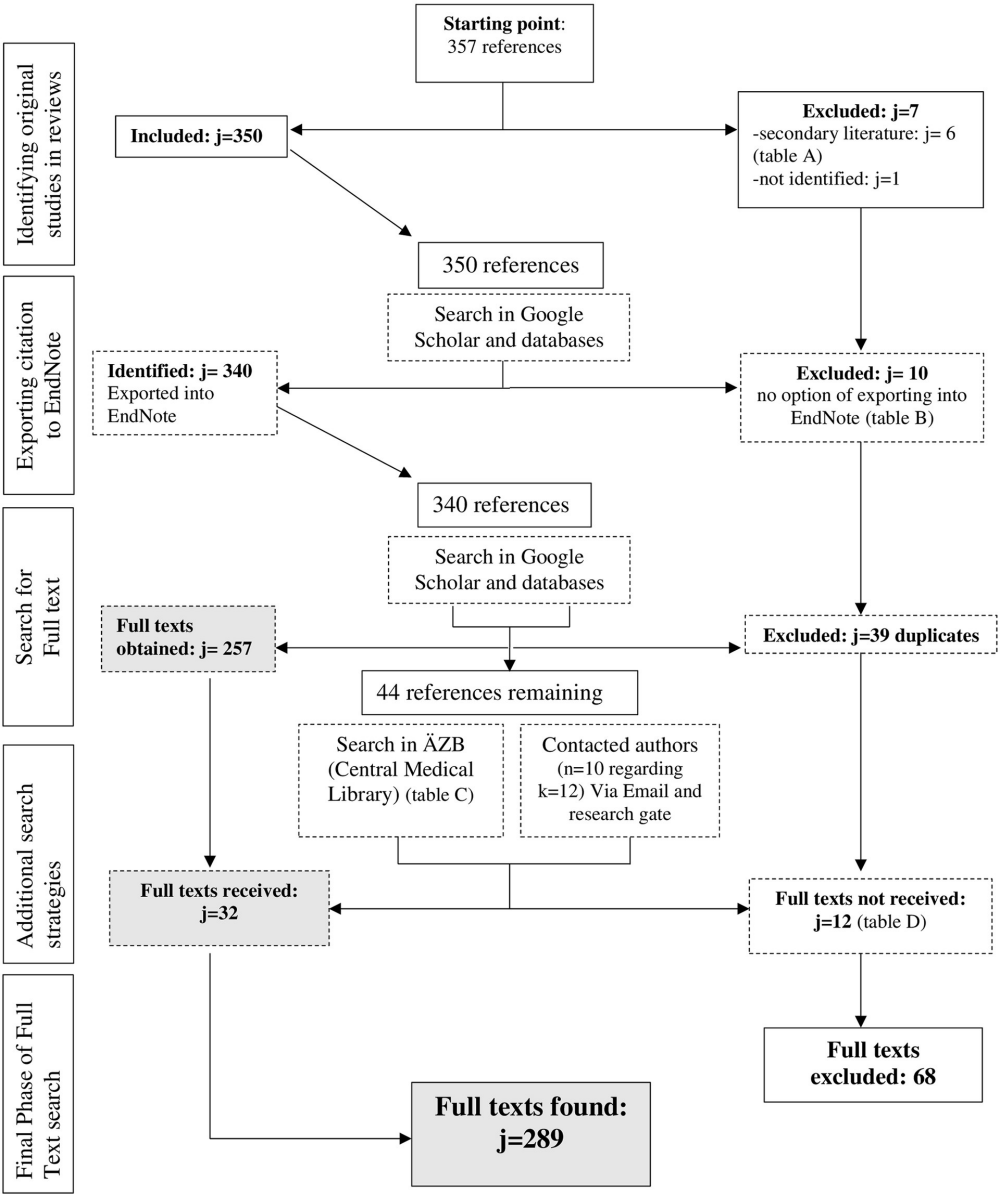
Flow chart of search process for full texts of *primary studies*.

The study selection for this *overview of reviews* consisted of two parts: First, *reviews* were searched (Fig 4), later full texts of *primary studies* included in each *review*, were searched (Fig 5).

#### 3.1.1 *Selection of* reviews

In the first part of the study selection, 9 databases were searched identifying 1,478 studies, and an additional search yielded further 16 references. After removing duplications (1,077), two independent researchers screened titles and abstracts of the remaining 417 studies (interrater reliability = 0.505; agreement rate: 92%). Records that did not meet the inclusion criteria were excluded (384). The remaining 33 full texts were screened to assess eligibility (interrater reliability = 0.760; agreement rate: 88%). At the end of the first part of the study selection process, 16 records were included in the *overview of reviews*. The 17 excluded full texts can also be found in Table 4.

**Table 4:** List of excluded reviews after full text screening (k = 17).

	Reference	Reason for exclusion	PICOS criteria
1	Aguado, V. C. (2017). La enfermera escolar: comunicación eficaz para la prevención y detención del acoso escolar = The school nurse: effective communication for prevention and arrest of bullying. *Revista Española de Comunicación en Salud*, *8*(2), 247–253.	Interventions that do not examine the effectiveness of the school nurse	EC7
2	Aronowitz, S. V., Kim, B., & Aronowitz, T. (2021). A mixed-studies review of the school-to-prison pipeline and a call to action for school nurses. *The Journal of School Nursing*, *37*(1), 51–60.	Outcomes that do not relate to the physiological and psychological health of students and teachers	EC11
3	Bradley, B. J. (1998). Establishing a research agenda for school nursing. *Journal of School Health*, *68*(2), 53–61.	Outcomes that do not relate to the physiological and psychological health of students and teachers	EC11
4	DeBell, D. (2006). School nurse practice: a decade of change. *Community Practitioner*, *79*(10), 324.	Interventions that do not examine the effectiveness of the school nurse	EC7
5	DeSocio, J., & Hootman, J. (2004). Children’s mental health and school success. *The Journal of School Nursing*, *20*(4), 189–196.	No examination of school nurse-led interventions	EC5
6	Dosa, N., & Ilardi, D. (2003). An opportunity for school nurses and pediatricians to collaborate. *School nurse news*, *20*(5), 16–22.	Full text not found	other
7	Forward, C. (2012). Measuring the effectiveness of school nursing interventions: A review of outcome tools. *British Journal of School Nursing*, *7*(10), 490–500.	Health outcomes for students or teachers wasn’t subject matter	EC11
8	Johnson, T., Weed, L. D., & Touger-Decker, R. (2012). School-based interventions for overweight and obesity in minority school children. *The Journal of School Nursing*, *28*(2), 116–123.	No examination of school nurse-led interventions	EC5
9	Markkula, V., & Muhli, U. H. (2013). Diskursen om den svenska skolsköterskans hälsostödjande arbete i kvalitativ forskning: En kvalitativ metasyntes. *Vård i norden*, *33*(2), 22–27.	Interventions that do not examine the effectiveness of the school nurse	EC7
10	McCabe, E. M., McDonald, C., Connolly, C., & Lipman, T. H. (2019). A review of school nurses’ self-efficacy in asthma care. *The Journal of School Nursing*, *35*(1), 15–26.	No health outcome for students or teachers of interest	EC11
11	Quinn, B. L., Lee, S. E., Bhagat, J., Holman, D. W., Keeler, E. A., & Rogal, M. (2020). A retrospective review of school nurse approaches to assessing pain. *Pain Management Nursing*, *21*(3), 233–237.	Not secondary level research	EC19
12	Ravenna, J., & Cleaver, K. (2016). School nurses’ experiences of managing young people with mental health problems: A scoping review. *The Journal of School Nursing*, *32*(1), 58–70.	No examination of school nurse-led interventions	EC5
13	Shannon, R. A., Bergren, M. D., & Matthews, A. (2010). Frequent visitors: Somatization in school-age children and implications for school nurses. *The Journal of School Nursing*, *26*(3), 169–182.	No examination of school nurse-led interventions	EC5
14	Strunk, J. A. (2008). The effect of school-based health clinics on teenage pregnancy and parenting outcomes: An integrated literature review. *The Journal of School Nursing*, *24*(1), 13–20.	No examination of school nurse-led interventions	EC5
15	Taylor, C., & Bailey, V. (2017). Nurse prescribing: An essential requirement or an expensive luxury for school nurses?. *British Journal of School Nursing*, *12*(7), 346–352.	Not secondary level research	EC19
16	Vessey, J. A., & Founding Oversight Board Members of MASNRN. (2007). Development of the Massachusetts School Nurse Research Network (MASNRN): A practice-based research network to improve the quality of school nursing practice. *The Journal of School Nursing*, *23*(2), 65–72.	Interventions that do not examine the effectiveness of the school nurse	EC7
17	Weismuller, P. C., Grasska, M. A., Alexander, M., White, C. G., & Kramer, P. (2007). Elementary school nurse interventions: Attendance and health outcomes. *The Journal of School Nursing*, *23*(2), 111–118.	Not secondary level research	EC19

#### 3.1.2 *Selection of* primary studies

In the second part of the study selection process, 357 *primary studies* included in each *review* were identified. Some *primary studies* were used in more than one *overview* (S1 Table). Among 357 *primary studies*, there were 6 *reviews* (Table 5a), 1 missing literature and 10 not exportable literature (Table 5b) that had to be excluded, leaving 340 *primary studies* which were exported into EndNote (EndNote 20.1, Bld 12060; j = 340). A total of 39 duplications were removed. 44 references were ordered via the central medical library [Ärztliche Zentral Bibliothek] (Table 6). A further 12 *primary studies* had to be excluded, as full texts could not be found (Table 5c), which left 289 *primary study* full texts included for our data analysis of *primary studies*.

**Table 5:** 5a, 5b, 5c. List of excluded studies.

	*5a*. *Reviews* identified in *Reviews* (k = 6)	*Review*
1	Barlow J., Brown S.S. & Fletcher J. (1997) Systematic Review of the School Entry Medical Examination. Health Services Research Unit, Oxford.	Wainwright et al. (2000) [39]
2	Wainwright, P.; Thomas, J.; Jones, M. (2000). Health Promotion and the Role of the School Nurse: a Systematic Review. Journal of Advanced Nursing, 5, 1083–1091.	Schmitt & Goerres (2012) [48]
3	DeBell, D. (2006). School Nurse Practice: a Decade of Change. Community Practitioner, 10, 324–327.	Schmitt & Goerres (2012) [48]
4	Edwards, D., Noyes, J., Lowes, L., Spencer, L. H., & Gregory, J. W. (2014). An ongoing struggle: a mixed-method systematic review of interventions, barriers and facilitators to achieving optimal self-care by children and young people with type 1 diabetes in educational settings. BMC pediatrics, 14(1), 1–27.	Stefanowicz & Stefanowicz (2018) [49]
5	Kelo M, Martikainen M, Eriksson E. Self-care of school-age children with diabetes: an integrative review. J Adv Nurs. 2011;67:2096–108. doi: 10.1111/j.1365-2648.2011.05682.x.	Stefanowicz & Stefanowicz (2018) [49]
6	Maughan, E. (2003). The impact of school nursing on school performance: A research synthesis. The Journal of School Nursing, 19(3), 163–171	Schmitt & Goerres (2012) [48]
	5b. References that could not be exported to EndNote (k = 10)	*Reviews* that included reference
1	Bergren M.D. & Mehl R. (1995a) Electronic Communication Part 111. Journal of School Nursing 11, 7±9.	Wainwright et al. (2000) [39]
2	British Paediatric Association. (1995) Health Needs of School Age Children. British Paediatric Association, London.	Wainwright et al. (2000) [39]
3	Cohen P. (1997) School nurses: in a class of their own. Healthlines 42, 14±16.	Wainwright et al. (2000) [39]
4	Department of Health (n.d.) Promoting effective health services for school aged children and young people: A good practice guide. The Stationery Office, London (Autor angeschrieben)	Turner & Mackay (2015) [50]
5	Health Visitors Association. (1991) Pro^®^ling school health. Health Visitors Association, London.	Wainwright et al. (2000) [39]
6	Health Visitors Association. (1992) Health assessments and the school nurse. Health Visitors Association, London.	Wainwright et al. (2000) [39]
7	Joyner, S. (2012). ‘What are school health nurses lived experiences of working with children and their families who are subject to a child protection plan?	Harding et al. (2019) [27]
8	Naish J. & Barr M. (1991) Rights of access. Health Visitor 64, 300±301.	Wainwright et al. (2000) [39]
9	NHS Wales (1988). Putting Patients First. HMSO, London.	Wainwright et al. (2000) [39]
10	Welsh Office (1997) Supporting Pupils with Medical Needs in Schools. Welsh Office Circular 34/97, Welsh Health circular 97/31, Welsh Office (Education Dept), Cardiff.	Wainwright et al. (2000) [39]
	5c. *Primary studies* excluded, as full texts not found	*Reviews* [reason for exclusion]
1	Anyanwu, I. (2005). The Face of Diversity. Challenges in School Health. School Nurse News, 1, 27.	Schmitt & Goerres (2012) [48] [not found]
2	Baldwin, C. M. (1998). Changing health outcomes for African American children: Utilizing a self-care health promotion curriculum in urban elementary schools. Journal of Multicultural Nursing & Health, 4(20), 40–45.	Stock et al. (2002) [51] [not found]
3	Bergren M.D. & Mehl R. (1995b) Health software for school nurses. Journal of School Nursing 11, 6±7.	Wainwright et al. (2000) [39] [not digitalized, no copy available]
4	Bergren M.D. & Murphy E.A. (1997) The best of the web for school health. Journal of School Nursing 13, 36±37.	Wainwright et al. (2000) [39] [not digitalized, no copy available]
5	Bergren M.D. (1996) School nurse politics on the web. Journal of School Nursing 12, 39±40.	Wainwright et al. (2000) [39] [not digitalized, no copy available]
6	Diao, W., Patel, J., Snitzer, M., Pond, M., Rabinowitz, M. P., Ceron, G.,… & Levin, A. V. (2016). The effectiveness of a mobile clinic in improving follow-up eye care for at-risk children. Journal of Pediatric Ophthalmology & Strabismus, 53(6), 344–348.	Best et al. (2018) [3] [not found]
7	Fox, P. G., Cowell, J. M., Montgomery, A. C., & Willgerodt, M. A. (1997). Southeast Asian refugee women and depression: A nursing intervention. The International Journal of Psychiatric Nursing Re- search, 4, 423–432.	Stock et al. (2002) [51] [not found]
8	Fox, P. G., Rossetti, J., Burns, K. R., & Popovich, J. (2005). Southeast Asian refugee children: a school-based mental health intervention. The international journal of psychiatric nursing research, 11(1), 1227–1236.	Tanner (2020) [52] [not found]
9	Kaufman, J., & Blanchon, D. (1996). Managed care for children with special needs: A care coordination model. Journal of Care Management, 2, 46–59.	McClanahan & Weismuller (2014) [53] [not found]
10	Palmore S. & Millar K. (1996) Some common characteristics of pregnant teens who choose childbirth. Journal of School Nursing 12, 19±22.	Wainwright et al. (2000) [39] [not found]
11	Rote S. (1997b) Healthy futures. Nursing Standard 11, 17.	Wainwright et al. (2000) [39] [not found]
12	Smith, S. (2008). The School Nurse as Prevention Specialist. School Nurse News, 11, 28–32.	Schmitt & Goerres (2012) [48] [not found]

**Table 6:** *Primary studies* ordered via the central medical library.

	Articles ordered via Central Medical Library [Ärztliche Zentral Bibliothek]	*Reviews* that included reference
1	Adams C. (1990) Perceptions of the comprehensive-based school nurse. Health Visitor 63, 90±92	Wainwright et al. (2000) [39]
2	Allensworth D.D. (1996) Guidelines for Adolescent Preventive Services: a Role for The School Nurse. Journal of School Health 66, 281±285.	Wainwright et al. (2000) [39]
3	Anyanwu, I. (2005). The Face of Diversity. Challenges in School Health. School Nurse News, 1, 27.	Schmitt & Goerres (2012) [48]
4	Bagnall P. (1995) School nurses’ response to the measles vaccination campaign. Nursing Times 91, 38±39.	Wainwright et al. (2000) [39]
5	Bagnall P. (1997) Children’s health: taking it seriously. British Journal of Community Health Nursing 2, 68.	Wainwright et al. (2000) [39]
6	Baldwin, C. M. (1998). Changing health out- comes for African American children: Utilizing a self-care health promotion curriculum in urban elementary schools. Journal of Multicultural Nursing & Health, 4(20), 40–45.	Stock et al. (2002) [51]
7	Barlow J., Brown S.S. & Fletcher J. (1997) Systematic Review of the School Entry Medical Examination. Health Services Research Unit, Oxford.	Wainwright et al. (2000) [39]
8	Barrett, J. C. (2000). A school-based care management services for children with special needs. Family and Community Health, 23, 36–42.	McClanahan & Weismuller (2014) [53]
9	Bhardwa, S. (2013). "Mental health in young people." Independent Nurse 6.	Turner & Mackay (2015) [50]
10	Bolton P. (1994) School entry screening by the school nurse. Health Visitor 67, 135±136.	Wainwright et al. (2000) [39]
11	Bonaiuto M.M. (1995) Students who depend on medical technology. Journal of School Nursing 11, 21±28.	Wainwright et al. (2000) [39]
12	Bradley, B. J. (1998). Establishing a research agenda for school nursing. *Journal of School Health*, *68*(2), 53–61.	Wainwright et al. (2000) [39]
13	Bradley,B.J.(1997). TheSchoolNurse as Health Educator. Journal of School Health, 1, 3–8.	Schmitt & Goerres (2012) [48]
14	Brother N. (1998) School nursing and student assistance. A natural partnership. Journal of School Nursing 14, 32±35.	Wainwright et al. (2000) [39]
15	Costante C. (1996) Supporting student success: School nurses make a difference. Journal of School Nursing 12, 4±26.	Wainwright et al. (2000) [39]
16	Diao, W., Patel, J., Snitzer, M., Pond, M., Rabinowitz, M. P., Ceron, G.,… & Levin, A. V. (2016). The effectiveness of a mobile clinic in improving follow-up eye care for at-risk children. Journal of Pediatric Ophthalmology & Strabismus, 53(6), 344–348.	Best et al. (2018) [3]
17	Fagan R. (1995) Health of the Nation Targets: where school nurses ^®^nd constraints on achievement. Nursing Standard 9, 36±40.	Wainwright et al. (2000) [39]
18	Few C., Hicken I. & Butterworth T. (1996) Alliances in school sex education: teachers and school nurses’ views. Health Visitor 69, 220±223.	Wainwright et al. (2000) [39]
19	Fox, P. G., Cowell, J. M., Montgomery, A. C., & Willgerodt, M. A. (1997). Southeast Asian refugee women and depression: A nursing intervention. The International Journal of Psychiatric Nursing Re- search, 4, 423–432.	Stock et al. (2002) [51]
20	Fox, P. G., Rossetti, J., Burns, K. R., & Popovich, J. (2005). Southeast Asian refugee children: a school-based mental health intervention. The international journal of psychiatric nursing research, 11(1), 1227–1236.	Tanner et al. (2020)[52]
21	France J (2013) New texting service for teenagers has all-round benefits. Nursing Standard 28(5): 13	Turner & Mackay (2016) [50]
22	Fryer, G. E., & Igoe, J. B. (1995). A relationship between avail- ability of school nurses and child well-being. Journal of School Nursing, 11(3), 12–18.	Wainwright et al. (2000) [39]
23	Gaffrey, E. A.; Dewey Bergren, M. (1998). School Health Services and Mana- ged Care. Journal of School Nurs- ing, 4, 5–20.	Schmitt & Goerres (2012) [48]
24	Henry. (1997) A nursing informatics approach for addressing national issues and priorities for school nursing services. Journal of School Nursing 13, 39±41.	Wainwright et al. (2000) [39]
25	Igoe J.B. (1994) School Nursing. Nursing Clinics of North America 29, 443±458.	Wainwright et al. (2000) [39]
26	Kaufman, J., & Blanchon, D. (1996). Managed care for children with special needs: A care coordination model. Journal of Care Management, 2, 46–59.	McClanahan & Weismuller (2014) [53]
27	Kimel L.S. (1996) Handwashing education can decrease illness absenteeism. Journal of School Nursing 12, 14±18.	Wainwright et al. (2000) [39]
28	Kornguth M.L. (1991) Preventing school absences due to illness. Journal of School Health 61, 272±274	Wainwright et al. (2000) [39]
29	Lamb J.M., Albrecht S. & Sereika S. (1998) Consideration of factors prior to implementing a smoking cessation program. Journal of School Nursing 14, 14±19	Wainwright et al. (2000) [39]
30	Land, M., & Barclay, L. (2008). Nurses’ contribution to child protection. Neonatal, paediatric and child health nursing, 11(1), 18–24.	Harding et al. (2019) [27]
31	Lunney M. (1996) The signi^®^cance of nursing classi^®^cation systems to school nursing. Journal of School Nursing 12, 35±37.	Wainwright et al. (2000) [39]
32	Lunney M., Cavendish R., Kraynyak Luise B. & Richardson K. (1997) Relevance of NANDA and health promotion diagnoses to School Nursing. Journal of School Nursing 13, 16±22.	Wainwright et al. (2000) [39]
33	Nutbeam D., Farley P. & Smith C. (1990) England and Wales. Perspectives in school health. Journal of School Health 60, 318±322.	Wainwright et al. (2000) [39]
34	Oda D.S. (1992) Is school nursing really the invisible practice? Journal of School Health 62, 112±113.	Wainwright et al. (2000) [39]
35	Palmore S. & Millar K. (1996) Some common characteristics of pregnant teens who choose childbirth. Journal of School Nursing 12, 19±22.	Wainwright et al. (2000) [39]
36	Reid J.A. (1991) Developing the role of the school nurse in public health. Health Education Journal 50, 118±122.	Wainwright et al. (2000) [39]
37	Resnicow K. & Allensworth D. (1996) Conducting a comprehensive school health program. Journal of School Health 66, 59±63.	Wainwright et al. (2000) [39]
38	Rote S. (1997b) Losing sight of the future. Nursing Times 93, 24, 58±59.	Wainwright et al. (2000) [39]
39	Schoenfeld D.J. (1996) Talking with school-age children about AIDS and death. Journal of School Nursing 12, 26±32.	Wainwright et al. (2000) [39]
40	Skelley JP, Luthin DR, Skelley JW, Kabagambe EK, Ashraf AP, Atchison J.A. Parental perspectives of diabetes management in Alabama public schools. South Med J. 2013;106:274–9. doi: 10.1097/smj.0b013e31828de4a4.	Stefanowicz & Stefanowicz (2018) [49]
41	Smith, S. (2008). The School Nurse as Prevention Specialist. School Nurse News, 11, 28–32.	Schmitt & Goerres (2012) [48]
42	Staudt, A.M., Alamgir, H., Long, D. L., Inscore, S. C.,&Wood, P. R. (2015). Developing and implementing a citywide asthma action plan: A community collaborative partnership. Southern Medical Journal, 108, 710–714. doi:10.14423/SMJ.0000000000000380	Best et al. (2018) [3]
43	Urbinati, D.; Steele, P.; Harter, B. J. E.; Harrell, D. (1996). The Evolution of the School Nurse Practitioner: Past, Present and Future. Journal of School Nursing, 2, 6–9.	Schmitt & Goerres (2012) [48]
44	Whitmore K. (1988) School refusal. Health Visitor 61, 349±351.	Wainwright et al. (2000) [39]

### 3.2 Study characteristics of *reviews*

All of the *reviews* included in our *overview of reviews* focus on school nurse-led interventions and their impact on children’s health outcomes. Each of the research papers focuses on different aspects of the content. Studies that summarized the scope of school nurses’ interventions and general health outcomes were examined by two research groups [3,51]. Two papers summarized evidence on vaccination rates for students [54,55]. Child maltreatment and prevention [27], asthma care [56], school performance [57,58], obesity prevention [31], diabetes [49], epileptic seizures [29] and mental health [50,59] were also scrutinized. While one study focused on children with complex needs [53], another paper concentrated on general health promotion [39]. One German study summarized evidence on the school nurse, with implications for the German school system [48]. The time periods covered by *reviews* were 1976 up to 2021. About 50% of *reviews* were conducted in the USA [3,31,51–53,55,56,58], while the other references were from the UK [27,39,50,57,59], Italy [54], Poland [49] and Germany [48] (Table 7).

**Table 7:** Characteristics of *reviews* (k = 16).

First author [country] (year)	Time-period covered	Study design (Type of Overview)	Number of included references	Subject matter							Summary findings	Funding
				Mental health	Somatic Illness	Health literacy	Prevention & Health Promotion	Interdisciplinary Aspects	Case management	Attendance and / academic achievement		
Best, Oppewal [3] [USA] (2018)	2011–2017	(Integrative Literature Review)	65		X	X			X		“School nurse interventions […] benefited school-aged children with life-threatening diseases like asthma and diabetes and children with serious health conditions […]”.	No funding
Guarinoni and Dignani [54] [Italy] (2021)	1976–2016	(Narrative Review)	9				X				“The present study shows that the school nurse plays a key role in increasing the rate of adhesion to immunization for school-aged children/ adolescents”.	Not mentioned
Harding, Davison-Fischer [27] [UK] (2019)	Until 2019	(Integrative Literature Review)	21				X				“Huge variety of activities that school nurses undertake to protect children from maltreatment. Several challenges to this role are identified, including time management and building relationships with children”.	Not mentioned
Isik and Isik [56] [USA] (2019)	2011–2018	(Integrative Literature Review)	12		X				X		“School nurses can drive effective asthma care […] and prevent health care fragmentation, emergency room visits, hospitalization, school absenteeism, and can increase asthma knowledge and the quality of life for students and parents”.	Not mentioned
Lineberry and Ickes [55] [USA] (2014)	1937–2013	(Systematic Review)	30	X	X	X	X				“While some studies of immunization compliance, attendance rates, body mass index screening, vision screening, and follow-up are promising, results are mixed and additional evidence is needed”.	No funding
Maughan [58] [USA] (2003)	1965–2003	(Research Synthesis)	15				X			X	“Nursing interventions targeted at specific populations, including parents, have had significant effects.”	Not mentioned
McClanahan and Weismuller [53] [USA] (2014)	1990–2013	(Integrative Literature Review)	25						X		“Recommendations for improving care coordination were elucidated in the review. Analysis of the literature can help assure application of best practice methods for the coordination of care for students in the school setting”.	No funding
Schmitt and Görres [48] [Germany] (2012)	1983–2009	(Integrative Literature Review)	34						X	X	“The School Nurse has a wide range of activities, with coordination and mediation as a central area of responsibility”.	Not mentioned
Schroeder, Travers [60] [USA] (2016)	unclear	(Systematic Review and Meta-Analysis)	8		X						“Findings […] suggest that school nurses can play a key role in implementing sustainable, effective school-based obesity interventions”.	National Institute of Nursing Research
Stefanowicz and Stefanowicz [49] [Poland] (2018)	unclear	(Literature Review)	12		X						“According to parents and children with type 1 diabetes mellitus, various forms of school nurse support […] are consistently effective and should have an impact on the condition, improvement of metabolic control, school activity and safety at school”.	No funding
Stock, Larter [51] [USA] (2002)	1980–2001	(Literature Review)	26	X	X	X	X				“15 articles documented positive outcomes related to school nurse services. A majority of the outcomes pertained to health education and prevention”.	Wash-ington State Office of Superinten-dent of Public Instruction
Tanner et al. [52] [USA] (2020)	1998–2017	(Integrative Review)	15	X							“School nurses play an active role in mental health interventions and should be involved in replicating and testing known mental health interventions to investigate their effectiveness for students with Psychogenic nonepileptic seizures (PNES)”.	Robert Wood Johnson Foundation: Future of Nursing Scholars Program
Tilley and Chambers [59] [UK] (2003)	1990–2003	(Systematic Review)	0	X							“The systematic review did not locate any current published evidence of existing screening tools being applied by school nurses to detect mental ill health among adolescents in schools. The effectiveness or ineffectiveness of such tools used by school nurses thus could not be evaluated”.	Welsh Office of Research and Development for Health and Social Care
Turner and Mackay [50] [UK] (2015)	unclear	(Literature Review)	29	X							“A variety of evaluation methods were used to identify outcomes, including qualitative and quantitative methods and standardised tools. However, the majority of papers described outcomes without reference to a systematic approach to evaluation”.	Not mentioned
Wainwright, Thomas [39] [UK] (2000)	1980–2000	(Brief of Literature)	48			X	X	X	X		“The results of the review were disappointing, in that little research of acceptable quality was found and little could be said about the effectiveness [of school nurses]. The result is therefore a more diffuse review that gives a summary of descriptive research and current views and opinions […]”.	Not mentioned
Yoder [57] [UK] (2020)	2002–2018	(Integrative Literature Review)	16							X	“[…] The presence of a school nurse is associated with reduced absenteeism and missed class time but not with academic achievement”.	No funding

### 3.3 Quality assessment of *reviews*

Out of a total of sixteen references, six studies are of moderate quality (score: 16–32) [31,32,49,52,56,57], ten are of low quality [3,27,39,48,50,51,54,55,58,59] and no study is of high quality (Table 8).

**Table 8:** Methodological quality assessment of *reviews* based on AMSTAR-2 criteria (k = 16).

	Best et al. (2018) [3]	Guarinoni & Dignani (2021) [54]	Harding et al. (2019) [27]	Isik & Isik (2019) [56]	Lineberry & Ickes (2014) [55]	Maughan (2003) [58]	McClanahan & Weismuller (2014) [53]	Schmitt & Görres (2012) [48]	Schroeder et al. (2016) [31]	Stefanowicz & Stefanowicz (2018) [49]	Stock et al. (2002) [51]	Tanner et al. (2020)[52]	Tilley & Chambers (2003) [59]	Turner & Mackay (2015) [50]	Wainwright et al. (2000) [39]	Yoder (2020) [57]
**A. Research question & selection process**																
1. Report of inclusion criteria (PICO)	3	0	2	1	2	3	2	1	3	4	3	3	2	0	3	4
2. Explanation for selected study designs	0	0	0	0	0	0	0	0	0	0	0	0	0	0	0	0
3. Comprehensive search strategy	3	3	3	4	2	2	4	2	2	3	3	3	2	4	4	2
4. Two independent researchers	4	4	3	3	0	0	4	0	0	0	0	0	0	0	3	0
5. List of excluded studies	0	0	0	0	0	0	0	0	0	3	0	0	0	0	0	0
**B. Assessment of included primary studies**																
1. Description of studies in adequate detail	0	3	3	2	3	3	0	1	4	3	2	3	0	2	1	3
2. Assessment of Risk of Bias (RoB)	0	0	0	0	0	0	0	0	3	0	0	0	0	0	0	0
3. Report of funding	0	0	0	0	0	0	0	0	0	0	0	0	0	0	0	0
4. Performance of meta-analysis with appropriate methods	0	0	0	0	0	0	0	0	4	0	0	0	0	0	0	0
**C. Interpretation of results**																
1. Consideration of RoB in results	0	0	0	0	0	0	0	0	4	0	0	0	0	0	0	0
2. Explanation of heterogeneity in results	0	4	4	4	0	4	4	4	4	4	4	4	4	4	0	4
**D. Other**																
1. Report of potential source of conflict	4	0	0	4	4	0	4	0	4	4	0	4	0	4	0	4
**Overall assessment**																
**1. Overall score (max = 48)**	**14**	**14**	**15**	**18**	**11**	**12**	**18**	**8**	**28**	**22**	**12**	**17**	**8**	**14**	**11**	**17**
**2. Assessment (high, moderate or low quality)**	L	L	L	M	L	L	M	L	M	M	L	M	L	L	L	M

*Notes*. Categorisation of quality rating (see table X): 0 = 0% of sub-criteria met, 1 = 1%–24% of sub-criteria met, 2 = 25%–49% of sub-criteria met, 3 = 50%–74% of sub-criteria met, 4 = 75%-100% of sub-criteria met; H = high study quality (overall score = 32 or more), M = moderate study quality (overall score = 16 to 32), L = low study quality (overall score = 16 or less).

### 3.4 Study characteristics of *primary studies*

Of the 289 *primary studies* included, there were 32 references (11%) that were identified as RCTs, 64 (22%) as Obs, a further 98 (34%) used a qualitative-descriptive design, 79 (27%) used a quantitative-descriptive design and 16 references (6%) could not be assigned to any study design. Studies were conducted in the USA (j = 210), UK (j = 59), Sweden (j = 8), Australia (j = 3), Finland (j = 3), Netherlands (j = 2), France (j = 2), Spain (j = 1) and Nigeria (j = 1).

**Fig 6:**
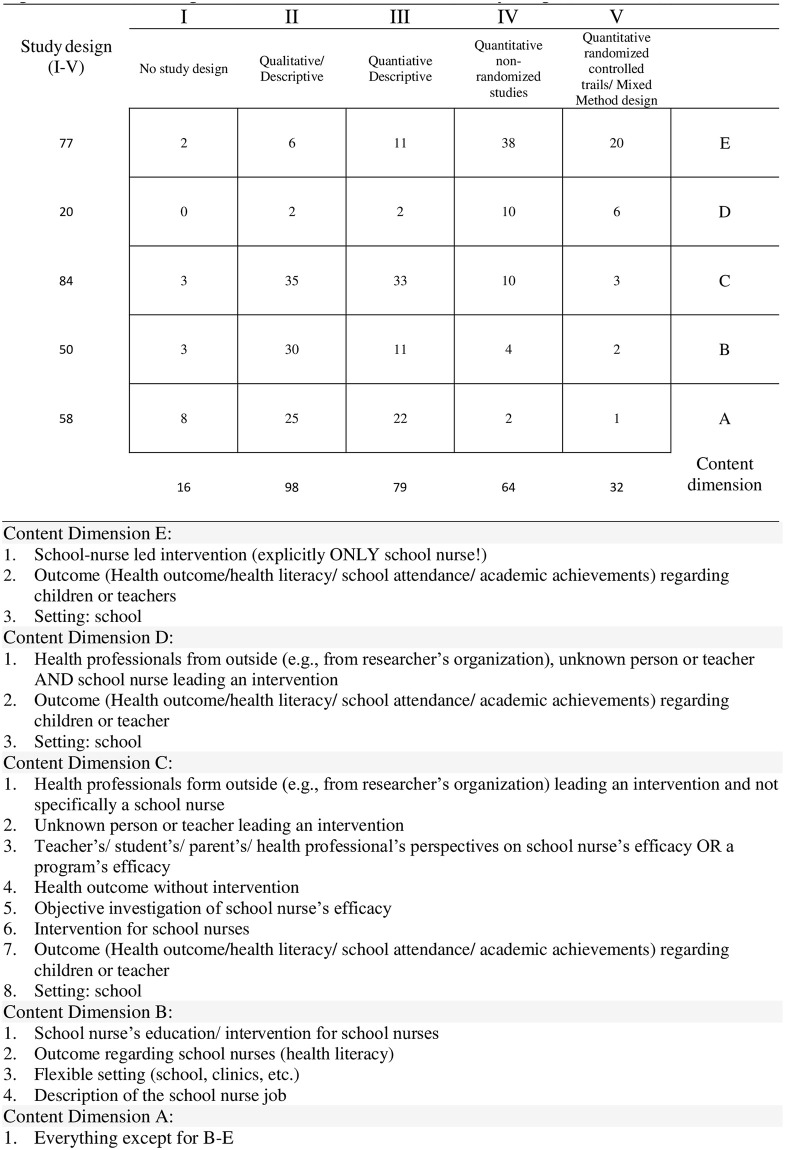
Matrix for categorization of content dimension and study design (with results).

A total of 77 references (27%) examined school nurse-led interventions, where a school nurse alone was involved, whereas 20 references (7%) examined interventions where a school nurse as well as other health professionals or teachers performed an intervention. Most studies (84; 29%) examined interventions conducted by other health professionals (not school nurses) or they described health outcomes without conducting an intervention. These studies focused on a program’s efficacy and school nurse’s efficacy from the perspective of teachers, parents or health professionals. A significant number of studies (50; 17%) focused on school nurses`training and described the school nursing job. The remaining studies (58; 20%) examined different aspects not relevant to the present study (S2 Table, Fig 6).

### 3.5 Selected *primary studies* to examine body of evidence

Relevant *primary studies* were selected according to two criteria: study design and content dimension (Fig 6). Studies with the study design RCT and Obs and content dimensions “D” and “E” were selected (total: 74 studies). There were eight references examining the outcome “school attendance” of which one study identified as an RCT [61]. From eleven studies investigating obesity prevention, six were RCTs [17,38,62–65]. Physical activity was investigated by three research groups of which one identified as RCT [66]. Researchers of eight studies investigated emotional health, of which two were RCTs [14,67]. Of 16 papers examining asthma care, six identified as RCTs [68–73]. One RCT examined smoking cessation [19] and one RCT investigated immunization rates as a health outcome [35]. Sexually transmitted virus prevention was covered by two RCTs [74,75] and evidence on teenage pregnancy, nutrition, and teasing and bullying, respectively, was investigated in one Obs [76–78]. Allergies [79], alcohol use prevention [80], hearing screening [81], cardiovascular health [82], psychosomatic headaches [83] and hygiene [34] were each assessed by one RCT. Two research groups investigating diabetes used RCTs [84,85]. Five research groups did not confine themselves to one specific outcome but addressed a range of different health outcomes in one *primary study*, of which one identified as RCT [86]. Assessing the RoB, 16 *primary studies* showed a low risk of bias, 33 showed a high risk and 25 showed an unclear risk of bias (Table 9).

**Table 9:** Body of Evidence according to GRADE assessment.

Author	Design RCT/Obs	Indirectiveness of Evidence			Publication bias	RoB [Scale] Category	Imprecision		Impact
		Population	Intervention	Measures			N	CI*	
School Attendance									
Allen [13]	Obs	Children in elementary schools	Full-time school nurse	All day attendance, check out for medical reasons, health care insurance	No indication	High [8] E, F	10,000	/	The percentage of student checkouts for medical reasons in schools with a full-time nurse (M = 11.1%) was statistically significantly lower, t(20) = 2.27, a< .05, d = 2.77 than schools without a fulltime nurse (M = 15.7%).
Long et al. [61]	RCT	Children with more than 14 days absence in one year	Activity of the school nurse and record of her activity	Mean days of absence	No indication	Low [2] E	302	/	The control group’s mean decline was 5.10, resulting in a statistically significant mean difference of 1.98 between the intervention and control groups. In other words, pupils in the intervention group showed a decline in absences which averaged 2 days more than the reduction experienced by those in the control group, and this difference can be viewed as a non-chance occurrence.
Foster & Keele [87]	Obs	Children in kindergarten up to grade 5	School nurses administer Over-the-Counter medications	Sent home rates	No indication	High [8] E, D	Approx. 10,000	/	There were no statistically significant differences found among sent home rates for the 2 school years after the policy change. The mean number of students sent home before the policy was implemented was 353, or 3.6%, whereas the mean number of students sent home in the first year after the policy was implemented was 342 students, or 3.4%, 2(23) = -.37, P = .72. The second year of implementation found 329 students, or 3.1%, were sent home.
Rodriguez, Rivera [88]	Obs	Children in pre-kindergarten to grade 8	Trained full-time school nurse and part-time school nurses	Average Daily Attendance was measured through parent report + verification of school clerk, health measures	Unlikely, funded by Lucile Packard Foundation for Children’s Health and the Lucile Packard Children’s Hospital	High [8] H, F	6,664	√	Decrease in the mean number of absences due to illness among students in demonstration schools, whereas the mean number of absences among students in control schools increased. This reduction in mean absences due to illness was statistically significant between demonstration and comparison groups (p < .05). students in demonstration schools were less likely than students in control schools to miss 1 or more days of school due to illness (OR = 0.876, 95% CI: 0.767–1.001, p < .05).
Telljohann, Dake [89]	Obs	Children with asthma in kindergarten to grade 6	Full-time school nurse (5 days) vs. part-time school nurse (2 days)	Grade, race, sex, school lunch status, and number of days missed	No indication	High [9] D, I	569	/	Students with full-time school nurses missed significantly fewer school days (M = 510.6, SD = 59.2) than students with part-time school nurses (M = 513.0, SD = 511.6) (t = 522.68, DF = 5566, p < .05).
Van Cura [90]	Obs	Students with lower socioeconomic status	School-based health centers	Academic outcomes and loss of seat time (data on early dismissal)	No indication	Unclear [7] G, F	764	/	SBHCs significantly reduced the number of early dismissals from school (p = .013) in a comparison with students who received school nursing services alone. Students not enrolled in an SBHC lost 3 times as much seat time as students enrolled in an SBHC.
Weismuller, Grasska [116]	Obs	Children in kindergarten to grade 5	Retrospective review of health records	Referrals to school nurse, interventions, outcome of school nurse intervention; Schools Administrative Student Information system (SASIS) to document reason for absence	No indication	High [10] G, J(1), I	240	/	The most common reason (65.8%) for referral was screening. After that, physical illness. No referrals to the school nurse for absenteeism and school nurse interventions were not targeted to attendance, despite 17% of students missing 11 or more school days. Documentation was sparse (primarily task related). Information about the outcome was insufficient to determine the effectiveness of nursing interventions.
Wiggs-Stayner, Purdy [91]	Obs	School-aged children and staff	Free Flu-Mist immunizations	Attendance rates	Unlikely, funded by the Parkview Hospital Community Health Improvement Program	Unclear [7] H(2)	277 + unknown control	/	The 2 schools receiving FluMist increased their attendance rates from 95.3% and 93.9% to 96.1% and 95.8%. Previously, the comparison schools each had a 94.6% attendance rate; one fell to 94.4% and the other rose very slightly to 94.7%. The differences in self- or parent reported influenza absences were not significant. However, the difference in days absent between individual vaccinated and nonvaccinated schools was statistically significant.
Overweight and Obesity Prevention									
Bonsergent, Agrinier [17,62]	2x RCT	Children in high schools	Education, environment and screening by school nurse	Weight and height (waist circumference later excluded)	No indication	Low [2] D(3), F	3,538	√	The 2-year change of outcomes was more favorable in the 12 month screening compared to the no-screening ones: a 0.11 lower increase in BMI (p<0.0303); a 0.04 greater decrease in BMI z-score (p<0.0173); and a 1.71% greater decrease in overweight/obesity prevalence (p_0.0386). Education and environment strategies were not more effective than no strategy intervention.
Hawthorne et al. [92]	Obs	Children in schools	Walking program 3 days a week/ 16 weeks	Body Mass Index (BMI), waist circumference, and cardio-respiratory (by using the Progressive Aerobic Cardiovascular Endurance Run test (PACER test)	Unlikely, funded by Kids Sports Stars for data analysis funding	High [8] F, G	1,074	/	Cardio-respiratory fitness increased by 37.1% over baseline in the entire sample (p < .01). The increase in fitness was observed in both genders and across all grade levels. Furthermore, significant improvements in fitness were observed among healthy weight, overweight, and obese youth. However, obese youth had a smaller overall change in fitness compared to overweight or healthy weight participants. No significance changes in BMI or waist circumference (p > .05);
Johnston et al. [38,63]	RCT	Children aged 7–9 years	Curriculum with health information + health professional	Weight and height to measure Body Mass Index (BMI); Grades in math, science and reading to measure academic outcome	No indication	Unclear [5] B, D, F	835	√	After 2 years, children who were overweight/obese in the professional-facilitated intervention (PFI) condition significantly reduced their standardized BMI (zBMI) compared to children in the Self-help (SH) condition (Wald χ2 = 28.7, p < .001). End-of-year grades decreased for overweight/obese students in both conditions; however, students in the PFI exhibited a smaller decrease in grades compared to the SH condition (Wald χ2 = 80.3, p < .001).
Melin and Lenner [93]	Obs	Overweight children 7 years of age	Dietary advice	Weight, BMI, Changes in well-being and lifestyle were measured with the help of a structured interview	No indication	High [10] G, J(4), I	20	/	A good (91%) or fair (54%) adherence to dietary advice was found in children who decreased or maintained their z-score respectively. Mean BMI z-score reduced [)0.16 (p = 0.03)] during the intervention period. Generally, parents and school nurses were satisfied with the program, helping them to set limits and be more self-confident in their role as ‘health adviser’ respectively.
Note. Detailed risk of bias assessment (1) Use of existing health records; documentation varied widely among school sites and study information was limited to existing record contents; (2) No information on control group (size, population, etc.); (3) Lack of an “ideal” anthropometric outcome to evaluate the effectiveness of prevention strategies. BMI might be considered an inappropriate way to evaluate weight status in children and adolescents, as it naturally increases with age; (4) Fear for stigmatization, considering the intervention design with visits to the nurse during school hours									
Pbert, Druker [65]	RCT	Overweight or obese adolescents in grades 9 to 12	School nurse-delivered cognitive behavioral counseling and an afterschool exercise program	BMI, 24-hour dietary recall interview, Physical activity (PA) with accelerometer, Survey to measure sedentary behaviour, TV, computer games, questionnaires to measure self-efficacy (11-item questionnaire) and perceived barriers	Unlikely, funded by National Institutes of Health, National Heart, Lung and Blood Institute	Low [2] G	126	√	Students in the intervention compared with control schools showed no significant differences in anthropometric variables including BMI, percent body fat, and waist circumference at follow-up. Students in intervention compared with control schools reported eating breakfast on significantly more days/weeks at follow-up, adjusted mean 4.65 vs. 3.84 days, respectively (adjusted mean difference 0.81 days; 95% CI 0.11–1.52). The mean number of days students reported being physically active in the past 7 days was similarly high in the intervention compared with control schools at follow-up, adjusted mean 4.54 days vs. 3.64 days, respectively (adjusted mean difference 0.89 days; 95% CI 0.25–1.53).
Pbert, Druker [64]	RCT	Overweight or obese adolescents in grades 9 to 12	School nurse-delivered counseling intervention “Lookin’ Good Feelin’ Good”, utilizing cognitive-behavioral techniques	BMI, 24-hour dietary recall interview, PA with accelerometer, Survey to measure sedentary behaviour, TV, computer games, questionnaires to measure self-efficacy and perceived barriers	Unlikely, funded by National Institutes of Health, National Heart, Lung and Blood Institute	Unclear [4] E, G	84	√	At 2 months, intervention participants ate breakfast on more days/week (difference = 1.01 days; 95% CI 0.11, 1.92), and had a lower intake of total sugar (difference = −45.79g; 95% CI −88.34, −3.24) and added sugar (difference = −51.35g; 95% CI −92.45, −10.26) compared to control participants. At 6 months, they were more likely to drink soda ≤ one time/day (OR 4.10: 95% CI 1.19, 16.93) and eat at fast food restaurants ≤ one time/week (OR 4.62: 95% CI 1.10, 23.76) compared to control participants. There were no significant differences in BMI, activity or caloric intake.
Sherman, Alexander [94]	Obs	Obese children in grades 4–6	9 lessons of a 9-week period which addressed self-esteem, food choices and nutrition, and PA and fitness	Weight skinfold measurements (desired outcome: amount of lean and fat tissue in the body), questionnaires to measure self-esteem and nutritional knowledge	No indication	High [10] G, I, F	26	/	Self-esteem increased significantly (p< .001) between the pre-test and post-test interval. Weight status and nutritional knowledge showed no improvement.
Speroni, Earley [95]	Obs	Children grade 2–5	Kids Living Fit-Intervention ($100 fee to Participate) PA activity, dietary education	BMI, waist circumference, questionnaires to measure food, activity, and satisfaction	No indication	Unclear [7] H	185	/	All KLF intervention groups in the four schools experienced a decrease in BMI percentile from the baseline measure to remeasure recorded at Weeks 12 and 24. The two single largest decreases in BMI percentile occurred in the contrast groups in Schools 1 and 4, with an 8.5 and 7.9 (p< .1) percentile point decrease, respectively. By comparison, participants in the KLF group in School 1 had a 4.0 (p< .1) percentile point decrease in BMI.
Tucker and Lanningham-Foster [96]	Obs	Children in grade 4 and 5	Refined health messaging (Let’s Go 5-2-1-0) program	BMI percentile, Healthy Habits Survey to measure nutrition, screen time, PA, and family eating patterns, a StepWatch Activity Monitor to measure PA levels	No indication	High [10] G, D, I	72	/	School A: BMI percentile did not change significantly over time. The mean/median number of servings of fruits and vegetables per day significantly increased from baseline to end of year (p = .001), while the number of servings of 100% fruit juice per day significantly decreased (p = .003). There was also a trend in minutes of self-reported active play, which increased from baseline to end of year (p = .057). Objectively measured participant PA levels increased significantly (p < .005) from baseline (M = 12,139, SD = 401 steps) to end of year (M = 15,120, SD = 680).
Wong and Cheng [97]	Obs	Obese children in primary schools	Motivational interviewing (MI) counselling; + telephone consultation for parents (MI+)	Change in weight for- height percentage with reference to a territory-wide growth survey, changes in weight-related behaviours and anthropometric measures	Unlikely, funded by the Hong Kong Institute of Education	Unclear [6] I	185	/	There was a significant decrease in the average calorie intake from food in the past seven days in the MI group (mean difference: 389 57; p < 0 01) and in the MI+ group (mean difference: 376 65; p < 0 01). It also showed a significant increase in the average calories consumed due to an increase in physical exercise in the past seven days in the MI group (mean difference: 2052 10; p < 0 01) and in the MI+ group (mean difference: 2590 64; p < 0 01). Control group had significant deterioration in their anthropometric measures.
Physical activity									
Robbins, Pfeiffer [98]	Obs	Middle school girls	Girls on the Move (3 motivational, individually tailored counseling sessions + after-school physical activity	Questionnaires on benefits/barriers of PA, self-efficacy, etc. to measure cognitive and affective variables related to PA, PA, cardiovascular, and body composition (BMI, waist circumference)	Unlikely, funded by National Heart, Lung, and Blood Institute and the National Institutes of Health	High [8] J(5), G	69	/	Linear regressions controlling for baseline measures showed no statistically significant group differences, but directionality of differences was consistent with greater intervention group improvement for minutes of moderate to vigorous physical activity/ hour (t = 0.95, p = .35), cardiovascular fitness (t = 1.26, p = .22), body mass index (BMI; t = −1.47, p = .15), BMI z-score (t = −1.19, p = .24), BMI percentile (t = −0.59, p = .56), percent body fat (t = −0.86, p = .39), and waist circumference (t = −0.19, p = .85).
Williams and Warrington [99]	Obs	Children in grade 3–5 (8–10 years of age) in elementary school	Pedometer-based walking program, Get Fit Kids	Change in step counts from the children’s pedometers, participant satisfaction, and program costs	No indication	Unclear [7] D, J(6)	231	√	There was a statistically significant difference in the step counts from Time 1 (M = 6,567.73, SD = 5,189.51) to Time 2 (M = 10,804.60, SD = 11,664.81), t = 3.374, p = 0.001 (two-tailed). The mean difference in the step counts was 4,236.87, with confidence levels ranging between 1,715.633 and 6,758.098.
Wright, Giger [66]	RCT	Children aged 8–12 years	Kids N Fitness (45 min PA), nutrition education, school-wide wellness activities	Child and Adolescent Trial for Cardiovascular Health School Physical Activity and Nutrition Student Questionnaire, anthropometric measures (Height, weight, body mass index, resting blood pressure, and waist circumference)	Unlikely, funded by the NIH/ NCMHD Loan Repayment Program and Robert Wood Johnson Foundation	Low [2] G	251	√	Significant results for students in the intervention, included for boys decreases in TV viewing; and girls increases in daily physical activity, physical education class attendance, and decreases in body mass index z-scores from baseline to the 12-month follow-up.
Note. Detailed risk of bias assessment: (5) non-compliance with accelerometer wear; (6) Not a validated questionnaire;									
Emotional Health									
Attwood et al. [14]	1 x RCT 1 x Obs	Children with mean age of 10.6 years	Computerized cognitive therapy	Spence Children’s Anxiety Scale (SCAS), Strengths and Difficulties Questionnaire (SDQ) to measure desired outcome of anxiety and parental perception of child behavior	No indication	Unclear [7] B, J(7),F, G	12	/	A statistically significant post-intervention reduction in total child anxiety ratings on the SCAS-C for the cCBT (Z =) 1.79, p < .05), but not for the gaming condition (Z =) 1.59, p > .05). Analysis of subscale scores revealed a statistically significant post-intervention reduction on the social anxiety (Z =) 1.80, p < .05) and generalized anxiety (Z =) 1.73, p < .05) sub-scale scores for the cCBT group only.
Clausson and Berg [23]	Obs	Children aged 11–17 and their families	Calgary Family Assessment Model (CFAM), Calgary Family Intervention Model (CFIM), Illness Beliefs Model (IBM)	SDQ to measure desired outcome of emotional symptoms, conduct, hyperactivity, peer problems, and prosocial behavior	No indication	High [10] G(8), F, C, E	4	/	The families reported feeling relief and described positive affective, behavioral, and cognitive changes as a consequence of the interventions. The school nurses experienced the family sessions as time-saving and easy-to-use tools in their work.
DeSocio et al. [100]	Obs	Children aged 10–12	Education program (brain, managing stress, resources, help)	16-item pre-and posttest covering content delivered in the modules to measure the desired outcome of knowledge gain	Unlikely, funded by Northwest Health Foundation	High [8] H, F	370	/	Overall, student scores improved significantly from pre- to posttest (mean score increased by 1.5, P = .000). Greatest improvements in: (a) knowledge that mental illness is not the same as mental retardation; (b) knowledge that genetic factors, internal thoughts, and environmental events contribute to mental health; (c) recognition that when someone talks about suicide, it is a message to be taken seriously-the person is not just trying to get attention; (d) awareness that relationships, as well as events, can generate stress; I knowledge of chemical changes that occur in the body during stress; and (f) awareness that it is not possible to tell if someone has a mental illness just by looking at them.
Houck, Darnell [101]	Obs	Adolescents with depressive symptoms and related problems	1: cognitive–behavioral intervention„ 2: A Peer Group Approach to Building Life Skills	Questionnaires to measure desired outcome of emotional distress, resources and coping skills and survey of risk to measure suicide risk factors	No indication	High [10] E, G, I	12	/	Assessment revealed that the students were at suicide risk. At the conclusion of the group intervention, there was a 55% decrease in suicidal ideation, a 27% decrease in perceived stress, and a 26% decrease in family distress. In addition, most of the students became engaged in formal treatment for the first time.
Lamb et al. [67]	RCT	Children 14–18 years in rural area	Screening of depressive symptoms, suicidal ideation, nurse-led coping skills group	The Jalowiec Coping Scale (JCS) to measure desired outcome of coping skills, The Reynolds Adolescent Depression Scale (RADS) to measure depressive symptoms	No indication	Unclear [3] G, J(9)	40	/	Intervention subjects showed marginally significant greater decrease than control subjects (p < .001), intervention subjects showed a marginally significant greater decrease than control subjects (p = .074). Analysis of the depression screening tool (RADS) score demonstrated that 86.9% of the intervention group showed decreased depressive symptomatology with mean improvement of 15 points, whereas 61.1% of controls showed mean improvement of 13.8 points on RADS.
Note. Detailed risk of bias assessment: (7) Absence of an appropriate comparison group limits the strength of any conclusions as it is unclear whether the reported improvements were due to cCBT or the passage of time; differences in both child-rated total anxiety and parent-rated SDQ scores between the cCBT and gaming groups at baseline; (8) Insufficient reporting of results, descriptive; (9) Unclear if control group didn’t realize they weren’t in the intervention group, as they didn’t receive any treatment, not even a placebo. It seems as if there was a lack of allocation concealment									
Muggeo, Stewart [102]	Obs	Children with anxiety symptoms aged 5–11 years	Child Anxiety Learning Modules (CALM), (cognitive behavioral strategy)	Screen for Child Anxiety-Related Emotional Disorders, Child and Parent Versions (SCARED) to measure desired outcome of anxiety; Woodcock–Johnson Tests—Achievement and Cognitive Batteries (WJ-III) to measure academic achievement and cognitive abilities	Possibly, funded by the Dep. Of Education’s Institute of Education Sciences	High [10] I, G, J(10)	11	√	Results indicated significant reductions in anxiety based on child self-reported SCARED-C; t(9) = 3.36, p = .004, 95% CI [3.24, 16.56], Cohen’s d = 0.81. Significant reductions in somatic symptoms were reported at post-intervention by parents CSI-24; t(10) = 2.49, p = .016, 95% CI [41, 7.58], Cohen’s d = .78 9 and children CSI-24; t(9) = 2.21, p = .027, 95% CI [-.18, 15.98], Cohen’s d = .85. Global functioning scores, as measured by the CGAS, were significantly higher at post-intervention, with a small effect size, t(10) = -3.45, p = .003, 95% CI [-12.11, -2.61], Cohen’s d = .35
Ramirez, Harland [103]	Obs	Trauma-exposed children	Listen, Protect, Connect (LPC)	Modified Child PTSD Symptom Scale, Center for Epidemiologic Studies Depression Scale (CES-D) to measure depression, Multidimensional Scale of Perceived Support (MSPSS) to measure perceived social support and Healthy Kids Resilience Measure of School Connectedness	Unlikely, funded by the University of Iowa Injury Prevention Research Center	High [10] G, E, I	20	√	A significant decline in depressive symptoms was seen from baseline to each follow-up period, all levels below the clinical cut point for depression. PTSD symptoms decreased 3.7 points from baseline to the 8-week follow-up, although this change was not statistically significant (range 15.5–11.8; p = 0.09). Total social support increased from baseline to the 2-week follow-up (p = 0.08) and increased significantly from baseline to the 8-week follow-up (p < 0.01). Students felt more connected to their school at 2- (mean = 63.8, p = 0.06) and 4-weeks (mean = 68.9, p < 0.01) than at baseline (mean = 58.6), but this relationship diminished by 8-weeks.
Stallard et al. [104,105]	Obs	Children aged 9 and 10	Evidence-based emotional health cognitive behaviour therapy programme, (FRIENDS)	Spence Children’s Anxiety Scale and Culture-Free Self-Esteem Questionnaire Form B to measure desired outcome of levels of anxiety and self-esteem	No indication	High [10] G, I, E	106	/	Initial ANOVAs revealed a significant change for total anxiety (F = 5.84, df = 2,315, p = 0.003) and self-esteem (F = 2.98, df = 2,315, p = 0.052) across time. Post-hoc comparisons using Tukey’s test revealed no significant change in anxiety or self-esteem over the two pre-intervention assessments (T1—T2) but a significant change from T1 to post intervention (T3) for both anxiety (p = 0.002) and self-esteem (p = 0.040). There were no significant differences between the three and 12-month follow- up (T3–T4) suggesting that post FRIENDS gains were maintained.
Note. Detailed risk of bias assessment: (10) Participation in study was on voluntary bases for students who were motivated. This could have an impact on the results when only motivated students are considered in study									
Asthma care									
Bruzzese et al. [106]	Obs	Children in Kindergarten up to grade 5	School nurses coordinating between families, primary care providers (PCPs), and school personnel	Paediatric Asthma Caregiver’s Quality of Life Questionnaire (PACQLQ) to measure desired outcome of impact on children’s quality of life; school absence	No indication	Unclear [7] H	591	/	Relative to controls, 12-months post-test intervention students had a reduction in activity limitations due to asthma (35% vs _9%, p, .05) and days with symptoms (26% vs 39%, p. .06). The intervention had no impact on the use of urgent health care services, school attendance, or caregiver’s quality of life. There were also no improvements at 24-months postintervention.
Christian-sen et al. [21]	Obs	Children in grade 4 (age 9–12 years)	Asthma education by school nurses	Asthma knowledge test and symptoms questionnaire to measure desired outcome of severity of asthma symptoms	No indication	Unclear [7] G	42	/	Mean scores for the asthma quiz improved from 9.9 (SEM = 0.44) to 13.7 (SEM = 0.30). Peak flowmeter scores improved from 3.9 (SEM = 0.33) to 6.4 (SEM = 0.29), and inhaler technique scores improved from 2.3 (SEM = 0.26) to 4.3 (SEM = 0.26). All changes in this group were highly significant (p -< 0.00001). After 180 days, the symptom scores validated for functional asthma severity were significantly lower in the educated group (2.87 + 0.447) compared with the control group (4.36 + 0.573, p = 0.0188). Asthma-related school absences, emergency department visits, and hospitalizations showed no differences between the education and control groups
Engelke et al. [25]	Obs	Children in grade 1–12	Case management	PedsQL 3.0 SF22 to measure desired outcome of quality of life, grades to measure academic achievements, individualized goals evaluated by school nurses	Unlikely, funded by a grant from the Kate B. Reynolds Charitable Trust.	High [8] E, D(11)	143	/	The proportion of children reporting symptom and treatment problems was significantly lower at the end of the program compared to baseline (p < .01). The largest improvement in asthma symptom and treatment problems was among students who met the goal of reducing emergency department (ED) visits/hospitalizations, symptom gain: n = 42, M = 16.0, SD = 17.0; treatment problem gain: M = 13.3, SD = 15.3) compared to those who did not met the goal [symptom gain: n = 19, M = 6.2, SD = 10.2, t(54). 2.20, p = .03, *η*2 = 08; treatment problem gain: M = 1.7, SD = 9.6, t(54). 2.86, p = .01, *η*2 = .13].
Fransisco et al. [107]	Obs	Children with asthma aged 5–14 years	School nurse training, 4 key messages from EPR-3, nurses supplied with assessment tools	self-reported Inhaled corticosteroid (ICS) use and adherence, CHSA-C to measure desired outcome of impairments and inspiratory flow rate, Forced expiratory volume (FEV) and EPR-3 to assess asthma control	No indication	High [9] E, J(12), D	178	√	At enrolment, 69.7% of students had “not well-controlled” or “Very poorly controlled” asthma. Postintervention, FEV significantly improved (82.9% to 92.1% predicted), and self-reported impairment and tobacco smoke exposure significantly declined (P < .001). For Teaming Up for Asthma Control (TUAC) students enrolled in Medicaid, there was an average 12-month health care cost difference (−$1,431) compared with controls.
Note. Detailed risk of bias assessment: (11) Not all students received grades and could be compared on academic outcome; (12) Control group was not described appropriately									
Halterman et al. [68]	RCT	Children with asthma aged 3–10 years	Preventive asthma medications by school nurses	Symptom-free days	No indication	Unclear [5] B, G	530	√	Children receiving preventive medications through school had significantly more symptom-free days compared to children in the control group (adjusted difference = 0.92 days per 2 weeks) and also had fewer night-time symptoms, less rescue medication use, and fewer days with limited activity (all P < .01).
Harrington et al. [69]	RCT	Children with asthma in kindergarten up until grade 8	Nurse-administered inhaled corticosteroids (ICS)	Morning doses of ICS, Questionnaires to measure desired outcome of asthma-related morbidity, quality of life, and health-care utilization	Unlikely, funded by EJF Philanthropies	Unclear [4] G, D, F	46	√	Intervention patients reported significantly less functional limitation (42.9% vs. 73.9%, p = 0.04), adjustment to family life (23.8% vs. 56.5%, p = 0.03), and sleep loss (1.7 vs. 4.1 nights in last 2weeks, p = 0.035) than control patients at the end of the 60-days study period. There were no differences in unscheduled health-care utilization by group.
Janevic et al. [108]	Obs	Children with asthma mean age 7 in underserved communities	Medical–social care coordination program	Childhood Asthma Control Test to measure daytime and night-time symptoms	Possibly, Funded by Merck Childhood Asthma Network, Inc. (MCAN)	High [8] C, F	805	√	At follow-up, intervention participants had 2.2 fewer symptom days per month (SD = 0.3; P < .01) and 1.9 fewer symptom nights per month (SD = 0.35; P < .01) than did the comparison group. The relative risk in the past year associated with the intervention was 0.63 (95% confidence interval [CI] = 0.45, 0.89) for an emergency department visit and 0.69 (95% CI = 0.47, 1.01) for hospitalization.
Levy et al. [70]	RCT	Medically underserved inner-city children aged 6–10 years	School-based asthma case management (open airways curriculum)	Student records to measure absences, hospital utilization; knowledge tests to measure student’s knowledge and skills	No indication	Unclear [3] E, F	243	/	Case management (CM) students had fewer school absences than their counterparts in Usual care (UC) schools (mean 4.38 vs 8.18 days, respectively) and experienced significantly fewer emergency department visits (p < .0001) and fewer hospital days (p < .05) than UC students. No such differences existed before program initiation. Replication and follow-up in year 2 showed continued significant improvements.
Liptzin et al. [109]	Obs	School aged children in inner-city schools	School centered asthma program “Step-Up Asthma Program”	Inhaler technique, Open Airways for School (OAS) scare and Kickin`Asthma (KA) score to measure desired outcome of asthma knowledge, Asthma control was measured with a questionnaire.	Possibly, authors received support by different organizations	High [9] I, D	252	/	Inhaler technique, OAS scores, and modified KA scores improved significantly from baseline to the 1-year follow-up. Students who participated in the Step-Up Asthma Program experienced decreased asthma exacerbations. Emergency department and urgent care visits, use of systemic corticosteroids, and missed school days all decreased significantly from baseline. Hospital admissions decreased significantly from baseline to the 1-year follow-up (P = .002), but baseline to 2-year follow-up was not significant (P = .2).
Mickel, Shanovich [110]	Obs	Children with asthma; 7–11 years of age in a Midwest metropolitan school district	Iggy and the Inhalers—an asthma education program	Children’s knowledge and family awareness of asthma management was measured with the help of test questions	Unlikely, funded by a Wisconsin State Asthma Coalition	High [10] F, I, D, G	147	√	Asthma knowledge increased significantly (p < .001) between pre-test and post-test, and this increase was retained at 1-month follow-up. This program evaluation suggests that our program had a significant, sustained impact on students’ asthma knowledge
Moricca, Grasska [111]	Obs	Children with asthma aged 7–12 years	Screening for asthma risk status, case management	CST-ELA and CST-Math scores to measure desired outcome of academic achievements, postintervention absenteeism	No indication	Unclear [7] G	142	√	Grade did emerge as a statistically significant predictor of students’ CST-Math scores with fifth to sixth grade students outperforming second to fourth grade students (p < .001). While year, gender, and the interaction of group and year failed to serve as statistically significant predictors of postintervention absenteeism (p > .05 for each), younger children (grades 2–4) missed approximately 2 more days per year than older children (Grades 5–6) 7.1 days (SE = 0.85). versus 5.1 days (SE. 0.42), p. .019.
Noyes, Bajorska [71]	RCT	Children with asthma aged 3–10 year	1 dose of preventive asthma medication	Mean number of symptom free days and parent questionnaire to measure desired outcome of health serves utilization	No indication	Low [1] F	525	√	The health benefit of the intervention was equal to ∼158 SFD gained per each 30-day period (P, .05) per 100 children. The programmatic expenses summed to an extra $4822 per 100 children per month. The net saving due to the intervention (reduction in medical costs and parental productivity, and improvement in school attendance) was $3240, resulting in the incremental cost-savings difference of $1583 and CE of $10 per 1 extra SFD gained.
Persaud, Barnett [72]	RCT	Children with asthma aged 8–12 years (mean 10,2)	School nurse-delivered asthma self-management and skills, personal peak flow meter	Devilbiss surveyor 1 Spirometer to measure Pulmonary function, 5 questionnaires (parent’s and child’s asthma knowledge, control, feeling, attitude)	Unlikely, funded in part by Health Services and Resources Administration, U.S. Public Health Service.	Unclear [3] G, F	36	/	Both treatment and control subjects demonstrated improvement in asthma knowledge and child health locus of control: 2.2 +/- 2.3 for the intervention and .8 +/-3.5 for the control group. The groups did not differ significantly in the magnitude of this effect. The intervention group experienced a small positive change in asthma attitude (1.4 +/- 8.7), while the control group showed a small negative change of -1.4 +/-8.1. The functional impact score was unchanged for both groups.
Splett, Erickson [73]	RCT	School-aged children with asthma	Enhanced asthma management, as defined in The Healthy Learners Asthma Initiative (HLAI) procedures	Daily visit log, student medication, and health records, clinic process improvements, attendance data,	Unlikely, funded by Member Organizations of the Healthy Learners Board, Controlling Asthma in American Cities	Unclear [5] H, E(13), D(14)	1,561	/	Control schools had significantly more total visits (p = .02) and episodic visits (p = .003) than intervention schools. No significant differences were found in attendance between control and intervention schools overall or for students with or without asthma for each of the 2 study years. From 1999–2000 to 2000–2001, attendance for students without asthma increased by 0.6% or nearly half of a day. This reached statistical significance for control (p = .04) and intervention schools (p = .02). There was no significant change for students with asthma, and a significant interaction was found between asthma status and time (p = .01).
Taras, Wright [112]	Obs	Children with asthma	Case management protocol	Asthma severity, medication, peak flow, case management provided, days absent	No indication	High [10] D, G, I	1,094	/	Students with moderate/severe asthma were more likely to receive a larger number of school nurse case management activities than students with mild asthma for all three years. Students with mild and moderate/ severe asthma who received at least one nurse case management activity in Year 1 were more likely to have an asthma medication available at school and to use a peak flow meter in Year 2. Change in absentee rate from Year 1 to Year 2, and Year 2 to Year 3, showed no significant association with nurse case management the previous year.
Note. Detailed risk of bias assessment: (13) Practice changes in the intervention group could not be controlled, risk of confounding factors; (14) Attendance rate might not be an appropriate tool to measure the effectiveness of an asthma initiative									
Trivedi, Patel [113]	Obs	Children with asthma (mean age 10,5)	Daily inhaled corticosteroid at school, supervised by their school nurse	Number of emergency department visits, hospital admissions, school absences, rescue medication use	No indication	High [10] G, I	84	/	Asthma-related ED visits over a 1-year period decreased 37.5%, from a pre-intervention mean of 0.8 visits to a post-intervention mean of 0.3 visits (p < 0.001). Asthma-related hospital admissions decreased from a pre-intervention mean of 0.3 admissions to post-intervention mean of 0 admissions (p < 0.001). Asthma rescue medication refills decreased by 46.3% from the pre- to post-intervention period (p = < .001). There were also non-significant declines in school absences and oral steroid use for children enrolled.
Smoking cessation									
Cameron et al. [19]	RCT	Children in grade 6 to 8	Teacher workshop, School nurse (SN) workshop, teacher self-preparation, sn-self-preparation	Breath sample and self-reports to measure desired outcome of smoking habits	Unlikely, funded by the National Heart, Lung, and Blood Institute (NHLBI)	Low [114]	3,972	/	The overall smoking rate was 18.6% (95% confidence interval [CI] = 16.8%, 20.4%). Fewer than half (46.3%; 95% CI = 44.1%, 48.5%) of the students had never smoked by the end of grade 8. All 4 treatment conditions produced smoking rates that were less than the control group rate but were not significant. After adjustment for extrabinomial variation with the Pearson goodness-of-fit statistic, this model showed a significant interaction between condition and senior smoking rate (F4,84 = 3.88, P = .006).
Lamb et al. [67]	Obs	Pregnant African American teens aged 15–20 years	Eight group sessions “Teen fresh start”	Questionnaire and self-report to measure desired outcome of smoking behavior	Unlikely, funded by the American Nurse Foundation	High [10] G, F, D, I	9	/	While the number of subjects was too small to demonstrate significance, the team was encouraged to learn that five girls quit smoking and two cut down on their smoking. Descriptive analysis of results.
Thomson [115]	Obs	School-aged children	School-based smoking cessation programme	Questionnaire to measure	No indication	High [9] H, E	4,505–4,999	/	The target schools showed a reduction in smoking prevalence of 8% compared to a reduction of only 5.7% in the comparison schools. School E showed the highest percentage with a reduction of 14.1%. In this school smoking prevalence was 25.3 in 2006–2007 and only 11.2 in 2008–2009.The Department of Health (DoH) requires that stop smoking services collect and submit information on the number of smokers who remain abstinent for 4 weeks following their quit date. Although much good work in terms of health promotion was reported during the 2005–2006 pilot project, there were no 4-week quitters. During the Local Area Agreement (LAA), the quit rate rose from 0 to 24%.
Immunization									
Ferson et al. [35]	RCT	Children in Kindergarten and primary schools	Letters and leaflets + telephone calls	Immunization rate	Unlikely, funded by the Prince Henry Hospital Centenary Research Fund	Low [1] J(15)	239	/	Excluding children lost to follow up and those fully immunized at the start of the study, 20 (37%) of 54 were immunized following the passive intervention, and 35 (71%) out of 49 following the active intervention (P = 0.001). Receipt of the letter and leaflet was associated with an increased uptake of booster vaccination (P = 0.036). These proportions are significantly different (Yates corrected) χ2 = 10.9, P = 0.001).
Note. Detailed risk of bias assessment: (15) Excluding children lost to follow up									
Luthy et al. [116]	Obs	Children in grade 6 and 7	Educational and incentive program	Tetanus, diphtheria, and acellular pertussis (Tdap) booster compliance rates	Unlikely, funded by Association of Pediatric nurse Practitioners and Wyeth Pharmaceutical Company	High [10] G, I	958	/	The compliance rate expanded from 4% to 57% during a 4-week intervention program. Notably, the Tetanus, diphtheria, and acellular pertussis (Tdap) immunization compliance rate in the previous year (2008) was 54%. Researchers concluded that the intervention did not improve compliance rates significantly.
Toole and Perry [117]	Obs	High risk-students in inner-city schools	Immunization audits + immunization at school at no cost	Immunization rate	No indication	High [8] I	2,222	/	Immunization compliance rates have gone from an average of 64% to an average of 97%. An audit of 2,222 kindergarten records performed 5 years after the intervention indicated that less than 1% had no immunization records on file, only 2% needed immunizations, and less than 3% were out of compliance.
Vernon, Conner [118]	Obs	School-aged children	A: inviting immunization-deficient children; B: permission slips; C: health education program	Immunization rate	No indication	Unclear [7] E	5,636	/	Using an average of 38 hours of school nurse time, Method A succeeded significantly better than Method B in immunizing more immunization-deficient children and raising immunization levels, while giving fewer unnecessary immunizations. Method C did not produce significant improvement of immunization levels.
Sexually transmitted Virus Prevention									
Borawski et al. [74]	RCT	Children in 9^th^ and 10^th^ grade high schools	Be Proud! Be Responsible (BPBR) curriculum taught by school nurses and teachers	Questionnaires to measure desired outcomes of knowledge, efficacy, participants’ beliefs, perceived peer beliefs, and behavioral intentions	No indication	Unclear [5] E, J(16), G	1,357	/	Both groups reported significant improvements in Human immunodeficiency virus (HIV)/ Sexually transmitted infections (STI)/condom knowledge immediately following the intervention, compared to controls. Yet, those taught by school nurses reported significant and sustained changes (up to 12months after intervention) in attitudes, beliefs, and efficacy, whereas those taught by health education teachers reported far fewer changes, with sustained improvement in condom knowledge only.
Grandahl et al. [75]	RCT	Teenagers 16 years of age	Specific Human papillomavirus (HPV) education guided by Health belief Model (HBM)	Questionnaire based on HBM to measure desired outcome of health behavior, beliefs, knowledge awareness	No indication	Low [1] D(17)	741	√	The intervention had a significant effect on HBM total score (p = 0.003), (i.e., the students perceived more benefits of vaccination, perceived themselves to be at increased risk for an HPV infection or HPV-related disease, considered HPV-related disease a severe threat and perceived fewer barriers against HPV vaccination), with a 2.559 points higher score for the intervention compared to the control group.
Note. Detailed risk of bias assessment: (16) Only 25% of the classes could be randomly assigned to school nurses, reducing the sample size and power to detect smaller intervention effects; sample size of teacher-led classrooms was not large enough; (17) It is questionable if questionnaires on beliefs and intentions are the method of choice to measure health behavior									
Teenage pregnancy									
Chen et al. [76]	Obs	Pregnant students	Monthly visits to the school nurse (nutrition, infant care, father of baby, finance, family support, future plans, education…	Birth weight and parental care was measured by conducting month prenatal care began and frequency of visits	No indication	High [8] D, E	578	/	The program effects on birth weight have two pathways. To test the direct effect, a two-tailed, paired t test was applied to compare the difference of birth weight between cases and controls (M = 27.6g, SD = 864.7g) and the result was not statistically significant (t (287) = 0.543; P>0.05). However, the finding showed a higher mean birth weight (27.8g) for cases than for controls.
Allergies									
Spina, McIntyre [79]	RCT	Children (mean age 15 years) diagnosed with a life-threatening allergy + auto-injectable epinephrine	Education (for intervention and control), showing school nurse their epinephrine	Periodic checks and expiry checks	No indication	Unclear [4] G, E(18)	77	/	No significant differences were found between the intervention and control groups in the proportion of students who had epinephrine available during the initial check (*x*^2^ = 1.63, df = 1, p = .2) or the final check at the end of the study (*x*^2^ = 1.73, df = 1, p = .189). In the intervention group, all participants who had unexpired epinephrine during the first check continued to have current epinephrine, while one person with expired epinephrine on the first check had acquired current epinephrine during the final check (p < .01). For the control group, of the 21 participants who reported having unexpired epinephrine during the first check, 5 participants had expired epinephrine during the final check.
Nutrition									
O’Donnell et al. [77]	Obs	Children in Kindergarten to grade 6	Educating teachers and offering mini grant materials	Knowledge test on nutrition for students to measure desired outcome of effectiveness of mini grant materials	No indication	High [10] D, E, F, I, G	1,106	/	In summary, most of the classroom teachers used the nutrition resource materials, and it would appear that the availability of these nutrition resource materials caused an increase in time spent on nutrition education.
Alcohol use prevention									
Werch, Carlson [80]	RCT	Children in grade 6–8 (mean age 12,2 years) in inner-city school	STARS program (Start Taking Alcohol Risks Seriously)	Alcohol use, Alcohol consumption patterns, negative consequences experienced during drinking	Unlikely, funded in part by National Institute on Alcohol Abuse and Alcoholism	Low [2] G	138	/	A significant difference was found on heavy alcohol use with intervention subjects showing a reduction and control subjects an increase in heavy drinking (t = -2.33, I20dJ; p = .02). No differences were found between groups on other alcohol use measures.
Note. Detailed risk of bias assessment: (18) The educational intervention available to both the control and the intervention group to ensure the safety of students. The education may have had an impact on increasing the likelihood that students would carry epinephrine.									
Hearing screening									
Sekhar et al. [119]	Obs	Children in Kindergarten, 1^st^-3^rd^ grade; adolescents in grade 7 and 11	High-frequency screen and PA-screen	PA-screen to measure low-frequency, conductive hearing losses; high-frequency screen for adolescent hearing loss	Unlikely, funded by Academic Pediatric Association and Child Health Bureau	High [10] G(19), D(20), F, J(21)	282	/	Five participants (2%) were referred on the Pennsylvania school screen, and 85 (30%) were referred on the high-frequency screen. Of the 48 who returned for gold standard testing with audiology, hearing loss was diagnosed in 9/48 (19%). Sensitivity of the Pennsylvania and high frequency screens were 13% (95% confidence interval [CI] 0–53%) and 100% (95% CI 66–100%) respectively.
Cardiovascular Health									
Harrell et al. [82]	RCT	Elementary school children aged 7–12 years	Classroom-based and risk-based interventions (nutrition, physical activity and smoking)	Questionnaires to measure desired outcome of knowledge, attitude and physical activity; Health outcome was measured with BMI, cholesterol level, blood pressure, aerobic power and skinfolds	Unlikely, funded by the National Institute for Nursing Research	Unclear [3] F, E	2,109	√	Total knowledge in the classroom-based intervention schools was significantly greater than that in the control schools (7.86; 95% CI = 3.32, 12.40). The physical activity score improved significantly only in the risk-based schools (3.87; 95% CI = 1.35, 6.39). There were statistically significant differences among the 3 groups in regard to changes in cholesterol (P< .01), but only the classroom-based approach reduced mean cholesterol significantly more than in the control group. Adjusted differences among the groups were nonsignificant for systolic blood pressure but were significant for diastolic blood pressure (P = .03). Both the classroom-based and risk-based groups exhibited a significantly smaller increase in diastolic blood pressure than the control group.
Psychosomatic headaches									
Larsson and Carlsson [83]	RCT	Children aged 10–15	Nurse-administered relaxation training	Headache diary to measure headache free days	No indication	Low [2] G	26	/	Nine pupils (69%) who were treated with relaxation training had achieved a clinical improvement level (of at least 50%), whereas only one subject (8%) in the no-treatment control condition had attained such an improvement level, a difference that was highly significant, *x*^2^(1) = 10.40, p < .001. Although a small improvement was noticed in the no-treatment control group at the 6-month follow-up, the difference between the two treatment conditions was still significant, *x*^2^ = 3.94, p < .05. Pearson product-moment correlation analysis revealed a nonsignificant association between children’s age and their headache (pre—post) improvement (r = .07).
Diabetes									
Izquierdo et al. [84]	RCT	Children with diabetes aged 5–14 years	Usual care + a videoconference between school nurse, child, and diabetes team every month.	Hemoglobin A1c to measure the long-term glycaemic control and Pediatric Quality of Life (PedsQL) to measure quality of life	No indication	Low [2] G	41	/	There was a significant difference in slopes between the telemedicine and usual care groups during the first 6 months (P < .02). A1c values increased from baseline to 6 months for students in the usual care group (not statistically significant) but decreased in the telemedicine cohort (P < .02), and the improvement was maintained over the next several months. No significant differences in slopes, or within-group slopes were observed after the 6-month point.
Note. Detailed risk of bias assessment: (19) Statistically, the small number of adolescents presenting for gold standard testing resulted in wider confidence intervals around the estimates of sensitivity and specificity; (20) Time lapse between two screenings too big; (21) Expectation bias									
Nguyen, Mason [85]	RCT	Children with high levels of HbA1c	Insulin glargine, insulin aspart, OneTouch Ultra glucometer, access to the GlucoMON system	Blood glucose records during school hours; Hemoglobin A1c (HbA1c) level and BMI (to measure weight gain)	Unlikely, funded by investigator-initiated grant Rubina Heptulla from Sanofi-Aventis	Unclear [4] G, F, J(22)	36	√	The HbA1c level remained unchanged in the control group but was decreased significantly in the intervention group. For the intervention group, the rate of hypoglycemia (BG<59 mg/dL) was 0.86 +/-0.55 episode/patient-week. No data of hypoglycemia in control group. There was no difference in BMI between the 2 groups either before or after the 3-month study period. There was no correlation between the change in BMI and the change in HbA1c level in the intervention group (r = 0.24).
Teasing and Bullying									
Vessey and O’Neill [78]	Obs	Children with diabetes aged 5–14 years	Identification of at-risk students, web-based program to build resiliency, biweekly support/discussion group	Child-Adolescent Teasing Scale (CATS) measuring 4 components of teasing, Pediatric Symptom Checklist (PSC) measuring parent’s perception of psychological functioning, Piers-Harris Children’s Self-concept Scale (PHCSCS) measuring self-concept	Unlikely, funded by Deborah Munroe Noonan Memorial Research Fund	High [10] I, E	65	/	Statistically significant, positive differences were noted in students’ total scores on the CATS (t = 3.432, p = .001) and PHCSCS (t = 2.546, p = .007). These results imply that the participants, after completing the study intervention, perceived that they experienced fewer bothersome peer interactions and that they felt better about themselves, as reflected in their view of their self-concepts. Scores on the PSC, reflective of parental assessments of their children’s global psychosocial functioning, did not differ significantly following the intervention
Hygiene									
Kimel [120]	Obs	Children in Kindergarten and 1^st^ grade	Handwashing program	Absentee rates	No indication	High [8] E, F	199	/	After the intervention, absenteeism was significantly higher among non-participants. Percentages of students absent because of flu-like illness in on-participating classes were approximately double those of participating classes. Chi-square values were significant at the p = 0.05 level: x2 (1, N = 199) = 22.225, p = .001
Morton and Schultz [121]	RCT	Children in Kindergarten to 3^rd^ grade	A 45-minute ‘‘Germ Unit” and use of alcohol gel	Communicable illnesses, vacations, respiratory-, gastrointestinal illnesses and days absent	Unlikely, funded by Maine Administrative School District	Low [1] D	253	/	Using McNemar’s test for dichotomous variables with paired subjects, significantly fewer children became ill while using the alcohol gel as an adjunct to regular handwashing than when using regular handwashing only (chi square 5 7.787; p 5 .0053). The odds of being absent due to infectious illness were reduced by 43% with the adjunct use of the alcohol gel. Fewer children were absent in total during phase two.
Note. Detailed risk of bias assessment: (22) No Blood glucose records were available for the control group, because these subjects did not bring their BG logbooks to their visits as required									
Studies with mixed outcomes									
A. Challenging behavior									
Buckland et al. [122]	Obs	Children aged 4–15 (mean 8)	School nurse-led interventions (group work, class work, partnership work)	SDQ (parent and teacher perspective) to measure desired outcome of hyperactivity, peer problems, conduct, emotional symptoms and prosocial behavior	No indication	High [10] G, F, E, C, I	6	√	Changes in Total difficulty score (TDS) of 3 or more were assumed to be clinically meaningful. Based on this, all six children were reported to have improved (clinically meaningful) according to either their parents or their teacher. However, in three cases, the teacher believed that children had deteriorated marginally (by two points in each case). The mean improvement in parents TDS ratings was 5.6 and in teacher rating 1.3.
Krug et al. [86]	RCT	Children in areas with high rates of crime (Kindergarten to grade 5)	PeaceBuilders	Number of school nurse visits to measure desired outcome of level of violence	No indication	High [10] E, D, F, G, A, B	3,899	/	When the data were aggregated across the intervention schools, the weekly rate of visits for all reasons per 1,000 student days decreased by 12.6% (P < .001). The patterns for injury-related visits were the same as the patterns for visits for all reasons. Rates of confirmed fighting-related injuries did not change significantly in the intervention schools but increased 56.0% (P = .01) in the control schools.
B. Chronic Illnesses (asthma, diabetes, severe allergies, seizures, and sickle-cell anemia)									
Engelke et al. [123]	Obs	Children aged 5–19	Care Coordination (teaching, direct care, school personnel, families, referrals)	PedsQL 3.0 SF22 (asthma treatment), PedsQL 3.0 (diabetes symptoms) and PedsQL 4.0 SF15 (physical health, emotional, social functioning)	Unlikely, funded by the Kate B. Reynolds Health Care Trust	High [9] J(23), C(24), F	114	/	For children with asthma, there was a significant improvement in total quality of life (p < .001, Eta squared = .47), treatment scale (p < .001, Eta squared = .49) and the symptom scale (p = .001, Eta squared = .18). For children with diabetes, there was a significant improvement in the treatment barrier subscale (p = .01, Eta squared = .19).
C. Physical activity and mental health									
Hoying and Melnyk [124]	Obs	Children aged 11–13	Creating Opportunities for Personal Empowerment (COPE), Healthy Lifestyles Thinking, Emotion, Exercise, and Nutrition (TEEN), physical activity and mental health	Healthy lifestyle belief scale, OMRONTM pedometer to measure PA, The Beck Youth Inventory (BYI-II) to measure depressive symptoms, anxiety, anger, disruptive behavior and self-concept	Possibly, Bernadette Melnyk’s company, COPE2THRIVE, disseminating the COPE program; funded by Sigma Theta Tau	High [10] F, G, I, E	31	/	Preadolescents who received COPE reported significant decreases in anxiety. Although there was not a statistically significant improvement in healthy lifestyle beliefs, there was a small to medium positive effect. Students demonstrated significant increases in PA. The subgroup of anxious, depressed, or low self-concept preadolescents who received the COPE intervention demonstrated significant increases in self-concept and significant decreases in anxiety and depression scores. Four out of six students who scored positive for suicidal ideation at T0 no longer scored positively for suicidal ideation at T1.
Note. Detailed risk of bias assessment: (23) Difficulty retrieving data from the PDA, it was not possible to determine the number of times a nurse provided a specific intervention; (24) Grades were not available for all children, all goals were not appropriate for all children									
D. Resilience and mixed health outcomes									
Olowokere and Okanlawon [125]	Obs	Vulnerable children in secondary schools	Experimental group (E)1: resilience training, E2: peer support group (basic life skill support and sharing of feelings + coping techniques) E3: E1+E2	Adapted anxiety, depression, and self-esteem questionnaires to measure psychosocial outcomes; knowledge scores	No indication	High [8] F, G(24)	109	/	No significant difference in the anxiety scores of the children in the intervention group compared with the control group (M = 5.37, t 0.870, p = .386). Significant reduction in the depression score in the intervention group compared with the control group postintervention (M = -4.94, t = -2.26, p = .03). For self-esteem, the independent t-test showed a significant increase in self-esteem scores in the intervention group (M = 3.27, t = -.2.26, p = .03). A significant increase in social connection was also observed in the intervention group compared with the control group (M = 2.86, t = 3.16, p = .002).
Note. Detailed risk of bias assessment: (24) Study with lager power >.80 needs to be conducted to be able to generalize effectiveness of the intervention									

*CI (Confidence interval) was described “√” and can be looked at in detail in respective study; “/” is not available.

### 3.6 Results based on health outcomes

#### 3.6.1 Findings on somatic health

Among the multitude of studies there are some significant results that are worth mentioning. One RCT study [61] with a low RoB showed that when a school nurse was available the number of days a student was absent was significantly reduced (on average two days per year) compared to the control group where there was no school nurse. Other studies examining school attendance were less reliable due to significant limitations (e.g. RoB).

*Asthma*. The majority of school nurse research examines asthma-related interventions, some providing meaningful results. The outcome variables studied vary greatly, which complicates the comparability of the results. Often research groups investigate more than one outcome such as the quality of life [69,106], school absenteeism [70,73,106,112,113], asthma knowledge [21,70,72,109,110], severity of asthma symptoms [21,108,112], quality of life [25], academic achievements [25,111], individualized goals [25], impairments and inspiratory flow rate [107], asthma control [72,107–109], symptom-free days [68,71], medication [69,112,113], health-care utilization [69–71,113], pulmonary function [72] and clinic process improvements [73]. While the heterogeneity in asthma research is striking (e.g. various health outcomes, populations, and types of interventions), a fair number of studies are of high quality with only a few limitations. One study with good reliability (low RoB) showed that preventive asthma medication for 530 students resulted in a significant reduction in symptoms compared to the control group. In addition, there were fewer night-time symptoms and rescue medication use and more peace of mind for the children [68]. Another study showed that nurse-administered inhaled corticosteroids (ICS) resulted in significantly fewer functional limitations, better adjustment to family life and improved sleep compared to the control group [69]. Results investigating asthma case management on medically underserved inner-city children show that there was less school absenteeism among children in the intervention group compared to the control group. They also experienced significantly fewer emergency department visits and fewer hospital days. A replication and follow-up in year 2 also showed continued significant improvements [70].

*Obesity*. In assessing the effectiveness of school nurse-led obesity prevention studies, j = 4 references with low risk of bias were of significance. All studies had in common that the BMI score was one of the main outcome measures, but the type of interventions to reduce the BMI score differed. One intervention consisted of educational programs on nutrition, an improved environment with healthy foods and more physical activity and screening procedures [17,62], but results showed no clear advantage for children in the intervention group. Although slight reductions in BMI were found, education and environmental strategies had no effect on BMI compared to the control group. Another intervention consisted of integrated health education for teachers and nutrition counselling for parents [63]. Risk of bias was unclear for this study due to lack of blinding and failure to adequately control confounding, so that results may be less reliable. However, results showed that overweight children achieved a significant reduction in weight after two years compared to the control group. School grades deteriorated in both groups, but the deterioration was less dramatic in the experimental group. In a third intervention conducted in 2013 [64] and replicated three years later [64,65], computerized cognitive behavioral counselling and an after-school physical activity program were implemented. In addition to BMI scores, diet, physical activity, sedentary behavior, self-efficacy, and perceived barriers were measured. Results showed no significant changes in BMI, body fat and waist circumference compared with control schools. A different way of looking at obesity prevention is to increase physical activity. One study investigated the impact of a Kids N Fitness intervention, with 45-minute physical activity sessions, nutrition education and wellness activities [66]. Children were asked to complete a questionnaire about their diet and anthropometric measures were collected. The study had a low risk of bias and the results showed that the intervention (delivered by school nurses) had a significant impact on BMI, sedentary behavior in boys and increased physical activity behavior in girls.

*Diabetes*. In assessing the effectiveness of school nurse-led diabetes management interventions, two references with low [84] and unclear [85] risk of bias were significant. One study used the hemoglobin A1c (HbA1c) value to measure long-term glycemic control. Results showed that monthly videoconferences between school nurse, child, and diabetes team showed significant improvements in the first 6 months. However, no further significant improvements were observed after the 6-month mark [84]. In the second study, blood glucose levels, HbA1c and BMI were measured and compared between experimental and control groups. Results showed that the HbA1c level remained unchanged in the control group but was significantly lower in the intervention group [85]. Neither group showed a significant change in BMI. Other investigated health outcomes such as teasing and bullying [78], resilience [125] and hyperactivity and peer problems [126] were not reliable due to limitations.

*Sexually transmitted illnesses*. Research groups that have set out to study sexually transmitted illness (STI) prevention interventions are consistent in that the interventions consist of educational programs for students. The outcomes measured by questionnaires relate to knowledge, beliefs, and behavioural intentions [74,75]. Despite the lack of adequate follow-up, both studies showed significant improvements in knowledge, attitudes and beliefs compared to the control group.

*Vaccination*. All studies on vaccination used immunisation rates as an outcome measure. Study interventions ranged from information brochures [35] to educational programmes [116] to free vaccination programmes [117,118] and results showed improved immunization rates across all studies.

*Smoking*. A reliable study on smoking cessation showed that properly educating teachers and school nurses had a significant impact on lowering smoking among school children [19]. Other studies examining smoking habits relied on self-reported questionnaires which however tends to lead to distortion [115,127].

#### 3.6.2 Findings on mental health

There are several studies that examine the impact of school nurse-led interventions on mental health. Only two studies were considered adequate for assessing the effectiveness of school nurse-led programs, since the others showed limitations that could affect the validity of the results.

*Anxiety*. One study examined the impact of a computerized cognitive therapy (cCBT) intervention on anxiety [14], the other the impact of screening and coping skill interventions on depressive symptoms [67]. Anxiety was measured with the Anxiety Scale (SCAS) and the Strengths and Difficulties Questionnaire (SDQ) and results showed a significant reduction in the total child anxiety rate after the cCBT intervention [14].

*Depression*. Depression and coping skills were measured using the Reynolds Adolescent Depression Scale (RADS) and the Jalowiec Coping Scale (JCS) but the risk of bias was unclear, as the study sample consisted of only 40 participants [67]. Nevertheless, the results of the RADS showed that almost 87% of the students in the intervention group experienced a reduction in depressive symptoms, compared to a 60% reduction in the students of the control group [67].

## 4 Discussion

With 16 reviews and 289 primary studies, the present study indicates a saturated field of research in the field of school nursing. However, in the quality analysis conducted, the opposite is observed, as the number of high quality and meaningful studies must be considered low. First, it is striking that there is no consistently established classification system of school nursing terms, which is why research is based on individual views of the effectiveness of school nurses. This results in a wide collection of literature that makes a judgment about the effectiveness of the school nurse very difficult. The present paper presents a standardized classification of school nursing work fields according to their approaches and strategies in their goals to make the school a health promoting environment. In addition, results of this paper will be discussed against the background of qualitative limitations, followed by an outlook for research in the field of school nursing.

### 4.1 Barriers in assessing the effectiveness of the school nurse

School nursing research faces many hurdles that need to be identified and discussed. The coverage ratio of school nurses in schools has a major impact on the effectiveness of school nurses in schools and has so far been inadequately represented in impact analyses. The background to this assumption is a study by Paterson and Zderad [128] who indicate that the supply key could highly impact successful care taking. Their results show that the key to successful patient care is the relationship between the nurse and the patient [128]. This humanistic theory can be applied to the school setting, which increases the importance of the school nurse’s supply key in schools. This assumption is supported by researchers who showed that smaller nurse-to student ratio lead to better student outcomes [129]. The recommended nurse-to-student ratio is 1:750 according to the Centers for Disease Control and Prevention (CDC) but hardly ever realized in most schools across America [55]. The effect of the coverage key on a school nurse intervention has so far been an understudied aspect and may be a relevant variable in evaluating the effectiveness of school nurse interventions.

A further hurdle in school nurse research is the ethical aspect of school nurse interventions. It is hardly ethical that children with high care needs are assigned to the control group and do not receive any intervention. On the other hand, the lack of a control group leads to a severe loss of study quality [44,101].

Furthermore, it is difficult to attribute the effectiveness of school nurse to a single intervention. Instead, a variety of factors may play a role that could influence the effectiveness of the school nurse intervention. Excluding confounding variables is nearly impossible in the school setting and in the research field of interpersonal interactions but a strict quality criterion according to the GRADE guidelines. The crux of the matter is whether school nurse research can find a way to satisfy both the holistic nature of school nurse work *and* meet the scientific criteria. Results show that this is possible in certain niches of school nurse activities. For example, school-nurse led immunization projects [35] and hygiene projects [121] show great success. This is especially important in a time of global pandemic, where high immunization rates are crucial for the overall health of society. In light of these findings preventative measures (aspect 3) through immunization and hygiene interventions by school nurses may be a future area of focus for school nurse efficacy analyses.

In contrast, studies focusing on the increasing trend of obesity, do not yet show sufficiently clear evidence in favor of appointing school nurses. This may be because measuring BMI is not a satisfactory way of assessing the body weight of children and adolescents, which naturally increases with age [17], but also that underlying variables such as depression or family difficulties (low socio-economic status) allied with poor nutritional understanding may be the underlying causes of obesity. This raises the interesting question of whether the increasing trend towards overweight can be reversed by education [65] (aspect 1) and physical activity programs [124] (aspect 3), or whether overweight children have other underlying problems, such as mental issues (aspect 2), which sometimes go unrecognized for a long time. For example, if children are already overweight, they are often bullied by their peers, which in turn puts the child under a lot of emotional stress, potentially causing mental health problems. Escaping this cycle and intervening early through preventive measures is a major challenge.

There are few well-designed studies examining mental health in school children. The reason for this needs to be explored in school nurse research. One reason might be that mental illnesses are very complex and take a variety of forms. Depending on the type and severity of the illness, effective treatment, which has to be tailored to the individual’s needs, often takes many years. On the other hand, the effectiveness of preventive measures can only be measured in long-term studies and research resources are often insufficient [130]. Furthermore, it is noticeable that in some studies the control group does not receive a pure placebo and screening procedures are often applied, which could indeed amount to an intervention effect [14,67]. This could possibly explain the lack of any significant difference between the control and experimental group [67]. Another difficulty in measuring mental health interventions is ensuring allocation concealment and blinding. Unlike medical trials, where a group may be given a placebo drug, in school nurse research it is obvious when a group is not receiving an intervention [67]. Attempts have been made to minimize this bias by randomly assigning whole schools to an experimental or control group [65], although this runs the risk of studying geographically or socio-economically disparate groups. Another difficulty in assessing the effectiveness of school nurses in dealing with mental health may be the different levels of qualification for the otherwise medical-oriented school nurse work. How and whether the school nurse has received specialized training differs in each research project, making comparisons downright impossible.

Research groups focusing on the implementation of asthma-care-related interventions for school children face other challenges. Here, the high degree of heterogeneity in these studies is striking and the research pool has so far been inconsistent regarding the measurement tools, interventions or populations studied. However, asthma research has a major advantage in efficacy analyses, namely the close proximity to medicine, where interventions are somewhat more controlled through the administration of medication by the school nurse, for example nurse-administered inhaled corticosteroids (ICS) [69]. Likewise, diabetes studies show promising results in that Hemoglobin A1c can be controlled by school nurses and the values to be measured (e.g. blood glucose level) are largely free of possible bias. Researchers are taking advantage of this fact and study results show that school nurses are of great benefit in improving diabetes management in children [84,85].

### 4.2 Effectiveness of school nurse interventions

**Fig 7:**
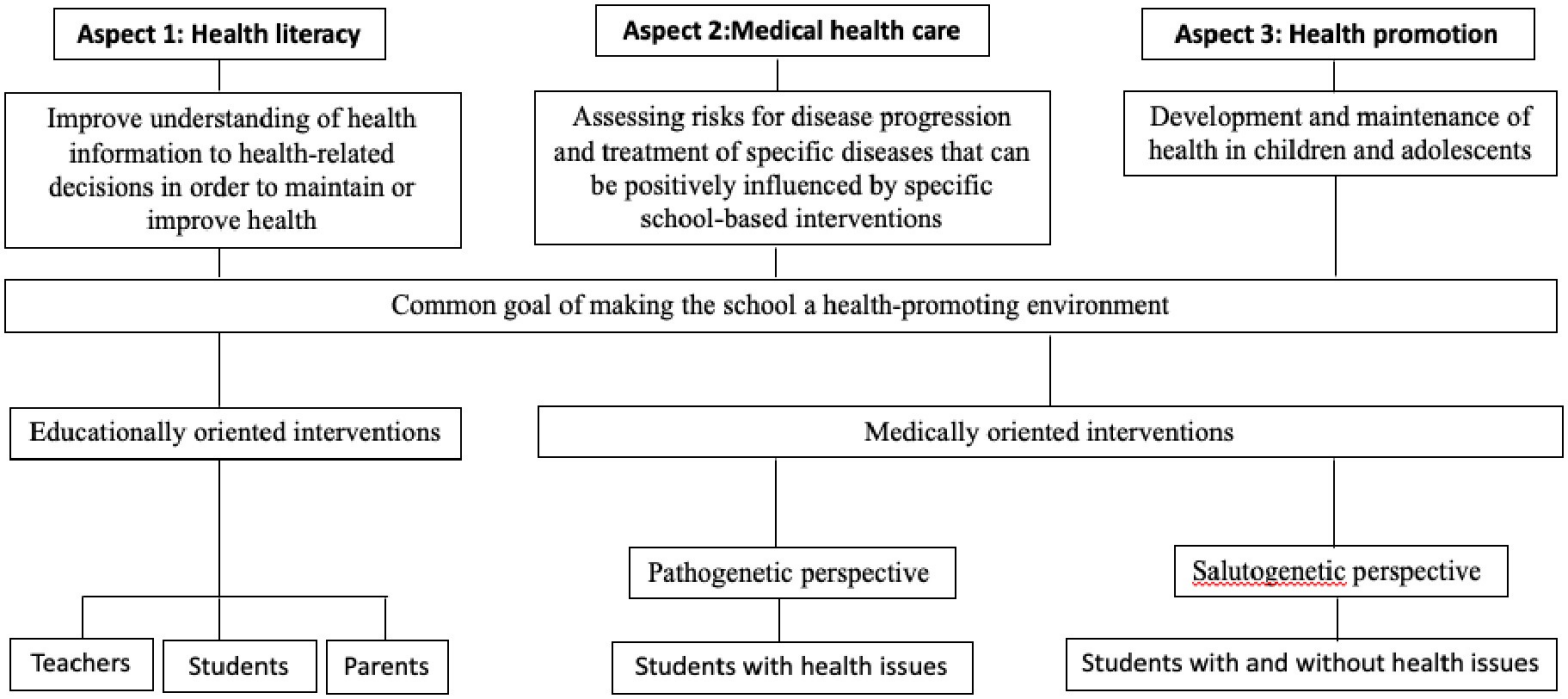
Classification of the school nurse’s working fields according to their approaches and perspectives. Notes. Three core aspects of school health 1) health literacy, 2) medical health care, and 3) health promotion complement each other in terms of the common goal of making the school a health-promoting environment but differ in their approaches and strategies to achieve this goal.

In the presents paper the authors have examined the effectiveness of the school nurse. Based on a classification of school nurse strategies and approaches, the effectiveness of the school nurse was evaluated from two of the three aspects (see Fig 7), namely from the standpoint of medical health care interventions (aspect 2) analyzing the effectiveness of the school nurse for children suffering from asthma, diabetes, obesity, anxiety, depression, and students smoking, and also from the standpoint of health promotion (aspect 3) analyzing the effectiveness of the school nurse in combating sexually transmitted diseases (STD) and increasing vaccination rates. The evaluation of school-based interventions included a quality assessment. Present findings are largely consistent with findings by other researchers across the globe who have previously criticized the lack of methodological quality and therefore the lack of robust, meaningful results in this field [3,39]. The main point of criticism is the paucity of reliable, evidence-based, quantitative data using randomized controlled trial designs [39,40,47]. The present paper acknowledges this criticism and therefore presents a more nuanced discussion. Results of the present work show that there are indeed meaningful studies that are evidence-based with randomized controlled trial designs that cover a variety of health outcomes [17,19,35,38,61–67,71,72,75,79,80,82–85,121,131]. However, school nurse research lacks reliability because for most of the respective health outcomes no comparative studies were identified through our research findings. Contrary to the opinion of other researchers [3,39], the present paper allows conclusions about the effectiveness of the school nurse for students with asthma and diabetes. There are compelling studies that confirm the effectiveness of the school nurse in asthma care [70,71]. In the area of diabetes, the effectiveness of the school nurse can also be proven [84,85]. Results of the present work conclude that measurement methods based on physiological parameters, such as blood glucose in diabetes prevention or peak flowmeter scores in asthma care, yield more meaningful results. A possible key point could be the proximity to medical research.

This *Overview of Reviews* shows that there are some well- and many not so well-researched health outcomes in school nurse research. The general debate on quality assessment strategies and research approaches in school nurse research need to be addressed continuously. Despite scientific challenges results of the present paper speak for the implementation of school nurses, especially in countries where school nurses have not been established, such as Germany. Here, large-scale, long-term studies to evaluate the effectiveness of the school nurse in her various functions seem promising [29,132].

### 4.3 Methodological limitations of *overviews of reviews*

*Overview of reviews* is a suitable tool for summarizing evidence in a particular field. However, there are some methodological limitations that need to be mentioned. Although the most recent study in this *overview of reviews* was published in 2021 [54], it cannot be guaranteed that the compiled evidence is complete and up-to-date [133]. Furthermore, the strength of a *review* is only as good as the *primary studies* it is drawn from [40]. If relevant *primary studies* are missing from a *review*, because they were not available or could not be identified by the review team, these findings will also be missing from this paper. It is striking that school nurse research lacks adequate quality evaluations, for both *primary studies* and *reviews*. *Reviews* included in this paper could not meet the strict guidelines of the AMSTAR-2 criteria, which is why the quality rating had to be adjusted downwards in order to still allow a determination about the quality of the *reviews*. This change in quality rating is not validated and risks biasing the results. For future research in educational and psychological research, there is a need for quality assessment tools that includes quality requirements that can be realistically met by researchers in educational or psychological contexts.

Studies with study designs other than RCTs or Obs were excluded from this study due to high methodological demands. It is worth considering whether studies with other study designs (e.g., qualitative study designs) provide relevant results that could not meet the strict qualitative guidelines of this study but nevertheless contribute meaningful information to school nurse research on the effectiveness of school nurses.

Another problem in the methodology of *overview of reviews* is the scope of the included *primary studies* which deviates from the subject-matter of the *overview of reviews*, sometimes dramatically. Ballard and Montgomery refer to this problem as "scope mismatch" [133]. In this *overview of reviews*, all *primary studies* included in each *review* were listed and tabulated. It is debatable whether the decision to include all *primary studies* adds value to the results.

### 4.4 Additional strengths and limitations of this *overview of reviews*

This is the first *overview of reviews* on the effectiveness of school nurses and undertakes a detailed analysis of studies for both *primary studies* and *reviews*. It shows that there is a clear need for a rigorous examination of the evidence on school nurses and a critical appraisal of the quality of research. All *reviews* dealing with the effectiveness of school nurses were included in this paper, but additional recent *primary studies* were not included. This paper is based on a comprehensive search, rigorous selection and data extraction and contains an assessment of the risk of bias of the included studies. Risk of bias in this study was limited to RCTs and Obs. This paper conducted a detailed analysis of current research on the effectiveness of school nurses and provides a sound basis for future research.

However, there are several limitations to this *overview of reviews*. The quality of *reviews* included was disappointing, as the database of *primary studies* does not allow for meta-analyses. A large number of the *primary studies* failed to provide convincing evidence on the effectiveness of school nurses. A precise assessment of the body of evidence for each health outcome was not possible due to lack of data (e.g. confidence interval). It should also be noted that the majority of the studies do not specify the ratio between school nurses and children. The supply key could be a possible confounding variable that cannot be controlled due to the absence of more information on this point. Furthermore, it should be mentioned that in multifaceted interventions it is not possible to determine which components are responsible for the observed effects. This is a fundamental problem of school nurse research. Additionally, the lack of a classification system of school nursing terms also effects this paper. Future research projects should choose their approach based on the classification system presented so that study designs can be developed based on the classification system. The final limitation is that the wealth of literature provides a sense of the effectiveness and relevance of the school nurse concept, while yet not being sufficient for policy decision purposes.

However, it is worth mentioning that in this *overview of reviews* the circle of evidence is closed by linking all sources of evidence together—even literature of lower quality or with less important interventions are included in the scientific discourse [134].

## 5 Conclusions

It is clear that school nurses play a key role in improving the health of children with asthma and diabetes. Research results on the effectiveness of school nurses in the context of combating obesity is less certain and calls for more conclusive research. The analysis of the *overviews* shows that the quality of the included *primary studies* is poor (few RCT and Obs). The evaluation of the *primary studies* shows that variables such as blood glucose or asthma labeling led to higher quality results. This gain in knowledge is invaluable for policy planners and scientists.

### 5.1 Conclusions for policy planners

The concept of the school nurse is known worldwide, although it is implemented differently in each country, even in each school. Differences relate to school nurse training, staffing ratios, qualifications and responsibilities. The heterogeneous deployment of the school nurse and differences in the implementation of the studies (e.g. school nurse as part of regular care vs. school nurse as part of a research project) complicate the comparability of study results. Nevertheless, our study shows the effectiveness of the school nurse for children with asthma and diabetes. In order to elevate the school nurse to a permanent component of standard medical care, evidence-based results in different areas of children’s mental and physical health are needed. This paper represents an initial contribution and recommends further evaluation of the effectiveness of the school nurse in light of existing studies.

Areas that have been insufficiently studied or cannot demonstrate evidence of such effectiveness are those dealing with mental health or problems associated with children from low socioeconomic backgrounds. The reasons for this lacuna need to be identified in future studies. One reason for the insufficient number of studies could be that studies on mental health are lengthy and costly, and they also do not meet the strict qualitative standards of research (RCTs or Obs). For ethical reasons, a control group often cannot be justified, and the practicablity of a control group in mental health studies is in most cases not implementable (e.g. lack of allocation concealment, lack of blinding).

Research groups studying school nurse effectiveness with respect to mental health or social inequity should consider setting other qualitative standards, such as including qualitative study designs in evaluating school nurse effectiveness. Policy planners should be aware of the practicablity and implementability of studies in these areas so as not to overlook the potential effectiveness of school nurses.

### 5.2 Conclusions for scientists

Randomized clinical trials certainly have their place, but voices from the social research community (e.g., APA) are growing louder, arguing that researchers would get better results if they used other methods as well (Clay, 2010). Over the past two decades, a movement towards alternative guidelines for the design and evaluation of complex interventions has emerged from the Medical Research Council (MRC). These guidelines are intended to help researchers choose appropriate methods, make the design of evaluations understandable, and weigh the findings from intervention studies in light of methodological and practical limitations. The authors of this paper argue that school nurse research should also engage with the MRC guidelines with a view to standardizing school nurse research as well as to establishing universal standards of school nurse research. Thus, in addition to RCTs and Obs, studies with other study designs can be integrated into the scientific discourse and provide an evidence-based contribution to the field of school nurse research.

## Supporting information

S1 Checklist (DOCX)

S1 Table All identified primary studies (j = 352). (DOCX)

S2 Table Characteristics of primary studies (k = 289) included. (DOCX)

S1 File EndNote primarystudies. (ENL)

S2 File EndNote reviews. (ENL)
