# Supplementary material for: The current state of international research on the effectiveness of school nurses in promoting the health of children and adolescents: An overview of reviews
Source: PLoS One. 2023 Feb 22;18(2):e0275724. doi: 10.1371/journal.pone.0275724 (PMC9946271; doi:10.1371/journal.pone.0275724)
Supplement: S1 Table — (DOCX) [file pone.0275724.s002.docx]

S1 Table. All identified primary studies (j=352)

|  | Best, Oppewal (1) | Guarinoni and Dignani (2) | Harding, Davison-Fischer (3) | Isik and Isik (4) | Lineberry and Ickes (5) | Maughan (6) | McClanahan and Weismuller (7) | Schmitt and Görres (8) | Schroeder, Travers (9) | Stefanowicz and Stefanowicz (10) | Stock, Larter (11) | Tanner [12] | Tilley and Chambers (13) | Turner and Mackay (14) | Wainwright, Thomas (15) | Yoder (16) |  |
| --- | --- | --- | --- | --- | --- | --- | --- | --- | --- | --- | --- | --- | --- | --- | --- | --- | --- |
| Total number of references according to Reviews | 65 | 9 | 21 | 12 | 30 | 15 | 25 | 34 | 11 | 12 | 15 | 15 | 0 | 29 | 48 | 16 | 357 |
| Secondary literature identified in Reviews |  |  |  |  |  |  |  | -3 |  | -2 |  |  |  |  | -1 |  | -6 |
| Other reasons | -1 |  |  |  |  |  |  |  | +2 |  |  |  |  |  |  |  | +1 |
| Primary studies identified in Reviews | 64 | 9 | 21 | 12 | 30 | 15 | 25 | 31 | 13 | 10 | 15 | 15 | 0 | 29 | 47 | 16 | 352 |
| No Autor [17] |  |  |  |  |  |  |  | X |  |  |  |  |  |  |  |  | 1 |
| Adams (18) |  |  |  |  |  |  |  |  |  |  |  |  |  |  | X |  | 1 |
| Alizadeh, Törnkvist (19) |  |  | X |  |  |  |  |  |  |  |  |  |  |  |  |  | 1 |
| Allen (20) |  |  |  |  | X | X |  |  |  |  |  |  |  |  |  | X | 3 |
| Allen, Henselman (21) | X |  |  |  |  |  |  |  |  |  |  |  |  |  |  |  | 1 |
| Allensworth and Bradley (22) |  |  |  |  |  |  |  |  |  |  |  |  |  |  | X |  | 1 |
| Allison, Nativio (23) | X |  |  |  |  |  |  |  |  |  |  |  |  |  |  |  | 1 |
| Amillategui, Calle (24) |  |  |  |  |  |  |  |  |  | X |  |  |  |  |  |  | 1 |
| Anderson (25) |  |  |  |  |  |  |  | X |  |  |  |  |  |  |  |  | 1 |
| Antonelli and Antonelli (26) |  |  |  |  |  |  | X |  |  |  |  |  |  |  |  |  | 1 |
| Antonelli, McAllister (27) |  |  |  |  |  |  | X |  |  |  |  |  |  |  |  |  | 1 |
| Antonelli, Stille (28) |  |  |  |  |  |  | X |  |  |  |  |  |  |  |  |  | 1 |
| Anyanwu (29) |  |  |  |  |  |  |  | X |  |  |  |  |  |  |  |  | 1 |
| Aruda, Kelly (30) |  |  |  | X |  |  |  |  |  |  |  |  |  |  |  |  | 1 |
| Atherton (31) |  |  |  |  |  |  |  |  |  |  |  |  |  | X |  |  | 1 |
| Attwood, Meadows (32) |  |  |  |  |  |  |  |  |  |  |  |  |  | X |  |  | 1 |
| Badger and Brown (33) |  |  |  |  |  |  |  | X |  |  |  |  |  |  |  |  | 1 |
| Bagnall (34) |  |  |  |  |  |  |  |  |  |  |  |  |  |  | X |  | 1 |
| Bagnall (35) |  |  |  |  |  |  |  |  |  |  |  |  |  |  | X |  | 1 |
| Baisch, Lundeen (36) |  |  |  |  | X |  |  |  |  |  |  |  |  |  |  |  | 1 |
| Baker, Hebbeler (37) |  |  |  | X |  |  |  |  |  |  |  |  |  |  |  |  | 1 |
| Baldwin (38) |  |  |  |  |  |  |  |  |  |  | X |  |  |  |  |  | 1 |
| Bannink, Broeren (39) |  |  |  |  |  |  |  |  |  |  |  | X |  |  |  |  | 1 |
| Barnard-Brak, Stevens (40) |  |  |  | X |  |  |  |  |  |  |  |  |  |  |  |  | 1 |
| Barrett (41) |  |  |  |  |  |  | X |  |  |  |  |  |  |  |  |  | 1 |
| Bartfay and Bartfay (42) |  |  |  |  |  |  |  |  |  |  | X |  |  |  |  |  | 1 |
| Bednarz (43) |  |  |  |  |  |  |  | X |  |  |  |  |  |  |  |  | 1 |
| Bergren [44] |  |  |  |  |  |  |  |  |  |  |  |  |  |  | X |  | 1 |
| Bergren (45) |  |  |  |  |  |  |  |  |  |  |  |  |  |  | X |  | 1 |
| Bergren and Mehl (44) |  |  |  |  |  |  |  |  |  |  |  |  |  |  | X |  | 1 |
| Bergren and Murphy (46) |  |  |  |  |  |  |  |  |  |  |  |  |  |  | X |  | 1 |
| Bergren (47) | X |  |  |  |  |  |  |  |  |  |  |  |  |  |  |  | 1 |
| Betz [48] |  |  |  |  |  |  | X |  |  |  |  |  |  |  |  |  | 1 |
| Bhardwa (49) |  |  |  |  |  |  |  |  |  |  |  |  |  | X |  |  | 1 |
| Blaakman, Cohen (50) | X |  |  |  |  |  |  |  |  |  |  |  |  |  |  |  | 1 |
| Blackwell, Robinson (51) | X |  |  |  |  |  |  |  |  |  |  |  |  |  |  |  | 1 |
| Bolton (52) |  |  |  |  |  |  |  |  |  |  |  |  |  |  | X |  | 1 |
| Bonaiuto (53) |  |  |  |  |  |  |  |  |  |  |  |  |  |  | X |  | 1 |
| Bonaiuto (54) |  |  |  |  |  |  | X | X |  |  |  |  |  |  |  |  | 2 |
| Bonny, Britto (55) |  |  |  |  |  | X |  |  |  |  |  |  |  |  |  |  | 1 |
| Bonsergent, Agrinier (56) |  |  |  |  |  |  |  |  | X |  |  |  |  |  |  |  | 1 |
| Bonsergent, Thilly (57) |  |  |  |  |  |  |  |  | X |  |  |  |  |  |  |  | 1 |
| Borawski, Tufts (58) | X |  |  |  |  |  |  |  |  |  |  |  |  |  |  |  | 1 |
| Boyer‐Chuanroong and Deaver (59) |  | X |  |  |  |  |  |  |  |  |  |  |  |  |  |  | 1 |
| Bradley (60) |  |  |  |  |  |  |  | X |  |  |  |  |  |  | X |  | 2 |
| Bradley (61) |  |  |  |  |  |  |  |  |  |  |  |  |  |  | X |  | 1 |
| Brindis, Sanghvi (62) |  |  |  |  |  |  |  | X |  |  |  |  |  |  |  |  | 1 |
| British Pediatric Association [63] |  |  |  |  |  |  |  |  |  |  |  |  |  |  | X |  | 1 |
| Brosnan (64) |  |  |  |  |  |  |  |  |  |  | X |  |  |  |  |  | 1 |
| Brother (65) |  |  |  |  |  |  |  |  |  |  |  |  |  |  | X |  | 1 |
| Broussard (66) |  |  |  |  |  |  |  | X |  |  |  |  |  |  |  |  | 1 |
| Brustrom [67] |  |  |  |  |  |  | X |  |  |  |  |  |  |  |  |  | 1 |
| Bruzzese, Evans (68) |  |  |  |  |  |  |  |  |  |  |  |  |  |  |  | X | 1 |
| Bryan and Cook (69) |  |  |  |  |  | X |  |  |  |  |  |  |  |  |  |  | 1 |
| Bucher, Dryer (70) |  |  |  |  | X |  |  |  |  |  |  |  |  |  |  |  | 1 |
| Buckland, Rose (71) |  |  |  |  |  |  |  | X |  |  |  |  |  | X |  |  | 2 |
| Butler (72) |  |  |  |  |  |  |  |  |  |  |  |  |  | X |  |  | 1 |
| Cady [73] |  |  |  |  |  |  | X |  |  |  |  |  |  |  |  |  | 1 |
| Cameron, Brown (74) |  |  |  |  |  | X |  |  |  |  | X |  |  |  |  |  | 2 |
| Carpenter, Lachance (75) | X |  |  |  |  |  |  |  |  |  |  |  |  |  |  |  | 1 |
| Carter (76) |  |  |  |  |  |  |  |  |  |  |  |  |  |  | X |  | 1 |
| Chally (77) |  |  |  |  |  |  |  |  |  |  | X |  |  |  |  |  | 1 |
| Chase, Chalmers (78) |  |  | X |  |  |  |  |  |  |  |  |  |  |  |  |  | 1 |
| Chen, Fitzgerald (79) |  |  |  |  |  | X |  |  |  |  |  |  |  |  |  |  | 1 |
| Chilvers (80) |  |  |  |  |  |  |  |  |  |  |  |  |  | X |  |  | 1 |
| Chokshi, Patel (81) | X |  |  |  |  |  |  |  |  |  |  |  |  |  |  |  | 1 |
| Christiansen, Martin (82) |  |  |  |  |  |  |  |  |  |  | X |  |  |  |  |  | 1 |
| Cicutto, To (83) |  |  |  | X |  |  |  |  |  |  |  |  |  |  |  |  | 1 |
| Clapp (84) |  |  |  |  |  |  |  |  |  |  |  |  |  | X |  |  | 1 |
| Clarke (85) |  |  | X |  |  |  |  |  |  |  |  |  |  |  |  |  | 1 |
| Clausson and Berg (86) |  |  |  |  |  |  |  |  |  |  |  | X |  |  |  |  | 1 |
| Coates (87) |  |  | X |  |  |  |  |  |  |  |  |  |  |  |  |  | 1 |
| Cohen [88] |  |  |  |  |  |  |  |  |  |  |  |  |  |  | X |  | 1 |
| Coleman and Hawkins (89) |  |  |  |  |  | X |  |  |  |  |  |  |  |  |  |  | 1 |
| Costante (90) |  |  |  |  |  |  |  |  |  |  |  |  |  |  | X |  | 1 |
| Cox, Fritz (91) |  |  |  |  |  |  |  |  |  | X |  |  |  |  |  |  | 1 |
| Crickmore, Jones (92) |  |  |  |  |  |  | X |  |  |  |  |  |  |  |  |  | 1 |
| Davies (93) |  |  |  |  |  |  |  |  |  |  |  |  |  | X |  |  | 1 |
| Davis (94) |  |  |  |  |  |  |  |  |  |  |  |  |  | X |  |  | 1 |
| Davis, Varni (95) | X | X |  |  |  |  |  |  |  |  |  |  |  |  |  |  | 2 |
| DeSocio [96] |  |  |  |  | X |  |  |  |  |  |  |  |  |  |  |  | 1 |
| Diao et al. [97] | X |  |  |  |  |  |  |  |  |  |  |  |  |  |  |  | 1 |
| Dixon (98) |  |  |  |  |  |  |  |  |  |  |  |  |  | X |  |  | 1 |
| Dodds (99) |  |  |  |  |  |  |  |  |  |  |  |  |  | X |  |  | 1 |
| Doggett, Faulkner (100) |  |  |  |  |  |  |  | X |  |  |  |  |  |  |  |  | 1 |
| DoH (101) |  |  |  |  |  |  |  |  |  |  |  |  |  |  | X |  | 1 |
| Downie, Chapman (102) |  |  |  |  |  |  |  | X |  |  |  |  |  |  |  |  | 1 |
| Driscoll, Volkening (103) |  |  |  |  |  |  |  |  |  | X |  |  |  |  |  |  | 1 |
| Eisbach and Driessnack (104) |  |  | X |  |  |  |  |  |  |  |  |  |  |  |  |  | 1 |
| Engelke, Guttu (105) |  |  |  |  |  |  | X |  |  |  |  |  |  |  |  |  | 1 |
| Engelke, Guttu (106) |  |  |  |  |  |  | X | X |  |  |  |  |  |  |  | X | 3 |
| Engelke, Swanson (107) | X |  |  | X |  |  | X |  |  |  |  |  |  |  |  |  | 3 |
| Engelke, Swanson (108) | X |  |  |  |  |  |  |  |  |  |  |  |  |  |  |  | 1 |
| Engh, Rahm (109) |  |  | X |  |  |  |  |  |  |  |  |  |  |  |  |  | 1 |
| Engh and Eriksson (110) |  |  | X |  |  |  |  |  |  |  |  |  |  |  |  |  | 1 |
| Fagan (111) |  |  |  |  |  |  |  |  |  |  |  |  |  |  | X |  | 1 |
| Ferson, Fitzsimmons (112) |  | X |  |  |  | X |  |  |  |  | X |  |  |  |  |  | 3 |
| Few (113) |  |  |  |  |  |  |  |  |  |  |  |  |  |  | X |  | 1 |
| Foster [114] |  |  |  |  | X |  |  |  |  |  |  |  |  |  |  |  | 1 |
| Fox, Cowell (115) |  |  |  |  |  |  |  |  |  |  | X |  |  |  |  |  | 1 |
| Fox, Rossetti (116) |  |  |  |  |  |  |  |  |  |  |  | X |  |  |  |  | 1 |
| France (117) |  |  |  |  |  |  |  |  |  |  |  |  |  | X |  |  | 1 |
| Francisco, Rood (118) | X |  |  |  |  |  |  |  |  |  |  |  |  |  |  |  | 1 |
| Fryer Jr and Igoe (119) |  |  |  |  |  | X |  |  |  |  |  |  |  |  |  |  | 1 |
| Fryer Jr and Igoe (120) |  |  |  |  |  |  |  | X |  |  |  |  |  |  |  |  | 1 |
| Gaffrey and Bergren (121) |  |  |  |  |  |  |  | X |  |  |  |  |  |  |  |  | 1 |
| Garwick, Svavarsdóttir (122) | X |  |  | X |  |  |  |  |  |  |  |  |  |  |  |  | 2 |
| Gilman, Williamson (123) |  |  |  |  | X |  |  |  |  |  |  |  |  |  |  |  | 1 |
| Gordon, Colby (124) |  |  |  |  |  |  | X |  |  |  |  |  |  |  |  |  | 1 |
| Gottfried (125) |  |  |  |  | X |  |  |  |  |  |  |  |  |  |  |  | 1 |
| Grandahl, Rosenblad (126) |  | X |  |  |  |  |  |  |  |  |  |  |  |  |  |  | 1 |
| Grudnikoff et al. [127] | X |  |  |  |  |  |  |  |  |  |  |  |  |  |  |  | 1 |
| Guttu, Engelke (128) |  |  |  |  | X |  |  |  |  |  |  |  |  |  |  |  | 1 |
| Hackett (129) |  |  | X |  |  |  |  |  |  |  |  |  |  |  |  |  | 1 |
| Halterman, Szilagyi (130) | X |  |  |  |  |  |  |  |  |  |  |  |  |  |  |  | 1 |
| Hanson, Aleman (131) |  |  |  | X |  |  |  |  |  |  |  |  |  |  |  |  | 1 |
| Harrell, McMurray (132) |  |  |  |  |  |  |  |  |  |  | X |  |  |  |  |  | 1 |
| Harrington, Langhans (133) |  |  |  |  |  |  |  |  |  |  |  |  |  |  |  | X | 1 |
| Hawkins, Hayes (134) |  |  |  |  |  |  |  | X |  |  |  |  |  |  |  |  | 1 |
| Hawthorne, Shaibi (135) |  |  |  |  |  |  |  |  | X |  |  |  |  |  |  |  | 1 |
| Hayes‐Bohn, Neumark‐Sztainer (136) |  |  |  |  |  |  |  |  |  | X |  |  |  |  |  |  | 1 |
| Hayter, Owen (137) |  |  |  |  |  |  |  |  |  |  |  |  |  | X |  |  | 1 |
| Health Visitors Association [138] |  |  |  |  |  |  |  |  |  |  |  |  |  |  | X |  | 1 |
| Health Visitors Association [139] |  |  |  |  |  |  |  |  |  |  |  |  |  |  | X |  | 1 |
| Hellems and Clarke (140) |  |  |  |  |  |  |  |  |  | X |  |  |  |  |  |  | 1 |
| Hendershot, Dake (141) |  |  |  |  | X |  |  |  |  |  |  |  |  |  |  |  | 1 |
| Hendershot, Telljohann (142) |  |  |  |  | X |  |  |  |  |  |  |  |  |  |  |  | 1 |
| Henry (143) |  |  |  |  |  |  |  |  |  |  |  |  |  |  | X |  | 1 |
| Hill and Hollis (144) | X |  |  |  | X |  |  |  |  |  |  |  |  |  |  |  | 2 |
| Houck, Darnell (145) |  |  |  |  |  |  |  |  |  |  |  | X |  |  |  |  | 1 |
| Houghton, Egan (146) |  |  |  |  |  |  |  |  |  |  |  |  |  |  | X |  | 1 |
| Hoying and Melnyk (147) |  |  |  |  |  |  |  |  |  |  |  | X |  |  |  |  | 1 |
| Igoe (148) |  |  |  |  |  |  |  |  |  |  |  |  |  |  | X |  | 1 |
| Izquierdo, Morin (149) |  |  |  |  |  |  |  |  |  |  |  |  |  |  |  | X | 1 |
| Janevic, Stoll (150) |  |  |  | X |  |  |  |  |  |  |  |  |  |  |  |  | 1 |
| Johansson and Ehnfors (151) |  |  |  |  |  |  |  |  |  |  |  | X |  |  |  |  | 1 |
| Johnston, Moreno (152) |  |  |  |  |  |  |  |  | X |  |  |  |  |  |  |  | 1 |
| Johnston, Moreno (153) |  |  |  |  |  |  |  |  | X |  |  |  |  |  |  |  | 1 |
| Jones and McEwen (154) |  |  |  |  |  |  |  |  |  |  |  |  |  | X |  |  | 1 |
| Jordan, MacKay (155) | X |  | X |  |  |  |  |  |  |  |  |  |  |  |  |  | 2 |
| Joyner [156] |  |  | X |  |  |  |  |  |  |  |  |  |  |  |  |  | 1 |
| Kaufman and Blanchon (157) |  |  |  |  |  |  | X |  |  |  |  |  |  |  |  |  | 1 |
| Kelly, Greaves (158) |  |  |  |  |  |  |  |  |  |  |  |  |  | X |  |  | 1 |
| Kemper, Helfrich (159) | X |  |  |  | X |  |  |  |  |  |  |  |  |  |  |  | 2 |
| Khubchandani et al [160] | X |  |  |  |  |  |  |  |  |  |  |  |  |  |  |  | 1 |
| Kim, Becker (161) |  |  |  |  |  |  |  |  |  |  |  | X |  |  |  |  | 1 |
| Kimel (162) |  |  |  |  | X | X |  |  |  |  |  |  |  |  | X |  | 3 |
| Kirchofer, Telljohann (163) |  |  |  |  | X |  |  |  |  |  |  |  |  |  |  |  | 1 |
| Knauer, Baker (164) |  |  |  | X |  |  |  |  |  |  |  |  |  |  |  |  | 1 |
| Kornguth (165) |  |  |  |  |  |  |  |  |  |  |  |  |  |  | X |  | 1 |
| Krenitsky-Korn (166) | X |  |  |  |  |  |  |  |  |  |  |  |  |  |  |  | 1 |
| Kroshus [167] | X |  |  |  |  |  |  |  |  |  |  |  |  |  |  |  | 1 |
| Krug, Brener (168) |  |  |  |  |  |  |  |  |  |  | X |  |  |  |  |  | 1 |
| Lamb, Albrecht (169) |  |  |  |  |  | X |  |  |  |  |  | X |  |  |  |  | 2 |
| Land and Barclay (170) |  |  | X |  |  |  |  |  |  |  |  |  |  |  |  |  | 1 |
| Larsson and Carlsson (171) |  |  |  |  |  | X |  |  |  |  |  |  |  |  |  |  | 1 |
| Lazdowsky, Rabner (172) | X |  |  |  |  |  |  |  |  |  |  |  |  |  |  |  | 1 |
| Lee and Kubik [173] | X |  |  |  |  |  |  |  |  |  |  |  |  |  |  |  | 1 |
| Leff and Bennett (174) |  |  |  |  |  |  |  |  |  |  |  |  |  |  | X |  | 1 |
| Lehmkuhl and Nabors (175) |  |  |  |  |  |  |  |  |  | X |  |  |  |  |  |  | 1 |
| Levy, Heffner (176) |  |  |  |  |  |  | X |  |  |  |  |  |  |  |  | X | 2 |
| Lewis et al. [177] |  |  |  |  |  |  | X |  |  |  |  |  |  |  |  |  | 1 |
| Liberatos, Leone (178) |  |  |  | X |  |  |  |  |  |  |  |  |  |  |  |  | 1 |
| Lightfoot and Bines (179) |  |  |  |  |  |  |  |  |  |  |  |  |  |  | X |  | 1 |
| Lightfoot and Bines (180) |  |  |  |  |  |  |  | X |  |  |  |  |  |  |  |  | 1 |
| Lightfoot and Bines (181) |  |  | X |  |  |  |  | X |  |  |  |  |  |  |  |  | 2 |
| Lindeke [182] |  |  |  |  |  |  | X |  |  |  |  |  |  |  |  |  | 1 |
| Liptzin, Gleason (183) |  |  |  | X |  |  |  |  |  |  |  |  |  |  |  |  | 1 |
| Long, Whitman (184) |  |  |  |  |  | X |  |  |  |  |  |  |  |  |  |  | 1 |
| Looman [185] |  |  |  |  |  |  | X |  |  |  |  |  |  |  |  |  | 1 |
| Lunney (186) |  |  |  |  |  |  |  |  |  |  |  |  |  |  | X |  | 1 |
| Lunney, Cavendish (187) |  |  |  |  |  |  |  |  |  |  |  |  |  |  | X |  | 1 |
| Lunstead [188] | X |  |  |  |  |  |  |  |  |  |  |  |  |  |  |  | 1 |
| Luthy, Thorpe (189) |  | X |  |  | X |  |  |  |  |  |  |  |  |  |  |  | 2 |
| Lynch (190) |  |  |  |  |  |  |  |  |  |  |  |  |  | X |  |  | 1 |
| Magalnick [191] |  |  |  |  |  |  |  | X |  |  |  |  |  |  |  |  | 1 |
| Magee [192] | X |  |  |  |  |  |  |  |  |  |  |  |  |  |  |  | 1 |
| Major, Clarke (193) |  |  |  |  | X |  |  |  |  |  |  |  |  |  |  |  | 1 |
| Maunder (194) |  |  |  |  |  |  |  | X |  |  |  |  |  |  |  |  | 1 |
| McKaig, Hindi‐Alexander (195) |  |  |  |  |  |  |  | X |  |  |  |  |  |  |  |  | 1 |
| Medaglia, Knorr (196) | X |  |  |  |  |  |  |  |  |  |  |  |  |  |  |  | 1 |
| Melin and Lenner (197) |  |  |  |  |  |  |  |  | X |  |  |  |  |  |  |  | 1 |
| Mickel, Shanovich (198) | X |  |  |  |  |  |  |  |  |  |  |  |  |  |  |  | 1 |
| Moricca, Grasska (199) | X |  |  | X |  |  | X |  |  |  |  |  |  |  |  | X | 4 |
| Morris et al. [200] | X |  |  |  |  |  |  |  |  |  |  |  |  |  |  |  | 1 |
| Morrison-Sandberg, Kubik (201) |  |  |  |  | X |  |  |  |  |  |  |  |  |  |  |  | 1 |
| Morton and Schultz (202) |  |  |  |  | X |  |  |  |  |  |  |  |  |  |  | X | 2 |
| Muggeo, Stewart (203) | X |  |  |  |  |  |  |  |  |  |  | X |  |  |  |  | 2 |
| Murray (204) |  |  |  |  |  |  |  | X |  |  |  |  |  |  |  |  | 1 |
| Naish and Barr [205] |  |  |  |  |  |  |  |  |  |  |  |  |  |  | X |  | 1 |
| Nauta, Byrne (206) |  |  |  |  | X |  |  |  |  |  |  |  |  |  |  |  | 1 |
| Nguyen, Mason (207) |  |  |  |  |  |  |  |  |  | X |  |  |  |  |  |  | 1 |
| NHS Wales [208] |  |  |  |  |  |  |  |  |  |  |  |  |  |  | X |  | 1 |
| Noyes, Bajorska (209) |  |  |  |  |  |  |  |  |  |  |  |  |  |  |  | X | 1 |
| Nutbeam, Farley (210) |  |  |  |  |  |  |  |  |  |  |  |  |  |  | X |  | 1 |
| O`Donnell and Alles [211] |  |  |  |  | X |  |  |  |  |  |  |  |  |  |  |  | 1 |
| O'Toole, O'Toole (212) |  |  | X |  |  |  |  |  |  |  |  |  |  |  |  |  | 1 |
| Oda (213) |  |  |  |  |  |  |  |  |  |  |  |  |  |  | X |  | 1 |
| Olowokere and Okanlawon (214) |  |  |  |  |  |  |  |  |  |  |  | X |  |  |  |  | 1 |
| Paavilainen, Åstedt‐Kurki (215) |  |  | X |  |  |  |  |  |  |  |  |  |  |  |  |  | 1 |
| Paavilainen, Helminen (216) |  |  | X |  |  |  |  |  |  |  |  |  |  |  |  |  | 1 |
| Paavilainen and Tarkka (217) |  |  | X |  |  |  |  |  |  |  |  |  |  |  |  |  | 1 |
| Pakieser, Starr (218) |  |  | X |  |  |  |  |  |  |  |  |  |  |  |  |  | 1 |
| Palmore and Millar (219) |  |  |  |  |  |  |  |  |  |  |  |  |  |  | X |  | 1 |
| Pbert, Druker (220) | X |  |  |  |  |  |  |  |  |  |  |  |  |  |  |  | 1 |
| Pbert, Druker (221) | X |  |  |  |  |  |  |  | X |  |  |  |  |  |  |  | 2 |
| Peckover and Trotter (222) |  |  | X |  |  |  |  |  |  |  |  |  |  |  |  |  | 1 |
| Peery, Engelke (223) | X |  |  |  |  |  |  |  |  | X |  |  |  |  |  |  | 2 |
| Pennington and Delaney (224) |  |  |  |  |  |  |  |  |  |  |  |  |  |  |  | X | 1 |
| Perry and Toole (225) |  |  |  |  |  | X |  |  |  |  |  |  |  |  |  |  | 1 |
| Persaud, Barnett (226) |  |  |  |  |  | X |  |  |  |  | X |  |  |  |  |  | 2 |
| Petitgout, Pelzer (227) |  |  |  |  |  |  | X |  |  |  |  |  |  |  |  |  | 1 |
| Powell et al. [228] | X |  |  |  |  |  |  |  |  |  |  |  |  |  |  |  | 1 |
| Presler (229) |  |  |  |  |  |  | X |  |  |  |  |  |  |  |  |  | 1 |
| Pryjmachuk, Graham (230) |  |  |  |  |  |  |  |  |  |  |  |  |  | X |  |  | 1 |
| Pulcini, Marshall (231) | X |  |  |  |  |  |  |  |  |  |  |  |  |  |  |  | 1 |
| Putman-Casdorph and Pinto [232] | X |  |  |  |  |  |  |  |  |  |  |  |  |  |  |  | 1 |
| Quaranta and Spencer [233] | X |  |  |  |  |  |  |  |  |  |  |  |  |  |  |  | 1 |
| Quelly [234] | X |  |  |  |  |  |  |  |  |  |  |  |  |  |  |  | 1 |
| Raible, Dick (235) | X |  |  |  |  |  |  |  |  |  |  |  |  |  |  |  | 1 |
| Ramirez, Harland (236) |  |  |  |  |  |  |  |  |  |  |  | X |  |  |  |  | 1 |
| Ramos, Greenberg (237) |  |  | X |  |  |  |  |  |  |  |  |  |  |  |  |  | 1 |
| Rasberry, Morris (238) | X |  |  |  |  |  |  |  |  |  |  |  |  |  |  |  | 1 |
| Rebmann, Elliott (239) | X |  |  |  |  |  |  |  |  |  |  |  |  |  |  |  | 1 |
| Reid (240) |  |  |  |  |  |  |  |  |  |  |  |  |  |  | X |  | 1 |
| Resnicow and Allensworth (241) |  |  |  |  |  |  |  |  |  |  |  |  |  |  | X |  | 1 |
| Rhodes et al. [242] | X |  |  |  |  |  |  |  |  |  |  |  |  |  |  |  | 1 |
| Rivkina, Tapke (243) | X |  |  |  |  |  |  |  |  |  |  |  |  |  |  |  | 1 |
| Robbins, Pfeiffer (244) |  |  |  |  |  |  |  |  | X |  |  |  |  |  |  |  | 1 |
| Roden (245) |  |  |  |  |  |  |  |  |  |  |  |  |  | X |  |  | 1 |
| Rodriguez, Rivera (246) | X |  |  |  |  |  |  |  |  |  |  |  |  |  |  | X | 2 |
| Rose [247] |  |  |  |  | X |  |  |  |  |  |  |  |  |  |  |  | 1 |
| Rose (248) |  |  |  |  |  |  |  |  |  |  |  |  |  | X |  |  | 1 |
| Rosen, DiClemente (249) | X |  |  |  |  |  |  |  |  |  |  |  |  |  |  |  | 1 |
| Rosen, Goodson (250) | X |  |  |  |  |  |  |  |  |  |  |  |  |  |  |  | 1 |
| Rote (251) |  |  |  |  |  |  |  |  |  |  |  |  |  |  | X |  | 1 |
| Rote (252) |  |  |  |  |  |  |  |  |  |  |  |  |  |  | X |  | 1 |
| Rustia, Hartley (253) |  |  |  |  |  |  |  | X |  |  |  |  |  |  |  |  | 1 |
| Salend and Mahoney (254) |  |  |  |  |  |  |  |  |  |  | X |  |  |  |  |  | 1 |
| Salmon, Moulton (255) |  |  |  |  | X |  |  |  |  |  |  |  |  |  |  |  | 1 |
| Savage and Goodall (256) |  |  |  |  |  |  |  |  |  |  |  |  |  | X |  |  | 1 |
| Schaffer, Anderson (257) | X |  |  |  |  |  |  |  |  |  |  |  |  |  |  |  | 1 |
| Schols, De Ruiter (258) |  |  | X |  |  |  |  |  |  |  |  |  |  |  |  |  | 1 |
| Schonfeld (259) |  |  |  |  |  |  |  |  |  |  |  |  |  |  | X |  | 1 |
| Schroeder [260] | X |  |  |  |  |  |  |  |  |  |  |  |  |  |  |  | 1 |
| Schwartz, Denham (261) |  |  |  |  |  |  |  |  |  | X |  |  |  |  |  |  | 1 |
| Scott and Hall [262] | X |  |  |  |  |  |  |  |  |  |  |  |  |  |  |  | 1 |
| Seidenberg (263) |  |  |  |  |  |  |  | X |  |  |  |  |  |  |  |  | 1 |
| Sekhar et al. [264] | X |  |  |  |  |  |  |  |  |  |  |  |  |  |  |  | 1 |
| Sekhar, Kraschnewski (265) |  |  | X |  |  |  |  |  |  |  |  |  |  |  |  |  | 1 |
| Sherman, Alexander (266) |  |  |  |  |  |  |  |  |  |  | X |  |  |  |  |  | 1 |
| Singer [267] | X |  |  |  |  |  |  |  |  |  |  |  |  |  |  |  | 1 |
| Skelley, Luthin (268) |  |  |  |  |  |  |  |  |  | X |  |  |  |  |  |  | 1 |
| Smith and Ilardi (269) |  |  |  |  |  |  |  | X |  |  |  |  |  |  |  |  | 1 |
| Snyder, Minnick (270) |  |  |  |  | X |  |  |  |  |  |  |  |  |  |  |  | 1 |
| Speroni, Earley (271) |  |  |  |  |  |  |  |  | X |  |  |  |  |  |  |  | 1 |
| Spina, McIntyre (272) | X |  |  |  |  |  |  |  |  |  |  |  |  |  |  |  | 1 |
| Splett, Erickson (273) |  |  |  |  |  |  |  |  |  |  |  |  |  |  |  | X | 1 |
| Spratt, Philip (274) |  |  |  |  |  |  |  |  |  |  |  | X |  |  |  |  | 1 |
| Sprinks (275) |  |  |  |  |  |  |  |  |  |  |  |  |  | X |  |  | 1 |
| Squires (276) |  |  |  |  |  |  |  |  |  |  |  |  |  | X |  |  | 1 |
| Stallard, Simpson (277) |  |  |  |  |  |  |  |  |  |  |  |  |  | X |  |  | 1 |
| Stallard, Simpson (278) |  |  |  |  |  |  |  |  |  |  |  | X |  |  |  |  | 1 |
| Stalter [279] | X |  |  |  | X |  |  |  |  |  |  |  |  |  |  |  | 2 |
| Stalter [280] | X |  |  |  |  |  |  |  |  |  |  |  |  |  |  |  | 1 |
| Stang, Story (281) |  |  |  |  | X |  |  |  |  |  |  |  |  |  |  |  | 1 |
| Staudt et al. [282] | X |  |  |  |  |  |  |  |  |  |  |  |  |  |  |  | 1 |
| Stephenson (283) |  |  |  |  | X |  |  | X |  |  |  |  |  |  |  |  | 2 |
| Steele et al. [284] | X |  |  |  |  |  |  |  |  |  |  |  |  |  |  |  | 1 |
| Streeting (285) |  |  |  |  |  |  |  |  |  |  |  |  |  | X |  |  | 1 |
| Swallow and Roberts (286) | X | X |  |  |  |  |  |  |  |  |  |  |  |  |  |  | 2 |
| Szychlinski, Schmeissing (287) | X |  |  |  |  |  |  |  |  |  |  |  |  |  |  |  | 1 |
| Taras, Wright (288) |  |  |  |  |  |  | X |  |  |  |  |  |  |  |  |  | 1 |
| Taylor, Lizzi (289) |  |  |  |  |  |  | X |  |  |  |  |  |  |  |  |  | 1 |
| Telljohann, Dake (290) |  |  |  |  | X |  |  |  |  |  |  |  |  |  |  | X | 2 |
| Terry, Patel (291) | X |  |  |  |  |  |  |  |  |  |  |  |  |  |  |  | 1 |
| Thompson (292) |  |  |  |  |  |  |  | X |  |  |  |  |  |  |  |  | 1 |
| Thomson (293) |  |  |  |  |  |  |  |  |  |  |  |  |  | X |  |  | 1 |
| Thurston and Walker (294) |  |  |  |  |  |  |  |  |  |  |  |  |  | X |  |  | 1 |
| Toole and Perry (295) |  | X |  |  |  |  |  |  |  |  |  |  |  |  |  |  | 1 |
| Triggle (296) |  |  |  |  |  |  |  |  |  |  |  |  |  | X |  |  | 1 |
| Trivedi, Patel (297) |  |  |  |  |  |  |  |  |  |  |  |  |  |  |  | X | 1 |
| Tsacoyianis (298) |  |  |  |  |  |  |  |  |  |  | X |  |  |  |  |  | 1 |
| Tucker and Lanningham-Foster (299) |  |  |  |  |  |  |  |  | X |  |  |  |  |  |  |  | 1 |
| Turner, Shield (300) |  |  |  |  |  |  |  |  |  |  |  |  |  | X |  |  | 1 |
| Urbinati, Steele (301) |  |  |  |  |  |  |  | X |  |  |  |  |  |  |  |  | 1 |
| Van Cura (302) |  |  |  |  |  |  |  |  |  |  |  |  |  |  |  | X | 1 |
| Van Roeyen (303) |  |  |  |  |  |  | X |  |  |  |  |  |  |  |  |  | 1 |
| Vanderpool, Breheny (304) |  | X |  |  |  |  |  |  |  |  |  |  |  |  |  |  | 1 |
| Vernberg, Nelson (305) |  |  |  |  | X |  |  |  |  |  |  |  |  |  |  |  | 1 |
| Vernon, Conner (306) |  | X |  |  |  |  |  |  |  |  |  |  |  |  |  |  | 1 |
| Vessey and O’Neill (307) | X |  |  |  |  |  |  |  |  |  |  | X |  |  |  |  | 2 |
| Wall (308) |  |  |  |  |  |  |  |  |  |  |  | X |  |  |  |  | 1 |
| Watson (309) |  |  |  |  |  |  |  | X |  |  |  |  |  |  |  |  | 1 |
| Weismuller, Grasska (310) |  |  |  |  | X |  |  | X |  |  |  |  |  |  |  |  | 2 |
| Wells [311] |  |  |  |  |  |  | X |  |  |  |  |  |  |  |  |  | 1 |
| Welsh Office [312] |  |  |  |  |  |  |  |  |  |  |  |  |  |  | X |  | 1 |
| Welsh Office [313] |  |  |  |  |  |  |  |  |  |  |  |  |  |  | X |  | 1 |
| Werch, Carlson (314) |  |  |  |  |  | X |  |  |  |  |  |  |  |  |  |  | 1 |
| White et al. [315] | X |  |  |  |  |  |  |  |  |  |  |  |  |  |  |  | 1 |
| Whitfield (316) |  |  |  |  |  |  |  |  |  |  |  |  |  |  | X |  | 1 |
| Whitmarsh (317) |  |  |  |  |  |  |  |  |  |  |  |  |  |  | X |  | 1 |
| Whitmore (318) |  |  |  |  |  |  |  |  |  |  |  |  |  |  | X |  | 1 |
| Wicklander (319) |  |  |  |  |  |  |  | X |  |  |  |  |  |  |  |  | 1 |
| Wiggs-Stayner, Purdy (320) |  |  |  |  | X |  |  |  |  |  |  |  |  |  |  | X | 2 |
| Williams and Warrington (321) |  |  |  |  |  |  |  |  | X |  |  |  |  |  |  |  | 1 |
| Wilson et al. [322] | X |  |  |  |  |  |  |  |  |  |  |  |  |  |  |  | 1 |
| Wing et al. [323] | X |  |  |  |  |  |  |  |  |  |  |  |  |  |  |  | 1 |
| Winkelstein (324) |  |  |  |  |  |  |  |  |  |  | X |  |  |  |  |  | 1 |
| Wong and Cheng (325) |  |  |  |  |  |  |  |  | X |  |  |  |  |  |  |  | 1 |
| Wright, Giger (326) |  |  |  |  |  |  |  |  | X |  |  |  |  |  |  |  | 1 |

Reference list:

1. Best NC, Oppewal S, Travers D. Exploring School Nurse Interventions and Health and Education Outcomes: An Integrative Review. J Sch Nurs. 2018;34(1):14-27. doi: https://dx.doi.org/10.1177/1059840517745359. PubMed PMID: 29207914.

2. Guarinoni M, Dignani L. Effectiveness of the school nurse role in increasing the vaccination coverage rate: a narrative review. Ann Ig. 2021;33(1):55-66.

3. Harding L, Davison-Fischer J, Bekaert S, Appleton JV. The role of the school nurse in protecting children and young people from maltreatment: An integrative review of the literature. International journal of nursing studies. 2019;92:60-72.

4. Isik E, Isik IS. Asthma care coordination in schools by school nurses: An integrative literature review. Public Health Nurs. 2019;36(4):498-506. doi: https://dx.doi.org/10.1111/phn.12610. PubMed PMID: 30968440.

5. Lineberry MJ, Ickes MJ. The role and impact of nurses in American elementary schools: A systematic review of the research. The Journal of School Nursing. 2014;31(1):22-33.

6. Maughan E. The impact of school nursing on school performance: a research synthesis. J Sch Nurs. 2003;19(3):163-71. PubMed PMID: 12755681.

7. McClanahan R, Weismuller PC. School nurses and care coordination for children with complex needs: an integrative review. J Sch Nurs. 2014;31(1):34-43. doi: https://dx.doi.org/10.1177/1059840514550484. PubMed PMID: 25266887.

8. Schmitt S, Görres S. [School nursing in Germany? - A review about tasks and roles of School Nurses]. Pflege. 2012;25(2):107-17. Epub 2012/04/05. doi: 10.1024/1012-5302/a000186. PubMed PMID: 22473734.

9. Schroeder K, Travers J, Smaldone A. Are School Nurses an Overlooked Resource in Reducing Childhood Obesity? A Systematic Review and Meta-Analysis. J Sch Health. 2016;86(5):309-21. doi: https://dx.doi.org/10.1111/josh.12386. PubMed PMID: 27040469.

10. Stefanowicz A, Stefanowicz J. The role of a school nurse in the care of a child with diabetes mellitus type 1-the perspectives of patients and their parents: Literature review. Slovenian Journal of Public Health. 2018;57(3):166.

11. Stock JL, Larter N, Kieckehefer GM, Thronson G, Maire J. Measuring outcomes of school nursing services. J Sch Nurs. 2002;18(6):353-9. PubMed PMID: 12463773.

12. Tanner A, Miller WR, Gaudecker Jv, Buelow JM. An integrative review of school-based mental health interventions and implications for psychogenic nonepileptic seizures. The Journal of School Nursing. 2020;36(1):33-48.

13. Tilley S, Chambers M. Research in brief. A systematic review on the effectiveness of school nurse implemented mental health screening available for adolescents in schools. Journal of Psychiatric & Mental Health Nursing (Wiley-Blackwell). 2003;10(5):625-6. PubMed PMID: 106732880. Language: English. Entry Date: 20050425. Revision Date: 20150711. Publication Type: Journal Article.

14. Turner G, Mackay S. The impact of school nurse interventions: Behaviour change and mental health. British Journal of School Nursing. 2015;10(10):494-506. doi: 10.12968/bjsn.2015.10.10.494. PubMed PMID: 111952980. Language: English. Entry Date: 20151230. Revision Date: 20151231. Publication Type: Article.

15. Wainwright P, Thomas J, Jones M. Health promotion and the role of the school nurse: a systematic review. Journal of Advanced Nursing. 2000;32(5):1083-91.

16. Yoder CM. School Nurses and Student Academic Outcomes: An Integrative Review. J Sch Nurs. 2020;36(1):49-60. doi: https://dx.doi.org/10.1177/1059840518824397. PubMed PMID: 30674219.

17. Initiating health programmes in schools and communities. Int Nurs Rev. 1997;44(3):76-8. Epub 1997/05/01. PubMed PMID: 9195252.

18. Adams C. Perceptions of the comprehensive-based school nurse. Health visitor. 1990;63(3):90-2.

19. Alizadeh V, Törnkvist L, Hylander I. Counselling teenage girls on problems related to the ‘protection of family honour’from the perspective of school nurses and counsellors. Health & social care in the community. 2011;19(5):476-84.

20. Allen G. The impact of elementary school nurses on student attendance. J Sch Nurs. 2003;19(4):225-31. Epub 2003/07/29. doi: 10.1177/10598405030190040801. PubMed PMID: 12882606.

21. Allen K, Henselman K, Laird B, Quiñones A, Reutzel T. Potential life-threatening events in schools involving rescue inhalers, epinephrine autoinjectors, and glucagon delivery devices: Reports from school nurses. The Journal of School Nursing. 2012;28(1):47-55.

22. Allensworth DD, Bradley B. Guidelines for adolescent preventive services: a role for the school nurse. Journal of school health. 1996;66(8):281-5.

23. Allison VL, Nativio DG, Mitchell AM, Ren D, Yuhasz J. Identifying symptoms of depression and anxiety in students in the school setting. The Journal of School Nursing. 2014;30(3):165-72.

24. Amillategui B, Calle J, Alvarez M, Cardiel M, Barrio R. Identifying the special needs of children with Type 1 diabetes in the school setting. An overview of parents’ perceptions. Diabetic Medicine. 2007;24(10):1073-9.

25. Anderson J. The changing role of school nurses--one state's experience. The Journal of school nursing: the official publication of the National Association of School Nurses. 1994;10(3):22-6.

26. Antonelli RC, Antonelli DM. Providing a medical home: the cost of care coordination services in a community-based, general pediatric practice. Pediatrics. 2004;113(5 Suppl):1522-8. Epub 2004/05/04. PubMed PMID: 15121921.

27. Antonelli RC, McAllister JW, Popp J. Making care coordination a critical component of the pediatric health system: a multidisciplinary framework. 2009.

28. Antonelli RC, Stille CJ, Antonelli DM. Care coordination for children and youth with special health care needs: a descriptive, multisite study of activities, personnel costs, and outcomes. Pediatrics. 2008;122(1):e209-16. Epub 2008/07/04. doi: 10.1542/peds.2007-2254. PubMed PMID: 18595966.

29. Anyanwu I. The face of diversity. Challenges in school health. School nurse news. 2005;22(1):27-.

30. Aruda MM, Kelly M, Newinsky K. Unmet needs of children with special health care needs in a specialized day school setting. The Journal of School Nursing. 2011;27(3):209-18.

31. Atherton C. On hand, in school. Community Pract. 2009;82(7):32-3. Epub 2009/07/25. PubMed PMID: 19626752.

32. Attwood M, Meadows S, Stallard P, Richardson T. Universal and targeted computerised cognitive behavioural therapy (Think, Feel, Do) for emotional health in schools: results from two exploratory studies. Child Adolesc Ment Health. 2012;17(3):173-8. Epub 2012/09/01. doi: 10.1111/j.1475-3588.2011.00627.x. PubMed PMID: 32847273.

33. Badger F, Brown I. Primary schools' use and perceptions of the school nursing service. International Journal of Health Promotion and Education. 2005;43(3):92-6.

34. Bagnall P. School nurses' response to the measles vaccination campaign. Nursing times. 1995;91(40):38-9.

35. Bagnall P. Children's health: taking it seriously. MA Healthcare London; 1997.

36. Baisch MJ, Lundeen SP, Murphy MK. Evidence‐based research on the value of school nurses in an urban school system. Journal of School Health. 2011;81(2):74-80.

37. Baker DL, Hebbeler K, Davis-Alldritt L, Anderson LS, Knauer H. School health services for children with special health care needs in California. The Journal of School Nursing. 2015;31(5):318-25.

38. Baldwin C. Changing health outcomes for African American children: Utilizing a self-care health promotion Curriculum in urban elementary schools. JOURNAL OF MULTICULTURAL NURSING AND HEALTH. 1998;4:40-5.

39. Bannink R, Broeren S, Joosten-van Zwanenburg E, van As E, van de Looij-Jansen P, Raat H. Effectiveness of a web-based tailored intervention (E-health4Uth) and consultation to promote adolescents’ health: randomized controlled trial. Journal of medical Internet research. 2014;16(5):e143.

40. Barnard-Brak L, Stevens T, Carpenter J. Care Coordination with Schools: The Role of Family-Centered Care for Children with Special Health Care Needs. Matern Child Health J. 2017;21(5):1073-8. doi: <https://dx.doi.org/10.1007/s10995-016-2203-x>. PubMed PMID: 28144766.

41. Barrett JC. A school-based care management service for children with special needs. Family & Community Health. 2000;23(2):36-42.

42. Bartfay WJ, Bartfay E. Promoting health in schools through a board game. Western journal of nursing research. 1994;16(4):438-46.

43. Bednarz P. The Omaha System: a model for describing school nurse case management. The Journal of school nursing: the official publication of the National Association of School Nurses. 1998;14(3):24-30.

44. Bergren M, Mehl R. Health software for school nurses. The Journal of school nursing: the official publication of the National Association of School Nurses. 1995;11(3):6-7.

45. Bergren M. School nurse politics on the web. The Journal of school nursing: the official publication of the National Association of School Nurses. 1996;12(3):39-40.

46. Bergren M, Murphy E. The best of the web for school health. The Journal of school nursing: the official publication of the National Association of School Nurses. 1997;13(5):36-7.

47. Bergren MD. The feasibility of collecting school nurse data. The Journal of School Nursing. 2016;32(5):337-46.

48. Betz CL, Redcay G. Dimensions of the transition service coordinator role. Journal for Specialists in Pediatric Nursing. 2005;10(2):49-59.

49. Bhardwa S. Mental health in young people. Independent Nurse. 2013;6.

50. Blaakman SW, Cohen A, Fagnano M, Halterman JS. Asthma medication adherence among urban teens: a qualitative analysis of barriers, facilitators and experiences with school-based care. Journal of Asthma. 2014;51(5):522-9.

51. Blackwell LS, Robinson AF, Proctor MR, Taylor AM. Same care, different populations: return-to-learn practices following concussion in primary and secondary schools. Journal of child neurology. 2017;32(3):327-33.

52. Bolton P. School entry screening by the school nurse. Health visitor. 1994;67(4):135-6.

53. Bonaiuto M. School nurses' competence in caring for students who depend on medical technology. The Journal of school nursing: the official publication of the National Association of School Nurses. 1995;11(4):21-2, 4.

54. Bonaiuto MM. School nurse case management: Achieving health and educational outcomes. The Journal of School Nursing. 2007;23(4):202-9.

55. Bonny AE, Britto MT, Klostermann BK, Hornung RW, Slap GB. School disconnectedness: Identifying adolescents at risk. Pediatrics. 2000;106(5):1017-21.

56. Bonsergent E, Agrinier N, Thilly N, Tessier S, Legrand K, Lecomte E, et al. Overweight and obesity prevention for adolescents: a cluster randomized controlled trial in a school setting. Am J Prev Med. 2013;44(1):30-9. Epub 2012/12/21. doi: 10.1016/j.amepre.2012.09.055. PubMed PMID: 23253647.

57. Bonsergent E, Thilly N, Legrand K, Agrinier N, Tessier S, Lecomte E, et al. Process evaluation of a school-based overweight and obesity screening strategy in adolescents. Glob Health Promot. 2013;20(2 Suppl):76-82. Epub 2013/05/25. doi: 10.1177/1757975913483330. PubMed PMID: 23678500.

58. Borawski EA, Tufts KA, Trapl ES, Hayman LL, Yoder LD, Lovegreen LD. Effectiveness of health education teachers and school nurses teaching sexually transmitted infections/human immunodeficiency virus prevention knowledge and skills in high school. Journal of School Health. 2015;85(3):189-96.

59. Boyer‐Chuanroong L, Deaver P. Meeting the preteen vaccine law: a pilot program in urban middle schools. Journal of School Health. 2000;70(2):39-44.

60. Bradley BJ. The school nurse as health educator. Journal of School Health. 1997;67(1):3-8.

61. Bradley BJ. Establishing a research agenda for school nursing. Journal of School Health. 1998;68(2):53-61.

62. Brindis CD, Sanghvi R, Melinkovich P, Kaplan DW, Ahlstrand KR, MPH SLP. Redesigning a school health workforce for a new health care environment: Training school nurses as nurse practitioners. Journal of school health. 1998;68(5):179-83.

63. Collis W, Graham S, Wyllie W. British Paediatric Association.

64. Brosnan C. Long-term results of an elementary sexuality program. Pediatric nursing. 1987;13(2):130-1.

65. Brother N. School nursing and student assistance: a natural partnership. The Journal of school nursing: the official publication of the National Association of School Nurses. 1998;14(1):32-5.

66. Broussard L. Empowerment in school nursing practice: A grounded theory approach. The Journal of School Nursing. 2007;23(6):322-8.

67. Brustrom J, Thibadeau J, John L, Liesmann J, Rose S. Care coordination in the spina bifida clinic setting: current practice and future directions. Journal of Pediatric Health Care. 2012;26(1):16-26.

68. Bruzzese JM, Evans D, Wiesemann S, Pinkett-Heller M, Levison MJ, Du Y, et al. Using school staff to establish a preventive network of care to improve elementary school students' control of asthma. J Sch Health. 2006;76(6):307-12. Epub 2006/08/22. doi: 10.1111/j.1746-1561.2006.00118.x. PubMed PMID: 16918861.

69. Bryan DS, Cook TS. Redirection of school nursing services in culturally deprived neighborhoods. American Journal of Public Health and the Nations Health. 1967;57(7):1164-76.

70. Bucher L, Dryer C, Hendrix E, Wong N. Statewide assessment of school‐age children with asthma in Delaware. Journal of school health. 1998;68(7):276-81.

71. Buckland L, Rose J, Greaves C. New roles for school nurses: Preventing exclusion. Community Practitioner. 2005;78(1):16.

72. Butler R. 'School Health Matters': a web-based health resource for the school community. Community Practitioner. 2013;86(10):30-2. PubMed PMID: 102831946. Language: English. Entry Date: 20150602. Revision Date: 20150602. Publication Type: Article.

73. Cady RG, Kelly AM, Finkelstein SM, Looman WS, Garwick AW. Attributes of advanced practice registered nurse care coordination for children with medical complexity. J Pediatr Health Care. 2014;28(4):305-12. Epub 2013/08/31. doi: 10.1016/j.pedhc.2013.06.005. PubMed PMID: 23988611; PubMed Central PMCID: PMCPMC3935987.

74. Cameron R, Brown KS, Best JA, Pelkman CL, Madill CL, Manske SR, et al. Effectiveness of a social influences smoking prevention program as a function of provider type, training method, and school risk. American Journal of Public Health. 1999;89(12):1827-31.

75. Carpenter LM, Lachance L, Wilkin M, Clark NM. Sustaining school‐based asthma interventions through policy and practice change. Journal of school health. 2013;83(12):859-66.

76. Carter B. Children's health care: a new era or more rhetoric? Journal of Child Health Care. 1997;1(4):161-2.

77. Chally PS. An eating disorders prevention program. Journal of Child and Adolescent Psychiatric Nursing. 1998;11(2):51-60.

78. Chase E, Chalmers H, Thomas F, Hollingworth K, Aggleton P. Shifting policies and enduring themes in school nursing. British Journal of School Nursing. 2010;5(10):492-500.

79. Chen SPC, Fitzgerald MC, DeStefano LM, Chen EH. Effects of a school nurse prenatal counseling program. Public Health Nursing. 1991;8(4):212-8.

80. Chilvers J. Implementation of a Facebook page by school nurses. Community Practitioner. 2011;84(4):33-5. PubMed PMID: 104851193. Language: English. Entry Date: 20110406. Revision Date: 20150820. Publication Type: Journal Article. Journal Subset: Blind Peer Reviewed.

81. Chokshi NY, Patel D, Davis CM. Long-term increase in epinephrine availability associated with school nurse training in food allergy. The Journal of Allergy and Clinical Immunology: In Practice. 2015;3(1):128-30.

82. Christiansen SC, Martin SB, Schleicher NC, Koziol JA, Mathews KP, Zuraw BL. Evaluation of a school-based asthma education program for inner-city children. Journal of Allergy and Clinical Immunology. 1997;100(5):613-7.

83. Cicutto L, To T, Murphy S. A randomized controlled trial of a public health nurse‐delivered asthma program to elementary schools. Journal of School Health. 2013;83(12):876-84.

84. Clapp A. Reducing the rate of teenage pregnancy. Practice Nurse. 2009;37(2):32-5. PubMed PMID: 105450880. Language: English. Entry Date: 20090327. Revision Date: 20171230. Publication Type: Journal Article.

85. Clarke ML. Out of the wilderness and into the fold: the school nurse and child protection. Child Abuse Review: Journal of the British Association for the Study and Prevention of Child Abuse and Neglect. 2000;9(5):364-74.

86. Clausson E, Berg A. Family intervention sessions: one useful way to improve schoolchildren's mental health. Journal of family nursing. 2008;14(3):289-313.

87. Coates M. School nursing, a priority for child-centred public health. British Journal of School Nursing. 2011;6(9):439-43.

88. Cohen J, Reddington C, Jacobs D, Meade R, Picard D, Singleton K, et al. School-related issues among HIV-infected children. Pediatrics. 1997;100(1):e8-e.

89. Coleman J, Hawkins W. The changing role of the nurse: an alternative to elimination. The Journal of school health. 1970;40(3):121-2.

90. Costante C. Supporting Student Success: School Nurses Make a Difference. NASN President. The Journal of school nursing: the official publication of the National Association of School Nurses. 1996;12(3):4-6.

91. Cox E, Fritz K, Hansen K, Brown R, Rajamanickam V, Wiles K, et al. Development and Validation of PRISM: A Survey Tool to Identify Diabetes Self-Management Barriers.(Problem Recognition in Illness Self-Management) Copyright: Elsevier Ireland Ltd. 2014.

92. Crickmore K, Jones A, Engelke MK, Mott JA. Managing pediatric asthma. Health Forum J. 2002;45(6):24-30. Epub 2003/01/01. PubMed PMID: 12506792.

93. Davies A. Enabling young people to talk about health and wellbeing. British Journal of School Nursing. 2012;7(10):512-3. doi: 10.12968/bjsn.2012.7.10.512. PubMed PMID: 108023277. Language: English. Entry Date: 20130204. Revision Date: 20200708. Publication Type: Journal Article.

94. Davis C. A lesson in quitting. Nursing Standard. 2007;21(29):18-9. PubMed PMID: 106291093. Language: English. Entry Date: 20070525. Revision Date: 20150711. Publication Type: Journal Article.

95. Davis WS, Varni SE, Barry SE, Frankowski BL, Harder VS. Increasing immunization compliance by reducing provisional admittance. The Journal of School Nursing. 2016;32(4):246-57.

96. DeSocio J, Hootman J. Children's Mental Health and School Success. 2004;20(4):189-96.

97. Diao W, Patel J, Snitzer M, Pond M, Rabinowitz MP, Ceron G, et al. The effectiveness of a mobile clinic in improving follow-up eye care for at-risk children. Journal of Pediatric Ophthalmology & Strabismus. 2016;53(6):344-8.

98. Dixon V. Teenage pregnancy: Identifying young people aspiring or ambivalent to parenthood. British Journal of School Nursing. 2014;9(1):38-44.

99. Dodds E. Are sexual health services in schools adequate? British Journal of School Nursing. 2011;6(8):397-403.

100. Doggett M-A, Faulkner A, Farrow S, Shelley A. School nurses: constraints and opportunities for the future. Journal of the Royal Society of Health. 1992;112(2):84-7.

101. DoH. Saving Lives: Our Healthier Nation. The Stationery Office London; 1999.

102. Downie J, Chapman R, Orb A, Juliff D. The everyday realities of the multi-dimensional role of the high school community nurse. Australian Journal of Advanced Nursing, The. 2002;19(3):15-24.

103. Driscoll KA, Volkening LK, Haro H, Ocean G, Wang Y, Jackson CC, et al. Are children with type 1 diabetes safe at school? Examining parent perceptions. Pediatric Diabetes. 2015;16(8):613-20.

104. Eisbach SS, Driessnack M. Am I sure I want to go down this road? Hesitations in the reporting of child maltreatment by nurses. Journal for specialists in pediatric nursing. 2010;15(4):317-23.

105. Engelke MK, Guttu M, Warren MB. Defining, delivering, and documenting the outcomes of case management by school nurses. J Sch Nurs. 2009;25(6):417-26. Epub 2009/09/25. doi: 10.1177/1059840509347377. PubMed PMID: 19776226.

106. Engelke MK, Guttu M, Warren MB, Swanson M. School nurse case management for children with chronic illness: Health, academic, and quality of life outcomes. The Journal of School Nursing. 2008;24(4):205-14.

107. Engelke MK, Swanson M, Guttu M. Process and outcomes of school nurse case management for students with asthma. The Journal of School Nursing. 2014;30(3):196-205.

108. Engelke MK, Swanson M, Guttu M, Warren MB, Lovern S. School nurses and children with diabetes: A descriptive study. North Carolina Medical Journal. 2011;72(5):351-8.

109. Engh LK, Rahm G, Eriksson U-B. School nurses avoid addressing child sexual abuse. The Journal of school nursing. 2017;33(2):133-42.

110. Engh LK, Eriksson U-B. The school nurse’s ability to detect and support abused children: A trust-creating process. The Journal of School Nursing. 2015;31(5):353-62.

111. Fagan R. Health of the Nation targets: where school nurses find constraints on achievement. Nursing standard (Royal College of Nursing (Great Britain): 1987). 1995;9(48):36-40.

112. Ferson M, Fitzsimmons G, Christie D, Woollett H. School health nurse interventions to increaseimmunisation uptake in school entrants. Public Health. 1995;109(1):25-9.

113. Few C. Alliances in school sex education: teachers and school nurses' views. Health visitor. 1996;69(6):220-3.

114. Foster LS, Keele R. Implementing an over-the-counter medication administration policy in an elementary school. The Journal of school nursing. 2006;22(2):108-13.

115. Fox P, Cowell J, Montgomery A, Willgerodt M. Southeast Asian refugee women and depression: a nursing intervention. The international journal of psychiatric nursing research. 1998;4(1):423-32.

116. Fox PG, Rossetti J, Burns KR, Popovich J. Southeast Asian refugee children: a school-based mental health intervention. The international journal of psychiatric nursing research. 2005;11(1):1227-36.

117. France J. New texting service for teenagers has all-round benefits. Nursing Standard. 2013;28(5):13.

118. Francisco B, Rood T, Nevel R, Foreman P, Homan S. Peer reviewed: Teaming up for asthma control: EPR-3 compliant school program in Missouri is effective and cost-efficient. Preventing chronic disease. 2017;14.

119. Fryer Jr G, Igoe J. A relationship between availability of school nurses and child well-being. The Journal of school nursing: the official publication of the National Association of School Nurses. 1995;11(3):12-8.

120. Fryer Jr GE, Igoe JB. Functions of school nurses and health assistants in US school health programs. Journal of School Health. 1996;66(2):55-8.

121. Gaffrey E, Bergren M. School health services and managed care: a unique partnership for child health. The Journal of school nursing: the official publication of the National Association of School Nurses. 1998;14(4):5-12, 4.

122. Garwick AW, Svavarsdóttir EK, Seppelt AM, Looman WS, Anderson LS, Örlygsdóttir B. Development of an international school nurse asthma care coordination model. Journal of advanced nursing. 2015;71(3):535-46.

123. Gilman S, Williamson MC, Nader PR, Dale S, McKevitt R. Task differentiation among elementary, middle and high school nurses. Journal of School Health. 1979;49(6):313-6.

124. Gordon JB, Colby HH, Bartelt T, Jablonski D, Krauthoefer ML, Havens P. A tertiary care-primary care partnership model for medically complex and fragile children and youth with special health care needs. Arch Pediatr Adolesc Med. 2007;161(10):937-44. Epub 2007/10/03. doi: 10.1001/archpedi.161.10.937. PubMed PMID: 17909136.

125. Gottfried MA. Understanding the institutional-level factors of urban school quality. Teachers College Record. 2012;114(12):1-32.

126. Grandahl M, Rosenblad A, Stenhammar C, Tydén T, Westerling R, Larsson M, et al. School-based intervention for the prevention of HPV among adolescents: a cluster randomised controlled study. BMJ open. 2016;6(1).

127. Grudnikoff E, Taneli T, Correll CU. Characteristics and disposition of youth referred from schools for emergency psychiatric evaluation. European child & adolescent psychiatry. 2015;24(7):731-43.

128. Guttu M, Engelke MK, Swanson M. Does the school nurse‐to‐student ratio make a difference? Journal of School Health. 2004;74(1):6-9.

129. Hackett AJ. The role of the school nurse in child protection. Community Practitioner. 2013;86(12).

130. Halterman JS, Szilagyi PG, Fisher SG, Fagnano M, Tremblay P, Conn KM, et al. Randomized controlled trial to improve care for urban children with asthma: results of the School-Based Asthma Therapy trial. Archives of pediatrics & adolescent medicine. 2011;165(3):262-8.

131. Hanson TK, Aleman M, Hart L, Yawn B. Increasing availability to and ascertaining value of asthma action plans in schools through use of technology and community collaboration. Journal of School Health. 2013;83(12):915-20.

132. Harrell JS, McMurray RG, Gansky SA, Bangdiwala SI, Bradley CB. A public health vs a risk-based intervention to improve cardiovascular health in elementary school children: the Cardiovascular Health in Children Study. American journal of public health. 1999;89(10):1529-35.

133. Harrington CB, Langhans E, Shelef DQ, Savitz M, Whitmore C, Teach SJ. A pilot randomized trial of school-based administration of inhaled corticosteroids for at-risk children with asthma. J Asthma. 2018;55(2):145-51. Epub 2017/06/09. doi: 10.1080/02770903.2017.1323915. PubMed PMID: 28594249.

134. Hawkins JW, Hayes ER, Corliss CP. School Nursing in America—1902‐1994: A Return to Public Health Nursing. Public Health Nursing. 1994;11(6):416-25.

135. Hawthorne A, Shaibi G, Gance-Cleveland B, McFall S. Grand Canyon Trekkers: school-based lunchtime walking program. The Journal of School Nursing. 2011;27(1):43-50.

136. Hayes‐Bohn R, Neumark‐Sztainer D, Mellin A, Patterson J. Adolescent and parent assessments of diabetes mellitus management at school. Journal of School Health. 2004;74(5):166-9.

137. Hayter M, Owen J, Cooke J. Developing and establishing school-based sexual health services: issues for school nursing practice. The Journal of School Nursing. 2012;28(6):433-41.

138. Association HV. Profiling School Health. London, HVA. 1991.

139. BARRIBALL KL, MACKENZIE A. The demand for measuring the impact of nursing interventions: a community perspective. Journal of Clinical Nursing. 1992;1(4):207-12.

140. Hellems MA, Clarke WL. Safe at school: a Virginia experience. Diabetes Care. 2007;30(6):1396-8.

141. Hendershot C, Dake JA, Price JH, Lartey GK. Elementary school nurses’ perceptions of student bullying. The Journal of School Nursing. 2006;22(4):229-36.

142. Hendershot C, Telljohann SK, Price JH, Dake JA, Mosca NW. Elementary school nurses’ perceptions and practices regarding body mass index measurement in school children. The Journal of School Nursing. 2008;24(5):298-309.

143. Henry S. A nursing informatics approach for addressing national issues and priorities for school nursing services. The Journal of school nursing: the official publication of the National Association of School Nurses. 1997;13(4):39-42.

144. Hill NJ, Hollis M. Teacher time spent on student health issues and school nurse presence. The Journal of School Nursing. 2012;28(3):181-6.

145. Houck GM, Darnell S, Lussman S. A support group intervention for at-risk female high school students. The Journal of School Nursing. 2002;18(4):212-8.

146. Houghton A, Egan S, Archinal G, Bradley O, Azam N. Selective medical examination at school entry: should we do it, and if so how? Journal of Public Health. 1992;14(2):111-6.

147. Hoying J, Melnyk BM. COPE: a pilot study with urban-dwelling minority sixth-grade youth to improve physical activity and mental health outcomes. The Journal of School Nursing. 2016;32(5):347-56.

148. Igoe JB. School nursing. Nurs Clin North Am. 1994;29(3):443-58. Epub 1994/09/01. PubMed PMID: 8090640.

149. Izquierdo R, Morin PC, Bratt K, Moreau Z, Meyer S, Ploutz-Snyder R, et al. School-centered telemedicine for children with type 1 diabetes mellitus. J Pediatr. 2009;155(3):374-9. Epub 2009/05/26. doi: 10.1016/j.jpeds.2009.03.014. PubMed PMID: 19464030.

150. Janevic MR, Stoll S, Wilkin M, Song PX, Baptist A, Lara M, et al. Pediatric asthma care coordination in underserved communities: a quasiexperimental study. American journal of public health. 2016;106(11):2012-8.

151. Johansson A, Ehnfors M. Mental health-promoting dialogue of school nurses from the perspective of adolescent pupils. Vård i norden. 2006;26(4):10-9.

152. Johnston CA, Moreno JP, El‐Mubasher A, Gallagher M, Tyler C, Woehler D. Impact of a school‐based pediatric obesity prevention program facilitated by health professionals. Journal of School Health. 2013a;83(3):171-81.

153. Johnston CA, Moreno JP, Gallagher MR, Wang J, Papaioannou MA, Tyler C, et al. Achieving long-term weight maintenance in Mexican-American adolescents with a school-based intervention. Journal of Adolescent Health. 2013b;53(3):335-41.

154. Jones L, McEwen A. Reducing secondhand smoke exposure at home. British Journal of School Nursing. 2012;7(8):389-93.

155. Jordan KS, MacKay P, Woods SJ. Child maltreatment: Optimizing recognition and reporting by school nurses. NASN school nurse. 2017;32(3):192-9.

156. Joyner. What are school health nurses lived experiences of working with children and their families who are subject to a child protection plan? 2012.

157. Kaufman J, Blanchon D. Managed care for children with special needs: A care coordination model. JOURNAL OF CARE MANAGEMENT-WESTPORT-. 1996;2:46-59.

158. Kelly N, Greaves C, Buckland L, Rose J. School nurses: well placed to address challenging behaviour. Community Practitioner. 2005;78(3):88.

159. Kemper AR, Helfrich A, Talbot J, Patel N. Outcomes of an elementary school-based vision screening program in North Carolina. The Journal of School Nursing. 2012;28(1):24-30.

160. Khubchandani J, Telljohann SK, Price JH, Dake JA, Hendershot C. Providing assistance to the victims of adolescent dating violence: A national assessment of school nurses' practices. Journal of school health. 2013;83(2):127-36.

161. Kim RE, Becker KD, Stephan SH, Hakimian S, Apocada D, Escudero PV, et al. Connecting students to mental health care: Pilot findings from an engagement program for school nurses. Advances in school mental health promotion. 2015;8(2):87-103.

162. Kimel L. Handwashing education can decrease illness absenteeism. The Journal of school nursing: the official publication of the National Association of School Nurses. 1996;12(2):14-6, 8.

163. Kirchofer G, Telljohann SK, Price JH, Dake JA, Ritchie M. Elementary school parents’/guardians’ perceptions of school health service personnel and the services they provide. Journal of School Health. 2007;77(9):607-14.

164. Knauer H, Baker DL, Hebbeler K, Davis-Alldritt L. The mismatch between children’s health needs and school resources. The Journal of School Nursing. 2015;31(5):326-33.

165. Kornguth ML. Preventing school absences due to illness. Journal of School Health. 1991;61(6):272-5.

166. Krenitsky-Korn S. High school students with asthma: attitudes about school health, absenteeism, and its impact on academic achievement. Pediatric nursing. 2011;37(2).

167. Kroshus E, Fischer AN, Nichols JF. Assessing the awareness and behaviors of US high school nurses with respect to the female athlete triad. The Journal of School Nursing. 2015;31(4):272-9.

168. Krug EG, Brener ND, Dahlberg LL, Ryan GW, Powell KE. The impact of an elementary school-based violence prevention program on visits to the school nurse. American journal of preventive medicine. 1997;13(6):459-63.

169. Lamb J, Albrecht S, Sereika S. Consideration of factors prior to implementing a smoking cessation program. The Journal of school nursing: the official publication of the National Association of School Nurses. 1998;14(1):14-9.

170. Land M, Barclay L. Nurses' contribution to child protection. Neonatal, paediatric and child health nursing. 2008;11(1):18-24.

171. Larsson B, Carlsson J. A school-based, nurse-administered relaxation training for children with chronic tension-type headache. Journal of Pediatric Psychology. 1996;21(5):603-14.

172. Lazdowsky L, Rabner J, Caruso A, Kaczynski K, Gottlieb S, Mahoney E, et al. “Headache Tools to Stay in School”: Assessment, Development, and Implementation of an Educational Guide for School Nurses. Journal of School Health. 2016;86(9):645-52.

173. Lee J, Kubik MY. Child’s weight status and parent’s response to a school-based body mass index screening and parent notification program. The Journal of School Nursing. 2015;31(4):300-5.

174. Leff S, Bennett J. Audit of school entry health assessments: to maximise efficient use of health personnel at school entry assessments at 5 years. Public health. 1996;110(5):289-92.

175. Lehmkuhl H, Nabors L. Children with diabetes: Satisfaction with school support, illness perceptions and HbA1c levels. Journal of Developmental and Physical Disabilities. 2008;20(2):101.

176. Levy M, Heffner B, Stewart T, Beeman G. The efficacy of asthma case management in an urban school district in reducing school absences and hospitalizations for asthma. J Sch Health. 2006;76(6):320-4. Epub 2006/08/22. doi: 10.1111/j.1746-1561.2006.00120.x. PubMed PMID: 16918863.

177. Lewis CC, Alford-Winston A, Billy-Kornas M, McCaustland MD, Tachman CP. Care management for children who are medically fragile/technology-dependent. Issues Compr Pediatr Nurs. 1992;15(2):73-91. Epub 1992/04/01. doi: 10.3109/01460869209078244. PubMed PMID: 1308008.

178. Liberatos P, Leone J, Craig AM, Frei EM, Fuentes N, Harris IM. Challenges of asthma management for school nurses in districts with high asthma hospitalization rates. Journal of School Health. 2013;83(12):867-75.

179. Lightfoot J, Bines W. The role of nursing in meeting the health needs of school age children outside hospital: University of York, Social Policy Research Unit; 1996.

180. Lightfoot J, Bines W. Keeping children healthy: role of the school nurse. Nursing times. 1998;94(21):65-8.

181. Lightfoot J, Bines W. Working to keep school children healthy: the complementary roles of school staff and school nurses. Journal of Public Health. 2000;22(1):74-80.

182. Lindeke LL, Leonard BJ, Presler B, Garwick A. Family-centered care coordination for children with special needs across multiple settings. J Pediatr Health Care. 2002;16(6):290-7. Epub 2002/11/19. PubMed PMID: 12436098.

183. Liptzin DR, Gleason MC, Cicutto LC, Cleveland CL, White MK, Faino AV, et al. Developing, implementing, and evaluating a school-centered asthma program: step-up asthma program. The Journal of Allergy and Clinical Immunology: In Practice. 2016;4(5):972-9. e1.

184. Long G, Whitman C, Johansson M, Williams C, Tuthill R. Evaluation of a school health program directed to children with history of high absence. American journal of public health. 1975;65(4):388-93.

185. Looman WS, Presler E, Erickson MM, Garwick AW, Cady RG, Kelly AM, et al. Care coordination for children with complex special health care needs: the value of the advanced practice nurse's enhanced scope of knowledge and practice. J Pediatr Health Care. 2013;27(4):293-303. Epub 2012/05/09. doi: 10.1016/j.pedhc.2012.03.002. PubMed PMID: 22560803; PubMed Central PMCID: PMCPMC3433641.

186. Lunney M. The significance of nursing classification systems to school nursing. The Journal of school nursing: the official publication of the National Association of School Nurses. 1996;12(2):35-7.

187. Lunney M, Cavendish R, Luise B, Richardson K. Relevance of NANDA and health promotion diagnoses to school nursing. National Association of School Nurses. The Journal of school nursing: the official publication of the National Association of School Nurses. 1997;13(5):16-22.

188. Lunstead J, Weitzman ER, Kaye D, Levy S. Screening and brief intervention in high schools: School nurses' practices and attitudes in Massachusetts. Substance abuse. 2017;38(3):257-60.

189. Luthy KE, Thorpe A, Dymock LC, Connely S. Evaluation of an intervention program to increase immunization compliance among school children. The Journal of School Nursing. 2011;27(4):252-7.

190. Lynch E. School of thought. Nursing Standard. 2008;22(27).

191. Magalnick H, Mazyck D. Role of the school nurse in providing school health services. Pediatrics. 2008;121(5):1052-6. Epub 2008/05/03. doi: 10.1542/peds.2008-0382. PubMed PMID: 18450912.

192. Magee JA, Kenney DM, Mullin E. Efficacy of and advocacy for postural screening in public schools. Orthopaedic Nursing. 2012;31(4):232-5.

193. Major DA, Clarke SM, Cardenas RA, Taylor-Fishwick JC, Kelly CS, Butterfoss FD. Providing asthma care in elementary schools: Understanding barriers to determine best practices. Family & Community Health. 2006;29(4):256-65.

194. Maunder Y. My day as a school nurse. Education & Health. 2004;22(1):8-10. PubMed PMID: 106665543. Language: English. Entry Date: 20041119. Revision Date: 20150711. Publication Type: Journal Article. Journal Subset: Biomedical.

195. McKaig C, Hindi‐Alexander M, Myers TR, Castiglia P. Implementation of the school nurse practitioner role: Barriers and facilitators. Journal of School Health. 1984;54(1):21-3.

196. Medaglia F, Knorr RS, Condon SK, Charleston AC. School-based pediatric asthma surveillance in Massachusetts from 2005 to 2009. Journal of School Health. 2013;83(12):907-14. doi: <https://dx.doi.org/10.1111/josh.12109>. PubMed PMID: 24261525.

197. Melin A, Lenner RA. Prevention of further weight gain in overweight school children, a pilot study. Scandinavian journal of caring sciences. 2009;23(3):498-505.

198. Mickel CF, Shanovich KK, Evans MD, Jackson DJ. Evaluation of a school-based asthma education protocol: iggy and the inhalers. The Journal of School Nursing. 2016;33(3):189-97.

199. Moricca ML, Grasska MA, M BM, Morphew T, Weismuller PC, Galant SP. School asthma screening and case management: attendance and learning outcomes. J Sch Nurs. 2013;29(2):104-12. Epub 2012/07/17. doi: 10.1177/1059840512452668. PubMed PMID: 22797976.

200. Morris P, Baker D, Belot C, Edwards A. Preparedness for students and staff with anaphylaxis. Journal of School Health. 2011;81(8):471-6.

201. Morrison-Sandberg LF, Kubik MY, Johnson KE. Obesity prevention practices of elementary school nurses in Minnesota: Findings from interviews with licensed school nurses. The Journal of School Nursing. 2011;27(1):13-21.

202. Morton JL, Schultz AA. Healthy Hands: Use of alcohol gel as an adjunct to handwashing in elementary school children. J Sch Nurs. 2004;20(3):161-7. Epub 2004/05/19. doi: 10.1177/10598405040200030601. PubMed PMID: 15147226.

203. Muggeo MA, Stewart CE, Drake KL, Ginsburg GS. A school nurse-delivered intervention for anxious children: An open trial. School Mental Health. 2017;9(2):157-71.

204. Murray R. Introduction to the American Academy of Pediatrics policy statement. SAGE Publications Sage CA: Los Angeles, CA; 2008.

205. Naish J, Barr M. Records. Rights of access. Health Visitor. 1991;64(9):300-1.

206. Nauta C, Byrne C, Wesley Y. School nurses and childhood obesity: An investigation of knowledge and practice among school nurses as they relate to childhood obesity. Issues in Comprehensive Pediatric Nursing. 2009;32(1):16-30.

207. Nguyen TM, Mason KJ, Sanders CG, Yazdani P, Heptulla RA. Targeting blood glucose management in school improves glycemic control in children with poorly controlled type 1 diabetes mellitus. The Journal of pediatrics. 2008;153(4):575-8.

208. Kelly MP, McDaid D, Ludbrook A, Powell J. Economic appraisal of public health interventions. London: Health Development Agency. 2005.

209. Noyes K, Bajorska A, Fisher S, Sauer J, Fagnano M, Halterman JS. Cost-effectiveness of the School-Based Asthma Therapy (SBAT) program. Pediatrics. 2013;131(3):e709-17. Epub 2013/02/13. doi: 10.1542/peds.2012-1883. PubMed PMID: 23400614; PubMed Central PMCID: PMCPMC3581846.

210. Nutbeam D, Farley P, Smith C. England and Wales: perspectives in school health. Journal of School Health. 1990;60(7):318-23.

211. O'Donnell NL, Alles WF. School Nurse Demonstrates that Mini‐Grant Funding Can Improve Elementary Nutrition Education. Journal of School Health. 1983;53(5):316-9.

212. O'Toole AW, O'Toole R, Webster SW, Lucal B. Nurses' diagnostic work on possible physical child abuse. Public health nursing. 1996;13(5):337-44.

213. Oda DS. Is school nursing really the" invisible practice?". Journal of School Health. 1992;62(3):112-4.

214. Olowokere A, Okanlawon F. The effects of a school-based psychosocial intervention on resilience and health outcomes among vulnerable children. The Journal of School Nursing. 2014;30(3):206-15.

215. Paavilainen E, Åstedt‐Kurki P, Paunonen M. School nurses’ operational modes and ways of collaborating in caring for child abusing families in Finland. Journal of Clinical Nursing. 2000;9(5):742-50.

216. Paavilainen E, Helminen M, Flinck A, Lehtumaki L. How public health nurses identify and intervene in child maltreatment based on the national clinical guideline. Nurs Res Pract. 2014;2014:425460. Epub 2014/12/17. doi: 10.1155/2014/425460. PubMed PMID: 25505986; PubMed Central PMCID: PMCPMC4253703.

217. Paavilainen E, Tarkka MT. Definition and identification of child abuse by Finnish public health nurses. Public health nursing. 2003;20(1):49-55.

218. Pakieser RA, Starr DK, LeBaugh D. Nebraska School Nurses Identify Emotional Maltreatment of School‐Age Children: A Replication of an Ohio Study. Journal for Specialists in Pediatric Nursing. 1998;3(4):137-.

219. Palmore S, Millar K. Some common characteristics of pregnant teen who choose childbirth. The Journal of school nursing: the official publication of the National Association of School Nurses. 1996;12(3):19-22.

220. Pbert L, Druker S, Barton B, Schneider KL, Olendzki B, Gapinski MA, et al. A school‐based program for overweight and obese adolescents: a randomized controlled trial. Journal of School Health. 2016;86(10):699-708.

221. Pbert L, Druker S, Gapinski MA, Gellar L, Magner R, Reed G, et al. A school nurse‐delivered intervention for overweight and obese adolescents. Journal of School Health. 2013;83(3):182-93.

222. Peckover S, Trotter F. Keeping the focus on children: the challenges of safeguarding children affected by domestic abuse. Health & social care in the community. 2015;23(4):399-407.

223. Peery AI, Engelke MK, Swanson MS. Parent and teacher perceptions of the impact of school nurse interventions on children’s self-management of diabetes. The Journal of School Nursing. 2012;28(4):268-74.

224. Pennington N, Delaney E. The number of students sent home by school nurses compared to unlicensed personnel. J Sch Nurs. 2008;24(5):290-7. Epub 2008/10/23. doi: 10.1177/1059840508322382. PubMed PMID: 18941153.

225. Perry CS, Toole KA. Impact of school nurse case management on asthma control in school-aged children. Journal of School Health. 2000;70(7):303-.

226. Persaud DI, Barnett SE, Weller SC, Baldwin CD, Niebuhr V, McCormick DP. An asthma self-management program for children, including instruction in peak flow monitoring by school nurses. Journal of Asthma. 1996;33(1):37-43.

227. Petitgout JM, Pelzer DE, McConkey SA, Hanrahan K. Development of a hospital-based care coordination program for children with special health care needs. J Pediatr Health Care. 2013;27(6):419-25. Epub 2012/05/12. doi: 10.1016/j.pedhc.2012.03.005. PubMed PMID: 22575784.

228. Powell SB, Engelke MK, Neil JA. Seizing the moment: Experiences of school nurses caring for students with overweight and obesity. The Journal of School Nursing. 2018;34(5):380-9.

229. Presler B. Care coordination for children with special health care needs. Orthop Nurs. 1998;17(2 Suppl):45-51. Epub 1998/05/28. PubMed PMID: 9601413.

230. Pryjmachuk S, Graham T, Haddad M, Tylee A. School nurses’ perspectives on managing mental health problems in children and young people. Journal of Clinical Nursing. 2012;21(5‐6):850-9.

231. Pulcini JM, Marshall GD, Jr., Naveed A. Presence of food allergy emergency action plans in Mississippi. Ann Allergy Asthma Immunol. 2011;107(2):127-32. doi: https://dx.doi.org/10.1016/j.anai.2011.05.019. PubMed PMID: 21802020.

232. Putman-Casdorph H, Pinto S. Preliminary testing of an asthma distance education program (ADEP) for school nurses in Appalachia. The Journal of School Nursing. 2011;27(6):411-5.

233. Quaranta JE, Spencer GA. Barriers to asthma management as identified by school nurses. The Journal of School Nursing. 2016;32(5):365-73.

234. Quelly SB. Influence of perceptions on school nurse practices to prevent childhood obesity. The Journal of School Nursing. 2014;30(4):292-302.

235. Raible CA, Dick R, Gilkerson F, Mattern CS, James L, Miller E. School Nurse‐Delivered Adolescent Relationship Abuse Prevention. Journal of School Health. 2017;87(7):524-30.

236. Ramirez M, Harland K, Frederick M, Shepherd R, Wong M, Cavanaugh JE. Listen protect connect for traumatized schoolchildren: a pilot study of psychological first aid. BMC psychology. 2013;1(1):26.

237. Ramos MM, Greenberg C, Sapien R, Bauer‐Creegan J, Hine B, Geary C. Behavioral health emergencies managed by school nurses working with adolescents. Journal of school health. 2013;83(10):712-7.

238. Rasberry CN, Morris E, Lesesne CA, Kroupa E, Topete P, Carver LH, et al. Communicating with school nurses about sexual orientation and sexual health: perspectives of teen young men who have sex with men. The Journal of School Nursing. 2015;31(5):334-44.

239. Rebmann T, Elliott MB, Artman D, VanNatta M, Wakefield M. Impact of an Education Intervention on Missouri K‐12 School Disaster and Biological Event Preparedness. Journal of school health. 2016;86(11):794-802.

240. Reid J. Developing the role of the school nurse in public health. Health Education Journal. 1991;50(3):118-22.

241. Resnicow K, Allensworth D. Conducting a comprehensive school health program. Journal of School Health. 1996;66(2):59-63.

242. Rhodes DL, Draper M, Woolman K, Cox C. Practices and attitudes of Missouri school nurses regarding immunization records and select immunizations of graduating high school seniors. Journal of community health. 2017;42(5):872-7.

243. Rivkina V, Tapke DE, Cardenas LD, Harvey-Gintoft B, Whyte SA, Gupta RS. Identifying barriers to chronic disease reporting in Chicago Public Schools: a mixed-methods approach. BMC Public Health. 2014;14:1250. doi: <https://dx.doi.org/10.1186/1471-2458-14-1250>. PubMed PMID: 25481628.

244. Robbins LB, Pfeiffer KA, Maier KS, Lo Y-J, Wesolek SM. Pilot intervention to increase physical activity among sedentary urban middle school girls: a two-group pretest–posttest quasi-experimental design. The Journal of School Nursing. 2012;28(4):302-15.

245. Roden D. Proving the value of school nursing services. Health visitor. 1997;70:462-3.

246. Rodriguez E, Rivera DA, Perlroth D, Becker E, Wang NE, Landau M. School nurses' role in asthma management, school absenteeism, and cost savings: a demonstration project. J Sch Health. 2013;83(12):842-50. Epub 2013/11/23. doi: 10.1111/josh.12102. PubMed PMID: 24261518.

247. Rose DA, Chen SP, Souter CM. Development of an in-service education program by school nurses. J Community Health Nurs. 1987;4(3):171-8. Epub 1987/01/01. doi: 10.1207/s15327655jchn0403_7. PubMed PMID: 3650296.

248. Rose S. Nit nurse to nitty gritty: former school nurse Sharon Rose shares her views on the profession today and explains how she believes strong links with the whole team, including health visitors, would help to improve morale and protect the future health of young people. Community Practitioner. 2013;86(7):42-4.

249. Rosen BL, DiClemente R, Shepard AL, Wilson KL, Fehr SK. Factors associated with school nurses’ HPV vaccine attitudes for school-aged youth. Psychology, health & medicine. 2017;22(5):535-45.

250. Rosen BL, Goodson P, Thompson B, Wilson KL. School nurses' knowledge, attitudes, perceptions of role as opinion leader, and professional practice regarding human papillomavirus vaccine for youth. Journal of School Health. 2015;85(2):73-81.

251. Rote S. Losing sight of the future. Nursing times. 1997a;93(24):58-9.

252. Rote S. Healthy futures. Nurs Stand. 1997b;11(24):17. Epub 1997/03/05. doi: 10.7748/ns.11.24.17.s28. PubMed PMID: 9087052.

253. Rustia J, Hartley R, Hansen G, Schulte D, Spielman L. Redefinition of school nursing practice: integrating the developmentally disabled. Journal of School Health. 1984;54(2):58-62.

254. Salend SJ, Mahoney S. Teaching Proper Health Habits to Mainstreamed Students Through Positive Reinforcement. Journal of School Health. 1982;52(9):539-42.

255. Salmon DA, Moulton LH, Omer SB, Chace LM, Klassen A, Talebian P, et al. Knowledge, attitudes, and beliefs of school nurses and personnel and associations with nonmedical immunization exemptions. Pediatrics. 2004;113(6):e552-e9.

256. Savage J, Goodall G. Making a drama out of teenage obesity. Nursing Standard. 2006;20(32):10-. doi: 10.7748/ns.20.32.10.s12. PubMed PMID: 106470841. Language: English. Entry Date: 20060714. Revision Date: 20200708. Publication Type: Journal Article.

257. Schaffer MA, Anderson LJ, Rising S. Public health interventions for school nursing practice. The Journal of School Nursing. 2016;32(3):195-208.

258. Schols MW, De Ruiter C, Öry FG. How do public child healthcare professionals and primary school teachers identify and handle child abuse cases? A qualitative study. BMC Public Health. 2013;13(1):1-16.

259. Schonfeld D. Talking with elementary school-age children about AIDS and death: principles and guidelines for school nurses. The Journal of school nursing: the official publication of the National Association of School Nurses. 1996;12(1):26-32.

260. Schroeder K, Jia H, Wang YC, Smaldone A. Implementation of a school nurse-led intervention for children with severe obesity in New York City schools. Journal of pediatric nursing. 2017;35:16-22.

261. Schwartz FL, Denham S, Heh V, Wapner A, Shubrook J. Experiences of children and adolescents with type 1 diabetes in school: Survey of children, parents, and schools. Diabetes Spectrum. 2010;23(1):47-55.

262. Scott LK, Hall LM. Reliability and validity of the acanthosis nigricans screening tool for use in elementary school-age children by school nurses. The Journal of School Nursing. 2012;28(6):442-7.

263. Seidenberg A. School nurses: Improve the reception and sharpen the image. Journal of School Health. 1984;54(9):363-5.

264. Sekhar DL, Zalewski TR, Ghossaini SN, King TS, Rhoades JA, Czarnecki B, et al. Pilot study of a high-frequency school-based hearing screen to detect adolescent hearing loss. Journal of medical screening. 2014;21(1):18-23.

265. Sekhar DL, Kraschnewski JL, Stuckey HL, Witt PD, Francis EB, Moore GA, et al. Opportunities and challenges in screening for childhood sexual abuse. Child abuse & neglect. 2018;85:156-63.

266. Sherman JB, Alexander MA, Gomez D, Marole P. Intervention program for obese school children. Journal of community health nursing. 1992;9(3):183-90.

267. Singer B. Perceptions of school nurses in the care of students with disabilities. The Journal of School Nursing. 2013;29(5):329-36.

268. Skelley JP, Luthin DR, Skelley JW, Kabagambe EK, Ashraf AP, Atchison JA. Parental perspectives of diabetes management in Alabama public schools. Southern medical journal. 2013;106(4):274-9.

269. Smith S, Ilardi D. The school nurse as prevention specialist. School nurse news. 2008;25(5):28-32.

270. Snyder AA, Minnick K, Anderson DE. Children from broken homes: visits to the school nurse. The Journal of school health. 1980;50(4):189-94.

271. Speroni KG, Earley C, Atherton M. Evaluating the effectiveness of the Kids Living Fit™ program: A comparative study. The Journal of school nursing. 2007;23(6):329-36.

272. Spina JL, McIntyre CL, Pulcini JA. An intervention to increase high school students’ compliance with carrying auto-injectable epinephrine: a MASNRN study. The Journal of School Nursing. 2012;28(3):230-7.

273. Splett PL, Erickson CD, Belseth SB, Jensen C. Evaluation and sustainability of the healthy learners asthma initiative. J Sch Health. 2006;76(6):276-82. Epub 2006/08/22. doi: 10.1111/j.1746-1561.2006.00112.x. PubMed PMID: 16918855.

274. Spratt J, Philip K, Shucksmith J, Kiger A, Gair D. ‘We are the ones that talk about difficult subjects’: nurses in schools working to support young people’s mental health. Pastoral Care in Education. 2010;28:131-44. doi: 10.1080/02643944.2010.482145.

275. Sprinks J. Nurse-led initiative focuses on parents to lessen health-related school absences. Nursing Children and Young People (through 2013). 2011;23(10):6.

276. Squires C. How can we make a difference? Delivering quality health education. British Journal of School Nursing. 2013;8(9):452-4.

277. Stallard P, Simpson N, Anderson S, Hibbert S, Osborn C. The FRIENDS emotional health programme: Initial findings from a school‐based project. Child and Adolescent Mental Health. 2007;12(1):32-7.

278. Stallard P, Simpson N, Anderson S, Goddard M. The FRIENDS emotional health prevention programme. European child & adolescent psychiatry. 2008;17(5):283-9.

279. Stalter AM, Chaudry RV, Polivka BJ. Regional differences as barriers to body mass index screening described by Ohio school nurses. Journal of School Health. 2011a;81(8):437-48.

280. Stalter AM, Kaylor M, Steinke JD, Barker RM. Parental perceptions of the rural school’s role in addressing childhood obesity. The Journal of School Nursing. 2011b;27(1):70-81.

281. Stang JS, Story M, Kalina B. School-based weight management services: perceptions and practices of school nurses and administrators. American Journal of Health Promotion. 1997;11(3):183-5.

282. Staudt AM, Alamgir H, Long DL, Inscore SC, Wood PR. Developing and Implementing a Citywide Asthma Action Plan: A Community Collaborative Partnership. Southern medical journal. 2015;108(12):710-4.

283. Stephenson C. Visits by elementary school children to the school nurse. Journal of School Health. 1983;53(10):594-9.

284. Steele RG, Wu YP, Cushing CC, Jensen CD. Evaluation of child health matters: a web-based tutorial to enhance school nurses’ communications with families about weight-related health. The Journal of School Nursing. 2013;29(2):151-60.

285. Streeting J. Observations from QK: personal reflections on being the full-time school nurse working within a multidisciplinary student support team at one London secondary school. Community Practitioner. 2010;83(4):38-40.

286. Swallow W, Roberts JC. An evidence-based project demonstrating increased school immunization compliance following a school nurse–initiated vaccine compliance strategy. The Journal of School Nursing. 2016;32(6):385-9.

287. Szychlinski C, Schmeissing KA, Fuleihan Z, Qamar N, Syed M, Pongracic JA, et al. Food allergy emergency preparedness in Illinois schools: rural disparity in guideline implementation. The Journal of Allergy and Clinical Immunology: In Practice. 2015;3(5):805-7. e8.

288. Taras H, Wright S, Brennan J, Campana J, Lofgren R. Impact of school nurse case management on students with asthma. J Sch Health. 2004;74(6):213-9. Epub 2004/10/08. doi: 10.1111/j.1746-1561.2004.tb07935.x. PubMed PMID: 15468525.

289. Taylor A, Lizzi M, Marx A, Chilkatowsky M, Trachtenberg SW, Ogle S. Implementing a care coordination program for children with special healthcare needs: partnering with families and providers. J Healthc Qual. 2013;35(5):70-7. Epub 2012/08/24. doi: 10.1111/j.1945-1474.2012.00215.x. PubMed PMID: 22913270.

290. Telljohann SK, Dake JA, Price JH. Effect of full-time versus part-time school nurses on attendance of elementary students with asthma. J Sch Nurs. 2004;20(6):331-4. Epub 2004/11/25. doi: 10.1177/10598405040200060701. PubMed PMID: 15560730.

291. Terry D, Patel AD, Cohen DM, Scherzer D, Kline J. Barriers to seizure management in schools: perceptions of school nurses. Journal of child neurology. 2016;31(14):1602-6.

292. Thompson J. School health services in the United States: a view from the United Kingdom. Journal of School Health. 1989;59(6):243-5.

293. Thomson H. A successful approach to reduce youth smoking in Leicestershire. British Journal of School Nursing. 2012;7(9):441-7.

294. Thurston C, Walker S. Experiences and training in delivering sexual health care. British Journal of School Nursing. 2011;6(6):289-93.

295. Toole K, Perry CS. Increasing immunization compliance. The Journal of School Nursing. 2004;20(4):203-8.

296. Triggle N. Helping Traveller families to brush up on health facts. Nursing Children and Young People (2014+). 2014;26(2):8.

297. Trivedi M, Patel J, Lessard D, Kremer T, Byatt N, Phipatanakul W, et al. School nurse asthma program reduces healthcare utilization in children with persistent asthma. J Asthma. 2017;55(10):1131-7. Epub 2017/12/06. doi: 10.1080/02770903.2017.1396473. PubMed PMID: 29206057; PubMed Central PMCID: PMCPMC5988937.

298. Tsacoyianis R. Indoor air pollutants and sick building syndrome: a case study and implications for the community health nurse. Public Health Nursing. 1997;14(1):58-75.

299. Tucker S, Lanningham-Foster LM. Nurse-led school-based child obesity prevention. The Journal of School Nursing. 2015;31(6):450-66.

300. Turner KM, Shield JP, Salisbury C. Practitioners' views on managing childhood obesity in primary care: a qualitative study. British Journal of General Practice. 2009;59(568):856-62.

301. Urbinati D, Steele P, Harter B, Harrell D. The evolution of the school nurse practitioner: past, present, and future. The Journal of school nursing: the official publication of the National Association of School Nurses. 1996;12(2):6-9.

302. Van Cura M. The relationship between school-based health centers, rates of early dismissal from school, and loss of seat time. J Sch Health. 2010;80(8):371-7. Epub 2010/07/14. doi: 10.1111/j.1746-1561.2010.00516.x. PubMed PMID: 20618619.

303. Van Roeyen LS. Management of pediatric asthma at home and in school. Nurs Clin North Am. 2013;48(1):165-75. Epub 2013/03/08. doi: 10.1016/j.cnur.2012.12.006. PubMed PMID: 23465450.

304. Vanderpool RC, Breheny PJ, Tiller PA, Huckelby CA, Edwards AD, Upchurch KD, et al. Implementation and evaluation of a school-based human papillomavirus vaccination program in rural Kentucky. American journal of preventive medicine. 2015;49(2):317-23.

305. Vernberg EM, Nelson TD, Fonagy P, Twemlow SW. Victimization, aggression, and visits to the school nurse for somatic complaints, illnesses, and physical injuries. Pediatrics. 2011;127(5):842-8.

306. Vernon TM, Conner JS, Shaw BS, Lampe JM, Doster ME. An evaluation of three techniques improving immunization levels in elementary schools. American journal of public health. 1976;66(5):457-60.

307. Vessey JA, O’Neill KM. Helping students with disabilities better address teasing and bullying situations: A MASNRN study. The Journal of School Nursing. 2011;27(2):139-48.

308. Wall RB. Tai chi and mindfulness-based stress reduction in a Boston public middle school. Journal of Pediatric Health Care. 2005;19(4):230-7.

309. Watson P. Back to school: no other health professional has such a unique role in the education community, says school nurse Paul Watson. Nursing Standard. 2008;22(20):64-5.

310. Weismuller PC, Grasska MA, Alexander M, White CG, Kramer P. Elementary school nurse interventions: Attendance and health outcomes. The Journal of School Nursing. 2007;23(2):111-8.

311. Wells N, Johnson R, Salyer S. Interdisciplinary collaboration. Clin Nurse Spec. 1998;12(4):161-8. Epub 1999/02/13. doi: 10.1097/00002800-199807000-00014. PubMed PMID: 9987223.

312. Office W. Supporting Pupils with Medical Needs in Schools. Welsh Of®ce Circular 34/97, Welsh Health circularn97/31, Welsh Of®ce (Education Dept), Cardiff. 1997.

313. Office W. Better Health, Better Wales. HMSO, London. Whit®eld C. (1995) Raising the pro®le of the school nurse. Nursing Times 91, 11. 1998.

314. Werch CE, Carlson JM, Pappas DM, DiClemente CC. Brief nurse consultations for preventing alcohol use among urban school youth. Journal of School Health. 1996;66(9):335-8.

315. White DH. SNP responds to research article: "School nurse practitioners analysis of questionnaire and time/motion data". Journal of School Health. 1981;51(9):584. PubMed PMID: 6913693.

316. Whitfield C. Raising the profile of the school nurse. Nurs Times. 1995;91(45):11. Epub 1995/11/08. PubMed PMID: 7494688.

317. Whitmarsh J. School nurses' skills in sexual health education. Nursing standard (Royal College of Nursing (Great Britain): 1987). 1997;11(27):35-41.

318. Whitmore K. School refusal. Health visitor. 1988;61(11):349-51.

319. Wicklander MK. The United Kingdom National Healthy School Standard: A framework for strengthening the school nurse role. The Journal of school nursing. 2005;21(3):132-8.

320. Wiggs-Stayner KS, Purdy TR, Go GN, McLaughlin NC, Tryzynka PS, Sines JR, et al. The impact of mass school immunization on school attendance. J Sch Nurs. 2006;22(4):219-22. Epub 2006/07/22. doi: 10.1177/10598405050220040601. PubMed PMID: 16856776.

321. Williams AD, Warrington V. Get Fit Kids: a feasibility study of a pedometer-based walking program. Bariatric Nursing and Surgical Patient Care. 2011;6(3):139-43.

322. Wilson EL, Egger JR, Konty KJ, Paladini M, Weiss D, Nguyen TQ. Description of a school nurse visit syndromic surveillance system and comparison to emergency department visits, New York City. Am J Public Health. 2014;104(1):e50-e6. doi: 10.2105/AJPH.2013.301411. PubMed Central PMCID: PMC24228684.

323. Wing R, Amanullah S, Jacobs E, Clark MA, Merritt C. Heads Up: Communication Is Key in School Nurses' Preparedness for Facilitating "Return to Learn" Following Concussion. Clin Pediatr (Phila). 2016;55(3):228-35. Epub 2015/07/02. doi: 10.1177/0009922815592879. PubMed PMID: 26130394.

324. Winkelstein ML. Teaching Pregnant Adolescents to Cope with Environmental Smoke: Culturally relevant materials and role playing are effective in a program for African American students. Initial results are promising. MCN: The American Journal of Maternal/Child Nursing. 1995;20(1):38-45.

325. Wong EM, Cheng MM. Effects of motivational interviewing to promote weight loss in obese children. Journal of Clinical Nursing. 2013;22(17-18):2519-30.

326. Wright K, Giger JN, Norris K, Suro Z. Impact of a nurse-directed, coordinated school health program to enhance physical activity behaviors and reduce body mass index among minority children: A parallel-group, randomized control trial. International Journal of Nursing Studies. 2013;50(6):727-37.
