# Supplementary material for: The current state of international research on the effectiveness of school nurses in promoting the health of children and adolescents: An overview of reviews
Source: PLoS One. 2023 Feb 22;18(2):e0275724. doi: 10.1371/journal.pone.0275724 (PMC9946271; doi:10.1371/journal.pone.0275724)
Supplement: S2 Table — (DOCX) [file pone.0275724.s003.docx]

S2 Table: Characteristics of *primary studies* (k=289) included

|  | Studied Population | | | | | | | | | | | |  |  | Subject matter | | | | | | | | | | | | | | | |  |  | |  |
| --- | --- | --- | --- | --- | --- | --- | --- | --- | --- | --- | --- | --- | --- | --- | --- | --- | --- | --- | --- | --- | --- | --- | --- | --- | --- | --- | --- | --- | --- | --- | --- | --- | --- | --- |
| First author (year) [country] | Disadvantaged children | | All school children | | Teachers | | Parents | | School Nurses | | Other | | Study design (Method) [number of participants] | Main topic | Mental health | | | Somatic Illness | | SN role and education | | Prevention & Health promotion and literacy | | Interdisciplinary aspects | | Attendance / academic achievement | | Care Coordination/ Case management | | | Intervention (Data collection) | Outcome | | Information value |
| Adams (1990) [UK] [1] | |  | | X | | X | | X | | X | |  | Qualitative (case study) [22 children, 8 teachers, 18 parents] | How pupils, parents and teachers view the school nurse, compared to school nurses’ own perception | |  | | |  | | X | |  | |  | |  | |  | No intervention (questionnaire) | | | Small differences between the perception of the school nurse, but not statistically significant | A-III  Limited |
| Alizadeh et al. (2011) [Sweden] [2] | |  | |  | |  | |  | | X | |  | Qualitative (grounded theory method) [10] | Violence related to protection of family honour | |  | | |  | |  | | X | |  | |  | |  | No intervention (semi-structured interview) | | | School nurses and counsellors felt professionally hampered as they could not always solve problems in regular ways | C-II  Limited |
| Allen (2003) [USA] [3] | |  | | X | |  | |  | |  | |  | Quantitative non-randomized study (cross-sectional) [10,000] | School attendance | |  | | |  | |  | |  | |  | | X | |  | Experimental group: full-time school nurse; Control group: no school nurse (archival records, structured interviews, daily checkout forms, and surveys of parents) | | | A full-time school nurse reduces the number of students who leave school because of a medical complaint | E-IV  High |
| Allen et al. (2012) [USA] [4] | |  | |  | |  | |  | | X | |  | Quantitative (survey) [2,040] | School nurse perspective on experiencing and managing somatic emergencies | |  | | | X | |  | |  | |  | |  | | X | No intervention (online survey) | | | Schools represented by nurses appear to be somewhat, but not ideally equipped to handle described somatic emergencies | C-II  Limited |
| Allensworth and Bradley (1996) [USA] [5] | |  | |  | |  | |  | | X | |  | Qualitative (narrative research) [n/a] | Possibility of using the traditional school nurse to promote and advance traditional care in schools | |  | | |  | |  | | X | |  | |  | | X | No intervention (no data) | | | Explored issues: screening tools, administering surveys, interdisciplinarity, confidentiality, barriers. | A-II  Limited |
| Allison et al. (2014) [USA] [6] | |  | | X | |  | |  | |  | |  | Quantitative descriptive (prevalence study without comparison group) [182] | Identifying symptoms of depression and anxiety in students in the school setting | | X | | |  | |  | |  | |  | |  | |  | The use of two validated screening tools to identify symptoms of depression and anxiety (questionnaire) | | | Both screens improved identification and referral of children suffering from anxiety & depression | E-III  Moderate |
| Amillategui et al. (2007) [Spain] [7] | |  | |  | |  | | X | |  | |  | Quantitative descriptive (survey) [499] | Identifying special needs of children with type 1 diabetes | | X | | |  | |  | |  | |  | |  | | X | No intervention (self-reporting questionnaire) | | | Training, increase of school nurses, better availability of diabetes associations | C-III  Moderate |
| Anderson (1994) [USA] [8] | |  | |  | |  | |  | | X | |  | Qualitative (qualitative description) [211] | School nurses` perception of their responsibilities | |  | | |  | |  | |  | |  | |  | | X | No intervention (questionnaire) | | | School nurse responsibilities had increased or stayed the same | B-II  Limited |
| Antonelli and Antonelli (2004) [USA] [9] | | X | |  | |  | |  | |  | |  | Quantitative descriptive (case series) [444] | Care coordination services for children with special health care needs (CSHCN) | |  | | |  | |  | |  | |  | |  | | X | No intervention (measurement of activities of Care coordination services for children with special health care needs (CSHCN) and average salaries) | | | Costs of providing care coordination services to CSHCN in a medical home are appreciable but not prohibitive. | A-III  Limited |
| Antonelli et al. (2009a) [USA] [10] | |  | |  | |  | |  | |  | | X | Qualitative (grounded theory method) [46] | Framework for paediatric care coordination (definition, characteristics, implementation model) | |  | | |  | |  | |  | |  | |  | | X | No intervention (literature review, consultation with key informants) | | | Effective care coordination is best provided in the context of a health care team with established relationships with families, clinicians and other professionals | A-II  Limited |
| Antonelli et al. (2009b) [USA] [11] | |  | |  | |  | |  | |  | | X | Quantitative descriptive (case series) [6 practices] | Testing use of the care-coordination measurement tool in paediatric practices | |  | | |  | |  | |  | |  | |  | | X | Record of all the non-reimbursable care-coordination activity encounters performed by any office-based personnel | | | Office-based nurses providing care coordination were responsible for reducing higher cost use outcomes | A-III  Limited |
| Aruda et al. (2011) [USA] [12] | |  | |  | |  | | X | |  | |  | Quantitative descriptive (survey) [41] | Parent’s view of health care needs for CSHCN; communication between schools and health care providers | |  | | |  | |  | |  | |  | |  | | X | No intervention (survey adapted from the American Academy of Paediatrics) | | | High utilization of primary care services but persistent difficulty accessing services | B-II  Limited |
| Atherton (2009) [UK] [13] | |  | | X | |  | |  | |  | |  | No study design [n/a] | Sexual health service | |  | | |  | |  | | X | |  | |  | |  | Contraception, advice and support were offered within a confidential environment (unknown) | | | 1,400 school children in first 1,5 years have made use of the service, very positive reports | C-I  Limited |
| Attwood et al. (2012) [UK] [14] | |  | | X | |  | |  | |  | |  | Study 1: Quantitative randomized controlled trial; Study 2: Quantitative non-randomized study (pre-post design) [12] | Effectiveness of computerised Cognitive Behaviour Therapy (cCBT) to improve emotional health in school | | X | | |  | |  | |  | |  | |  | |  | Study 1: Experimental group: cCBT; Control group: Gaming Study 2: “Think, Feel, Do” one-on-one intervention by school nurses | | | This study shows that cCBT in schools is viable and may result in immediate therapeutic benefits | E-IV  High |
| No Autor (1997) [unknown] [15] | |  | |  | |  | |  | | X | |  | Qualitative (phenomenology) [n/a] | Initiating health programmes in schools and communities | |  | | |  | |  | | X | |  | |  | |  | No intervention (n/a) | | | By assessing the situation carefully, taking time to generate trust, nurses can make a real difference to many young lives. | C-II  Limited |
| Badger and Brown (2005) [UK] [16] | |  | |  | | X | |  | |  | |  | Qualitative (case study) [48] | Primary school’s perception on school nurse service | |  | | |  | |  | |  | | X | |  | | X | No intervention (questionnaire) | | | Schools were largely satisfied with current levels of school nurse input | B-II  Limited |
| Bagnall (1995) [UK] [17] | |  | |  | |  | |  | | X | |  | Qualitative (case study) [288] | Success of school nurses in rubella and measles immunisation programme | |  | | |  | |  | | X | |  | |  | |  | No intervention (questionnaire) | | | School nurses played a vital part in avoiding a major measles epidemic | C-II  Limited |
| Bagnall (1997) [UK] [18] | |  | | X | |  | |  | |  | |  | No study design | Children’s health | |  | | |  | |  | | X | |  | |  | |  | No intervention (n/a) | | | No outcome | A-I  Limited |
| Baisch et al. (2011) [USA] [19] | |  | |  | | X | |  | |  | | X | Mixed-method approach: (1) quantitative non-randomized (cross-sectional) (2) quantitative descriptive (survey) [634] | Value of school nurses | |  | | |  | |  | | X | |  | |  | |  | No intervention (survey, electronic school record) | | | School nurses have positive effects on immunization rates, accuracy of student health records, and management of health concerns | C-IV  Moderate |
| Baker et al. (2015) [USA] [20] | |  | |  | |  | |  | | X | |  | Qualitative (qualitative description) [446] | How do schools meet the needs of CSHCN children and who provides these services | |  | | |  | |  | |  | |  | |  | | X | No intervention (cross-sectional online survey) | | | Lack of identification of CSHCN; communi-cation barriers impair the ability to deliver care | A-II  Limited |
| Bannink et al. (2014) [The Netherlands] [21] | |  | | X | |  | |  | |  | |  | Quantitative randomized controlled trials [1,256] | Effectiveness of a web-based tailored intervention | |  | | |  | |  | | X | |  | |  | |  | The E-health4Uth Intervention (questionnaire) | | | Minor positive results in health-related quality of life | C-V  Moderate |
| Barnard-Brak et al. (2017) [USA] [22] | |  | |  | |  | | X | |  | |  | Quantitative descriptive (survey) [40,242] | The role of family-centred care for children with special health care needs | |  | | |  | |  | |  | |  | |  | | X | No intervention (survey) | | | Family-centred care is associated with fewer absences and improved care coordination with schools | A-III  Limited |
| Barrett (2000) [USA] [23] | |  | |  | |  | |  | | X | |  | Study 1: Qualitative (case study) [n/a]; Study 2: Quantitative descriptive (survey) [unknown] | Improved case management service for children with special needs through School Nurse education | |  | | |  | | X | |  | |  | |  | | X | No intervention (n/a) | | | This program has highlighted the need for coordination of services at schools. | B-II  Limited |
|  | |  | |  | |  | |  | |  | |  |  |  | |  | | |  | |  | |  | |  | |  | |  |  | | |  |  |
| Bartfay and Bartfay (1994) [Canada] [24] | |  | | X | |  | |  | |  | |  | Quantitative randomized controlled trials [23] | Promoting health by increasing knowledge about the prevention of chronic diseases | |  | | |  | |  | | X | |  | |  | |  | Board game (knowledge test) | | | Significant gains in knowledge about chronic illnesses | C-IV  Moderate |
| Bednarz (1998) [USA] [25] | |  | |  | |  | |  | | X | |  | Qualitative (case study) [1] | Demonstrate how useful the Omaha System is as a tool to school nurse case management | |  | | |  | |  | |  | |  | |  | | X | Omaha System was implemented (unknown) | | | Omaha System as a classification system is beneficial to school nursing practice and offers a way to show their effectiveness | B-II  Limited |
| Bergren (2016) [USA] [26] | |  | |  | |  | |  | | X | |  | Qualitive (case study) [90] | Evaluating the feasibility of collecting school nurse data | |  | | |  | | X | |  | |  | |  | |  | Audio streamed webinar (survey) | | | Data collection was feasible for 76% of those who elected to participate | A-II  Limited |
|  | |  | |  | |  | |  | |  | |  |  |  | |  | | |  | |  | |  | |  | |  | |  |  | | |  |  |
| Betz and Redcay (2005) [USA] [27] | |  | |  | |  | |  | | X | |  | Qualitative (case study) [n/a] | Dimensions of the Transition Service Coordinator Role | |  | | |  | |  | |  | | X | |  | | X | No intervention (n/a) | | | A transition service coordinator requires nurses to provide direct services blending both paediatric and adult healthcare needs and to function in advanced practice roles | B-II  Limited |
|  | |  | |  | |  | |  | |  | |  |  |  | |  | | |  | |  | |  | |  | |  | |  |  | | |  |  |
| Bhardwa (2013) [UK] [28] | |  | | X | |  | |  | |  | |  | No study design | Doc Ready App as a tool to promote mental health | | X | | |  | |  | |  | |  | |  | |  | No intervention (no data) | | | School nurses can engage with these innovations (apps) to break down barriers. | B-I  Limited |
| Blaakman et al. (2014) [USA] [29] | | X | |  | |  | |  | |  | |  | Qualitative (qualitative descriptive) [28] | Asthma care | |  | | | X | |  | |  | |  | |  | | X | Research nurses provided asthma education (semi-structured interviews) | | | The school nurse’s role as an ally may support teens’ transition to medication independence | C-III  Moderate |
| Blackwell et al. (2017) [USA] [30] | |  | |  | |  | |  | | X | |  | Quantitative descriptive (cross-sectional survey) [142] | Helping students transition back to school following a concussion | |  | | | X | |  | | X | |  | |  | | X | No intervention (survey) | | | Communication among treating physicians, parents, and school personnel, improved education and standardized management tools | C-III  Moderate |
| Bolton (1994) [UK] [31] | |  | |  | | X | | X | | X | | X | Quantitative descriptive (survey) [82 parents, 10 nurs., 18 teach., 6 doctors] | School entry screening by school nurses | |  | | |  | |  | |  | |  | |  | | X | Screening tool for school nurses (survey) | | | The system has been shown to be satisfactory for the children and their parents | C-III  Moderate |
| Bonaiuto (1995) [USA] [32] | |  | |  | |  | |  | | X | |  | Qualitative (phenomenology) [20] | School nurses' competence in caring for students who depend on medical technology | |  | | |  | | X | |  | |  | |  | |  | No intervention (questionnaire) | | | School nurses generally felt competent in dealing with children who require medical technology in school | B-II  Limited |
| Bonaiuto (2007) [USA] [33] | |  | | X | |  | |  | |  | |  | Quantitative descriptive (case study) [240, 520, 685, 1,625] | Impact of school nurse case management on health outcomes | |  | | |  | |  | | X | |  | | X | | X | School nurse case management (retrospective review of health records documented by school nurses) | | | 84% of the students’ case management improved in 1 or more of the 5 target health outcomes. | E-III  Moderate |
| Bonny et al. (2000) [USA] [34] | |  | | X | |  | |  | |  | |  | Quantitative descriptive (survey) [1,959] | School disconnectedness: identifying adolescents at risk | | X | | | X | |  | | X | |  | | X | |  | No intervention (modified version of the in-school survey) | | | Declining health status, increasing school nurse visits, cigarette use, and lack of out-of-school involvement are associated with disconnectedness | A-III  Limited |
| Bonsergent et al. (2013a) [France] [35] | |  | | X | |  | |  | |  | |  | Quantitative randomized controlled trial [3,538] | Overweight and obesity prevention for adolescents | |  | | | X | |  | | X | |  | |  | |  | a) education: nutritional knowledge/ skills; b) environment: healthy dietary items/ physical activity; c) screening and care: if necessary, adapted care | | | Education and environment were not more effective than no strategy intervention. Screening and care showed small effects | D-V  High |
| Bonsergent et al. (2013b) [France] [36] | | X | |  | |  | |  | |  | |  | Quantitative randomized controlled trial [1,804] | Process evaluation of screening strategy for overweight and obese students | |  | | | X | |  | |  | |  | |  | | X | Detection of overweight and obesity, medical interview and adapted care management | | | A structured strategy combining screening and adapted care management is effective in reducing overweight but presents challenges in encouraging adolescents to participate. | D-V  High |
| Borawski et al. (2015) [USA and Canada] [37] | |  | | X | |  | |  | |  | |  | Quantitative randomized controlled trial [1,357] | Effectiveness of health education by teachers and school nurses | |  | | |  | |  | | X | |  | |  | |  | Intervention group taught by teacher and intervention group taught by school nurse (questionnaire) | | | Equally effective changes when taught by teachers and school nurses; teaching technical and interpersonal skills may require a school nurse | E-V  High |
| Boyer-Chanroong and Deaver (2000) [USA] [38] | |  | | X | |  | |  | |  | |  | Qualitative (case study) [unknown] | Strategies for school nurses to implement preteen requirements for vaccinations | |  | | |  | | X | | X | |  | |  | |  | Education on vaccination laws + Information for parents (keeping track of vaccination levels of individual students) | | | Possible to achieve a reasonably high level of compliance even in the first year of a new preteen vaccination law | B-II  Limited |
| Bradley (1997) [USA] [39] | |  | |  | |  | |  | |  | | X | Qualitative (narrative research) | Framework to assist nurses in evaluating their ability to be effective health care providers in schools | |  | | |  | | X | | X | |  | |  | |  | No intervention (n/a) | | | School nurses are challenged to determine which contributions they are most qualified for. | B-II  Limited |
| Bradley (1998) [USA] [40] | |  | |  | |  | |  | | X | |  | Qualitative (case study) [20] | What research-based information is needed to improve and communicate school nursing services | |  | | |  | |  | |  | |  | |  | |  | No intervention (focus groups) | | | Research priorities derived from the group process | A-II  Limited |
| Brindis et al. (1998) [USA] [41] | |  | |  | |  | |  | | X | |  | Quantitative descriptive (case series) [6] | Redesigning a school health workforce | |  | | |  | | X | |  | |  | |  | |  | Training program for school nurses (number and types of student visits and interviews) | | | Expanding the traditional role of the school nurse is one key avenue to shaping the field of school health for the 21st century. | B-III  Limited |
| Bronsnan (1987) [USA] [42] | |  | | X | |  | |  | |  | |  | Quantitative non-randomized study (non-randomized controlled trials) [410] | Long-term course of an elementary sexuality program | |  | | |  | |  | | X | |  | |  | |  | Sex education program (test scores) | | | The nurse is an important resource in elementary school sex education | C-IV  Moderate |
| Brother (1998) [USA] [43] | |  | |  | |  | |  | | X | |  | Qualitative (narrative research) [1] | Proposal for a student assistant program by a school nurse | |  | | |  | |  | | X | |  | |  | | X | No intervention (n/a) | | | The partnership between school nursing and student assistance works well at our high school | C-II  Limited |
| Broussard (2008) [USA] [44] | |  | |  | |  | |  | | X | |  | Qualitative (grounded theory method) [10] | Empowerment in school nursing practice | |  | | |  | | X | |  | |  | |  | |  | No intervention (interviews) | | | Feeling of having a positive impact on the health of children, leading to job satisfaction and feelings of value. | B-II  Limited |
| Brustrom et al. (2012) [USA] [45] | |  | |  | |  | |  | |  | | X | Qualitative (qualitative description) [43 clinic staff and 38 care givers] | Care Coordination in the spina Bifida clinic setting | |  | | |  | |  | |  | |  | |  | | X | No intervention (interviews) | | | Study findings suggest ways that care might be coordinated optimally in spina bifida clinics. | A-II  Limited |
| Bruzzese et al. (2006) [USA] [46] | | X | |  | |  | |  | |  | |  | Quantitative non-randomized study (controlled trials) [591] | Preventive network of care to improve school students’ control of asthma | |  | | | X | | X | |  | |  | |  | |  | School nurses training and preventative care activities for children with asthma (telephone interviews, number of symptoms, activity-logs by nurses) | | | The intervention had no impact on the use of urgent health care services, school attendance, or caregiver’s quality of life | E-IV  High |
| Bryan and Cook (1967) [USA] [47] | |  | |  | |  | |  | | X | |  | Quantitative non-randomized study (controlled trials) [Exp.:307; Control.:284] | Redirection of school nursing services in culturally deprived neighbourhoods. | | |  | |  | | X | |  | |  | |  | |  | School nurse guide and workshops (health form filled out by parents, health examination, dental examination, parental referrals) | | | Personal contacts by the school nurse with parents in culturally diverse areas will increase parental promotion of children’s health. | C-IV  Moderate |
| Bucher et al. (1998) [USA] [48] | |  | |  | |  | |  | | X | |  | Quantitative descriptive (survey) [125] | Asthma care | |  | | | X | |  | |  | |  | |  | |  | No intervention (questionnaires) | | | The findings indicated a need for asthma education, as well as guidelines and protocols for the treatment of children with asthma | A-III  Limited |
| Buckland et al. (2005a) [UK] [49] | |  | |  | |  | |  | | X | |  | Qualitative (phenomenology) – First of three papers [2] | School Nurse innovation project (preventing exclusion) | |  | | |  | |  | | X | |  | |  | |  | No intervention (experience of two school nurses) | | | School nurses can take on the role as a child-centered health practitioner | C-II  Limited |
| Buckland et al. (2005b) [UK] [50] | | X | |  | |  | |  | |  | |  | Quantitative non-randomized study (pre-post design) – Second of three papers [parents and teachers of 6 children] | School nurse-led intervention to tackle challenging behaviour | |  | | |  | |  | | X | |  | |  | |  | Range of nurse-led interventions in three schools (SDQ) | | | Interventions were affected in some cases | E-IV  High |
| Butler (2013) [UK] [51] | |  | | X | |  | |  | |  | |  | Qualitative (narrative research) [n/a] | Web-based health resource “Health Matters” website | |  | | |  | |  | |  | |  | |  | |  | No intervention (n/a) | | | To engage our teenage population in health matters, we need to embrace new technologies. | E-II  Limited |
|  | |  | |  | |  | |  | |  | |  |  |  | |  | | |  | |  | |  | |  | |  | |  |  | | |  |  |
| Cady et al. (2014) [USA] [52] | | X | |  | |  | |  | |  | |  | Quantitative descriptive (survey) [27] | Attributes of advanced practice registered nurse (APRN) Care Coordination for Children with Medical Complexity | |  | | | X | |  | |  | |  | |  | | X | No intervention (telehealth and survey) | | | The APRN care coordination model has potential for changing the health management processes for children with medical complexity. | A-III  Limited |
| Cameron et al. (1999) [Canada] [53] | |  | | X | |  | |  | |  | |  | Quantitative randomized controlled trials [3,972] | Provider and training method effectiveness on outcomes of a social influences smoking prevention program | |  | | |  | |  | | X | |  | |  | |  | 3-6 lessons (survey) | | | Reduced smoking rates in high-risk schools but not in low-risk schools, teachers and nurses were equally effective providers | E-V  High |
| Carpenter et al. (2013) [USA] [54] | | X | |  | |  | |  | |  | |  | Quantitative non-randomized study (pre-post design) [330] | Sustaining school-based asthma interventions through policy and practice change | |  | | | X | |  | |  | |  | |  | |  | Varying interventions based on needs of communities; school-based programs with asthma action plans (survey, qualitative data reported by assessors) | | | Significant improvements regarding asthma symptoms and management | C-IV  Moderate |
| Carter (1997) [UK] [55] | |  | |  | |  | |  | |  | | X | Qualitative (phenomenology) [n/a] | Call for governmental action to improve child health services | |  | | |  | |  | | X | |  | |  | |  | No intervention (no data) | | | School nursing is associated with high costs and sharing responsibilities with families and is therefore not implemented as much as it should be | A-II  Limited |
| Chally (1998) [USA] [56] | |  | |  | | X | |  | |  | |  | Quantitative randomized controlled trial [117] | Develop, implement and evaluate a school personnel training program on eating disorders | |  | | |  | | X | | X | |  | |  | |  | Training program (count of students at risk, survey) | | | Significant difference in knowledge about risk factors of eating disorders in high school personnel | B-V  Limited |
| Chase et al. (2010) [UK] [57] | |  | |  | |  | |  | | X | |  | Qualitative (phenomenology) [62] | Identifying aspects of school nurse management | |  | | |  | | X | |  | |  | |  | |  | No intervention (interviews, survey, case studies) | | | School nurses have a variety of tasks including health promotion, Personal, social and health education (PSHE) education and more ‘traditional’ nursing roles | B-II  Limited |
| Chen et al. (1991) [USA] [58] | |  | |  | |  | |  | | X | |  | Quantitative non-randomized study (case-control study) [578] | Effects of a school-based, nurse-run prenatal counselling program for pregnant adolescents | |  | | |  | |  | | X | |  | |  | | X | School nurse-led counseling program (counting visits to school nurse and birth weight of child) | | | Significantly higher % received adequate prenatal care than did the controls, results on birth weight not significant | E-IV  High |
| Chilvers (2011) [UK] [59] | |  | |  | |  | |  | |  | | X | Qualitative (narrative research) [n/a] | Implementing social media use into the school nurse practice | |  | | |  | | X | | X | |  | |  | |  | No intervention (n/a) | | | Social media can increase the accessibility to health services for young people | A-II  Limited |
| Chokshi et al. (2015) [USA] [60] | |  | |  | |  | |  | | X | |  | Quantitative non-randomized study (case-control study) [62] | School nurse training in food allergy for a long-term increase in epinephrine availability | |  | | | X | |  | |  | |  | |  | |  | School nurse training (survey) | | | Significant increase in epinephrine availability in both non-low and low socioeconomic schools | C-IV  Moderate |
| Christiansen et al. (1997) [USA] [61] | | X | |  | |  | |  | |  | |  | Quantitative non-randomized study (case-control study [42] | Asthma education program for students to increase knowledge and decrease asthma severity | |  | | | X | | X | |  | |  | |  | |  | Asthma education for students by nursing staff (quiz, physical measures) | | | Highly significant changes in knowledge, significant changes in asthma symptoms | E-IV  High |
| Cicutto et al. (2013) [Canada and USA] [62] | | X | |  | |  | |  | |  | |  | Quantitative randomized controlled trial [1,316] | Public health nurse-delivered asthma program to elementary schools | |  | | | X | |  | |  | |  | | X | |  | Asthma self-management education program and a program for an asthma-friendly school environment (surveys, interviews, urgent care numbers) | | | Improvements at the child and school level for the intervention group | C-V  Moderate |
| Clapp (2009) [UK] [63] | | X | |  | |  | |  | |  | |  | No study design | Reducing teenage pregnancy rates | |  | | |  | | X | | X | |  | |  | |  | No intervention (no data) | | | The service has been successful in reaching high-risk groups | B-I  Limited |
| Clarke (2000) [UK] [64] | |  | |  | |  | |  | | X | |  | Quantitative descriptive (survey) [93] | Perceptions of workers on the handling of child protection issues | |  | | |  | | X | | X | |  | |  | |  | No intervention (interviews) | | | School nurses are often the most significant health worker who need more recognition | C-III  Moderate |
| Clausson and Berg (2008) [Sweden] [65] | | X | |  | |  | |  | |  | |  | Quantitative non-randomized studies (pre-post design) [4] | Effectiveness of therapeutic conversations with families among adolescent girls in a school setting | | X | | |  | |  | |  | |  | |  | |  | Therapeutic conversations with families using genograms, ecomaps, interventive questions, and other family nursing interventions (SDQ) | | | Involving the family when school children’s recurrent mental health problems are addressed may reduce future suffering. | E-IV  High |
| Coates (2011) [UK] [66] | |  | |  | |  | |  | | X | |  | Quantitative descriptive (survey) [62] | School nurse’s current practice and difficulties | |  | | |  | |  | | X | |  | |  | |  | No intervention (survey) | | | School nurses suffer from staff shortage, high and demanding caseloads, and an inability to truly carry out the government’s public health agenda | A-III  Limited |
|  | |  | |  | |  | |  | |  | |  |  |  | |  | | |  | |  | |  | |  | |  | |  |  | | |  |  |
| Coleman and Hawkins (1970) [USA] [67] | | X | |  | |  | |  | |  | |  | Qualitative (case study) [175] | Early identification of learning defects to prevent failing by nurses and teachers | |  | | |  | |  | |  | | X | | X | |  | Close observation of and interaction with children (unknown) | | | School nurses must become involved in the early identification and remediation of developmental lags and health problems in children | D-II  Limited |
| Costante (1996) [USA] [68] | |  | | X | |  | |  | |  | |  | Qualitative (narrative research) [n/a] | Supporting student success: School nurses make a difference | |  | | |  | | X | | X | |  | |  | |  | No intervention (n/a) | | | Formal/ informal research for a better documentation of interventions | C-II  Limited |
| Cox et al. (2014) [USA] [69] | | X | |  | |  | |  | |  | |  | Quantitative descriptive (survey) [425] | Development and validation of a survey tool (PRISM) to identify diabetes self-management barriers | |  | | | X | |  | |  | |  | |  | |  | No intervention (survey, medical record) | | | PRISM could be used in clinical practice to identify each child and family’s unique self-management barriers | A-III  Limited |
| Crickmore et al. (2002) [USA] [70] | | X | |  | |  | |  | |  | |  | Quantitative non-randomized study (pre-post design) [277] | Development of an asthma management program, increase of medical facilities | |  | | | X | |  | |  | |  | |  | |  | Multiple interventions, including home visits and phone calls by one social worker and two clinicians (number of hospital admissions) | | | Intervention group had a significant reduction in number of inpatient admissions and days spent in hospital | A-IV  Limited |
| Davies (2012) [UK] [71] | |  | | X | |  | |  | |  | |  | Qualitative (phenomenology) [n/a] | Talking about health and well-being issues | | X | | |  | |  | |  | |  | |  | |  | No intervention (n/a) | | | “Talking Wellbeing” discussion kit can be used in schools and at home | C-II  Limited |
| Davis (2007) [UK] [72] | |  | | X | |  | |  | |  | |  | Qualitative (phenomenology) [n/a] | “Smoke Free is Healthy” project | |  | | |  | |  | | X | |  | |  | |  | No intervention (n/aa) | | | Apart from the “stop smoking clinic” many parents called to talk about other health issues they are worried about. | C-II  Limited |
| Davis et al. (2016) [USA] [73] | |  | |  | |  | |  | | X | |  | Quantitative non-randomized study (pre-post design) [8] | Project to reduce the number of students admitted to schools who are under-immunized | |  | | |  | |  | | X | |  | |  | | X | Liaisons were trained to teach school nurses (training + questionnaire) | | | We cannot assess directly how much better the student body is protected as a result of the project. | C-IV  Moderate |
| DeSocio et al. (2006) [USA] [74] | |  | | X | |  | |  | |  | |  | Quantitative non-randomized study (pre-post design) [370] | Teaching children about mental health and illness: a school nurse health education program | | X | | |  | |  | |  | |  | |  | |  | A mental health education program (16-item test score) | | | Statistically significant improvements in their knowledge of mental health and mental illness. | E-IV  High |
| Dixon (2014) [UK] [75] | | X | |  | |  | |  | |  | |  | Qualitive (qualitative description) [n/a] | Teenage pregnancy intention and barriers that may prevent them from being identified | |  | | |  | |  | | X | |  | |  | |  | No intervention (n/a) | | | School nurses are encouraged to critically reflect on their perceptions of teenage parenting | D-II  Limited |
| Dodds (2011) [UK] [76] | |  | |  | |  | |  | | X | | X | Mixed Method design (convergent design) [19] | Attitudes of school nurses and teachers regarding the delivery of sex and relationship education (SRE) and sexual health services (SHS). | |  | | |  | |  | |  | |  | |  | | X | No intervention (qualitative and quantitative questionnaire) | | | Study has highlighted inconsistencies in how relationship education (SRE) and Sexual helaht service (SES) are implemented and prioritized in secondary schools in Nothingham. | C-V  Moderate |
| Doggett et al. (1992) [UK] [77] | |  | |  | |  | |  | | X | |  | Qualitative (phenomenology) [n/a] | Constraints and opportunities for school nurses’ future | |  | | |  | | X | |  | |  | |  | |  | No intervention (n/a) | | | The main keyword for school nurses’ future development must be flexibility | B-II  Limited |
| DoH (1999) [UK] [78] | |  | | X | |  | |  | |  | |  | Quantitative (narrative research) [n/a] | Health care in the UK | | X | | | X | |  | | X | |  | |  | | X | No intervention (n/a) | | | Health statistics in UK | A-II  Limited |
| Downie et al. (2002) [Australia] [79] | |  | |  | |  | |  | | X | |  | Qualitative (case study) [9] | Identification and description of the high school nurse | |  | | |  | | X | |  | |  | |  | |  | No intervention (diary + interview) | | | Findings highlight the complex and demanding work of high school nurses | B-II  Limited |
| Driscoll et al. (2015) [USA] [80] | |  | |  | |  | | X | |  | |  | Quantitative descriptive (survey) [435] | Are children with Typ-1 Diabetes safe at school? | |  | | | X | |  | | X | |  | |  | |  | No intervention (survey) | | | Most parents reported that schools had nurses available for the school day and they felt that their child was safe | C-III  Limited |
| Eisbach and Driessnack (2010) [USA] [81] | |  | |  | |  | |  | | X | | X | Qualitive (qualitative description) [23] | Reporting of child maltreatment by healthcare providers | | X | | | X | |  | |  | |  | |  | | X | No intervention (interviews) | | | Nurse’s knowledge and professional comfort level, stop to contextualize the assessment and to reflect on available services influenced the reporting decision. | C-II  Limited |
| Engelke et al. (2009) [USA] [82] | |  | |  | |  | |  | | X | |  | Qualitative (case study) [n/a] | Defining, delivering, and documenting the outcomes of case management by school nurses | |  | | |  | |  | |  | |  | |  | | X | No intervention (n/a) | | | Most school nurses need support, to provide case management and evaluate outcomes. | A-II  Limited |
| Engelke et al. (2008) [USA] [83] | | X | |  | |  | |  | |  | |  | Quantitative non-randomized study (pre-post design) [114] | School nurse Case management for children with chronic illness | |  | | | X | |  | | X | |  | |  | | X | Case management (questionnaires for students and self-reports by school nurse) | | | Children experienced an improvement in quality of life and gained skills and knowledge to manage their illness more effectively. | E-IV  High |
| Engelke et al. (2014) [USA] [84] | | X | |  | |  | |  | |  | |  | Quantitative non-randomized study (pre-post design) [143] | School nurse case management for students with asthma | |  | | | X | |  | |  | |  | |  | | X | Number of interventions by school nurses (questionnaires + academic achievements) | | | Huge complexity of case management of school nurses and goal attainment is related to the outcomes. | E-IV  High |
| Engelke et al. (2011) [USA] [85] | | X | |  | |  | |  | |  | |  | Qualitative (qualitative description) [86] | Care provided to children with diabetes by school nurses using case management | |  | | | X | |  | |  | |  | |  | | X | School nurses were asked to be proactive, to intervene at least weekly, and to focus on responding to problems and also on self-management; teaching school personnel (questionnaire) | | | School nurses are effective in using case management to enhance the health and well-being of children with diabetes. | E-II  Limited |
| Engh Kraft et al. (2017) [Sweden] [86] | |  | |  | |  | |  | | X | |  | Qualitative (grounded theory method) [23] | Ability of the school nurses to detect and support sexually abused children | |  | | |  | |  | | X | |  | |  | |  | No intervention (focus groups) | | | The main concern of the school nurses was support and assistance to children who are sexually abused | C-II  Limited |
| Engh Kraft et al. (2017) [Sweden] [87] | |  | |  | |  | |  | | X | |  | Qualitative (grounded theory) [23] | Explore the ability of the school nurses to detect and support sexually abused children | |  | | |  | |  | | X | |  | |  | |  | No intervention (secondary analysis of focus groups) | | | School nurses avoided addressing child sexual abuse (CSA) due to arousal of strong emotions, ambivalence, and a complicated disclosure process | C-II  Limited |
| Fagan (1995) [UK] [88] | |  | |  | |  | |  | | X | |  | Qualitative (qualitative description) [73] | Investigate school nurses’ perceptions of their contributions towards ‘health of the nation’ targets | |  | | |  | |  | | X | |  | |  | |  | No intervention (group interviews, individual interviews) | | | School nurses function well and have potential to affect health positively, however, there are four main areas of constraint | C-II  Limited |
| Ferson et al. (1995) [Australia] [89] | |  | | X | |  | |  | |  | |  | Quantitative randomized controlled trials [239] | Immunisation uptake in school entrants | |  | | | X | |  | | X | |  | |  | |  | Passive intervention: letter to parents; Active intervention: letter + phone call to parents (surveyed by telephone) | | | Higher rates of immunization in active intervention group | E-V  High |
| Few (1996) [UK] [90] | |  | |  | | X | |  | | X | |  | Quantitative descriptive (survey) [43 teachers, 27 school nurses] | Alliances in school sex education between teachers and school nurses | |  | | |  | |  | | X | | X | |  | |  | Joint training event (questionnaire, semi-structured interviews) | | | Teachers have positive perceptions on school nurse’s role, limitation of school nurses’ current practice, training needs for both groups | C-III  Moderate |
| Foster and Keele (2006) [USA] [91] | |  | | X | |  | |  | |  | |  | Quantitative non-randomized study (pre-post design) [approx. 10,000] | Implementing an Over-the-Counter medication administration policy in an elementary school | |  | | |  | |  | |  | |  | | X | |  | Over-the-Counter medication by school nurses (sent home rates) | | | Not significant findings but intervention shows that fewer students are sent home | E-IV  High |
| France (2013) [UK] [92] | | X | |  | |  | |  | | X | |  | No study design | New texting service for teenagers has all-round benefits | |  | | |  | |  | |  | |  | |  | | X | No intervention (n/a) | | | A school nurse’s perspective helping scores of children | A-I  Limited |
| Francisco et al. (2017) [USA] [93] | | X | |  | |  | |  | | X | |  | Quantitative non-randomized study (pre-post design) [54 school nurses and 178 children] | Improve asthma control among children by increasing the competency of school nurses | |  | | | X | | X | |  | |  | |  | |  | School nurse education (school nurse check-ups) | | | Students’ outcomes improved, and health care utilization costs declined | E-IV  High |
| Fryer Jr and Igoe (1995) [USA] [94] | |  | |  | |  | |  | |  | | X | Quantitative descriptive (survey) [482 districts] | Relationship between availability of school nurses and well-being | |  | | |  | |  | | X | |  | |  | |  | No intervention (survey) | | | School nurses were rated very competent in their performance but considered underutilized in non-clinical areas | C-III  Moderate |
| Fryer Jr and Igoe (1996) [USA] [95] | |  | |  | |  | |  | | X | | X | Quantitative descriptive (prevalence study without comparison group) [482] | Functions of school nurses and health assistance | |  | | |  | |  | |  | |  | |  | | X | No intervention (questionnaire) | | | While use of health assistants increased the past three years, school nurses were not being displaced. | B-III  Limited |
| Gaffrey and Bergren (1998) [USA] [96] | |  | |  | |  | |  | | X | |  | No study design | Information for health care providers is needed. | |  | | |  | |  | | X | | X | |  | |  | No intervention (literature, outcomes of national summit) | | | Inclusion of the school nurse promotes effective, timely, accessible and cost-effective services for children | A-I  Limited |
| Garwick et al. (2015) [USA, Iceland] [97] | |  | |  | |  | |  | | X | |  | Qualitative (qualitative description) [32] | Comparison of school nurses in Iceland and the USA regarding asthma care, development of care coordination model | |  | | | X | |  | |  | |  | |  | | X | No intervention (n/a) | | | Similar asthma care coordination process, high levels of complexity and diversity in approaches | C-II  Limited |
|  | |  | |  | |  | |  | |  | |  |  |  | |  | | |  | |  | |  | |  | |  | |  |  | | |  |  |
| Gilman et al. (1979) [USA] [98] | |  | |  | |  | |  | | X | |  | Quantitative descriptive (case series) [41] | Task differentiation among elementary, middle and high school nurses | |  | | |  | |  | | X | |  | |  | |  | No intervention (self-report) | | | Nurse activities differ significantly, time spent by nurse in direct contact with students increases with increasing school levels | B-III  Limited |
| Gordon et al. (2007) [USA] [99] | | X | |  | |  | |  | |  | |  | Quantitative non-randomized study (pre-post design) [227] | Impact of a tertiary care center special needs program | |  | | | X | |  | |  | |  | |  | | X | Care coordination provided by nurse case manager, sometimes with physician (tertiary care center resource utilization, charges, payments) | | | Significant decrease in number of hospitalizations and payments, therefore improved health care and reduced costs | B-IV  Limited |
| Gottfried (2012) [USA] [100] | |  | | X | |  | |  | |  | |  | Quantitative descriptive (case series) [674] | Which school-level factors contribute to school quality | |  | | |  | |  | |  | |  | | X | |  | No intervention (students’ achievement scores, school budget data) | | | School quality higher in schools with e.g., music and language programs. | A-III  Limited |
| Grandahl et al. (2016) [Sweden] [101] | |  | | X | |  | |  | |  | |  | Quantitative randomized controlled trial [741] | Improving primary prevention of Human papillomavirus HPV infection | |  | | | X | |  | | X | |  | |  | |  | School nurse-delivered face-to-face structured information about HPV and prevention (questionnaire) | | | Intervention had favorable effects on beliefs about primary prevention of HPV and increased HPV vaccination rates | E-V  High |
|  | |  | |  | |  | |  | |  | |  |  |  | |  | | |  | |  | |  | |  | |  | |  |  | | |  |  |
| Grudnikoff et al. (2015) [USA] [102] | |  | |  | |  | |  | |  | | X | Quantitative descriptive (case series) [551 psychiatric Emergency Department (ED) evaluations] | Characteristics and disposition of youth referred from schools for emergency psychiatric evaluation | |  | | |  | |  | |  | |  | |  | | X | No intervention (retrospective analysis of ED transferals) | | | In-school screening, which occurred infrequently, reduced unnecessary evaluations by 52 %. | C-III  Moderate |
| Guttu et al. (2004) [USA] [103] | |  | |  | |  | |  | | X | |  | Quantitative descriptive (prevalence study) [57 counties] | Examination of nurse-to-student ratio | | X | | | X | |  | | X | |  | |  | |  | No intervention (self-report) | | | Better nurse-to-student ratios are related to better services for children with diabetes and asthma | C-III  Moderate |
| Hackett (2013) [UK] [104] | |  | |  | |  | |  | | X | |  | Qualitative (phenomenology) [6] | School nurses’ perceptions of their role in child protection, identify training needs | |  | | | X | |  | | X | |  | |  | |  | No intervention (semi-structured interviews) | | | Perceived confusion and lack of clarity regarding school nurse’s role and involvement and child protection | B-II  Limited |
| Halterman et al. (2011) [USA] [105] | | X | |  | |  | |  | |  | |  | Quantitative randomized controlled trial [530] | Impact of a school-based asthma therapy trial on asthma symptoms among urban children with persistent asthma | |  | | | X | |  | |  | |  | |  | |  | Preventive asthma medication administration, directly observed by school nurses (mean number of symptom-free days by survey) | | | Intervention group significantly more symptom-free days compared to control group | E-V  High |
| Hanson et al. (2013) [USA] [106] | |  | |  | |  | |  | | X | |  | Quantitative descriptive (survey) [65] | School nurses’ responses to the electronic exchange of the Asthma Action Plan, value and efficiency of Asthma Action Plan (AAP) | |  | | | X | |  | |  | |  | |  | |  | Implementation of a portal designed for electronic exchange of the AAP between providers and schools (survey) | | | AAP enables more efficient management of asthma and increases school nurses’ self-efficacy | A-III  Limited |
| Harrell et al. (1999) [USA] [107] | |  | | X | |  | |  | |  | |  | Quantitative randomized controlled trial [2,109] | Determine effects of interventions to reduce cardiovascular disease risk factors in children | |  | | | X | | X | | X | |  | |  | |  | Classroom-based intervention for all participants, risk-based intervention for those with risk factors (nutrition, physical activity, smoking prevention) (physiologic test) | | | Physical activity in risk-based group and post-test knowledge in classroom-based group significantly higher than control, reduced cholesterol and body fat levels | D-V  High |
| Harrington et al. (2018) [USA] [108] | | X | |  | |  | |  | |  | |  | Quantitative randomized controlled trial [46] | Nurse-based intervention to create adherence to inhaled corticosteroids among children with asthma | |  | | | X | |  | |  | |  | |  | |  | Intervention group received Inhaled corticosteroids (ICS) every morning at school over 60-day treatment period, control group administered ICS at home (survey, interview) | | | Intervention patients reported significantly less functional limitation, adjustment to family life and sleep loss, no differences in unscheduled health-care utilization | E-V  High |
| Hawkins et al. (1994) [USA] [109] | |  | |  | |  | |  | | X | |  | Qualitative (ethnography) | Examine the evolution of school nursing | |  | | |  | |  | | X | |  | |  | |  | No intervention (written materials by school nurses, journals, books, newspapers) | | | Importance of the school nurse currently increases again | A-II  Limited |
| Hawthorne et al. (2011) [USA] [110] | | X | |  | |  | |  | |  | |  | Quantitative non-randomized study (pre-post design) [1,074] | Decrease incidence of childhood overweight with a walking program | |  | | | X | |  | | X | |  | |  | |  | Walking program 3 days a week for 16 weeks (BMI, waist circumference, cardio-respiratory) | | | No significant changes in BMI or waist circumference, but significant changes in cardio-respiratory fitness | D-IV  High |
| Hayes-Bohn et al. (2004) [USA] [111] | | X | |  | |  | |  | |  | |  | Qualitative (phenomenology) [30] | Opinions, concerns and recommendations regarding care of Type 1 diabetes in schools | |  | | | X | |  | |  | |  | |  | |  | No intervention (semi-structured interviews) | | | Expressed concerns about limited knowledge of diabetes in school personnel, limited healthy food options | B-II  Limited |
| Hayter et al. (2012) [UK] [112] | |  | |  | |  | |  | | X | |  | Qualitative (phenomenology) [51] | Experiences of clinicians / managers regarding school-based sexual health clinics | |  | | |  | |  | | X | | X | |  | |  | No intervention (interview) | | | Importance of involving young people in services, dealing with objections, building alliances with school staff | A-II  Limited |
| Hellems and Clarke (2007) [USA] [113] | | X | |  | |  | |  | |  | |  | Quantitative descriptive (survey) [185] | Determine which school personnel assist type 1 diabetic students | |  | | | X | |  | |  | |  | |  | |  | No intervention (survey with parents) | | | Type 1 diabetes students can be cared for by a variety of trained medical and nonmedical personnel | C-III  Moderate |
|  | |  | |  | |  | |  | |  | |  |  |  | |  | | |  | |  | |  | |  | |  | |  |  | | |  |  |
| Hendershot et al. (2006) [USA] [114] | |  | |  | |  | |  | | X | |  | Quantitative descriptive (survey) [404] | Elementary school nurses’ perceptions of student bullying | | X | | |  | |  | | X | |  | |  | |  | No intervention (survey) | | | School nurses are effective in reducing school bullying however, not supported by research | A-III  Limited |
| Hendershot et al. (2008) [USA] [115] | |  | |  | |  | |  | | X | |  | Quantitative descriptive (survey) [2,629] | Elementary school nurses’ perceptions regarding BMI of students with and without mandated BMI policies | |  | | | X | |  | | X | |  | |  | |  | No intervention (survey) | | | Mandates have a positive influence on school nurses and their measurement of BMI and increases the likelihood that nurses will measure BMI | A-III  Limited |
| Henry (1997) [USA] [116] | |  | |  | |  | |  | | X | |  | Quantitative (narrative research) [n/a] | Addressing national issues and priorities for school nursing services. | |  | | |  | |  | | X | |  | |  | | X | No intervention (n/a) | | | The program is designed to grow and to be as flexible as the students` needs require. | B-II  Limited |
| Hill and Hollis (2012) [USA] [117] | |  | |  | | X | |  | | X | |  | Quantitative descriptive (survey) [442 teachers, 7 nurses] | Elementary school teacher time spent on student health issues and relationship to school nurse services | |  | | |  | |  | | X | | X | | X | |  | No intervention (survey with traditional and special needs classroom teachers) | | | With school nurse presence there are fewer early releases, increased communication, less time spent on health issues, more safety, more health information | C-III  Moderate |
| Houck et al. (2002) [USA] [118] | | X | |  | |  | |  | |  | |  | Quantitative non-randomized study (pre-post design) [12] | School-based support groups for depressed adolescents | | X | | |  | |  | | X | |  | |  | |  | Cognitive–behavioural intervention and a school-based skill-building intervention. (questionnaire) | | | 55% decrease in suicidal ideation, a 27% decrease in perceived stress, and a 26% decrease in family distress | E-IV  High |
| Houghton et al. (1992) [UK] [119] | |  | | X | |  | |  | |  | |  | Quantitative descriptive (incidence study) [82] | Impact of applying selection criteria for medical examination at school entry | |  | | |  | |  | | X | |  | |  | |  | Medical examination (medical information) | | | System where school nurse is responsible for examination is preferable | D-III  Moderate |
| Hoying and Melnyk (2016) [USA] [120] | | X | |  | |  | |  | |  | |  | Quantitative non-randomized study (pre-post design) [31] | Pilot Study with urban-dwelling minority youth to improve physical activity and mental health outcomes | | X | | | X | |  | | X | |  | |  | |  | COPE and Healthy Lifestyles TEEN (Healthy Lifestyle Beliefs Scale, OMRONTM pedometer, The Beck Youth Inventory) | | | The COPE program is a promising intervention that can improve physical and mental health outcomes | D-IV  High |
| Igoe (1994) [USA] [121] | |  | |  | |  | |  | | X | |  | Qualitative (narrative research) [n/a] | School nurse developments and framework for school nurse practice | |  | | |  | |  | |  | |  | |  | | X | No intervention (n/a) | | | School nurses design and implement innovative health education programs that foster a healthy and safe climate at school | B-II  Limited |
|  | |  | |  | |  | |  | |  | |  |  |  | |  | | |  | |  | |  | |  | |  | |  |  | | |  |  |
| Izquierdo et al. (2009) [USA] [122] | | X | |  | |  | |  | |  | |  | Quantitative randomized controlled trial [41] | Feasibility and effectiveness of telemedicine for type 1 diabetes children | |  | | | X | |  | |  | |  | |  | |  | Usual care and telemedicine unit (videoconference) (haemoglobin A1c levels, paediatric life quality) | | | Haemoglobin (A1c) values decreased in telemedicine cohort and life quality of significantly improved | E-V  High |
| Janevic et al. (2016) [USA] [123] | | X | |  | |  | |  | |  | |  | Quantitative non-randomized study (pre-post design) [805] | Effect of care coordination on asthma outcomes among children in underserved urban communities | |  | | | X | |  | |  | |  | |  | | X | Medical-social care coordination program (survey with parents regarding asthma symptoms and health care utilization) | | | Care coordination may improve paediatric asthma symptom control and reduce emergency department visits | D-IV  High |
| Johansson and Ehnfors (2006) [Sweden] [124] | |  | | X | |  | |  | |  | |  | Quantitative (Qualitative description) [26] | Mental health-promoting dialogue of school nurses from the perspective of adolescent pupils | | X | | |  | |  | |  | |  | |  | |  | No intervention (interviews, focus groups) | | | The findings show that the prerequisites for a mental health-promoting dialogue with the school nurse include content and setting. | C-II  Limited |
| Johnston et al. (2013a) [USA] [125] | |  | | X | |  | |  | |  | |  | Quantitative randomized controlled trial [835] | Impact of a school-based paediatric obesity prevention program | |  | | | X | |  | | X | |  | | X | |  | Professional-facilitated intervention**,** promotion of seven healthy messages with multiple classroom interventions (weight: BMI, academic: Grade Point average (GPA) | | | Obesity prevention program was effective in reducing zBMI compared to self-help condition | D-IV  High |
| Johnston et al. (2013b) [USA] [126] | | X | |  | |  | |  | |  | |  | Quantitative randomized controlled trial [71] | Outcomes of a school-based intensive lifestyle weight management program | |  | | | X | |  | | X | |  | |  | |  | Instructor-led intervention to modify eating and physical activity behaviours (BMI) | | | Instructor lead intervention (ILI) participants showed significantly greater decreased in zBMI compared with self-help controls | D-V  High |
| Jones and McEwen (2012) [UK] [127] | | X | |  | |  | |  | |  | |  | Qualitative (study not completed) [n/a] | Impact of second-hand smoke exposure and a training module for school nurses | |  | | |  | | X | | X | |  | |  | |  | Training module for school nurses to help parents create smoke-free homes (ongoing) | | | Training modules can help school nurses to intervene effectively with smoke exposure | C-II  Limited |
| Jordan et al. (2017) [USA] [128] | |  | |  | |  | |  | | X | |  | Quantitative non-randomized study (pre-post design) [174] | Effectiveness of educational intervention for school nurses regarding children at risk of maltreatment | |  | | |  | | X | | X | |  | |  | |  | Education intervention for all school nurses regarding child maltreatment (survey) | | | 10% increase in knowledge, significant increase in self-efficacy regarding child maltreatment | C-IV  Moderate |
| Kelly et al. (2005) [UK] [129] | | X | |  | |  | |  | |  | |  | Qualitative (phenomenology) [27] | Views on nurse-led service to address challenging behaviour in children and prevent exclusion | | X | | |  | |  | |  | |  | | X | |  | Implemented service from prior studies (interview) | | | Positive changes for children and their families | C-II  Limited |
|  | |  | |  | |  | |  | |  | |  |  |  | |  | | |  | |  | |  | |  | |  | |  |  | | |  |  |
| Kemper et al. (2012) [USA] [130] | |  | | X | |  | |  | |  | |  | Quantitative descriptive (case series) [2,726] | Statewide school nurse vision screening program | |  | | |  | |  | | X | |  | | X | |  | Screening program (screening results) | | | Identification of 3 cases for every 100 children screened, receiving contact lenses | E-III  Moderate |
| Khubchandani et al. (2013) [USA] [131] | |  | |  | |  | |  | | X | |  | Quantitative descriptive (survey) [750] | Providing assistance to the victims of adolescent dating violence | |  | | |  | | X | | X | |  | |  | |  | No intervention (survey) | | | Nurses appear to need more training in Adolescent dating violence (ADV). There are a number of barriers in helping student victims of ADV. | B-III  Limited |
| Kim et al. (2015) [USA] [132] | |  | | X | |  | |  | | X | |  | Qualitative (Qualitative description) [6 School Nurses and 25 adolescents] | Connecting Students to Mental Health Care | | X | | |  | |  | |  | |  | |  | | X | Engagement protocol (different questionnaires and focus groups) | | | Small increase in school nurses’ use of Engagement Protocol (EP) and in adolescents’ readiness for services following training. | C-II  Limited |
| Kimel (1996) [USA] [133] | |  | | X | |  | |  | |  | |  | Quantitative non-randomized study (pre-post design) [199] | Handwashing program for elementary students by school nurses | |  | | |  | |  | | X | |  | | X | |  | Surveying teachers, inspecting handwashing facilities, providing classroom presentations (absenteeism records) | | | Significant decrease in absenteeism for illness | E-IV  High |
|  | |  | |  | |  | |  | |  | |  |  |  | |  | | |  | |  | |  | |  | |  | |  |  | | |  |  |
| Kirchofer et al. (2007) [USA] [134] | |  | |  | |  | | X | |  | |  | Quantitative descriptive (survey) [369] | Parents’ perceptions regarding the importance of school health services | |  | | |  | |  | | X | | X | |  | |  | No intervention (survey) | | | Parents were mostly supportive of school nurses and should be perceived as allies in ensuring job security | C-III  Moderate |
| Knauer et al. (2015) [USA] [135] | |  | |  | |  | |  | |  | | X | Qualitative (qualitative description) [31] | Role of public schools in supporting CSHCN | |  | | | X | |  | |  | | X | |  | | X | No intervention (interview) | | | Possible improvement opportunities in increased funding, monitoring and of school health services | B-II  Limited |
| Kornguth (1991) [USA] [136] | |  | | X | |  | |  | |  | |  | Qualitative (narrative research) [n/a] | Gap of health information regarding school-aged children, summarized research findings | |  | | |  | | X | |  | |  | | X | |  | No intervention (n/a) | | | Small health problems can interfere with learning, immunization programs need to be maintained, specific plans for each district necessary | A-II  Limited |
| Krenitsky-Korn (2011) [USA] [137] | | X | |  | |  | |  | |  | |  | Mixed method design (convergent design) [57] | Compare relationship between students with asthma to students without asthma in various fields | |  | | | X | |  | | X | |  | | X | | X | No intervention (survey, attendance records, academic achievement) | | | The school nurse as a case manager, provider of treatments, and facilitator for the process of sports participation plays an important role for students with asthma | C-III  Moderate |
| Kroshus (2015) [USA] [138] | |  | |  | |  | |  | | X | |  | Quantitative descriptive (survey) [370] | Assessing the awareness and behaviors of U.S. high school nurses with respect to the female athlete triad | | X | | |  | |  | |  | |  | |  | | X | No intervention (survey) | | | Results indicate that only 19% of the nurses were able to identify the three components of the Triad | C-III  Moderate |
| Krug et al. (1997) [USA] [139] | |  | |  | |  | |  | | X | |  | Quantitative randomized controlled trial [3,899] | Effectiveness of an elementary school-based violence prevention program | |  | | |  | |  | | X | |  | |  | |  | Violence prevention program (weekly number of nurse visits) | | | Children were visiting the nurse less often following the implementation of the program | D-V  High |
| Lamb et al. (1998a) [USA] [140] | | X | |  | |  | |  | |  | |  | Quantitative non-randomized study (pre-post design) [9] | Evaluation of access, recruitment and retention of pregnant African American teens for a subsequent smoking cessation intervention program | |  | | |  | | X | | X | |  | |  | |  | Eight group sessions (saliva samples, self-report smoking history) | | | Out of a clinic and a school site to assess the factors, only the school site was successful | D-IV  High |
| Lamb et al. (1998b) [USA] [141] | | X | |  | |  | |  | |  | |  | Quantitative randomized controlled trial [40] | Program to help high school students with depressive symptoms to effectively cope | | X | | |  | |  | |  | |  | |  | |  | Treatment with nurse-led, 8-week cognitive skills group, conducted at school (survey) | | | Reduced depressive symptoms in females and wider range of coping compared with controls, no effect for male participants | E-V  High |
| Land and Barclay (2008) [Australia] [142] | |  | |  | |  | |  | | X | |  | Qualitative (qualitative description) [10] | Nurses’ contribution to child protection | |  | | |  | | X | |  | |  | |  | |  | No intervention (semi-structured open-end questions) | | | Organizational structure, institutional practices and legislative frameworks influenced nurses’ effectiveness | B-II  Limited |
| Larsson and Carlsson (1996) [Sweden] [143] | | X | |  | |  | |  | |  | |  | Quantitative randomized controlled trial [26] | Efficacy of a school-based, nurse-administered relaxation training for children with chronic tension-type headache | |  | | | X | |  | | X | |  | |  | |  | Ten 20-minute sessions to train relaxation techniques (survey, headache diary, parents’ records of children’s headaches) | | | More reduction in headache activity in intervention group than in control group, clinically significant headache improvements | E-V  High |
| Lazdowsky et al. (2016) [USA] [144] | |  | | X | |  | |  | | X | |  | Study 1: Quantitative descriptive (survey) [109 children, 5 nurses]; Study 2: Quantitative descriptive (survey) [27] | Assessment, development, and implementation of an educational guide for school nurses regarding chronic headaches in children and adolescents | |  | | | X | | X | |  | | X | |  | | X | “Headache Tools to Stay in School” training for school nurses (surveys and interviews on practices prior to intervention, survey after intervention, weekly number of school nurse visits) | | | “Headache Tools to Stay in School” is a useful resource in facilitating interdisciplinary communication to meet the needs of students with headaches | C- III  Moderate |
| Lee and Kubik (2015) [USA] [145] | |  | |  | |  | | X | |  | |  | Quantitative descriptive (survey) [122] | Parent’s response to a school-based BMI screening and parent notification program | |  | | | X | |  | | X | |  | |  | |  | School-based body mass index screening and parent notification program (questionnaire) | | | Low-intensity intervention can support overweight children, and their parents. | C-III  Moderate |
| Leff and Bennett (1996) [UK] [146] | |  | |  | |  | |  | | X | | X | Quantitative non-randomized study (cross-sectional) [11 doctors, 26 school nurses] | Maximize efficient use of health personnel at school entry assessment | |  | | |  | |  | |  | | X | |  | | X | Introducing nurse assessments (record decision making process and outcomes) | | | Number of children referred to other services did not change, but proportion of children whose needs were discussed with headteacher was reduced | C-IV  Moderate |
| Lehmkuhl and Nabors (2008) [USA] [147] | | X | |  | |  | |  | |  | |  | Quantitative descriptive (survey) [58] | Children’s perceptions of their satisfaction with support from school nurses, teachers and classroom friends regarding type 1 diabetes | |  | | | X | | X | |  | |  | |  | | X | No intervention (survey, diabetes measures (e.g., Glycosylated haemoglobin (HbA1c) levels)) | | | Children were satisfied with their support at school, but would like more support during after-school activities, higher satisfaction correlated with lower HbA1c levels | C-III  Moderate |
| Levy et al. (2006) [USA] [148] | | X | |  | |  | |  | |  | |  | Quantitative randomized controlled trial [243] | Effectiveness of a school-based asthma case management with medically underserved children | |  | | | X | |  | |  | |  | | X | | X | Nurse-led asthma case management: weekly group sessions, care coordination (school absences, hospital utilization, asthma knowledge and skills) | | | Intervention group had fewer school absences and significantly fewer emergency department visits and fewer hospital days after intervention | E-V  High |
|  | |  | |  | |  | |  | |  | |  |  |  | |  | | |  | |  | |  | |  | |  | |  |  | | |  |  |
| Lewis et al. (1992) [USA] [149] | | X | |  | |  | |  | |  | |  | Qualitative (case study) [n/a] | Care management for children who are medically fragile/technology dependent. | |  | | |  | |  | |  | |  | |  | | X | No intervention (n/a) | | | Care management has received more attention as the number of children with special health-care needs has increased. | C-II  Limited |
| Liberatos et al. (2013) [USA] [150] | |  | |  | |  | |  | | X | |  | Quantitative descriptive (survey) [44] | Barriers to asthma management by school nurses in higher-risk school district and access to which extend National Asthma Education and Prevention Program (NAEPP) recommendations are followed | |  | | | X | | X | |  | | X | |  | |  | No intervention (survey) | | | Nurses learned of children with asthma mainly through school records and students with symptoms rather than through parents | C-III  Moderate |
| Lightfoot and Bines (2000) [UK] [151] | |  | |  | | X | |  | | X | | X | Quantitative (quantitative description) [78] | Working to keep school children healthy: the complementary roles of school staff and school nurses | |  | | |  | | X | |  | | X | |  | |  | No intervention (semi-structured interviews and focus groups) | | | To optimize school nurses' contribution to child health, a more strategic approach is required, along with research into the effectiveness of school nursing interventions. | C-III  Moderate |
|  | |  | |  | |  | |  | |  | |  |  |  | |  | | |  | |  | |  | |  | |  | |  |  | | |  |  |
| Lightfoot and Bines (1998) [UK] [152] | |  | |  | |  | |  | | X | |  | Qualitative (qualitative description) [105 interviews, 7 discussion groups] | Generate information on nurses’ role in meeting health needs of children | |  | | |  | | X | | X | | X | |  | |  | No intervention (semi-structured interviews, discussion groups) | | | Four key issues: needs-led service, using limited resources effectively, appropriate setting for nursing work | C-II  Limited |
| Lindeke et al. (2002) [USA] [153] | |  | |  | |  | |  | | X | |  | Qualitative (case study) [n/a] | Family-centered care coordination for children with special needs across multiple settings | |  | | |  | |  | |  | |  | |  | | X | No intervention (n/a) | | | Paediatric nurse practitioners (PNPs) may act as change agents to infuse family-centered care principles into existing and future care coordination models. | A-II  Limited |
| Liptzin et al. (2016) [USA] [154] | | X | |  | |  | |  | |  | |  | Quantitative non-randomized study (pre-post design) [252] | School centered asthma program: Step-Up Asthma Program | |  | | |  | | X | | X | |  | |  | | X | Step-Up Asthma program (asthma knowledge, inhaler technique, number of asthma exacerbations) | | | A guideline-based school-centered asthma program can significantly reduce asthma morbidity | D-IV  High |
| Long et al. (1975) [USA] [155] | | X | |  | |  | |  | |  | |  | Quantitative randomized controlled trial [302] | Nursing intervention program directed towards high absence school children | |  | | |  | |  | |  | |  | | X | |  | Usual nurse care and, additionally, high absence pupils received focused attention from the nurse (attendance records) | | | Intervention group showed a statistically significant difference in absence decline | E-V  High |
| Looman et al. (2013) [USA] [156] | | X | |  | |  | |  | |  | |  | Quantitative randomized controlled trial [94] | Care coordination for children with complex special health care needs: TeleFamilies study | |  | | | X | |  | |  | |  | |  | | X | TeleNursing and Advanced Practice Nurse (APN) care coordination (number of encounters via telephone, webcam, chart communication, fax, in the clinic or hospital setting) | | | Having an APN care coordinator can potentially decrease fragmentation of care and improving the efficiency of care delivered by the team. | A-V  Limited |
| Lunney (1996) [USA] [157] | |  | |  | |  | |  | | X | |  | Qualitative (narrative) [n/a] | Significance of nursing classification systems to school nursing | |  | | |  | |  | | X | | X | |  | |  | No intervention (n/a) | | | Nursing classification systems facilitate the development of a national computer database for nursing practice | A-II  Limited |
| Lunney et al. (1997) [USA] [158] | |  | |  | |  | |  | | X | |  | Quantitative descriptive (survey) [490] | Describe the relevance of nursing diagnoses approved by NANDA | |  | | |  | | X | | X | |  | |  | |  | No intervention (survey) | | | NANDA and health promotion diagnoses are relevant to school nursing | A-III  Limited |
| Lunstead (2016) [USA] [159] | |  | |  | |  | |  | | X | |  | Quantitative descriptive (survey) [144] | Screening and brief intervention in high schools: school nurses' practices and attitudes | |  | | |  | |  | | X | |  | |  | |  | No intervention (questionnaire) | | | Implementation of SBIRT that focuses on standardized, annual screening has the potential to deliver high-quality care in this setting. | C-III  Moderate |
| Luthy et al. (2011) [USA] [160] | |  | | X | |  | |  | |  | |  | Quantitative non-randomized study (pre-post design) [958] | Educational and incentive program in elementary schools to increase immunization compliance rates | |  | | |  | | X | | X | |  | |  | |  | 4-week nurse-led intervention program with weekly classes (compliance rates) | | | Intervention did not improve compliance rates significantly | E-IV  High |
| Lynch (2008) [UK] [161] | |  | | X | |  | |  | |  | |  | No study design | School nurse service addressing health challenges among students | |  | | |  | | X | | X | |  | |  | |  | Center for students to talk confidentially, test for pregnancy, provide contraception (n/a) | | | The center is welcomed by school staff, students and parents | E-I  Limited |
| Magalnick (2008) [USA] [162] | |  | | X | |  | |  | |  | |  | Qualitative (case study) [n/a] | Role of the school nurse in providing school health services | |  | | |  | | X | | X | |  | |  | |  | No intervention (n/a) | | | The American Academy of Paediatrics (AAP) supports having a full-time school nurse in every school to ensure a strong connection with each student’s medical home. | C-II  Limited |
| Magee et al. (2012) [USA] [163] | |  | | X | |  | |  | |  | |  | Quantitative descriptive (prevalence study without comparison group) [437] | Efficacy of and advocacy for postural screening in public schools | |  | | | X | |  | | X | |  | |  | |  | Postural screening (screening performance + questionnaire) | | | Poor efficacy for a postural screening program. However, nurses surveyed reported that it was a valuable health screening tool. | E-III  Moderate |
| Major et al. (2006) [USA] [164] | |  | |  | |  | |  | | X | |  | Qualitative (qualitative description) [32] | Providing asthma care in elementary schools | |  | | |  | | X | |  | |  | |  | |  | No intervention (focus groups) | | | 4 barriers identified: lack of education, lack of communication, lack of resources, and lack of respect. | B-II  Limited |
| Maunder (2004) [UK] [165] | |  | |  | |  | |  | | X | |  | No study design | A typical day in the life of a school nurse | |  | | |  | | X | |  | |  | |  | |  | No intervention (n/a) | | | No outcome | B-I  Limited |
| McKaig et al. (1984) [USA] [166] | |  | |  | |  | |  | | X | |  | Qualitative (case study) [16] | Implementation of the School Nurse Practitioner Role | |  | | |  | | X | |  | |  | |  | |  | No intervention (questionnaire for a structured interview) | | | Role change and boundary encroachment were the main barriers identified. | B-II  Limited |
| Medaglia et al. (2013) [USA] [167] | | X | |  | |  | |  | |  | |  | Quantitative descriptive (survey) [approx. 2,000 schools each year: total 5 years] | School-based asthma surveillance program | |  | | | X | |  | |  | |  | |  | |  | No intervention (survey) | | | Evidence that a school-based paediatric asthma surveillance system can be developed and successfully implemented. | B-III  Limited |
| Melin and Lenner (2009) [Sweden] [168] | | X | |  | |  | |  | |  | |  | Quantitative non-randomized study (cohort study) [20] | Prevention of further weight gain in overweight school children | |  | | | X | |  | |  | |  | |  | |  | Dietary and lifestyle advice and support by school nurses monthly for one year (questionnaire) | | | Overweight progression can modify by increased awareness of the problem of their food habits and lifestyle practices. | E-IV  High |
| Mickel et al. (2016) [USA] [169] | | X | |  | |  | |  | |  | |  | Quantitative non-randomized study (pre-post design) [147] | Evaluation of a school-based asthma education protocol | |  | | | X | |  | |  | |  | |  | |  | Iggy - asthma education video, poster, comic book, sticker, and trading card program (test questions based on Iggy content) | | | Asthma knowledge increased significantly and retained after one month follow-up | E-IV  High |
| Moricca et al. (2013) [USA] [170] | |  | | X | |  | |  | |  | |  | Quantitative non-randomized study (pre-post design) [Gr.1: non-responsive n=26; Gr.2: (Case Manag. (CM) n=40; Gr.3: control n=76] | School asthma screening and case management: attendance and learning | |  | | |  | |  | |  | |  | | X | | X | Screening and case Management for children with asthma (questionnaires, academic testing and attendance rates) | | | School nurse screening, CM, and collaboration with a medical provider resulted in early identification, referral, and treatment of students at risk for asthma | E-IV  High |
| Morris et al. (2011) [USA] [171] | |  | |  | |  | |  | | X | |  | Quantitative (survey) [171] | Preparedness for students and staff with anaphylaxis | |  | | | X | | X | | X | |  | |  | |  | No intervention (survey) | | | 30 percent of school nurses reported using 1 student’s prescribed medication to rescue another student in distress. | B-III  Moderate |
| Morrison-Sandberg et al. (2011) [USA] [172] | |  | |  | |  | |  | | X | |  | Qualitative (case study) [21] | Obesity prevention practices of elementary school nurses | |  | | | X | | X | | X | |  | |  | |  | No intervention (semi-structured interviews) | | | School nurses are well positioned to provide prevention services that will contribute to reducing childhood obesity | B-II  Limited |
| Morton and Schultz (2004) [UK] [173] | |  | | X | |  | |  | |  | |  | Quantitative randomized control trials (cross-over design) [253] | Alcohol gel as an adjunct to handwashing in reducing absenteeism secondary to infectious illness | |  | | |  | |  | | X | |  | |  | |  | 45-minute ‘‘Germ Unit’’ was taught in experimental group and a standard unit on hand hygiene was taught in control group. | | | Alcohol gel as an adjunct to handwashing was shown to be effective in reducing absenteeism due to infectious illness by 43%. | E-V  High |
| Muggeo et al. (2017) [USA] [174] | | X | |  | |  | |  | |  | |  | Quantitative non-randomized study (pre-post design) [253] | School nurse-delivered intervention for anxious children | | X | | |  | |  | |  | |  | |  | |  | CALM (Training Satisfaction and Feedback Questionnaire; Session Summary Form; Intervention Fidelity Form; CALM Satisfaction Form; Exit Interview) | | | Outcome measures revealed significant reductions in anxiety, somatic symptoms, and concentration problems | E-IV  High |
| Murray (2008) [USA] [175] | |  | |  | |  | |  | | X | |  | Qualitative (case study) [n/a] | The role of the school nurse in providing school health services | |  | | |  | | X | |  | |  | |  | |  | No intervention (n/a) | | | Recommendations are offered to facilitate the working relationship between the school nurse and the child’s medical home. | A-II  Limited |
| Nauta et al. (2009) [USA] [176] | |  | |  | |  | |  | | X | |  | Quantitative descriptive (survey) [103] | Knowledge and practice among school nurses as they relate to childhood obesity. | |  | | | X | |  | | X | |  | |  | | X | No intervention (revised Pender’s Health Promotion model tool) | | | 35% of the school nurses reported a lack of competence in recommending weight-loss programs for children | B-III  Limited |
| Nguyen et al. (2008) [USA] [177] | | X | |  | |  | |  | |  | |  | Quantitative randomized control trials [36] | Blood glucose management in school for children with poorly controlled Typ-1 diabetes | |  | | | X | |  | |  | |  | |  | |  | 3-month intervention: access to the GlucoMON system (screening) | | | E.^[[1]](#footnote-1)^: Average HbA1c^[[2]](#footnote-2)^ was lowered by 1.6%, suggesting that this intervention helps this difficult group of patients. | E-V  High |
| Noyes et al. (2013) [USA] [178] | | X | |  | |  | |  | |  | |  | Quantitative randomized control trials [525] | Cost-effectiveness of the school-based asthma therapy program | |  | | | X | |  | |  | |  | |  | |  | Administration of 1 dose of preventive asthma medication at school by the school nurse (mean number of symptom-free days) | | | School-based asthma therapy program could be an economically effective program for children aged 3 to 10 years. | E-V  High |
| Nutbeam et al. (1990) [UK] [179] | |  | |  | |  | |  | |  | | X | Qualitative (phenomenology) [n/a] | Perspectives in school health in England and Wales | |  | | |  | |  | | X | |  | |  | | X | No intervention (n/a) | | | The evaluation of the concept of the “health promoting school” is highlighted as a key area for future development. | A-II  Limited |
|  | |  | |  | |  | |  | |  | |  |  |  | |  | | |  | |  | |  | |  | |  | |  |  | | |  |  |
| O’Donnell et al. (1983) [USA] [180] | |  | | X | | X | |  | |  | |  | Quantitative non-randomized study (pre-post design) [54 teachers and 1,106 students] | School nurse demonstrates that mini-grant funding can improve elementary nutrition education | |  | | |  | |  | |  | |  | |  | | X | Nutrition education | | | It appears that the school nurse can have a positive effect on the health education curriculum. | E-IV  High |
| O'Toole et al. (1996) [USA] [181] | | X | |  | |  | |  | |  | |  | Quantitative descriptive (survey) [1,036] | Nurses’ diagnostic work on possible physical child abuse | |  | | |  | |  | | X | |  | |  | |  | No intervention (questionnaire) | | | Nurses focused primarily on the child’s injuries and behaviour and wanted more information about the abuse event. | E-III  Moderate |
| Oda (1992) [USA] [182] | |  | |  | |  | |  | |  | | X | Qualitative (narrative research) [n/a] | Is school nursing really the “Invisible Practice?” | |  | | |  | |  | | X | |  | |  | | X | No intervention (n/a) | | | The “invisibility” must be transformed to a strong presence readily recognizable and recognized | A-I  Limited |
| Olowokere and Okanlawon (2014) [Nigeria] [183] | | X | |  | | X | |  | | X | |  | Quantitative non-randomized study (pre-post design) [109] | The effects of a school-based psychosocial intervention on resilience and health outcomes among vulnerable children | |  | | |  | |  | | X | |  | |  | |  | Exp.1: resilience training, Exp.2: peer support group Exp.3: Exp.1+Exp.2 (anxiety, depression and self-esteem questionnaires + knowledge scores) | | | Significant increase in knowledge of nurses and teachers; children’s depression scores significantly reduced post intervention. | E-IV  High |
| Paavilainen et al. (2000) [Finland] [184] | |  | |  | |  | |  | | X | |  | Qualitative description [20] | School nurses caring for child abusing families | |  | | |  | |  | | X | | X | |  | |  | No intervention (semi-structured interviews) | | | It is important that nurses are trained to work with families who abuse their children | B-II  Limited |
| Paavilainen et al. (2014) [Finland] [185] | |  | |  | |  | |  | | X | |  | Quantitative descriptive (survey) [367] | How school nurses intervene and help in child maltreatment | |  | | |  | |  | | X | |  | |  | |  | No intervention (Web-based questionnaires on how school nurses implement the National Clinical Guideline) | | | School nurses worked quite well with children who had experienced maltreatment and families | C-III  Moderate |
| Paavilainen and Tarkka (2003) [Finland] [186] | |  | |  | |  | |  | | X | |  | Qualitative (qualitative description) [20] | Definition and identification of child abuse by Finnish school nurses | |  | | |  | |  | | X | |  | |  | |  | No intervention (focussed interviews) | | | Knowledge of how abused children and their families should be cared for is lacking | B-II  Limited |
| Pakieser et al. (1998) [USA] [187] | |  | |  | |  | |  | | X | |  | Quantitative descriptive (survey) [121] | Emotional maltreatment of school-age children | | X | | |  | |  | | X | |  | |  | |  | No intervention (questionnaire) | | | No nurse characteristics correlated with referral rates. | C-III  Moderate |
| Pbert et al. (2016) [USA] [188] | | X | |  | |  | |  | |  | |  | Randomized controlled trial [126] | School-based program for overweight and obese adolescents | |  | | | X | |  | | X | |  | |  | |  | School nurse-delivered cognitive-behavioural counselling intervention plus school-based after school exercise program (anthropometric and behavioural assessments) | | | At follow-up, students in intervention compared with control schools were not different in BMI, percent body fat, and waist circumference | E-V  High |
| Pbert et al. (2013) [USA] [189] | | X | |  | |  | |  | |  | |  | Randomized controlled trial [84] | School nurse-delivered intervention for overweight and obese adolescents | |  | | | X | |  | | X | |  | |  | |  | School nurse-delivered counselling intervention utilizing cognitive-behavioural techniques (Tanita Scale, BMI, blood pressure and waist circumstance) | | | A brief school nurse-delivered intervention was feasible, acceptable, and improved selected obesogenic behaviours, but not BMI. | E-V  High |
| Peckover and Trotter (2015) [UK] [190] | |  | |  | |  | |  | | X | | X | Qualitative (qualitative descriptive) [23] | Challenges of safeguarding children affected by domestic abuse | |  | | |  | |  | | X | |  | |  | | X | No intervention (focus groups) | | | Discussion focused upon professional’s awareness of domestic abuse, how they assessed and met young peoples’ needs | C-II  Limited |
| Peery et al. (2012) [USA] [191] | | X | |  | | X | | X | |  | |  | Quantitative non-randomized (pre-post design) [69] | Parent and teacher perceptions of the impact of school nurse intervention on children’s self-management of diabetes | |  | | | X | |  | |  | |  | |  | |  | Case Management (questionnaire) | | | Parents and teachers do not always agree on how well a child is managing their diabetes | B-IV  Limited |
| Pennington and Delaney (2008) [USA] [192] | |  | | X | |  | |  | |  | |  | Quantitative descriptive (prevalence study) [3,132] | Number of students sent home by school nurses compared to unlicensed personnel | |  | | |  | |  | |  | |  | |  | | X | No intervention (incident/illness report) | | | 5% of students seen by the school nurse and 18% of students seen by an unlicensed school employee were sent home. | E-III  Moderate |
| Perry and Toole (2000) [USA] [193] | | X | |  | |  | |  | |  | |  | Qualitative descriptive (pre-post design) [17] | Impact of school nurse case management on asthma control | |  | | | X | |  | | X | |  | |  | | X | School-based education model (unknown) | | | Reviewing medications at a health care visit is insufficient for parents to become competent in medication administration. | E-II  Limited |
| Persaud et al. (1996) [USA] [194] | | X | |  | |  | |  | |  | |  | Randomized controlled trial [36] | An asthma self-management program | |  | | | X | |  | |  | |  | |  | |  | School nurses taught children asthma self-management principles and skills, including peak flow monitoring, in 20-min, individual sessions over an 8-week period (pulmonary function) | | | No significant differences in the number of postintervention emergency room visits and days absent but children showed less anxiety in experimental group | E-V  High |
| Petitgout et al. (2013) [USA] [195] | | X | |  | |  | |  | |  | |  | Qualitative (case study) [n/a] | Hospital-based care coordination program for children with special health care needs | |  | | |  | |  | |  | |  | |  | | X | No intervention (n/a) | | | Improved outcomes, including decreased length of stay, decreased cost, and high family satisfaction | A-II  Limited |
| Powell et al. (2017) [USA] [196] | |  | |  | |  | |  | | X | |  | Qualitative (qualitative description) [10] | Experiences of school nurses caring for students with overweight and obesity | | X | | |  | |  | |  | |  | |  | |  | No intervention (in-depth, open-end interviews) | | | Findings provide a better understanding of the experiences of school nurses caring for children with overweight. | B-II  Limited |
| Presler (1998) [USA] [197] | | X | |  | |  | |  | |  | |  | Qualitative (case study) [n/a] | Care coordination for children with special needs | |  | | |  | |  | |  | |  | |  | | X | No intervention (n/a) | | | Children's Healthcare Options Improved Through Collaborative Efforts and Services (CHOICES) shows that children with special health care needs are better served. | C-II  Limited |
| Pryjmachuk et al. (2012) [UK] [198] | |  | |  | |  | |  | | X | |  | Qualitative (qualitative descriptive) [33] | School nurses’ perspectives on managing mental health problems | |  | | |  | |  | |  | |  | |  | | X | No intervention (focus group) | | | School nurses can be engaged in mental health work though, their role should focus on health promotion, assessment and early intervention | C-II  Limited |
| Pulcini et al. (2011) [USA] [199] | |  | |  | |  | |  | | X | |  | Quantitative descriptive (survey) [194] | Food allergy emergency action plans | |  | | | X | |  | |  | |  | |  | |  | No intervention (investigator-developed food allergy survey) | | | Food allergy Emergency Action Plan (EAPs) for allergic students, are in inconsistent use in Mississippi. | E-III  Moderate |
|  | |  | |  | |  | |  | |  | |  |  |  | |  | | |  | |  | |  | |  | |  | |  |  | | |  |  |
| Putman-Casdorph and Pinto (2011) [USA] [200] | |  | |  | |  | |  | | X | |  | Quantitative non-randomized study (pre-post design) [20] | Preliminary testing of an asthma distance education program for school nurses in appalachia | |  | | |  | | X | |  | |  | |  | |  | Asthma education module following the National Institutes of Health (NIH), National Asthma Education Prevention Program (NAEPP) Asthma Care Guidelines (2007) (School Health Personnel Questionnaire SHPQ) | | | Findings show that distance learning technology could be a viable solution for school nurses to receive asthma continuing education. | B-IV  Limited |
| Quaranta and Spencer (2016) [USA] [201] | |  | |  | |  | |  | | X | |  | Quantitative (quantitative description) [537] | Barriers to asthma management as identified by school nurses | |  | | | X | |  | | X | |  | |  | |  | No intervention (questionnaires) | | | As numbers of barriers increased, performance of asthma management behaviours decreased. | B-III  Limited |
| Quelly (2013) [USA] [202] | |  | |  | |  | |  | | X | |  | Quantitative non-randomized study (cross-sectional analytic study) [171] | Influence of perceptions on school nurse practices to prevent childhood obesity | |  | | | X | |  | |  | |  | |  | |  | No intervention (survey, child level practice scale, perceived benefits scale, perceived barriers scale, level of practice scale, self-efficacy scale) | | | Mediation testing identified perceived barriers as a partial mediator of the influence of self-efficacy on child-level practices. | B-IV  Limited |
| Raible et al. (2017) [USA] [203] | |  | | X | |  | |  | |  | |  | Qualitative (case study) [566] | School nurse-delivered adolescent relationship abuse prevention | |  | | |  | |  | | X | |  | |  | |  | Adolescent relationship abuse prevention intervention (qualitative interviews) | | | School nurses identified strategies to achieve school and student support for this intervention. | E-II  Limited |
| Ramirez et al. (2013) [USA] [204] | | X | |  | |  | |  | |  | |  | Quantitative non-randomized study (pre-post design) [20] | Listen protect connect for traumatized schoolchildren: a pilot study of psychological first aid | | X | | |  | |  | |  | |  | |  | |  | Listen Protect Connect (LPC) (questionnaires) | | | Students who received the intervention had reduced depressive and posttraumatic stress symptoms from baseline throughout follow-up period. | E-IV  High |
| Ramos et al. (2013) [USA] [205] | |  | |  | |  | |  | | X | |  | Quantitative descriptive (survey) [186] | Behavioural health emergencies managed by school nurses | |  | | |  | |  | |  | |  | |  | | X | No intervention (survey) | | | School nurses provide enough emergency management for behavioural health problems | C-II  Limited |
| Rasberry et al. (2015) [USA] [206] | | X | |  | |  | |  | |  | |  | Quantitative descriptive (case series) [447] | Sexual orientation and sexual health: perspectives of teen young men who have sex with men | |  | | |  | |  | | X | |  | |  | |  | No intervention (cross-sectional data through web-based questionnaires and interviews) | | | Results show that men are reluctant to talk with school nurses when uncertain about their perception of Lesbian, Gay, Bisexual, Transgender, Queer (LGBTQ) people. | D-III  Moderate |
|  | |  | |  | |  | |  | |  | |  |  |  | |  | | |  | |  | |  | |  | |  | |  |  | | |  |  |
| Rebmann et al. (2016) [USA] [207] | |  | |  | |  | |  | | X | |  | Quantitative non-randomized study (pre-post design) [47] | Impact of an education intervention on school disaster and biological event preparedness | |  | | |  | |  | | X | |  | |  | |  | Online education modules for school nurses (school biological event readiness was measured using 35 indicators) | | | The education intervention was effective at improving school preparedness, though the impact was small. | C-IV  Moderate |
| Reid (1991) [UK] [208] | |  | |  | |  | |  | | X | |  | Qualitative (case study) [n/a] | The role of the school nurse in public health | |  | | |  | | X | |  | |  | |  | |  | No intervention (n/a) | | | For the future, we need a clearer public health approach to the school age population. | A-II  Limited |
| Resnicow and Allensworth (1996) [USA] [209] | |  | |  | |  | |  | | X | | X | Qualitative (case study) [n/a] | Conducting a comprehensive school health program | |  | | |  | |  | |  | | X | |  | | X | No intervention (n/a) | | | This article proposes the school health coordinator as an essential element in the eight-component model of the Comprehensive School Health Program (CSHP) | A-II  Limited |
| Rhodes et al. (2017) [USA] [210] | |  | |  | |  | |  | | X | |  | Quantitative descriptive (survey) [197] | Practices and attitudes of missouri school nurses regarding immunization records | |  | | |  | |  | | X | |  | |  | |  | No intervention (survey) | | | Approximately 1/2 and 1/3 of nurses do not communicate with parents/students about HPV or meningococcal vaccines, respectively | B-III  Limited |
| Rivkina et al. (2014) [USA] [211] | |  | |  | |  | | X | | X | |  | Mixed method design: (1) Qualitative (qualitative description); (2) Quantitative descriptive (survey) | Identifying barriers to chronic disease reporting | |  | | | X | |  | |  | |  | |  | |  | No intervention (focus group, parent interview and school nurse interview) | | | Increasing parental knowledge of the reporting process will allow schools to better identify and manage their students’ chronic conditions | B-II  Limited |
| Robbins et al. (2012) [USA] [212] | |  | | X | |  | |  | |  | |  | Quantitative non-randomized study (pre-post design) [69] | Pilot intervention to increase physical activity among sedentary urban middle school girls | |  | | |  | |  | | X | |  | |  | |  | Intervention was based on the Health Promotion Model (cognitive and affective variables, physical activity, cardiovascular and body composition) | | | Intervention effect was non-significant but its directionality, along with the positive gain related to the effect size, lends support for continued evaluation of the intervention | E-IV  High |
| Roden (1997) [UK] [213] | |  | | X | |  | |  | |  | |  | No study design | Proving the value of school nursing service | |  | | |  | |  | | X | |  | |  | |  | No intervention (n/a) | | | Providing clinical effectiveness is a priority of NHS, as well as school nursing | C-I  Limited |
|  | |  | |  | |  | |  | |  | |  |  |  | |  | | |  | |  | |  | |  | |  | |  |  | | |  |  |
| Rodriguez et al. (2013) [USA] [214] | |  | | X | |  | |  | |  | |  | Quantitative non-randomized study (pre-post design) [6,664] | Asthma management, school absenteeism, and cost savings | |  | | |  | |  | |  | |  | | X | |  | Exp.: Full-time school nurse; Cont.: Part-time school nurse (student health and well-being measures before and after implementation) | | | School nurses play an important role in improving asthma management among students, which can impact absenteeism and economic costs. | E-IV  High |
| Rose et al. (1987) [USA] [215] | |  | |  | | X | |  | |  | |  | Qualitative (case study) [102] | Development of an In-Service education program by school nurses | |  | | |  | | X | |  | |  | |  | |  | Health education program and materials for teachers (questionnaire) | | | 63% rated the program as effective and productive. | A-II  Limited |
| Rose (2013) [UK] [216] | |  | |  | |  | |  | | X | |  | No study design | School Nursing from the perspective of Sharon Rose | |  | | |  | | X | |  | |  | |  | |  | No intervention (n/a) | | | School nurses' work is the continuation of what health visitors do for families and children of years | A-I  Limited |
| Rosen et al. (2017) [USA] [217] | |  | |  | |  | |  | | X | |  | Quantitative descriptive (survey) [413] | Factors associated with school nurses’ HPV vaccine attitudes for school-aged youth | |  | | |  | |  | | X | |  | |  | |  | No intervention (web-based survey) | | | Focus on school nurses’ perception of their role as opinion leaders and knowledge to increase positive attitudes towards HPV vaccination for youth. | C-III  Moderate |
| Rosen et al. (2015) [USA] [218] | |  | |  | |  | |  | | X | |  | Quantitative descriptive (survey) [505] | School nurses' knowledge, attitudes, perceptions of role as opinion leader, regarding HPV vaccines | |  | | |  | |  | |  | |  | |  | |  | No intervention (survey) | | | Overall, school nurses had knowledge about HPV and the vaccine, and positive attitudes toward the vaccine | C-III  Moderate |
| Rote (1997) [USA] [219] | |  | |  | |  | |  | | X | |  | No study design | Commentary on cutting back on school nurses | |  | | |  | |  | | X | |  | |  | |  | No intervention (no data) | | | Cutting back on school nurses now will cost more for the health service in the future | C-I  Limited |
| Rustia et al. (1984) [USA] [220] | |  | |  | | X | | X | |  | | X | Quantitative descriptive (survey) [505] | Redefinition of school nursing practice | |  | | |  | |  | |  | |  | |  | | X | No intervention (interviews, survey and environmental assessments) | | | School nurses have not been meeting school health needs through an analysis of one specific need | A-III  Limited |
| Salend and Mahoney (1982) [USA] [221] | | X | |  | |  | |  | |  | |  | Quantitative descriptive (case study) [2] | Teaching proper health habits to students through positive reinforcement | |  | | |  | |  | | X | |  | |  | |  | Positive reinforcement program | | | Intervention by the school nurse was effective in increasing the students’ ability to demonstrate appropriate hygiene habits | E-III  Moderate |
|  | |  | |  | |  | |  | |  | |  |  |  | |  | | |  | |  | |  | |  | |  | |  |  | | |  |  |
| Salmon et al. (2004) [USA] [222] | |  | |  | |  | |  | | X | |  | Quantitative descriptive (survey) [594] | Knowledge, attitudes, and beliefs towards immunization | |  | | |  | |  | | X | |  | |  | |  | No intervention (survey) | | | Many school personnel seem unaware of the seriousness of some vaccine-preventable diseases | C-III  Moderate |
| Savage and Goodall (2006) [UK] [223] | |  | | X | |  | |  | |  | |  | No study design | Making a drama out of teenage obesity | |  | | |  | | X | |  | |  | |  | |  | No intervention (n/a) | | | School nurses tackle teenage obesity by performing as overweight teenagers. | A-I  Limited |
| Schaffer et al. (2016) [USA] [224] | |  | |  | |  | |  | | X | |  | Quantitative descriptive (survey) [605] | The public health intervention wheel | |  | | |  | | X | |  | |  | |  | |  | No intervention (survey) | | | The public health intervention wheel is a relevant and useful framework to explain school nursing practice | A-III  Limited |
| Schols et al. (2013) [The Netherlands] [225] | |  | |  | | X | |  | | X | |  | Qualitative descriptive (focus groups) [16 teachers, 17 nurses] | Identifying and handling child abuse cases | |  | | |  | |  | | X | |  | |  | |  | No intervention (focus groups) | | | Frontline workers are in need of supportive tools in the child abuse detection and reporting process | C-II  Limited |
|  | |  | |  | |  | |  | |  | |  |  |  | |  | | |  | |  | |  | |  | |  | |  |  | | |  |  |
| Schonfeld (1996) [USA] [226] | |  | | X | |  | |  | |  | |  | No study design | Principles and guidelines for talking with elementary school-age children about AIDS and death | |  | | | X | |  | | X | |  | |  | |  | No intervention (no data) | | | Nurses and teachers have the task of imparting to young students what they know, while being sensitive to the questioning that precedes a child’s comprehension of such unfortunate facts | A-I  Limited |
| Schroeder et al. (2017) [USA] [227] | | X | |  | |  | |  | |  | |  | Quantitative descriptive (prevalence study without comparison group) [1,054] | Evaluate implementation of the Healthy Options and Physical Activity Program (HOP) program by examining the proportion and characteristics of eligible children | |  | | | X | |  | |  | |  | |  | |  | No intervention (student electronic health records, NYC DOHMH Office of School Health records, and New York Center for Economic Opportunity poverty data) | | | Low nurse workload, low school poverty, higher grade level, higher BMI percentile, and chronic illness diagnosis were associated with student enrolment in HOP | A-III  Limited |
| Schwartz et al. (2010) [USA] [228] | | X | |  | |  | |  | |  | |  | Quantitative descriptive (survey) [80] | Children and adolescents with Type 1 diabetes in school | |  | | | X | |  | |  | |  | |  | |  | No intervention (questionnaire) | | | Children’s and parent’s perceptions and experiences are generally good and have improved compared to earlier surveys | C-III  Moderate |
| Scott and Hall (2012) [USA] [229] | |  | | X | |  | |  | |  | |  | Quantitative descriptive (case series) [288] | Reliability and validity of the acanthosis Nigricans screening Tool | |  | | | X | |  | | X | |  | |  | |  | No intervention (measuring the interrater reliability of Acanthosis Nigricans (AN) screening tool) | | | The AN screening tool is easy to use and reliable for use with elementary school-age children of various ethnic groups. | B-III  Limited |
| Seidenberg (1984) [USA] [230] | |  | |  | |  | |  | | X | |  | Not scientific | Diversity of school nursing practice today | |  | | |  | |  | |  | |  | |  | |  | No intervention (n/a) | | | Autonomy and knowledge are essential for the successful completion of the school nursing role | A-I  Limited |
| Sekhar et al. (2014) [USA] [231] | |  | | X | |  | |  | |  | |  | Quantitative randomized trial (prevalence study without comparison group) [282] | Pilot study of a high-frequency school-based hearing screen to detect adolescent hearing loss | |  | | | X | |  | |  | |  | |  | |  | Pennsylvania’s school hearing screen compared to high-frequency screen (screenings) | | | Current school hearing screens have low sensitivity for detection of adolescent hearing loss | E-IV  High |
| Sekhar et al. (2018) [USA] [232] | |  | |  | | X | | X | | X | | X | Qualitative (qualitative descriptive) [62] | Opportunities and challenges in screening for childhood sexual abuse | |  | | |  | |  | | X | |  | |  | |  | No intervention (focus groups) | | | Next steps should involve partnering with evidence-based programs, e.g., Safe Touches (Pulido et al., 2015) | C-II  Limited |
| Sherman et al. (1992) [USA] [233] | | X | |  | |  | |  | |  | |  | Quantitative non-randomized study (pre-post design) [26] | Intervention program for obese school children | |  | | | X | |  | |  | |  | | X | |  | Modelling positive behaviours (self-esteem, exercise record, nutritional knowledge) | | | Weight status and nutritional knowledge showed no improvement. | E-IV  High |
| Singer (2013) [USA] [234] | | X | |  | |  | |  | | X | |  | Qualitative (narrative research) [8 SN] | Perceptions of school nurses in the care of students with disabilities | |  | | |  | | X | |  | |  | |  | |  | No intervention (interviews, observations, and field notes) | | | School nurses lacked education and encountered challenges with communication, health assessments, and screenings. | B-II  Limited |
| Skelley et al. (2013) [USA] [235] | |  | | X | |  | |  | |  | |  | Quantitative descriptive (survey) | Assess parental perspectives on care for children with diabetes in the Alabama public school system | |  | | | X | |  | |  | |  | |  | | X | No intervention (survey based on American Diabetes Association) | | | Students who conveniently check their Blood Glucose Level (BGLs) at school were more likely to participate in all school activities and parents were more satisfied | A-III  Limited |
| Snyder et al. (1980) [USA] [236] | | X | |  | |  | |  | |  | |  | Quantitative descriptive (prevalence study without comparison group) [610] | School children from broken homes and frequency of school nurse visits | |  | | |  | |  | | X | |  | |  | |  | No intervention (nurse’s log of student visits and student enrolment Cards) | | | Children from broken homes visit the school nurse in greater numbers than children from intact homes. | C-III  Moderate |
| Speroni et al. (2007) [USA] [237] | |  | | X | |  | |  | |  | |  | Quantitative non-randomized study (cross-sectional analytic study) [185] | Effectiveness of the Kids Living Fit Program | |  | | |  | |  | | X | |  | |  | |  | Kids Living Fit (KLF) intervention: fitness program and presentations on diet (food and activity diaries + pedometers, BMI, waist circumference) | | | Intervention group: decrease in BMI and smaller increase in waist circumference | D-IV  High |
| Spina et al. (2012) [USA] [238] | | X | |  | |  | |  | |  | |  | Quantitative randomized study [77] | Intervention to increase compliance with carrying auto-injectable epinephrine | |  | | | X | |  | | X | |  | |  | |  | Educational program (standardized data collection instruments by school nurses) | | | Periodic checks for the availability of epinephrine didn’t increase | E-V  High |
| Splett et al. (2006) [USA] [239] | | X | |  | |  | |  | |  | |  | Quantitative randomized study [1,561] | Evaluation of the healthy learner’s asthma initiative | |  | | | X | |  | |  | |  | | X | |  | The Healthy Learners Asthma Initiative (HLAI) (Daily visit log, student medication, and health records were audited) | | | Indication of sustained implementation of enhanced asthma care; increased asthma communication between school, parents and health care providers. | D-V  High |
| Spratt et al. (2010) [UK] [240] | |  | |  | |  | |  | | X | |  | Qualitative (case study) [25] | Nurses’ roles in supporting mental health | |  | | |  | | X | | X | |  | |  | |  | No intervention (interviews) | | | School nurses are a unique resource in order to build relationships in promoting mental health | B-II  Limited |
| Sprinks (2011) [USA] [241] | |  | | X | |  | |  | |  | |  | No study design | Nurse-led initiative focuses on parents to lessen health-related school absences | |  | | |  | |  | |  | |  | | X | |  | Pocket guides, school nurses talked to parents of children who are at risk of becoming persistently absent, daily drop-in clinic | | | Parents were less likely to keep children at home for long periods | E-I  Limited |
| Squires (2013) [UK] [242] | |  | |  | |  | |  | | X | |  | Qualitative (narrative research) [n/a] | Delivering quality health education: Clare Squires reflects on her delivery of the subject | |  | | |  | |  | | X | |  | |  | |  | No intervention (n/a) | | | “A single positive contact can make a substantial and lasting impact on children” | A-II  Limited |
| Stallard et al. (2007) [UK] [243] | |  | | X | |  | |  | |  | |  | Quantitative non-randomized study (pre-post design) [106] | The FRIENDS Emotional Health Programme | | X | | |  | |  | |  | |  | |  | |  | Evidence-based emotional health cognitive behaviour therapy programme (FRIENDS); (Levels of anxiety and self-esteem) | | | Children with the most severe emotional problems benefited from the programme | E-IV  High |
| Stallard et al. (2008) [UK] [244] | |  | | X | |  | |  | |  | |  | Quantitative non-randomized study (12-month follow-up) [63] | The FRIENDS emotional health programme: 12-month follow-up | | X | | |  | |  | |  | |  | |  | |  | No intervention - reassessment (levels of anxiety and self-esteem) | | | The significant improvements in emotional health were maintained 12 months after completing the programme. | E-IV  High |
| Stalter (2011a) [USA] [245] | |  | |  | |  | |  | | X | |  | Qualitative (Qualitative description) [25] | Barriers to Body Mass Index Screening | |  | | | X | |  | | X | |  | |  | |  | No intervention (semi structured focus group questions) | | | Key barriers to BMI screening were a lack of privacy, time, policy, and workload of school nurses. | C-II  Limited |
| Stalter (2011b) [USA] [246] | |  | |  | |  | | X | |  | |  | Quantitative descriptive (survey) [65] | Parental perceptions on school’s role in addressing obesity | |  | | | X | |  | |  | |  | |  | |  | No intervention (Parental Perceptions of BMI and Obesity in the School-Age Child’) | | | Significance between overweight parents who had overweight children. | A-III  Limited |
| Stang et al. (1997) [USA] [247] | |  | |  | |  | |  | | X | | X | Qualitative description [630 school nurses + 1,137 school administrators] | School-based weight management services: perception and practices | |  | | | X | |  | |  | |  | |  | |  | No intervention (questionnaire) | | | The majority believed that being overweight affects the physical, social, and emotional health of children. | C-II  Limited |
| Staudt et al. (2015) [USA] [248] | |  | |  | |  | |  | | X | |  | Quantitative descriptive (survey) [School Nurses of 16 Schools] | Implementing a citywide asthma action plan | | X | | |  | |  | |  | |  | |  | |  | Asthma action plan (survey) | | | The implementation of a citywide asthma plan was successful | E-III  Moderate |
| Steele et al. (2013) [USA] [249] | |  | |  | |  | |  | | X | |  | Quantitative randomized controlled trials [526] | A web-based tutorial to enhance school nurses’ communications with families about weight-related health | |  | | | X | | X | |  | |  | |  | |  | Web-based tutorial to improve school nurses’ communications (Child Health Matters -CHM-) (knowledge tests, Client Satisfaction Questionnaire -CSQ-8-) | | | Immediate-access nurses demonstrated significant increases in knowledge and decreases in perceived barriers | B-V  Limited |
| Stephenson (1983) [USA] [250] | |  | | X | |  | |  | |  | |  | Qualitative (qualitative description) [551] | Visits by elementary school children to the school nurse | |  | | |  | |  | | X | |  | |  | |  | No intervention (flow sheets from school nurse visits, data computer sheet, health records) | | | Health seeking behaviour was a learned process, no difference in number of visitations by boys or girls. | A-II  Limited |
|  | |  | |  | |  | |  | |  | |  |  |  | |  | | |  | |  | |  | |  | |  | |  |  | | |  |  |
| Streeting (2010) [UK] [251] | |  | |  | |  | |  | | X | |  | Qualitative (narrative research) [n/a] | Personal reflections on working within a multi-disciplinary student support team as a school nurse | |  | | |  | |  | |  | |  | |  | | X | No intervention (n/a) | | | Working 'multi-agency' demands a high level of competence and experience from all practitioners | A-II  Limited |
| Swallow and Roberts (2016) [USA] [252] | |  | | X | |  | |  | |  | |  | Quantitative descriptive (prevalence study) [1,834 in 1st year; 1,760 in 2nd year; 1,835 in 3rd year] | School immunization compliance | |  | | |  | |  | | X | |  | |  | |  | Three letters to parents including an explanation of exclusion and a date when exclusion would apply (SMS Power-school, a student data management system) | | | Postintervention, vaccine compliance was 99.6%, exceeding both national and state averages. | C-III  Moderate |
| Szychlinski et al. (2015) [USA] [253] | |  | |  | |  | |  | | X | |  | Quantitative descriptive (survey) [460] | Food allergy emergency preparedness | |  | | |  | | X | |  | |  | |  | |  | No intervention (cross-sectional online survey) | | | Opportunities for improvement in food allergy policy implementation, especially in rural communities | C-III  Moderate |
| Taras et al. (2004) [USA] [254] | | X | |  | |  | |  | |  | |  | Quantitative non-randomized study (cross-sectional analytic design) [1,094] | Impact of school nurse case management on students with asthma | |  | | | X | |  | |  | |  | |  | | X | School nurse case management intervention: (asthma tracking tool) | | | Students with intervention were more likely to have an asthma medication at school, to use a peak flow meter at school, and to have a change in asthma severity the following year | E-IV  High |
| Taylor et al. (2013) [USA] [255] | |  | |  | |  | |  | |  | | X | Quantitative descriptive (survey) [91 families] | Care coordination program for children with special healthcare needs | |  | | |  | |  | |  | |  | |  | | X | Care Coordination (CC) Counsellor (cross-sectional survey) | | | A CC Counsellor role and supporting tools offers a way to connect patients, families, and health providers to support coordinated, continuous care. | B-III  Limited |
| Telljohann et al. (2004) [USA] [256] | | X | |  | |  | |  | |  | |  | Quantitative non-randomized study (cross-sectional analytic study) [569] | Full-time vs part-time school nurses on attendance of students with asthma | |  | | | X | |  | |  | |  | | X | |  | Full-time school nurse and part-time school nurse (form with grade, race, sex, school lunch status, and number of days missed) | | | Students with full-time nurses missed significantly fewer days than did their counterparts with asthma and part-time nurses | E-IV  High |
| Terry et al. (2016) [USA] [257] | |  | |  | |  | |  | | X | |  | Quantitative descriptive (survey) [83] | School nurses’ perceptions of barriers to optimal management of seizures in schools | |  | | | X | |  | |  | |  | |  | |  | No intervention (online survey) | | | A barrier in urban schools is decreased availability of a nurse to identify seizures and administer treatment. | C-III  Moderate |
| Thompson (1989) [UK] [258] | |  | |  | |  | |  | | X | |  | Qualitative (narrative research) [n/a] | Comparing school health services in America and the UK | |  | | |  | | X | |  | |  | |  | |  | No intervention (n/a) | | | In both countries the foundation for the health of the school child should be laid down with good preventive health care | A-II  Limited |
| Thomson (2012) [UK] [259] | |  | | X | |  | |  | |  | |  | Quantitative non-randomized study [between 4,505 and 4,999 students] | A successful approach to reduce youth smoking | |  | | | X | |  | | X | |  | |  | |  | Multi-faceted approach: school-based stop smoking services + school nurse education (questionnaire) | | | Intervention schools showed a reduction in smoking prevalence of 8% compared to a reduction of only 5.7% in comparison schools | E-IV  High |
| Thurston and Walker (2011) [UK] [260] | |  | |  | |  | |  | | X | |  | Qualitative (narrative research) [19] | Experiences and training in delivering sexual health care | |  | | |  | | X | |  | |  | |  | |  | No intervention (survey) | | | A need for more training: social, ethical, legal and religious issues, gender inequality and sexual assault. | B-II  Limited |
| Toole and Perry (2004) [USA] [261] | |  | | X | |  | |  | |  | |  | Quantitative non-randomized trials (pre-post design) [2,222] | Increasing immunization compliance | |  | | |  | |  | | X | |  | |  | |  | Immunization for free + information for parents (immunization audit) | | | Compliance in the district has risen from an overall level of 50–60% to 90–100% | D-IV  High |
| Triggle (2014) [UK] [262] | | X | |  | |  | |  | |  | |  | Qualitative (narrative research) [n/a] | Nurse-led service that supports children from traveller families | |  | | |  | |  | | X | |  | |  | |  | School Nurses for a “difficult-to-reach community” (n/a) | | | “As a service, they are always upfront with the parents of the children they work with, pointing out that where there is a risk of neglect or abuse, they have a duty to intervene. “ | E-II  Limited |
| Trivedi et al. (2017) [USA] [263] | | X | |  | |  | |  | |  | |  | Quantitative non-randomized trials (pre-post design) [84] | School nurse asthma program reduces healthcare utilization | |  | | | X | |  | | X | |  | |  | |  | School nurse-supervised asthma therapy program: daily inhaled corticosteroid at school (Emergency Department visits, hospital admissions) | | | Significant reduction in healthcare utilization for children enrolled in this program | E-IV  High |
| Tsacoyianis (1997) [USA] [264] | |  | |  | |  | |  | |  | | X | Quantitative descriptive (case study) [54 sheets] | Indoor Air Pollutants and Sick Building Syndrome | |  | | | X | |  | |  | |  | |  | |  | No intervention (Material Safety Data Sheets (MSDS) and Product Data Sheets) | | | Imminent need for community health nurses to assume a proactive role in promoting environmental health | A-III  Limited |
| Tucker and Lanningham-Foster (2015) [USA] [265] | |  | | X | |  | |  | |  | |  | Quantitative non-randomized (pre-post design) [72] | Nurse-led School-based child obesity prevention | |  | | |  | |  | | X | |  | |  | |  | Let’s Go 5-2-1-0 program (PA, body mass index percentile, and self-reported health habits) | | | Statistically significant increases in PA levels and improvements in child-reported health habits | E-IV  High |
| Turner et al. (2009) [UK] [266] | |  | |  | |  | |  | | X | | X | Qualitative (phenomenology) [30] | Practitioners’ views on managing childhood obesity in primary care | |  | | | X | |  | |  | |  | |  | | X | No intervention (interviews) | | | Practitioners do not currently view primary care as an effective treatment setting for childhood obesity | C-II  Limited |
| Urbinati et al. (1996) [USA] [267] | |  | |  | |  | |  | | X | |  | Qualitative (phenomenology) | Evolution of the school nurse practitioner | |  | | | X | | X | |  | |  | |  | |  | No intervention (n/a) | | | Lives of children can be influenced positively through the school nurse | C-II  Limited |
| Van Cura (2010) [USA] [268] | |  | | X | |  | |  | |  | |  | Quantitative non-randomized (cross-sectional analytic study) [764 ‘‘walk-in’’ visits] | Relationship between school-based health centers (SBHC) and academic outcomes | |  | | |  | |  | |  | |  | | X | |  | SBHC and traditional school nursing services | | | Findings suggest that SBHCs have a direct impact on educational outcomes such as attendance | E-IV  High |
| Van Roeyen (2013) [USA] [269] | | X | |  | |  | |  | |  | |  | Qualitative (case study; summary of findings) [n/a] | Management of paediatric asthma at home and in school | |  | | | X | |  | |  | |  | |  | |  | No intervention (n/a) | | | Asthma management is positively impacted through improved education for children and their families | C-II  Limited |
| Vanderpool et al. (2015) [USA] [270] | |  | | X | |  | |  | |  | |  | Quantitative descriptive (prevalence study without comparison group) [395] | Implementation and evaluation of a school-based human papillomavirus vaccination program | |  | | |  | |  | | X | |  | |  | |  | HPV vaccination project (rates of returned parental consent forms and HPV vaccination rates) | | | School-based immunization programs are an effective strategy for improving HPV vaccination rates | E-III  Moderate |
| Vernberg et al. (2011) [USA] [271] | |  | | X | |  | |  | |  | |  | Quantitative descriptive (prevalence study without comparison group) [590] | Victimization, aggression, and visits to the school nurse for somatic complaints | |  | | | X | |  | | X | |  | |  | |  | No intervention (self-report, peer report and school nurse’s logs) | | | Involvement in aggressor-victim interactions, as either aggressor, victim, or both, is associated with more frequent health complaints | C-III  Moderate |
| Vernon et al. (1976) [USA] [272] | |  | | X | |  | |  | |  | |  | Quantitative non-randomized trials (cohort study) [5,636] | Improving immunization levels | |  | | |  | |  | | X | |  | |  | |  | Method A, B, C compared between each other and compared with pre-clinic review (immunization rates) | | | School immunization record review and extra attention to immunization- deficient children will increase immunization rates | E-IV  High |
| Vessey and O’Neill (2011) [USA] [273] | | X | |  | |  | |  | |  | |  | Mixed Method: (1) Quantitative non-randomized trials (pre-post design; (2) Qualitative (qualitative description) [65] | Helping students with disabilities better address teasing and bullying situations | | X | | |  | |  | |  | |  | |  | |  | Web-based program: 12-session, biweekly support/discussion group intervention (Child-Adolescent Teasing Scale; Paediatric Symptom Checklist; Piers-Harris Children’s Self-concept Scale) | | | Students reported being significantly less bothered by teasing and possessed significantly improved self-concepts. | E-IV  High |
| Wall (2005) [USA] [274] | |  | | X | |  | |  | |  | |  | Qualitative (narrative research) [unknown] | Tai Chi and mindfulness-based stress reduction in a Boston public middle school | | X | | |  | |  | | X | |  | |  | |  | Combined Tai Chi and mindfulness-based stress reduction | | | Students experienced well-being, calmness, relaxation, improved sleep, less reactivity, increased self-care, self-awareness, and a sense of interconnection | C-II  Limited |
| Watson (2008) [UK] [275] | |  | |  | |  | |  | | X | |  | Qualitative (narrative research) [1] | The unique role of the school nurse | |  | | |  | | X | |  | |  | |  | |  | No intervention (n/a) | | | The modern school nurse is a highly trained and skilled health promotion specialist | C-II  Limited |
| Weismuller et al. (2007) [USA] [276] | |  | | X | |  | |  | |  | |  | Quantitative non-randomized trial (case study) [240 health folders] | Documented linkages between school nurse interventions and elementary student attendance and health. | |  | | |  | |  | | X | |  | | X | |  | Number of interventions examined (frequency of interventions and school absences) | | | 134 interventions provided, 56 had information on outcomes, but were insufficient to determine the effectiveness of nursing interventions. | E-IV  High |
| Wells et al. (1998) [USA] [277] | |  | |  | |  | |  | |  | | X | Quantitative non-randomized trial (pre-post design) [335] | Interdisciplinary collaboration in an acute care hospital located in an academic medical centre | |  | | |  | |  | |  | | X | |  | |  | No intervention (composition of hospital staff) | | | Findings suggest the importance of perceived physician involvement in collaborative practice. | A-IV  Limited |
| Werch et al. (1996) [USA] [278] | |  | | X | |  | |  | |  | |  | Quantitative randomized controlled trials [138] | Preventing alcohol use among urban school youth | |  | | |  | |  | | X | |  | |  | |  | Start Taking Alcohol Risks Seriously (STARS) program (alcohol use acquisition) | | | Significant difference on heavy alcohol use with intervention subjects showing a reduction and control subjects an increase in heavy drinking | E-V  High |
| White et al. (2016) [USA] [279] | |  | |  | |  | |  | |  | | X | Quantitative descriptive (survey) [6,574 schools] | Anaphylaxis in Schools: Results of the EpiPen 4 schools survey Combined Analysis | |  | | | X | |  | | X | |  | |  | |  | No intervention (cross-sectional survey) | | | For students, anaphylactic events were reported across all grades. A majority of schools permitted only the school nurse to treat anaphylaxis | A-III  Limited |
| Whitmarsh (1997) [UK] [280] | |  | |  | |  | |  | | X | |  | Qualitative (qualitative description) [50] | School nurses' skills in sexual health education | |  | | |  | | X | |  | |  | |  | |  | No intervention (questionnaire) | | | The respondents were considerably more confident about their knowledge base than their teaching skills | B-II  Limited |
| Whitmore (1988) [UK] [281] | |  | | X | |  | |  | |  | |  | Qualitative (narrative research) [n/a] | School refusal | |  | | |  | |  | |  | |  | | X | |  | No intervention (n/a) | | | The two most significant elements in the prognosis of school refusal, are early diagnosis and early return to school | E-II  Limited |
| Wicklander (2005) [USA + UK] [282] | |  | |  | |  | |  | | X | |  | Qualitative description (case study) [n/a] | A framework for strengthening the school nurse role | |  | | |  | | X | | X | |  | |  | | X | No intervention (n/a) | | | The framework is a model to help strengthen both the school nurse role and school health programs | C-II  Limited |
| Wiggs-Stayner et al. (2006) [USA] [283] | |  | | X | |  | |  | |  | |  | Quantitative non-randomized trials (cohort study) [277+ unknown control] | Impact of mass school immunization on school attendance | |  | | |  | |  | | X | |  | | X | |  | Free FluMist immunizations on site (self- or parent-reported influenza + attendance rates) | | | The difference in days absent between individuals vaccinated and nonvaccinated schools was statistically significant | E-IV  High |
| Williams and Warrington (2011) [USA] [284] | |  | | X | |  | |  | |  | |  | Quantitative non-randomized trials (pre-post design) [231] | A feasibility study of a pedometer-based walking program | |  | | | X | |  | | X | |  | |  | |  | 12-week, pedometer-based walking program (change in step counts, participant satisfaction, and program costs, BMI and teacher satisfaction | | | Early potential benefits for a pedometer-based walking program for elementary-aged children | D-IV  High |
| Wilson et al. (2014) [USA] [285] | |  | |  | |  | |  | | X | |  | Quantitative descriptive (case series) [n/a] | Description of a school nurse visit syndromic surveillance system and comparison to emergency department visits | |  | | | X | |  | |  | |  | |  | |  | No intervention (school nurse visit data) | | | 6.7 million visits to the school nurse and 0.9 million visits to the Emergency Department. | A-III  Limited |
| Wing et al. (2014) [USA] [286] | |  | |  | |  | |  | | X | |  | Quantitative descriptive (survey) [151] | Communication is key in school nurses’ preparedness for facilitating “Return to Learn” following concussion | |  | | | X | |  | |  | |  | |  | |  | No intervention (survey) | | | 19% felt that they did not have the training necessary to function within the academic rehabilitation team for students | C-III  Moderate |
| Winkelstein (1995) [USA] [287] | | X | |  | |  | |  | |  | |  | Qualitative (case study) [120] | Teaching pregnant adolescents to cope with environmental smoke | |  | | | X | |  | | X | |  | |  | |  | Study guide containing questions by teacher, educational program by paediatric nurse | | | At the end of the two classes, students were able to meet all learning objectives | C-II  Limited |
| Wong and Cheng (2013) [Hong Kong] [288] | | X | |  | |  | |  | |  | |  | Quantitative non-randomized trials (pre-post design) [185] | Effects of motivational interviewing to promote weight loss in obese children | |  | | |  | |  | |  | |  | |  | |  | Motivational interviewing and telephone consultation for parents (change in weight for-height percentage) | | | Motivational interviewing appears to be a promising intervention for promoting weight loss in obese children | D-IV  High |
| Wright et al. (2013) [USA] [289] | | X | |  | |  | |  | |  | |  | Quantitative randomized controlled trial [251] | Enhance physical activity behaviours and reduce body mass index among minority children | |  | | |  | |  | | X | |  | |  | |  | Kids N Fitness, a 6-week program with physical activity and nutrition education (Child and Adolescent Trial for Cardiovascular Health School Physical Activity and Nutrition Student Questionnaire) | | | Study shows the value of utilizing nurses to implement a culturally sensitive, coordinated, intervention to decrease disparities in activity and TV viewing among underserved girls and boys | E-V  High |

*Notes.* Information value rated based on matrix for categorization of content dimension and study design.

1. Adams C. Perceptions of the comprehensive-based school nurse. Health visitor. 1990;63(3):90-2.

2. Alizadeh V, Törnkvist L, Hylander I. Counselling teenage girls on problems related to the ‘protection of family honour’from the perspective of school nurses and
 counsellors. Health & social care in the community. 2011;19(5):476-84.

3. Allen G. The impact of elementary school nurses on student attendance. J Sch Nurs. 2003;19(4):225-31. Epub 2003/07/29. doi: 10.1177/10598405030190040801.
 PubMed PMID: 12882606.

4. Allen K, Henselman K, Laird B, Quiñones A, Reutzel T. Potential life-threatening events in schools involving rescue inhalers, epinephrine autoinjectors, and
 glucagon delivery devices: Reports from school nurses. The Journal of School Nursing. 2012;28(1):47-55.

5. Allensworth DD, Bradley B. Guidelines for adolescent preventive services: a role for the school nurse. Journal of school health. 1996;66(8):281-5.

6. Allison VL, Nativio DG, Mitchell AM, Ren D, Yuhasz J. Identifying symptoms of depression and anxiety in students in the school setting. The Journal of School
 Nursing. 2014;30(3):165-72.

7. Amillategui B, Calle J, Alvarez M, Cardiel M, Barrio R. Identifying the special needs of children with Type 1 diabetes in the school setting. An overview of parents’
 perceptions. Diabetic Medicine. 2007;24(10):1073-9.

8. Anderson J. The changing role of school nurses--one state's experience. The Journal of school nursing: the official publication of the National Association of School
 Nurses. 1994;10(3):22-6.

9. Antonelli RC, Antonelli DM. Providing a medical home: the cost of care coordination services in a community-based, general pediatric practice. Pediatrics.
 2004;113(5 Suppl):1522-8. Epub 2004/05/04. PubMed PMID: 15121921.

10. Antonelli RC, McAllister JW, Popp J. Making care coordination a critical component of the pediatric health system: a multidisciplinary framework. 2009.

11. Antonelli RC, Stille CJ, Antonelli DM. Care coordination for children and youth with special health care needs: a descriptive, multisite study of activities, personnel
 costs, and outcomes. Pediatrics. 2008;122(1):e209-16. Epub 2008/07/04. doi: 10.1542/peds.2007-2254. PubMed PMID: 18595966.

12. Aruda MM, Kelly M, Newinsky K. Unmet needs of children with special health care needs in a specialized day school setting. The Journal of School Nursing.
 2011;27(3):209-18.

13. Atherton C. On hand, in school. Community Pract. 2009;82(7):32-3. Epub 2009/07/25. PubMed PMID: 19626752.

14. Attwood M, Meadows S, Stallard P, Richardson T. Universal and targeted computerised cognitive behavioural therapy (Think, Feel, Do) for emotional health in
 schools: results from two exploratory studies. Child Adolesc Ment Health. 2012;17(3):173-8. Epub 2012/09/01. doi: 10.1111/j.1475-3588.2011.00627.x. PubMed
 PMID: 32847273.

15. author n. Initiating health programmes in schools and communities. Int Nurs Rev. 1997;44(3):76-8. Epub 1997/05/01. PubMed PMID: 9195252.

16. Badger F, Brown I. Primary schools' use and perceptions of the school nursing service. International Journal of Health Promotion and Education. 2005;43(3):92-6.

17. Bagnall P. School nurses' response to the measles vaccination campaign. Nursing times. 1995;91(40):38-9.

18. Bagnall P. Children's health: taking it seriously. MA Healthcare London; 1997.

19. Baisch MJ, Lundeen SP, Murphy MK. Evidence‐based research on the value of school nurses in an urban school system. Journal of School Health. 2011;81(2):74-80.

20. Baker DL, Hebbeler K, Davis-Alldritt L, Anderson LS, Knauer H. School health services for children with special health care needs in California. The Journal of
 School Nursing. 2015;31(5):318-25.

21. Bannink R, Broeren S, Joosten-van Zwanenburg E, van As E, van de Looij-Jansen P, Raat H. Effectiveness of a web-based tailored intervention (E-health4Uth) and
 consultation to promote adolescents’ health: randomized controlled trial. Journal of medical Internet research. 2014;16(5):e143.

22. Barnard-Brak L, Stevens T, Carpenter J. Care Coordination with Schools: The Role of Family-Centered Care for Children with Special Health Care Needs. Matern
 Child Health J. 2017;21(5):1073-8. doi: https://dx.doi.org/10.1007/s10995-016-2203-x. PubMed PMID: 28144766.

23. Barrett JC. A school-based care management service for children with special needs. Family & Community Health. 2000;23(2):36-42.

24. Bartfay WJ, Bartfay E. Promoting health in schools through a board game. Western journal of nursing research. 1994;16(4):438-46.

25. Bednarz P. The Omaha System: a model for describing school nurse case management. The Journal of school nursing: the official publication of the National
 Association of School Nurses. 1998;14(3):24-30.

26. Bergren MD. The feasibility of collecting school nurse data. The Journal of School Nursing. 2016;32(5):337-46.

27. Betz CL, Redcay G. Dimensions of the transition service coordinator role. Journal for Specialists in Pediatric Nursing. 2005;10(2):49-59.

28. Bhardwa S. Mental health in young people. Independent Nurse. 2013;6.

29. Blaakman SW, Cohen A, Fagnano M, Halterman JS. Asthma medication adherence among urban teens: a qualitative analysis of barriers, facilitators and experiences
 with school-based care. Journal of Asthma. 2014;51(5):522-9.

30. Blackwell LS, Robinson AF, Proctor MR, Taylor AM. Same care, different populations: return-to-learn practices following concussion in primary and secondary
 schools. Journal of child neurology. 2017;32(3):327-33.

31. Bolton P. School entry screening by the school nurse. Health visitor. 1994;67(4):135-6.

32. Bonaiuto M. School nurses' competence in caring for students who depend on medical technology. The Journal of school nursing: the official publication of the
 National Association of School Nurses. 1995;11(4):21-2, 4.

33. Bonaiuto MM. School nurse case management: Achieving health and educational outcomes. The Journal of School Nursing. 2007;23(4):202-9.

34. Bonny AE, Britto MT, Klostermann BK, Hornung RW, Slap GB. School disconnectedness: Identifying adolescents at risk. Pediatrics. 2000;106(5):1017-21.

35. Bonsergent E, Agrinier N, Thilly N, Tessier S, Legrand K, Lecomte E, et al. Overweight and obesity prevention for adolescents: a cluster randomized controlled trial
 in a school setting. Am J Prev Med. 2013;44(1):30-9. Epub 2012/12/21. doi: 10.1016/j.amepre.2012.09.055. PubMed PMID: 23253647.

36. Bonsergent E, Thilly N, Legrand K, Agrinier N, Tessier S, Lecomte E, et al. Process evaluation of a school-based overweight and obesity screening strategy in
 adolescents. Glob Health Promot. 2013;20(2 Suppl):76-82. Epub 2013/05/25. doi: 10.1177/1757975913483330. PubMed PMID: 23678500.

37. Borawski EA, Tufts KA, Trapl ES, Hayman LL, Yoder LD, Lovegreen LD. Effectiveness of health education teachers and school nurses teaching sexually transmitted
 infections/human immunodeficiency virus prevention knowledge and skills in high school. Journal of School Health. 2015;85(3):189-96.

38. Boyer‐Chuanroong L, Deaver P. Meeting the preteen vaccine law: a pilot program in urban middle schools. Journal of School Health. 2000;70(2):39-44.

39. Bradley BJ. The school nurse as health educator. Journal of School Health. 1997;67(1):3-8.

40. Bradley BJ. Establishing a research agenda for school nursing. Journal of School Health. 1998;68(2):53-61.

41. Brindis CD, Sanghvi R, Melinkovich P, Kaplan DW, Ahlstrand KR, MPH SLP. Redesigning a school health workforce for a new health care environment: Training
 school nurses as nurse practitioners. Journal of school health. 1998;68(5):179-83.

42. Brosnan C. Long-term results of an elementary sexuality program. Pediatric nursing. 1987;13(2):130-1.

43. Brother N. School nursing and student assistance: a natural partnership. The Journal of school nursing: the official publication of the National Association of School
 Nurses. 1998;14(1):32-5.

44. Broussard L. Empowerment in school nursing practice: A grounded theory approach. The Journal of School Nursing. 2007;23(6):322-8.

45. Brustrom J, Thibadeau J, John L, Liesmann J, Rose S. Care coordination in the spina bifida clinic setting: current practice and future directions. Journal of Pediatric
 Health Care. 2012;26(1):16-26.

46. Bruzzese JM, Evans D, Wiesemann S, Pinkett-Heller M, Levison MJ, Du Y, et al. Using school staff to establish a preventive network of care to improve elementary
 school students' control of asthma. J Sch Health. 2006;76(6):307-12. Epub 2006/08/22. doi: 10.1111/j.1746-1561.2006.00118.x. PubMed PMID: 16918861.

47. Bryan DS, Cook TS. Redirection of school nursing services in culturally deprived neighborhoods. American Journal of Public Health and the Nations Health.
 1967;57(7):1164-76.

48. Bucher L, Dryer C, Hendrix E, Wong N. Statewide assessment of school‐age children with asthma in Delaware. Journal of school health. 1998;68(7):276-81.

49. Buckland L, Rose J, Greaves C. New roles for school nurses: Preventing exclusion. Community Practitioner. 2005;78(1):16.

50. Buckland L, Rose J, Greaves C. Making a difference to families: tackling challenging behaviour. Community Practitioner. 2005;78(2):50-5. PubMed PMID:
 106643542. Language: English. Entry Date: 20050610. Revision Date: 20150820. Publication Type: Journal Article.

51. Butler R. 'School Health Matters': a web-based health resource for the school community. Community Practitioner. 2013;86(10):30-2. PubMed PMID: 102831946.
 Language: English. Entry Date: 20150602. Revision Date: 20150602. Publication Type: Article.

52. Cady RG, Kelly AM, Finkelstein SM, Looman WS, Garwick AW. Attributes of advanced practice registered nurse care coordination for children with medical
 complexity. J Pediatr Health Care. 2014;28(4):305-12. Epub 2013/08/31. doi: 10.1016/j.pedhc.2013.06.005. PubMed PMID: 23988611; PubMed Central PMCID:
 PMCPMC3935987.

53. Cameron R, Brown KS, Best JA, Pelkman CL, Madill CL, Manske SR, et al. Effectiveness of a social influences smoking prevention program as a function of
 provider type, training method, and school risk. American Journal of Public Health. 1999;89(12):1827-31.

54. Carpenter LM, Lachance L, Wilkin M, Clark NM. Sustaining school‐based asthma interventions through policy and practice change. Journal of school health.
 2013;83(12):859-66.

55. Carter B. Children's health care: a new era or more rhetoric? Journal of Child Health Care. 1997;1(4):161-2.

56. Chally PS. An eating disorders prevention program. Journal of Child and Adolescent Psychiatric Nursing. 1998;11(2):51-60.

57. Chase E, Chalmers H, Thomas F, Hollingworth K, Aggleton P. Shifting policies and enduring themes in school nursing. British Journal of School Nursing.
 2010;5(10):492-500.

58. Chen SPC, Fitzgerald MC, DeStefano LM, Chen EH. Effects of a school nurse prenatal counseling program. Public Health Nursing. 1991;8(4):212-8.

59. Chilvers J. Implementation of a Facebook page by school nurses. Community Practitioner. 2011;84(4):33-5. PubMed PMID: 104851193. Language: English. Entry
 Date: 20110406. Revision Date: 20150820. Publication Type: Journal Article. Journal Subset: Blind Peer Reviewed.

60. Chokshi NY, Patel D, Davis CM. Long-term increase in epinephrine availability associated with school nurse training in food allergy. The Journal of Allergy and
 Clinical Immunology: In Practice. 2015;3(1):128-30.

61. Christiansen SC, Martin SB, Schleicher NC, Koziol JA, Mathews KP, Zuraw BL. Evaluation of a school-based asthma education program for inner-city children.
 Journal of Allergy and Clinical Immunology. 1997;100(5):613-7.

62. Cicutto L, To T, Murphy S. A randomized controlled trial of a public health nurse‐delivered asthma program to elementary schools. Journal of School Health.
 2013;83(12):876-84.

63. Clapp A. Reducing the rate of teenage pregnancy. Practice Nurse. 2009;37(2):32-5. PubMed PMID: 105450880. Language: English. Entry Date: 20090327. Revision
 Date: 20171230. Publication Type: Journal Article.

64. Clarke ML. Out of the wilderness and into the fold: the school nurse and child protection. Child Abuse Review: Journal of the British Association for the Study and
 Prevention of Child Abuse and Neglect. 2000;9(5):364-74.

65. Clausson E, Berg A. Family intervention sessions: one useful way to improve schoolchildren's mental health. Journal of family nursing. 2008;14(3):289-313.

66. Coates M. School nursing, a priority for child-centred public health. British Journal of School Nursing. 2011;6(9):439-43.

67. Coleman J, Hawkins W. The changing role of the nurse: an alternative to elimination. The Journal of school health. 1970;40(3):121-2.

68. Costante C. Supporting Student Success: School Nurses Make a Difference. NASN President. The Journal of school nursing: the official publication of the National
 Association of School Nurses. 1996;12(3):4-6.

69. Cox E, Fritz K, Hansen K, Brown R, Rajamanickam V, Wiles K, et al. Development and Validation of PRISM: A Survey Tool to Identify Diabetes Self-Management
 Barriers.(Problem Recognition in Illness Self-Management) Copyright: Elsevier Ireland Ltd. 2014.

70. Crickmore K, Jones A, Engelke MK, Mott JA. Managing pediatric asthma. Health Forum J. 2002;45(6):24-30. Epub 2003/01/01. PubMed PMID: 12506792.

71. Davies A. Enabling young people to talk about health and wellbeing. British Journal of School Nursing. 2012;7(10):512-3. doi: 10.12968/bjsn.2012.7.10.512.
 PubMed PMID: 108023277. Language: English. Entry Date: 20130204. Revision Date: 20200708. Publication Type: Journal Article.

72. Davis C. A lesson in quitting. Nursing Standard. 2007;21(29):18-9. PubMed PMID: 106291093. Language: English. Entry Date: 20070525. Revision Date: 20150711.
 Publication Type: Journal Article.

73. Davis WS, Varni SE, Barry SE, Frankowski BL, Harder VS. Increasing immunization compliance by reducing provisional admittance. The Journal of School
 Nursing. 2016;32(4):246-57.

74. DeSocio J, Stember L, Schrinsky J. Teaching children about mental health and illness: A school nurse health education program. The Journal of school nursing.
 2006;22(2):81-6.

75. Dixon V. Teenage pregnancy: Identifying young people aspiring or ambivalent to parenthood. British Journal of School Nursing. 2014;9(1):38-44.

76. Dodds E. Are sexual health services in schools adequate? British Journal of School Nursing. 2011;6(8):397-403.

77. Doggett M-A, Faulkner A, Farrow S, Shelley A. School nurses: constraints and opportunities for the future. Journal of the Royal Society of Health. 1992;112(2):84-7.

78. DoH. Saving Lives: Our Healthier Nation. The Stationery Office London; 1999.

79. Downie J, Chapman R, Orb A, Juliff D. The everyday realities of the multi-dimensional role of the high school community nurse. Australian Journal of Advanced
 Nursing, The. 2002;19(3):15-24.

80. Driscoll KA, Volkening LK, Haro H, Ocean G, Wang Y, Jackson CC, et al. Are children with type 1 diabetes safe at school? Examining parent perceptions. Pediatric
 Diabetes. 2015;16(8):613-20.

81. Eisbach SS, Driessnack M. Am I sure I want to go down this road? Hesitations in the reporting of child maltreatment by nurses. Journal for specialists in pediatric
 nursing. 2010;15(4):317-23.

82. Engelke MK, Guttu M, Warren MB. Defining, delivering, and documenting the outcomes of case management by school nurses. J Sch Nurs. 2009;25(6):417-26. Epub
 2009/09/25. doi: 10.1177/1059840509347377. PubMed PMID: 19776226.

83. Engelke KM, Guttu M, Warren MB, Swanson M. School nurse case management for children with chronic illness: health, academic, and quality of life outcomes. J
 Sch Nurs. 2008;24(4):205-14. Epub 2008/09/02. doi: 10.1177/1059840508319929. PubMed PMID: 18757353.

84. Engelke MK, Swanson M, Guttu M. Process and outcomes of school nurse case management for students with asthma. The Journal of School Nursing.
 2014;30(3):196-205.

85. Engelke MK, Swanson M, Guttu M, Warren MB, Lovern S. School nurses and children with diabetes: A descriptive study. North Carolina Medical Journal.
 2011;72(5):351-8.

86. Engh LK, Eriksson U-B. The school nurse’s ability to detect and support abused children: A trust-creating process. The Journal of School Nursing. 2015;31(5):353-62

87. Engh LK, Rahm G, Eriksson U-B. School nurses avoid addressing child sexual abuse. The Journal of school nursing. 2017;33(2):133-42.

88. Fagan R. Health of the Nation targets: where school nurses find constraints on achievement. Nursing standard (Royal College of Nursing (Great Britain): 1987).
1995;9(48):36-40.

89. Ferson M, Fitzsimmons G, Christie D, Woollett H. School health nurse interventions to increaseimmunisation uptake in school entrants. Public Health.
 1995;109(1):25-9.

90. Few C. Alliances in school sex education: teachers and school nurses' views. Health visitor. 1996;69(6):220-3.

91. Foster LS, Keele R. Implementing an over-the-counter medication administration policy in an elementary school. The Journal of school nursing. 2006;22(2):108-13.

92. France J. New texting service for teenagers has all-round benefits. Nursing Standard. 2013;28(5):13.

93. Francisco B, Rood T, Nevel R, Foreman P, Homan S. Peer reviewed: Teaming up for asthma control: EPR-3 compliant school program in Missouri is effective and
 cost-efficient. Preventing chronic disease. 2017;14.

94. Fryer Jr G, Igoe J. A relationship between availability of school nurses and child well-being. The Journal of school nursing: the official publication of the National
 Association of School Nurses. 1995;11(3):12-8.

95. Fryer Jr GE, Igoe JB. Functions of school nurses and health assistants in US school health programs. Journal of School Health. 1996;66(2):55-8.

96. Gaffrey E, Bergren M. School health services and managed care: a unique partnership for child health. The Journal of school nursing: the official publication of the
 National Association of School Nurses. 1998;14(4):5-12, 4.

97. Garwick AW, Svavarsdóttir EK, Seppelt AM, Looman WS, Anderson LS, Örlygsdóttir B. Development of an international school nurse asthma care coordination
 model. Journal of advanced nursing. 2015;71(3):535-46.

98. Gilman S, Williamson MC, Nader PR, Dale S, McKevitt R. Task differentiation among elementary, middle and high school nurses. Journal of School Health.
 1979;49(6):313-6.

99. Gordon JB, Colby HH, Bartelt T, Jablonski D, Krauthoefer ML, Havens P. A tertiary care-primary care partnership model for medically complex and fragile children
 and youth with special health care needs. Arch Pediatr Adolesc Med. 2007;161(10):937-44. Epub 2007/10/03. doi: 10.1001/archpedi.161.10.937. PubMed PMID:
 17909136.

100. Gottfried MA. Understanding the institutional-level factors of urban school quality. Teachers College Record. 2012;114(12):1-32.

101. Grandahl M, Rosenblad A, Stenhammar C, Tydén T, Westerling R, Larsson M, et al. School-based intervention for the prevention of HPV among adolescents: a
 cluster randomised controlled study. BMJ open. 2016;6(1).

102. Grudnikoff E, Taneli T, Correll CU. Characteristics and disposition of youth referred from schools for emergency psychiatric evaluation. European child & adolescent
 psychiatry. 2015;24(7):731-43.

103. Guttu M, Engelke MK, Swanson M. Does the school nurse‐to‐student ratio make a difference? Journal of School Health. 2004;74(1):6-9.

104. Hackett AJ. The role of the school nurse in child protection. Community Practitioner. 2013;86(12).

105. Halterman JS, Szilagyi PG, Fisher SG, Fagnano M, Tremblay P, Conn KM, et al. Randomized controlled trial to improve care for urban children with asthma: results
 of the School-Based Asthma Therapy trial. Archives of pediatrics & adolescent medicine. 2011;165(3):262-8.

106. Hanson TK, Aleman M, Hart L, Yawn B. Increasing availability to and ascertaining value of asthma action plans in schools through use of technology and community
 collaboration. Journal of School Health. 2013;83(12):915-20.

107. Harrell JS, McMurray RG, Gansky SA, Bangdiwala SI, Bradley CB. A public health vs a risk-based intervention to improve cardiovascular health in elementary
 school children: the Cardiovascular Health in Children Study. American journal of public health. 1999;89(10):1529-35.

108. Harrington CB, Langhans E, Shelef DQ, Savitz M, Whitmore C, Teach SJ. A pilot randomized trial of school-based administration of inhaled corticosteroids for at-
 risk children with asthma. J Asthma. 2018;55(2):145-51. Epub 2017/06/09. doi: 10.1080/02770903.2017.1323915. PubMed PMID: 28594249.

109. Hawkins JW, Hayes ER, Corliss CP. School Nursing in America—1902‐1994: A Return to Public Health Nursing. Public Health Nursing. 1994;11(6):416-25.

110. Hawthorne A, Shaibi G, Gance-Cleveland B, McFall S. Grand Canyon Trekkers: school-based lunchtime walking program. The Journal of School Nursing.
 2011;27(1):43-50.

111. Hayes‐Bohn R, Neumark‐Sztainer D, Mellin A, Patterson J. Adolescent and parent assessments of diabetes mellitus management at school. Journal of School Health.
 2004;74(5):166-9.

112. Hayter M, Owen J, Cooke J. Developing and establishing school-based sexual health services: issues for school nursing practice. The Journal of School Nursing.
 2012;28(6):433-41.

113. Hellems MA, Clarke WL. Safe at school: a Virginia experience. Diabetes Care. 2007;30(6):1396-8.

114. Hendershot C, Dake JA, Price JH, Lartey GK. Elementary school nurses’ perceptions of student bullying. The Journal of School Nursing. 2006;22(4):229-36.

115. Hendershot C, Telljohann SK, Price JH, Dake JA, Mosca NW. Elementary school nurses’ perceptions and practices regarding body mass index measurement in
 school children. The Journal of School Nursing. 2008;24(5):298-309.

116. Henry S. A nursing informatics approach for addressing national issues and priorities for school nursing services. The Journal of school nursing: the official
 publication of the National Association of School Nurses. 1997;13(4):39-42.

117. Hill NJ, Hollis M. Teacher time spent on student health issues and school nurse presence. The Journal of School Nursing. 2012;28(3):181-6.

118. Houck GM, Darnell S, Lussman S. A support group intervention for at-risk female high school students. The Journal of School Nursing. 2002;18(4):212-8.

119. Houghton A, Egan S, Archinal G, Bradley O, Azam N. Selective medical examination at school entry: should we do it, and if so how? Journal of Public Health.
 1992;14(2):111-6.

120. Hoying J, Melnyk BM. COPE: a pilot study with urban-dwelling minority sixth-grade youth to improve physical activity and mental health outcomes. The Journal of
 School Nursing. 2016;32(5):347-56.

121. Igoe JB. School nursing. Nurs Clin North Am. 1994;29(3):443-58. Epub 1994/09/01. PubMed PMID: 8090640.

122. Izquierdo R, Morin PC, Bratt K, Moreau Z, Meyer S, Ploutz-Snyder R, et al. School-centered telemedicine for children with type 1 diabetes mellitus. J Pediatr.
 2009;155(3):374-9. Epub 2009/05/26. doi: 10.1016/j.jpeds.2009.03.014. PubMed PMID: 19464030.

123. Janevic MR, Stoll S, Wilkin M, Song PX, Baptist A, Lara M, et al. Pediatric asthma care coordination in underserved communities: a quasiexperimental study.
 American journal of public health. 2016;106(11):2012-8.

124. Johansson A, Ehnfors M. Mental health-promoting dialogue of school nurses from the perspective of adolescent pupils. Vård i norden. 2006;26(4):10-9.

125. Johnston CA, Moreno JP, El‐Mubasher A, Gallagher M, Tyler C, Woehler D. Impact of a school‐based pediatric obesity prevention program facilitated by health
 professionals. Journal of School Health. 2013a;83(3):171-81.

126. Johnston CA, Moreno JP, Gallagher MR, Wang J, Papaioannou MA, Tyler C, et al. Achieving long-term weight maintenance in Mexican-American adolescents with
 a school-based intervention. Journal of Adolescent Health. 2013b;53(3):335-41.

127. Jones L, McEwen A. Reducing secondhand smoke exposure at home. British Journal of School Nursing. 2012;7(8):389-93.

128. Jordan KS, MacKay P, Woods SJ. Child maltreatment: Optimizing recognition and reporting by school nurses. NASN school nurse. 2017;32(3):192-9.

129. Kelly N, Greaves C, Buckland L, Rose J. School nurses: well placed to address challenging behaviour. Community Practitioner. 2005;78(3):88.

130. Kemper AR, Helfrich A, Talbot J, Patel N. Outcomes of an elementary school-based vision screening program in North Carolina. The Journal of School Nursing.
 2012;28(1):24-30.

131. Khubchandani J, Telljohann SK, Price JH, Dake JA, Hendershot C. Providing assistance to the victims of adolescent dating violence: A national assessment of school
 nurses' practices. Journal of school health. 2013;83(2):127-36.

132. Kim RE, Becker KD, Stephan SH, Hakimian S, Apocada D, Escudero PV, et al. Connecting students to mental health care: Pilot findings from an engagement
 program for school nurses. Advances in school mental health promotion. 2015;8(2):87-103.

133. Kimel L. Handwashing education can decrease illness absenteeism. The Journal of school nursing: the official publication of the National Association of School
 Nurses. 1996;12(2):14-6, 8.

134. Kirchofer G, Telljohann SK, Price JH, Dake JA, Ritchie M. Elementary school parents’/guardians’ perceptions of school health service personnel and the services
 they provide. Journal of School Health. 2007;77(9):607-14.

135. Knauer H, Baker DL, Hebbeler K, Davis-Alldritt L. The mismatch between children’s health needs and school resources. The Journal of School Nursing.
 2015;31(5):326-33.

136. Kornguth ML. Preventing school absences due to illness. Journal of School Health. 1991;61(6):272-5.

137. Krenitsky-Korn S. High school students with asthma: attitudes about school health, absenteeism, and its impact on academic achievement. Pediatric nursing.
 2011;37(2).

138. Kroshus E, Fischer AN, Nichols JF. Assessing the awareness and behaviors of US high school nurses with respect to the female athlete triad. The Journal of School
 Nursing. 2015;31(4):272-9.

139. Krug EG, Brener ND, Dahlberg LL, Ryan GW, Powell KE. The impact of an elementary school-based violence prevention program on visits to the school nurse.
 American journal of preventive medicine. 1997;13(6):459-63.

140. Lamb J, Albrecht S, Sereika S. Consideration of factors prior to implementing a smoking cessation program. The Journal of school nursing: the official publication of
 the National Association of School Nurses. 1998b;14(1):14-9.

141. LAMB JM, Puskar KR, SEREIKA SM, CORCORAN M. School-based intervention to promote coping in rural teens. MCN: The American Journal of Maternal/Child
 Nursing. 1998a;23(4):187-94.

142. Land M, Barclay L. Nurses' contribution to child protection. Neonatal, paediatric and child health nursing. 2008;11(1):18-24.

143. Larsson B, Carlsson J. A school-based, nurse-administered relaxation training for children with chronic tension-type headache. Journal of Pediatric Psychology.
 1996;21(5):603-14.

144. Lazdowsky L, Rabner J, Caruso A, Kaczynski K, Gottlieb S, Mahoney E, et al. “Headache Tools to Stay in School”: Assessment, Development, and Implementation
 of an Educational Guide for School Nurses. Journal of School Health. 2016;86(9):645-52.

145. Lee J, Kubik MY. Child’s weight status and parent’s response to a school-based body mass index screening and parent notification program. The Journal of School
 Nursing. 2015;31(4):300-5.

146. Leff S, Bennett J. Audit of school entry health assessments: to maximise efficient use of health personnel at school entry assessments at 5 years. Public health.
 1996;110(5):289-92.

147. Lehmkuhl H, Nabors L. Children with diabetes: Satisfaction with school support, illness perceptions and HbA1c levels. Journal of Developmental and Physical
 Disabilities. 2008;20(2):101.

148. Levy M, Heffner B, Stewart T, Beeman G. The efficacy of asthma case management in an urban school district in reducing school absences and hospitalizations for
 asthma. J Sch Health. 2006;76(6):320-4. Epub 2006/08/22. doi: 10.1111/j.1746-1561.2006.00120.x. PubMed PMID: 16918863.

149. Lewis CC, Alford-Winston A, Billy-Kornas M, McCaustland MD, Tachman CP. Care management for children who are medically fragile/technology-dependent.
 Issues Compr Pediatr Nurs. 1992;15(2):73-91. Epub 1992/04/01. doi: 10.3109/01460869209078244. PubMed PMID: 1308008.

150. Liberatos P, Leone J, Craig AM, Frei EM, Fuentes N, Harris IM. Challenges of asthma management for school nurses in districts with high asthma hospitalization
 rates. Journal of School Health. 2013;83(12):867-75.

151. Lightfoot J, Bines W. Working to keep school children healthy: the complementary roles of school staff and school nurses. Journal of Public Health. 2000;22(1):74-
 80.

152. Lightfoot J, Bines W. Keeping children healthy: role of the school nurse. Nursing times. 1998;94(21):65-8.

153. Lindeke LL, Leonard BJ, Presler B, Garwick A. Family-centered care coordination for children with special needs across multiple settings. J Pediatr Health Care.
 2002;16(6):290-7. Epub 2002/11/19. PubMed PMID: 12436098.

154. Liptzin DR, Gleason MC, Cicutto LC, Cleveland CL, White MK, Faino AV, et al. Developing, implementing, and evaluating a school-centered asthma program: step-
 up asthma program. The Journal of Allergy and Clinical Immunology: In Practice. 2016;4(5):972-9. e1.

155. Long G, Whitman C, Johansson M, Williams C, Tuthill R. Evaluation of a school health program directed to children with history of high absence. American journal
 of public health. 1975;65(4):388-93.

156. Looman WS, Presler E, Erickson MM, Garwick AW, Cady RG, Kelly AM, et al. Care coordination for children with complex special health care needs: the value of
 the advanced practice nurse's enhanced scope of knowledge and practice. J Pediatr Health Care. 2013;27(4):293-303. Epub 2012/05/09. doi:
 10.1016/j.pedhc.2012.03.002. PubMed PMID: 22560803; PubMed Central PMCID: PMCPMC3433641.

157. Lunney M. The significance of nursing classification systems to school nursing. The Journal of school nursing: the official publication of the National Association of
 School Nurses. 1996;12(2):35-7.

158. Lunney M, Cavendish R, Luise B, Richardson K. Relevance of NANDA and health promotion diagnoses to school nursing. National Association of School Nurses.
 The Journal of school nursing: the official publication of the National Association of School Nurses. 1997;13(5):16-22.

159. Lunstead J, Weitzman ER, Kaye D, Levy S. Screening and brief intervention in high schools: School nurses' practices and attitudes in Massachusetts. Substance
 abuse. 2017;38(3):257-60.

160. Luthy KE, Thorpe A, Dymock LC, Connely S. Evaluation of an intervention program to increase immunization compliance among school children. The Journal of
 School Nursing. 2011;27(4):252-7.

161. Lynch E. School of thought. Nursing Standard. 2008;22(27).

162. Magalnick H, Mazyck D. Role of the school nurse in providing school health services. Pediatrics. 2008;121(5):1052-6. Epub 2008/05/03. doi: 10.1542/peds.2008-
 0382. PubMed PMID: 18450912.

163. Magee JA, Kenney DM, Mullin E. Efficacy of and advocacy for postural screening in public schools. Orthopaedic Nursing. 2012;31(4):232-5.

164. Major DA, Clarke SM, Cardenas RA, Taylor-Fishwick JC, Kelly CS, Butterfoss FD. Providing asthma care in elementary schools: Understanding barriers to
 determine best practices. Family & Community Health. 2006;29(4):256-65.

165. Maunder Y. My day as a school nurse. Education & Health. 2004;22(1):8-10. PubMed PMID: 106665543. Language: English. Entry Date: 20041119. Revision Date:
 20150711. Publication Type: Journal Article. Journal Subset: Biomedical.

166. McKaig C, Hindi‐Alexander M, Myers TR, Castiglia P. Implementation of the school nurse practitioner role: Barriers and facilitators. Journal of School Health.
 1984;54(1):21-3.

167. Medaglia F, Knorr RS, Condon SK, Charleston AC. School-based pediatric asthma surveillance in Massachusetts from 2005 to 2009. Journal of School Health.
 2013;83(12):907-14. doi: https://dx.doi.org/10.1111/josh.12109. PubMed PMID: 24261525.

168. Melin A, Lenner RA. Prevention of further weight gain in overweight school children, a pilot study. Scandinavian journal of caring sciences. 2009;23(3):498-505.

169. Mickel CF, Shanovich KK, Evans MD, Jackson DJ. Evaluation of a school-based asthma education protocol: iggy and the inhalers. The Journal of School Nursing.
 2016;33(3):189-97.

170. Moricca ML, Grasska MA, M BM, Morphew T, Weismuller PC, Galant SP. School asthma screening and case management: attendance and learning outcomes. J Sch
 Nurs. 2013;29(2):104-12. Epub 2012/07/17. doi: 10.1177/1059840512452668. PubMed PMID: 22797976.

171. Morris P, Baker D, Belot C, Edwards A. Preparedness for students and staff with anaphylaxis. Journal of School Health. 2011;81(8):471-6.

172. Morrison-Sandberg LF, Kubik MY, Johnson KE. Obesity prevention practices of elementary school nurses in Minnesota: Findings from interviews with licensed
 school nurses. The Journal of School Nursing. 2011;27(1):13-21.

173. Morton JL, Schultz AA. Healthy hands: use of alcohol gel as an adjunct to handwashing in elementary school children. The Journal of School Nursing.
 2004;20(3):161-7.

174. Muggeo MA, Stewart CE, Drake KL, Ginsburg GS. A school nurse-delivered intervention for anxious children: An open trial. School Mental Health. 2017;9(2):157-
 71.

175. Murray R. Introduction to the American Academy of Pediatrics policy statement. SAGE Publications Sage CA: Los Angeles, CA; 2008.

176. Nauta C, Byrne C, Wesley Y. School nurses and childhood obesity: An investigation of knowledge and practice among school nurses as they relate to childhood
 obesity. Issues in Comprehensive Pediatric Nursing. 2009;32(1):16-30.

177. Nguyen TM, Mason KJ, Sanders CG, Yazdani P, Heptulla RA. Targeting blood glucose management in school improves glycemic control in children with poorly
 controlled type 1 diabetes mellitus. The Journal of pediatrics. 2008;153(4):575-8.

178. Noyes K, Bajorska A, Fisher S, Sauer J, Fagnano M, Halterman JS. Cost-effectiveness of the School-Based Asthma Therapy (SBAT) program. Pediatrics. 2013;131(3):e709-17. Epub 2013/02/13. doi: 10.1542/peds.2012-1883. PubMed PMID: 23400614; PubMed Central PMCID: PMCPMC3581846.

179. Nutbeam D, Farley P, Smith C. England and Wales: perspectives in school health. Journal of School Health. 1990;60(7):318-23.

180. O'Donnell NL, Alles WF. School Nurse Demonstrates that Mini‐Grant Funding Can Improve Elementary Nutrition Education. Journal of School Health. 1983;53(5):316-9.

181. O'Toole AW, O'Toole R, Webster SW, Lucal B. Nurses' diagnostic work on possible physical child abuse. Public health nursing. 1996;13(5):337-44.

182. Oda DS. Is school nursing really the" invisible practice?". Journal of School Health. 1992;62(3):112-4.

183. Olowokere A, Okanlawon F. The effects of a school-based psychosocial intervention on resilience and health outcomes among vulnerable children. The Journal of School Nursing. 2014;30(3):206-15.

184. Paavilainen E, Åstedt‐Kurki P, Paunonen M. School nurses’ operational modes and ways of collaborating in caring for child abusing families in Finland. Journal of Clinical Nursing. 2000;9(5):742-50.

185. Paavilainen E, Helminen M, Flinck A, Lehtumaki L. How public health nurses identify and intervene in child maltreatment based on the national clinical guideline. Nurs Res Pract. 2014;2014:425460. Epub 2014/12/17. doi: 10.1155/2014/425460. PubMed PMID: 25505986; PubMed Central PMCID: PMCPMC4253703.

186. Paavilainen E, Tarkka MT. Definition and identification of child abuse by Finnish public health nurses. Public health nursing. 2003;20(1):49-55.

187. Pakieser RA, Starr DK, LeBaugh D. Nebraska School Nurses Identify Emotional Maltreatment of School‐Age Children: A Replication of an Ohio Study. Journal for Specialists in Pediatric Nursing. 1998;3(4):137-.

188. Pbert L, Druker S, Barton B, Schneider KL, Olendzki B, Gapinski MA, et al. A school‐based program for overweight and obese adolescents: a randomized controlled trial. Journal of School Health. 2016;86(10):699-708.

189. Pbert L, Druker S, Gapinski MA, Gellar L, Magner R, Reed G, et al. A school nurse‐delivered intervention for overweight and obese adolescents. Journal of School Health. 2013;83(3):182-93.

190. Peckover S, Trotter F. Keeping the focus on children: the challenges of safeguarding children affected by domestic abuse. Health & social care in the community. 2015;23(4):399-407.

191. Peery AI, Engelke MK, Swanson MS. Parent and teacher perceptions of the impact of school nurse interventions on children’s self-management of diabetes. The Journal of School Nursing. 2012;28(4):268-74.

192. Pennington N, Delaney E. The number of students sent home by school nurses compared to unlicensed personnel. J Sch Nurs. 2008;24(5):290-7. Epub 2008/10/23. doi: 10.1177/1059840508322382. PubMed PMID: 18941153.

193. Perry CS, Toole KA. Impact of school nurse case management on asthma control in school-aged children. Journal of School Health. 2000;70(7):303-.

194. Persaud DI, Barnett SE, Weller SC, Baldwin CD, Niebuhr V, McCormick DP. An asthma self-management program for children, including instruction in peak flow monitoring by school nurses. Journal of Asthma. 1996;33(1):37-43.

195. Petitgout JM, Pelzer DE, McConkey SA, Hanrahan K. Development of a hospital-based care coordination program for children with special health care needs. J Pediatr Health Care. 2013;27(6):419-25. Epub 2012/05/12. doi: 10.1016/j.pedhc.2012.03.005. PubMed PMID: 22575784.

196. Powell SB, Engelke MK, Neil JA. Seizing the moment: Experiences of school nurses caring for students with overweight and obesity. The Journal of School Nursing. 2018;34(5):380-9.

197. Presler B. Care coordination for children with special health care needs. Orthop Nurs. 1998;17(2 Suppl):45-51. Epub 1998/05/28. PubMed PMID: 9601413.

198. Pryjmachuk S, Graham T, Haddad M, Tylee A. School nurses’ perspectives on managing mental health problems in children and young people. Journal of Clinical Nursing. 2012;21(5‐6):850-9.

199. Pulcini JM, Marshall GD, Jr., Naveed A. Presence of food allergy emergency action plans in Mississippi. Ann Allergy Asthma Immunol. 2011;107(2):127-32. doi: https://dx.doi.org/10.1016/j.anai.2011.05.019. PubMed PMID: 21802020.

200. Putman-Casdorph H, Pinto S. Preliminary testing of an asthma distance education program (ADEP) for school nurses in Appalachia. The Journal of School Nursing. 2011;27(6):411-5.

201. Quaranta JE, Spencer GA. Barriers to asthma management as identified by school nurses. The Journal of School Nursing. 2016;32(5):365-73.

202. Quelly SB. Influence of perceptions on school nurse practices to prevent childhood obesity. The Journal of School Nursing. 2014;30(4):292-302.

203. Raible CA, Dick R, Gilkerson F, Mattern CS, James L, Miller E. School Nurse‐Delivered Adolescent Relationship Abuse Prevention. Journal of School Health. 2017;87(7):524-30.

204. Ramirez M, Harland K, Frederick M, Shepherd R, Wong M, Cavanaugh JE. Listen protect connect for traumatized schoolchildren: a pilot study of psychological first aid. BMC psychology. 2013;1(1):26.

205. Ramos MM, Greenberg C, Sapien R, Bauer‐Creegan J, Hine B, Geary C. Behavioral health emergencies managed by school nurses working with adolescents. Journal of school health. 2013;83(10):712-7.

206. Rasberry CN, Morris E, Lesesne CA, Kroupa E, Topete P, Carver LH, et al. Communicating with school nurses about sexual orientation and sexual health: perspectives of teen young men who have sex with men. The Journal of School Nursing. 2015;31(5):334-44.

207. Rebmann T, Elliott MB, Artman D, VanNatta M, Wakefield M. Impact of an Education Intervention on Missouri K‐12 School Disaster and Biological Event Preparedness. Journal of school health. 2016;86(11):794-802.

208. Reid J. Developing the role of the school nurse in public health. Health Education Journal. 1991;50(3):118-22.

209. Resnicow K, Allensworth D. Conducting a comprehensive school health program. Journal of School Health. 1996;66(2):59-63.

210. Rhodes DL, Draper M, Woolman K, Cox C. Practices and attitudes of Missouri school nurses regarding immunization records and select immunizations of graduating high school seniors. Journal of community health. 2017;42(5):872-7.

211. Rivkina V, Tapke DE, Cardenas LD, Harvey-Gintoft B, Whyte SA, Gupta RS. Identifying barriers to chronic disease reporting in Chicago Public Schools: a mixed-methods approach. BMC Public Health. 2014;14:1250. doi: https://dx.doi.org/10.1186/1471-2458-14-1250. PubMed PMID: 25481628.

212. Robbins LB, Pfeiffer KA, Maier KS, Lo Y-J, Wesolek SM. Pilot intervention to increase physical activity among sedentary urban middle school girls: a two-group pretest–posttest quasi-experimental design. The Journal of School Nursing. 2012;28(4):302-15.

213. Roden D. Proving the value of school nursing services. Health visitor. 1997;70:462-3.

214. Rodriguez E, Rivera DA, Perlroth D, Becker E, Wang NE, Landau M. School nurses' role in asthma management, school absenteeism, and cost savings: a demonstration project. J Sch Health. 2013;83(12):842-50. Epub 2013/11/23. doi: 10.1111/josh.12102. PubMed PMID: 24261518.

215. Rose DA, Chen SP, Souter CM. Development of an in-service education program by school nurses. J Community Health Nurs. 1987;4(3):171-8. Epub 1987/01/01. doi: 10.1207/s15327655jchn0403_7. PubMed PMID: 3650296.

216. Rose S. Nit nurse to nitty gritty: former school nurse Sharon Rose shares her views on the profession today and explains how she believes strong links with the whole team, including health visitors, would help to improve morale and protect the future health of young people. Community Practitioner. 2013;86(7):42-4.

217. Rosen BL, DiClemente R, Shepard AL, Wilson KL, Fehr SK. Factors associated with school nurses’ HPV vaccine attitudes for school-aged youth. Psychology, health & medicine. 2017;22(5):535-45.

218. Rosen BL, Goodson P, Thompson B, Wilson KL. School nurses' knowledge, attitudes, perceptions of role as opinion leader, and professional practice regarding human papillomavirus vaccine for youth. Journal of School Health. 2015;85(2):73-81.

219. Rote S. Losing sight of the future. Nursing times. 1997a;93(24):58-9.

220. Rustia J, Hartley R, Hansen G, Schulte D, Spielman L. Redefinition of school nursing practice: integrating the developmentally disabled. Journal of School Health. 1984;54(2):58-62.

221. Salend SJ, Mahoney S. Teaching Proper Health Habits to Mainstreamed Students Through Positive Reinforcement. Journal of School Health. 1982;52(9):539-42.

222. Salmon DA, Moulton LH, Omer SB, Chace LM, Klassen A, Talebian P, et al. Knowledge, attitudes, and beliefs of school nurses and personnel and associations with nonmedical immunization exemptions. Pediatrics. 2004;113(6):e552-e9.

223. Savage J, Goodall G. Making a drama out of teenage obesity. Nursing Standard. 2006;20(32):10-. doi: 10.7748/ns.20.32.10.s12. PubMed PMID: 106470841. Language: English. Entry Date: 20060714. Revision Date: 20200708. Publication Type: Journal Article.

224. Schaffer MA, Anderson LJ, Rising S. Public health interventions for school nursing practice. The Journal of School Nursing. 2016;32(3):195-208.

225. Schols MW, De Ruiter C, Öry FG. How do public child healthcare professionals and primary school teachers identify and handle child abuse cases? A qualitative study. BMC Public Health. 2013;13(1):1-16.

226. Schonfeld D. Talking with elementary school-age children about AIDS and death: principles and guidelines for school nurses. The Journal of school nursing: the official publication of the National Association of School Nurses. 1996;12(1):26-32.

227. Schroeder K, Jia H, Wang YC, Smaldone A. Implementation of a school nurse-led intervention for children with severe obesity in New York City schools. Journal of pediatric nursing. 2017;35:16-22.

228. Schwartz FL, Denham S, Heh V, Wapner A, Shubrook J. Experiences of children and adolescents with type 1 diabetes in school: Survey of children, parents, and schools. Diabetes Spectrum. 2010;23(1):47-55.

229. Scott LK, Hall LM. Reliability and validity of the acanthosis nigricans screening tool for use in elementary school-age children by school nurses. The Journal of School Nursing. 2012;28(6):442-7.

230. Seidenberg A. School nurses: Improve the reception and sharpen the image. Journal of School Health. 1984;54(9):363-5.

231. Sekhar DL, Zalewski TR, Ghossaini SN, King TS, Rhoades JA, Czarnecki B, et al. Pilot study of a high-frequency school-based hearing screen to detect adolescent hearing loss. Journal of medical screening. 2014;21(1):18-23.

232. Sekhar DL, Kraschnewski JL, Stuckey HL, Witt PD, Francis EB, Moore GA, et al. Opportunities and challenges in screening for childhood sexual abuse. Child abuse & neglect. 2018;85:156-63.

233. Sherman JB, Alexander MA, Gomez D, Marole P. Intervention program for obese school children. Journal of community health nursing. 1992;9(3):183-90.

234. Singer B. Perceptions of school nurses in the care of students with disabilities. The Journal of School Nursing. 2013;29(5):329-36.

235. Skelley JP, Luthin DR, Skelley JW, Kabagambe EK, Ashraf AP, Atchison JA. Parental perspectives of diabetes management in Alabama public schools. Southern medical journal. 2013;106(4):274-9.

236. Snyder AA, Minnick K, Anderson DE. Children from broken homes: visits to the school nurse. The Journal of school health. 1980;50(4):189-94.

237. Speroni KG, Earley C, Atherton M. Evaluating the effectiveness of the Kids Living Fit™ program: A comparative study. The Journal of school nursing. 2007;23(6):329-36.

238. Spina JL, McIntyre CL, Pulcini JA. An intervention to increase high school students’ compliance with carrying auto-injectable epinephrine: a MASNRN study. The Journal of School Nursing. 2012;28(3):230-7.

239. Splett PL, Erickson CD, Belseth SB, Jensen C. Evaluation and sustainability of the healthy learners asthma initiative. J Sch Health. 2006;76(6):276-82. Epub 2006/08/22. doi: 10.1111/j.1746-1561.2006.00112.x. PubMed PMID: 16918855.

240. Spratt J, Philip K, Shucksmith J, Kiger A, Gair D. ‘We are the ones that talk about difficult subjects’: nurses in schools working to support young people’s mental health. Pastoral Care in Education. 2010;28:131-44. doi: 10.1080/02643944.2010.482145.

241. Sprinks J. Nurse-led initiative focuses on parents to lessen health-related school absences. Nursing Children and Young People (through 2013). 2011;23(10):6.

242. Squires C. How can we make a difference? Delivering quality health education. British Journal of School Nursing. 2013;8(9):452-4.

243. Stallard P, Simpson N, Anderson S, Hibbert S, Osborn C. The FRIENDS emotional health programme: Initial findings from a school‐based project. Child and Adolescent Mental Health. 2007;12(1):32-7.

244. Stallard P, Simpson N, Anderson S, Goddard M. The FRIENDS emotional health prevention programme. European child & adolescent psychiatry. 2008;17(5):283-9.

245. Stalter AM, Chaudry RV, Polivka BJ. Regional differences as barriers to body mass index screening described by Ohio school nurses. Journal of School Health. 2011a;81(8):437-48.

246. Stalter AM, Kaylor M, Steinke JD, Barker RM. Parental perceptions of the rural school’s role in addressing childhood obesity. The Journal of School Nursing. 2011b;27(1):70-81.

247. Stang JS, Story M, Kalina B. School-based weight management services: perceptions and practices of school nurses and administrators. American Journal of Health Promotion. 1997;11(3):183-5.

248. Staudt AM, Alamgir H, Long DL, Inscore SC, Wood PR. Developing and Implementing a Citywide Asthma Action Plan: A Community Collaborative Partnership. Southern medical journal. 2015;108(12):710-4.

249. Steele RG, Wu YP, Cushing CC, Jensen CD. Evaluation of child health matters: a web-based tutorial to enhance school nurses’ communications with families about weight-related health. The Journal of School Nursing. 2013;29(2):151-60.

250. Stephenson C. Visits by elementary school children to the school nurse. Journal of School Health. 1983;53(10):594-9.

251. Streeting J. Observations from QK: personal reflections on being the full-time school nurse working within a multidisciplinary student support team at one London secondary school. Community Practitioner. 2010;83(4):38-40.

252. Swallow W, Roberts JC. An evidence-based project demonstrating increased school immunization compliance following a school nurse–initiated vaccine compliance strategy. The Journal of School Nursing. 2016;32(6):385-9.

253. Szychlinski C, Schmeissing KA, Fuleihan Z, Qamar N, Syed M, Pongracic JA, et al. Food allergy emergency preparedness in Illinois schools: rural disparity in guideline implementation. The Journal of Allergy and Clinical Immunology: In Practice. 2015;3(5):805-7. e8.

254. Taras H, Wright S, Brennan J, Campana J, Lofgren R. Impact of school nurse case management on students with asthma. J Sch Health. 2004;74(6):213-9. Epub 2004/10/08. doi: 10.1111/j.1746-1561.2004.tb07935.x. PubMed PMID: 15468525.

255. Taylor A, Lizzi M, Marx A, Chilkatowsky M, Trachtenberg SW, Ogle S. Implementing a care coordination program for children with special healthcare needs: partnering with families and providers. J Healthc Qual. 2013;35(5):70-7. Epub 2012/08/24. doi: 10.1111/j.1945-1474.2012.00215.x. PubMed PMID: 22913270.

256. Telljohann SK, Dake JA, Price JH. Effect of full-time versus part-time school nurses on attendance of elementary students with asthma. J Sch Nurs. 2004;20(6):331-4. Epub 2004/11/25. doi: 10.1177/10598405040200060701. PubMed PMID: 15560730.

257. Terry D, Patel AD, Cohen DM, Scherzer D, Kline J. Barriers to seizure management in schools: perceptions of school nurses. Journal of child neurology. 2016;31(14):1602-6.

258. Thompson J. School health services in the United States: a view from the United Kingdom. Journal of School Health. 1989;59(6):243-5.

259. Thomson H. A successful approach to reduce youth smoking in Leicestershire. British Journal of School Nursing. 2012;7(9):441-7.

260. Thurston C, Walker S. Experiences and training in delivering sexual health care. British Journal of School Nursing. 2011;6(6):289-93.

261. Toole K, Perry CS. Increasing immunization compliance. The Journal of School Nursing. 2004;20(4):203-8.

262. Triggle N. Helping Traveller families to brush up on health facts. Nursing Children and Young People (2014+). 2014;26(2):8.

263. Trivedi M, Patel J, Lessard D, Kremer T, Byatt N, Phipatanakul W, et al. School nurse asthma program reduces healthcare utilization in children with persistent asthma. J Asthma. 2017;55(10):1131-7. Epub 2017/12/06. doi: 10.1080/02770903.2017.1396473. PubMed PMID: 29206057; PubMed Central PMCID: PMCPMC5988937.

264. Tsacoyianis R. Indoor air pollutants and sick building syndrome: a case study and implications for the community health nurse. Public Health Nursing. 1997;14(1):58-75.

265. Tucker S, Lanningham-Foster LM. Nurse-led school-based child obesity prevention. The Journal of School Nursing. 2015;31(6):450-66.

266. Turner KM, Shield JP, Salisbury C. Practitioners' views on managing childhood obesity in primary care: a qualitative study. British Journal of General Practice. 2009;59(568):856-62.

267. Urbinati D, Steele P, Harter B, Harrell D. The evolution of the school nurse practitioner: past, present, and future. The Journal of school nursing: the official publication of the National Association of School Nurses. 1996;12(2):6-9.

268. Van Cura M. The relationship between school-based health centers, rates of early dismissal from school, and loss of seat time. J Sch Health. 2010;80(8):371-7. Epub 2010/07/14. doi: 10.1111/j.1746-1561.2010.00516.x. PubMed PMID: 20618619.

269. Van Roeyen LS. Management of pediatric asthma at home and in school. Nurs Clin North Am. 2013;48(1):165-75. Epub 2013/03/08. doi: 10.1016/j.cnur.2012.12.006. PubMed PMID: 23465450.

270. Vanderpool RC, Breheny PJ, Tiller PA, Huckelby CA, Edwards AD, Upchurch KD, et al. Implementation and evaluation of a school-based human papillomavirus vaccination program in rural Kentucky. American journal of preventive medicine. 2015;49(2):317-23.

271. Vernberg EM, Nelson TD, Fonagy P, Twemlow SW. Victimization, aggression, and visits to the school nurse for somatic complaints, illnesses, and physical injuries. Pediatrics. 2011;127(5):842-8.

272. Vernon TM, Conner JS, Shaw BS, Lampe JM, Doster ME. An evaluation of three techniques improving immunization levels in elementary schools. American journal of public health. 1976;66(5):457-60.

273. Vessey JA, O’Neill KM. Helping students with disabilities better address teasing and bullying situations: A MASNRN study. The Journal of School Nursing. 2011;27(2):139-48.

274. Wall RB. Tai chi and mindfulness-based stress reduction in a Boston public middle school. Journal of Pediatric Health Care. 2005;19(4):230-7.

275. Watson P. Back to school: no other health professional has such a unique role in the education community, says school nurse Paul Watson. Nursing Standard. 2008;22(20):64-5.

276. Weismuller PC, Grasska MA, Alexander M, White CG, Kramer P. Elementary school nurse interventions: Attendance and health outcomes. The Journal of School Nursing. 2007;23(2):111-8.

277. Wells N, Johnson R, Salyer S. Interdisciplinary collaboration. Clin Nurse Spec. 1998;12(4):161-8. Epub 1999/02/13. doi: 10.1097/00002800-199807000-00014. PubMed PMID: 9987223.

278. Werch CE, Carlson JM, Pappas DM, DiClemente CC. Brief nurse consultations for preventing alcohol use among urban school youth. Journal of School Health. 1996;66(9):335-8.

279. White MV, Hogue SL, Odom D, Cooney D, Bartsch J, Goss D, et al. Anaphylaxis in schools: Results of the EPIPEN4SCHOOLS survey combined analysis. Pediatric allergy, immunology, and pulmonology. 2016;29(3):149-54.

280. Whitmarsh J. School nurses' skills in sexual health education. Nursing standard (Royal College of Nursing (Great Britain): 1987). 1997;11(27):35-41.

281. Whitmore K. School refusal. Health visitor. 1988;61(11):349-51.

282. Wicklander MK. The United Kingdom National Healthy School Standard: A framework for strengthening the school nurse role. The Journal of school nursing. 2005;21(3):132-8.

283. Wiggs-Stayner KS, Purdy TR, Go GN, McLaughlin NC, Tryzynka PS, Sines JR, et al. The impact of mass school immunization on school attendance. J Sch Nurs. 2006;22(4):219-22. Epub 2006/07/22. doi: 10.1177/10598405050220040601. PubMed PMID: 16856776.

284. Williams AD, Warrington V. Get Fit Kids: a feasibility study of a pedometer-based walking program. Bariatric Nursing and Surgical Patient Care. 2011;6(3):139-43.

285. Wilson EL, Egger JR, Konty KJ, Paladini M, Weiss D, Nguyen TQ. Description of a school nurse visit syndromic surveillance system and comparison to emergency department visits, New York City. American journal of public health. 2014;104(1):e50-e6.

286. Wing R, Amanullah S, Jacobs E, Clark MA, Merritt C. Heads Up: Communication Is Key in School Nurses' Preparedness for Facilitating "Return to Learn" Following Concussion. Clin Pediatr (Phila). 2016;55(3):228-35. Epub 2015/07/02. doi: 10.1177/0009922815592879. PubMed PMID: 26130394.

287. Winkelstein ML. Teaching Pregnant Adolescents to Cope with Environmental Smoke: Culturally relevant materials and role playing are effective in a program for African American students. Initial results are promising. MCN: The American Journal of Maternal/Child Nursing. 1995;20(1):38-45.

288. Wong EM, Cheng MM. Effects of motivational interviewing to promote weight loss in obese children. Journal of Clinical Nursing. 2013;22(17-18):2519-30.

289. Wright K, Giger JN, Norris K, Suro Z. Impact of a nurse-directed, coordinated school health program to enhance physical activity behaviors and reduce body mass index among minority children: A parallel-group, randomized control trial. International Journal of Nursing Studies. 2013;50(6):727-37.

1. Experimental group [↑](#footnote-ref-1)
2. Glycosylated haemoglobin [↑](#footnote-ref-2)
